# Unsymmetrical Diaryl Borinium Cation: From Solvent-Dependent Radical Behavior to an Elusive Chloroborinium Intermediate

**DOI:** 10.1021/acs.inorgchem.6c03613

**Published:** 2026-08-28

**Authors:** Bo-An Chen, Hsi-Ching Tseng, Yi-Hung Liu, Yu-Jiang Lin, Liang-Yan Hsu, Ching-Wen Chiu

**Affiliations:** † Department of Chemistry, 33561 National Taiwan University, Taipei 10617, Taiwan; ‡ Instrumentation Center, National Taiwan University, Taipei 10617, Taiwan; § 71556 Institute of Atomic and Molecular Science, Academia Sinica, Taipei 106319, Taiwan

## Abstract

The diaryl borinium cation ([Ar_2_B]^+^), featuring two vacant p-orbitals at the boron center, represents one of the most reactive species among known boranylium cations. To harness its extreme electron deficiency, we utilized a sterically demanding 2,6-dimesitylphenyl (Dmp) substituent to stabilize an unsymmetrically substituted dicoordinate boron cation, [Dmp–B–Mes]^+^ ([**2**]^+^). We demonstrate that this structural asymmetry unlocks the unusual reactivity of borinium cations, ranging from distinct radical reactions to regioselective metathesis processes. The one-electron reduction of [**2**]^+^ generates a partially saturated 9-borafluorene radical 4^•^, which exhibits solvent-dependent decomposition pathways. In addition to this neutral radical reactivity, the incorporation of the Dmp group enables a highly regioselective B–C_Mes_ metathesis reaction with Et_3_SiH and ^*t*^BuCl. This strategy provides synthetic access to elusive hydrido- and chloroborinium intermediates. The existence of the transient [Dmp–B–Cl]^+^ intermediate ([**9**]^+^) was verified by fluoride-trapping experiments and supported by computational analysis, which revealed a stabilizing intramolecular borinium−π interaction. Overall, this study highlights the unique reactivity of unsymmetrical diaryl borinium cations and demonstrates the experimental accessibility to highly reactive borinium species bearing H and Cl substituents.

## Graphical abstract

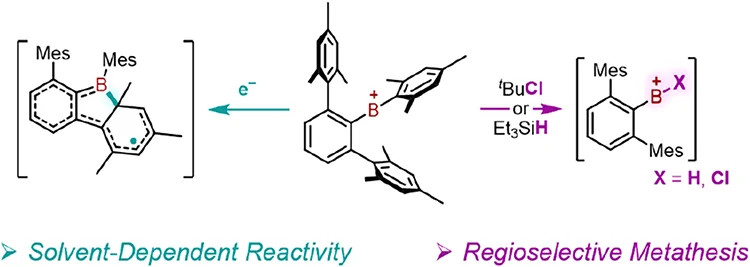

## Introduction

Because of their enhanced Lewis acidity, boron cations have been studied extensively over the past few decades. Among the three classes of boranylium ions, tetracoordinate boronium and tricoordinate borenium cations are relatively stable and have found widespread application in catalysis.^1^ In contrast, dicoordinate borinium cations, possessing only four valence electrons and two empty p-orbitals, are highly reactive and remain a synthetic challenge.

Early efforts toward isolable borinium cations relied on the incorporation of π-donating amino or imino substituents at boron, giving rise to diamido^2^ or diimido-borinium cations,^3^ such as [B­(N^*i*^Pr_2_)_2_]^+^ ([**I**]^+^) and [^*t*^Bu_3_PN–B–NP^*t*^Bu_3_]^+^ ([**II**]^+^) (Figure 1a). While these substituents provide substantial electronic stabilization through N → B π donation, they also diminish the Lewis acidity and reactivity of the boron center.^4^ In contrast, all-carbon-substituted borinium cations possess a much more electron-deficient boron center and undergo a wide range of bond activation reactions. The first example, the dimesitylborinium cation ([**III**]^+^, Figure 1a), was reported by Shoji and co-workers in 2014.^5^ Nearly all of its reactions proceed through B–C bond cleavage, highlighting the intrinsic instability of the C–B^+^–C linkage.^6^ The only exception is its reduction by lithium to afford a tetramesityl diborane(4).^7^ Despite their fundamental importance, borinium cations bearing hydride or halide substituents have remained largely unexplored. In particular, early studies by Nöth and co-workers on the putative halo-borinium cations [TMP–B–X]^+^ (X = F, Cl, Br, I) yielded inconclusive results, and the existence of free halo-borinium cations remains unclear (Figure 1b).^8^

**Figure 1:**
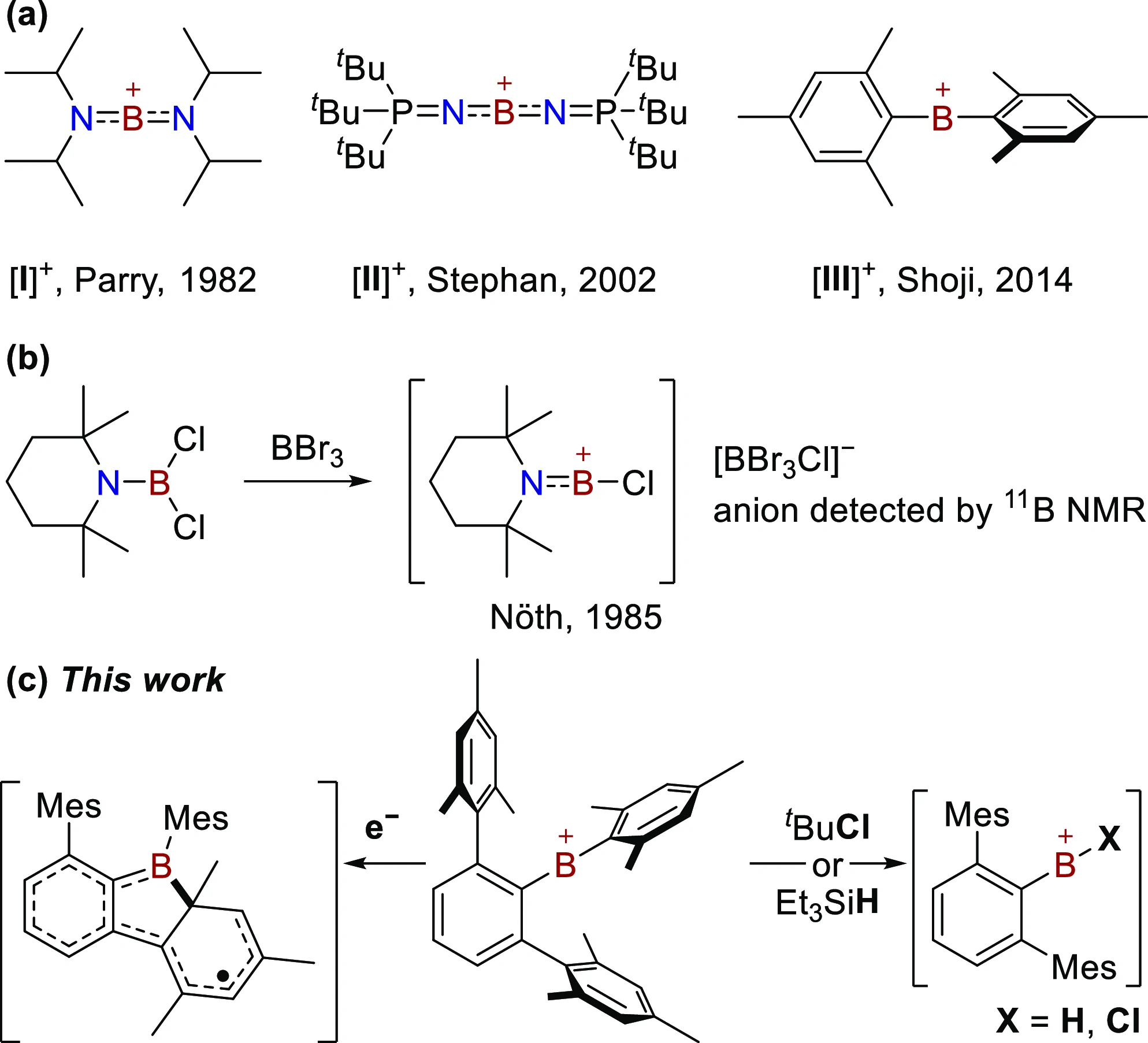
(a) Selected borinium cations. (b) Previous attempt to generate a chloroborinium species. (c) Illustrative scheme of the reaction presented in this work.

To harness the highly electron-deficient nature of diaryl borinium cations, we employed a sterically demanding *m*-terphenyl substituent, the 2,6-dimesitylphenyl group (Dmp), to provide both kinetic and thermodynamic stabilization at the dicoordinate boron center. In addition, the Dmp substituent can engage in intramolecular cation−π interactions, as evidenced by the crystal structure of [Dmp_2_Al]^+^, a heavy congener of diaryl borinium cations.^9^ Given that various *m*-terphenyl-stabilized low-coordinate complexes have shown remarkable stability and catalytic potential,^10^ we envisioned that the unique steric and electronic properties of the Dmp group could stabilize otherwise inaccessible reaction intermediates, thereby enabling mechanistic investigations of B–C bond cleavage processes in borinium cations. Herein, we report the synthesis and reactivity of an unsymmetrical diaryl borinium cation, [Dmp–B–Mes]^+^ ([**2**]^+^). We demonstrate that the Dmp substituent facilitates both solvent-dependent reduction chemistry and the experimental observation of a transient chloroborinium intermediate (Figure 1c).

## Results and Discussion

### Synthesis of the Targeted Borinium Salt

The targeted borinium salt [**2**]­[B­(C_6_F_5_)_4_] was obtained via chloride abstraction from the chloroborane precursor **1**,^5^ which was prepared by the reaction of Mes–BCl_2_ ^11^ and Dmp–Li in toluene (Figure 2a).^12^ The treatment of a colorless *ortho*-dichlorobenzene (*o*-DCB) solution of **1** with one equivalent of an arene- stabilized triethylsilylium salt ([Et_3_Si­(mes)]­[B­(C_6_F_5_)_4_])^13^ resulted in an immediate color change to golden yellow. In the ^1^H NMR spectrum of the reaction mixture, a new set of significantly downfield-shifted Dmp ligand signals emerged, consistent with the formation of a cationic species. Notably, the *para*-H resonance of the Dmp group shifted from 7.44 to 7.97 ppm. After all volatiles were removed *in vacuo*, the residue was washed with cyclohexane and *n*-pentane to afford [**2**]­[B­(C_6_F_5_)_4_] as a bright-yellow powder. Despite the steric protection provided by the bulky Dmp ligand, [**2**]^+^ still exhibited extremely high reactivity. [**2**]­[B­(C_6_F_5_)_4_] is insoluble in nonpolar hydrocarbons (*e*.*g*., alkanes, benzene, and toluene), reacts stoichiometrically with dichloromethane and diethyl ether, and initiates the polymerization of tetrahydrofuran. Consequently, [**2**]­[B­(C_6_F_5_)_4_] is only soluble and stable for several hours at room temperature in *o*-DCB and *ortho*-difluorobenzene (*o*-DFB). It gradually decomposed in solution within 24 h. Signals corresponding to B­(C_6_F_5_)_3_ were observed in the ^19^F NMR spectrum of the resulting dark purple solution, implying that the decomposition of [**2**]^+^ involved a reaction with the [B­(C_6_F_5_)_4_]^−^ anion.

**Figure 2:**
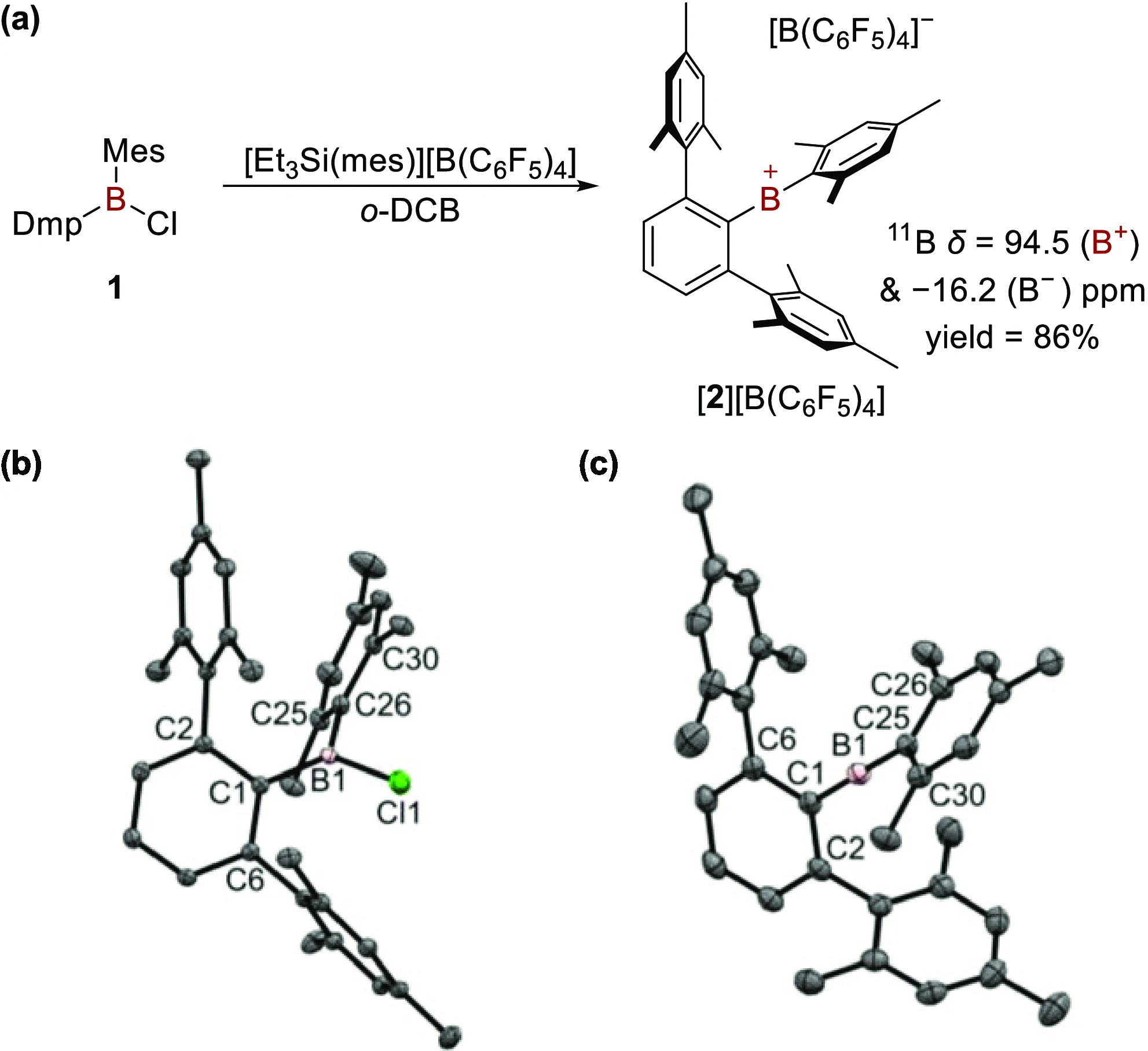
(a) Synthesis of [**2**]­[B­(C_6_F_5_)_4_]. (b) Thermal ellipsoid plot (50%) of **1**. (c) Thermal ellipsoid plot (50%) of [**2**]­[B­(C_6_F_5_)_4_]. Hydrogen atoms and counteranions are omitted for clarity.

Single-crystal X-ray diffraction (SC-XRD) analyses of **1** and [**2**]­[B­(C_6_F_5_)_4_] confirmed the molecular structures of both species (Figure 2b,c). The formation of the borinium cation is evidenced by the linear geometry of the boron center in [**2**]^+^ (∠C–B–C = 178.8°). Compared with the tricoordinate precursor **1** (B–C^Dmp^: 1.575 Å; B–C_Mes_: 1.570 Å), the B–C bonds in [**2**]^+^ are significantly shorter (B–C_Dmp_: 1.474 Å; B–C_Mes_: 1.457 Å), reflecting the hybridization change of boron from sp^2^ to sp and the increased B–C double-bond character. The conjugation between boron and arenes is further supported by the dihedral angle between the aryl rings (59.86°), which is notably smaller than that observed in [**III**]­[B­(C_6_F_5_)_4_] (89.05°). Unlike the structural distortions observed in [Dmp_2_Al]^+^, the flanking mesityl rings in [**2**]^+^ show no significant bending toward the boron center, suggesting that cation−π interactions do not have a significant structural influence in [**2**]^+^.

### Reactivity Studies

With [**2**]­[B­(C_6_F_5_)_4_] in hand, we performed a one-electron reduction of [**2**]^+^ to a diarylboryl radical (**3** ^•^) with decamethylcobaltocene (Cp*_2_Co) or potassium graphite (KC_8_) in benzene suspension (Figure 3a). Although the steric bulk of the Dmp substituent prevented **3** ^•^ from forming a B–B bonded dimer,^7^ the intramolecular addition of the boryl radical to one of the flanking mesityl rings transferred the spin density from boron to the aryl ring, affording the 9-borafluorene-containing radical (**4** ^•^). The formation of **4** ^•^ was supported by electron paramagnetic resonance (EPR) spectroscopy at 179 K. As shown in Figure 3c, an isotropic signal centered at *g* = 2.0038 was observed, albeit no clear hyperfine coupling could be identified to unambiguously confirm its electronic structure, and all attempts to isolate **4** ^•^ were unsuccessful because of its limited stability (see Supporting Information (SI) for detail discussion). Interestingly, **4** ^•^ underwent solvent-dependent decomposition. In an aromatic solvent (benzene), radical **4** ^•^ readily dimerized at room temperature to yield two diastereomers of a partially saturated 9-borafluorene dimer (**5**, Figure 3d). In contrast, in alkane solvents (cyclohexane/*n*-pentane = 1:1), a demethylated product, 1,9-dimesityl-5,7-dimethyl-9-borafluorene (**6**), was isolated and structurally characterized (Figure 3e). The release of methane was confirmed by performing the reaction in cyclohexane-*d*_12_ in a sealed tube, where CH_4_ and CH_3_D were unambiguously detected by selective band heteronuclear single quantum coherence spectroscopy (HSQC) (Figure S4).^14^ These findings established that the decomposition of boryl radical **3** ^•^ begins with intramolecular addition to a mesityl ring to generate the carbon-centered radical **4** ^•^, which either dimerizes to form **5** or abstracts a hydrogen atom to afford **6** via 1,2-elimination of methane (Figure 3a).

**Figure 3:**
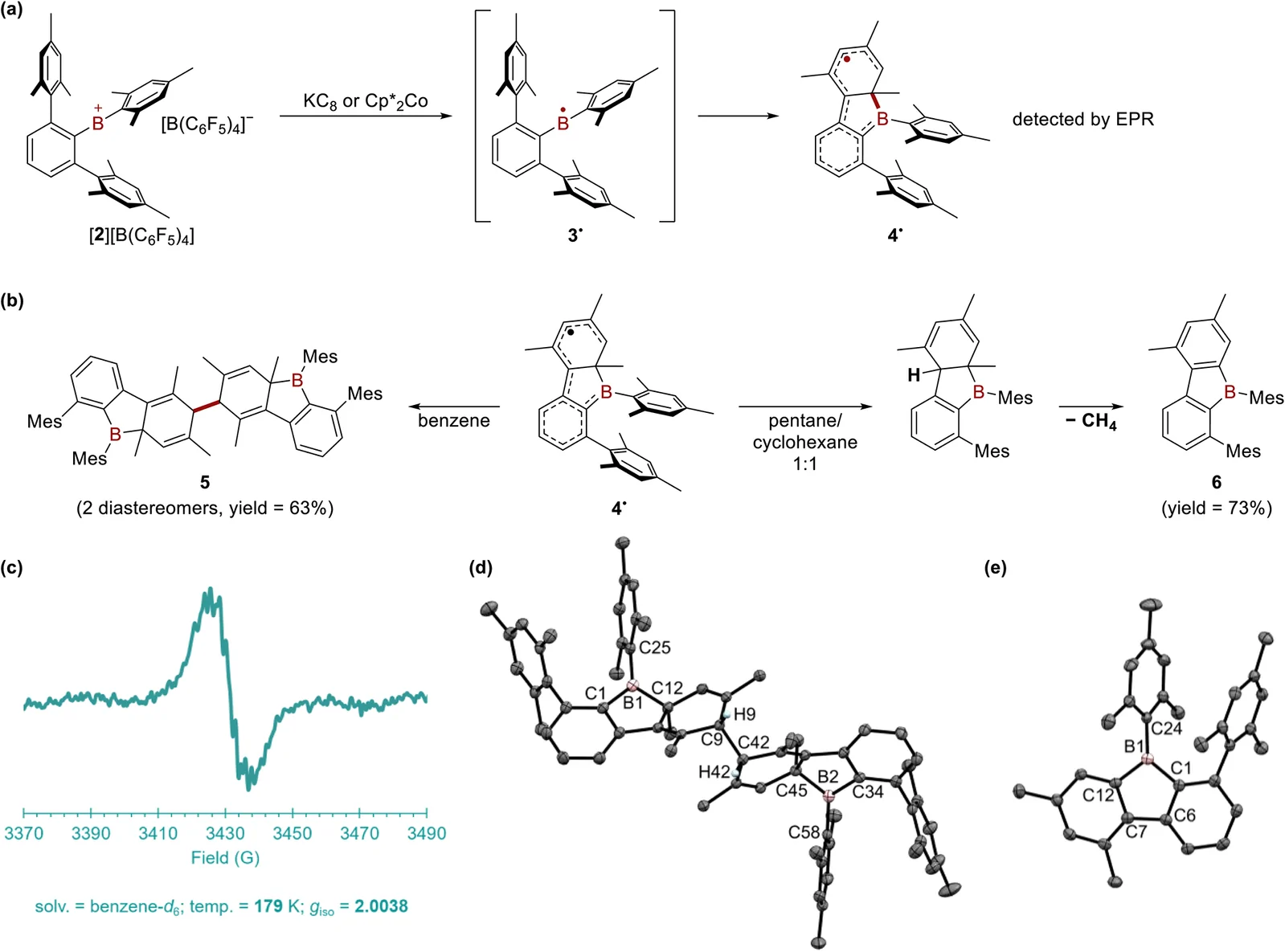
(a) Reduction reactions of [**2**]­[B­(C_6_F_5_)_4_] to generate radical **4** ^•^. (b) Different decomposition pathway of **4** ^•^ to generate **5** and **6**. (c) Low-temperature (179 K) EPR spectrum of radical **4** ^•^, prepared from the reaction mixture of [**2**]­[B­(C_6_F_5_)_4_] and Cp*_2_Co in benzene-*d*_6_. (d) Thermal ellipsoid plot (50%) of **5**. (e) Thermal ellipsoid plot (50%) of **6**. Some hydrogen atoms have been omitted for clarity.

To investigate the metathesis reactivity and regioselectivity of the unsymmetrical borinium cation, [**2**]­[B­(C_6_F_5_)_4_] was reacted with triethylsilane (Et_3_SiH) and *tert*-butyl chloride (^*t*^BuCl) in *o*-DFB at room temperature. Treatment of [**2**]­[B­(C_6_F_5_)_4_] with excess Et_3_SiH resulted in the exclusive cleavage of the B–C_Mes+_ bond, yielding dimeric hydridoborane [Dmp–BH­(μ-H)]_2_ (**7**)^15^ in 82% and [Et_3_Si–H–SiEt_3_]^+^, which was isolated as [Et_3_Si­(mes)]­[B­(C_6_F_5_)_4_], in 41% yield (Figure 4a). The formation of the Dmp–BH_2_ dimer is analogous to the reported reaction between [**III**]^+^ and Et_3_SiH, in which the resulting Mes–BH_2_ was isolated as a diboron cation, a borinium–borane adduct.^6c^ Likewise, the reaction of [**2**]­[B­(C_6_F_5_)_4_] with two equivalents of ^*t*^BuCl afforded Dmp–BCl_2_ (**8**),^16^ and a mesitylenium salt ([MesH_2_]­[B­(C_6_F_5_)_4_], Figure S16) in 98 and 52% yields, respectively (Figure 4b). These findings are significant, as the formation of **7** and **8** in high yields confirms the excellent regioselectivity of the metathesis reaction. The more sterically hindered and π-stabilizing Dmp ligand remains intact while the mesityl group is preferentially cleaved.

**Figure 4:**
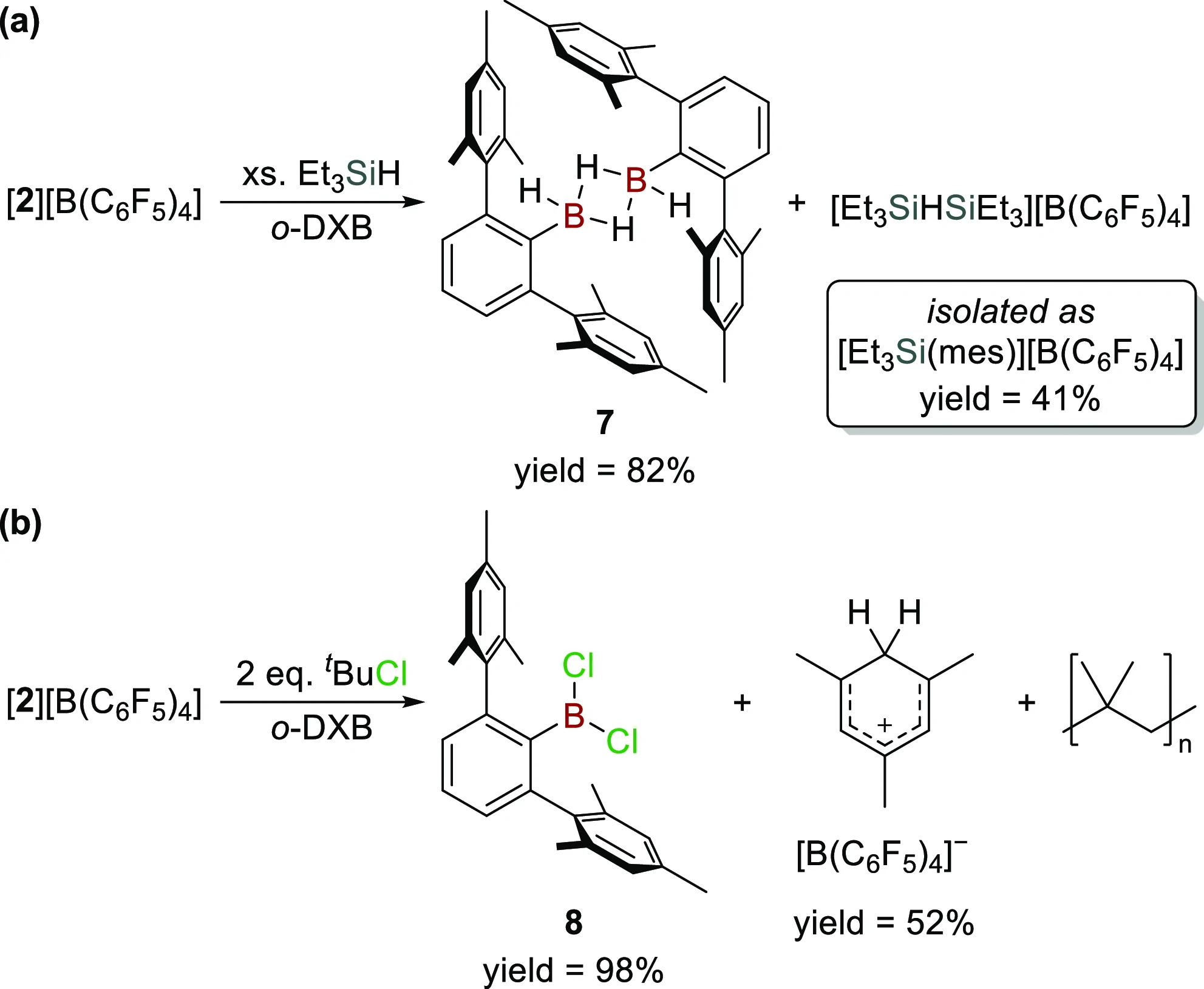
Metathesis reactions of [**2**]­[B­(C_6_F_5_)_4_] with (a) triethylsilane (Et_3_SiH) and (b) *tert*-butyl chloride (^*t*^BuCl). *o*-DXB stands for either *o*-DFB, *o*-DCB, or *o*-DCB-*d*_4_.

The isolation of silylium and mesitylenium cations provides strong evidence for the existence of highly Lewis acidic, transient intermediates, which we hypothesize to be the putative hydridoborinium [Dmp–B–H]^+^ and chloroborinium [Dmp–B–Cl]^+^ cations. The reaction pathway for forming **8** is proposed to proceed through a regioselective metathesis reaction of [**2**]^+^ and ^*t*^BuCl, yielding [Dmp–B–Cl]^+^ ([**9**]^+^), *iso*-butene, and mesitylene (Figure 5a). The subsequent reaction of [Dmp–B–Cl]^+^ with the second equivalent of ^*t*^BuCl afforded **8** and a *tert*-butyl cation, which then transfers its proton to mesitylene, generating *iso*-butene and mesitylenium. Under the reaction conditions, the generated *iso*-butene underwent polymerization, while the mesitylenium cation was isolated as single crystals (Figure S16).

**Figure 5:**
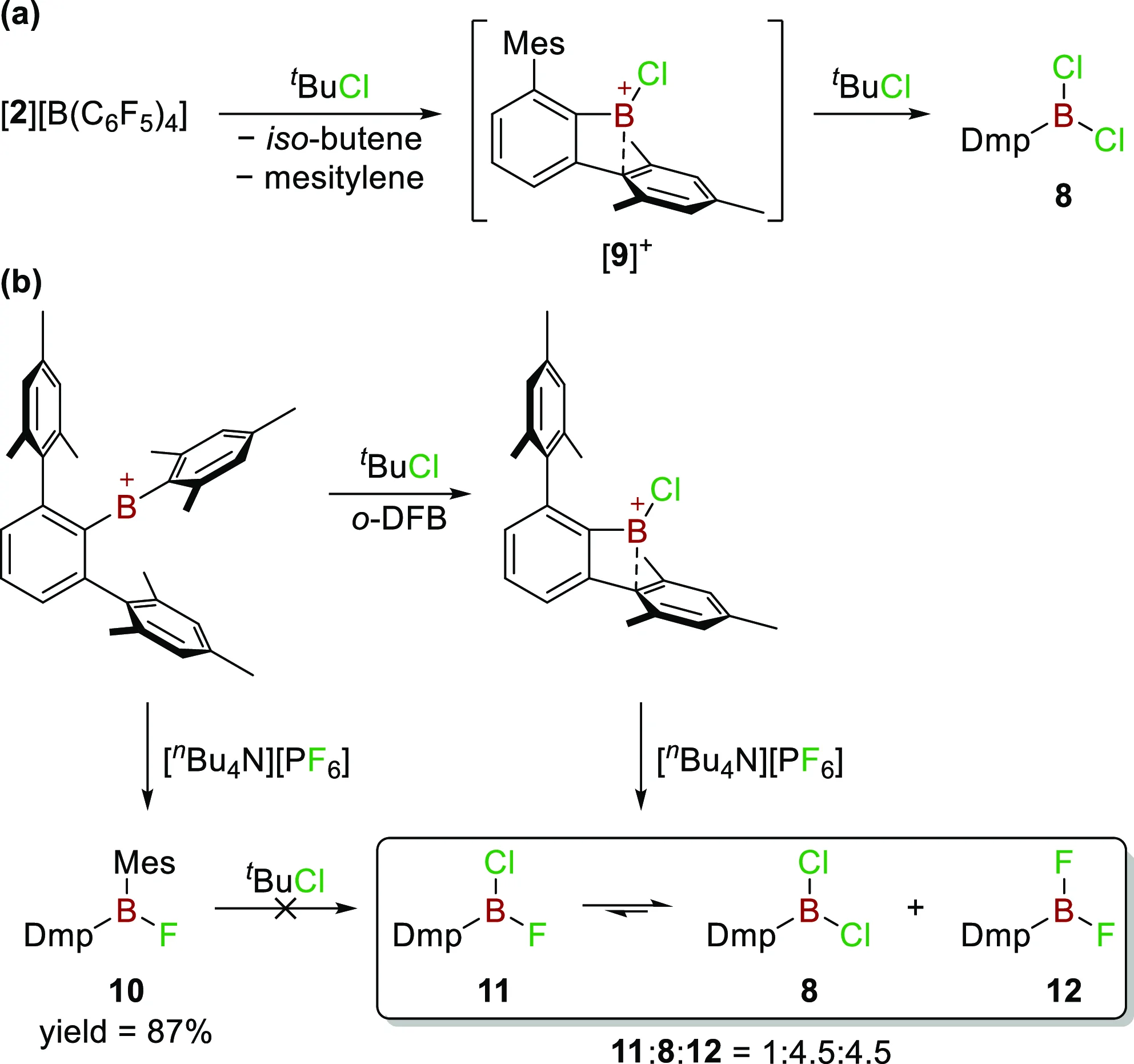
(a) Proposed reaction pathway for [**2**]^+^ to **8**. (b) Control and trapping experiments of the intermediate [Dmp–B–Cl]^+^ ([**9**]^+^) with [^*n*^Bu_4_N]­[PF_6_].

In an attempt to isolate [Dmp–B–Cl]^+^ ([**9**]^+^), [**2**]­[B­(C_6_F_5_)_4_] was reacted with ^*t*^BuCl in a 1:1 ratio. Upon addition of ^*t*^BuCl, an immediate color change from bright yellow to deep red was observed. The ^1^H NMR spectrum of [**9**]^+^ exhibited a new set of Dmp signals, which was tentatively assigned to the solvent-coordinated [Dmp–B–Cl]^+^ cation (*vide infra*). The same species can also be generated from the reaction between Dmp–BCl_2_ and [Et_3_Si­(mes)]^+^, further supporting the assignment of the intermediate to a chloroborinium species (Figure S5). As all attempts to crystallize this red intermediate were unsuccessful, we sought to trap the reactive species with fluoride.

The reaction of the putative [Dmp–B–Cl]^+^ intermediate with [^*n*^Bu_4_N]­[PF_6_] yielded a mixture of Dmp–BClF (**11**), Dmp–BCl_2_ (**8**), and Dmp–BF_2_ (**12**) in a 1:4.5:4.5 ratio, consistent with the generation and subsequent disproportionation of Dmp–BClF.^17^ Under the same reaction conditions, the addition of [^*n*^Bu_4_N]­[PF_6_] to [**2**]­[B­(C_6_F_5_)_4_] resulted in the formation of diaryl fluoroborane Dmp­(Mes)­BF (**10**) in a quantitative yield (Figure 5b). These two fluoride addition reactions provide experimental support for the existence of a Dmp-stabilized chloroborinium intermediate.

### Computational Results

The observed regioselectivity of the metathesis reaction between [**2**]^+^ and ^*t*^BuCl was investigated via quantum chemical computations performed with the ORCA 6.1.0 software.^18^ Geometry optimizations were conducted using density functional theory (DFT) at the r^2^SCAN0-D4-gCP/def2-TZVP level of theory.^19^ The resulting transition states and intermediates were verified with the intrinsic reaction coordinate (IRC) analysis.^20^ To improve the energetic accuracy, single-point energies were refined at the DLPNO-CCSD­(T)^21^/cc-pVTZ^22^ level, and the solvation energies in *o*-DCB were calculated using the COSMO-RS implicit solvation model.^23^

As summarized in Figure 6, the metathesis reaction is categorized into three steps: (i) coordination of ^*t*^BuCl at the boron center, (ii) protonation of the arene *ipso*-carbon, and (iii) dissociation of the arene. Two competitive pathways were computed: pathway A involving B–C_Mes_ cleavage (cyan trace) and pathway B involving B–C^Dmp^ cleavage (red trace). Regioselectivity is determined during the protonation step (TS2), which exhibits a significant ΔΔ^‡^ *G*° of 25.47 kcal/mol. This substantial energy difference is attributed to the steric hindrance of the *m-*terphenyl group, which effectively prohibits protonation at the *ipso*-carbon of the Dmp group. The resulting arene-coordinated chloroborinium cation ([*anti*-**Int2a**]^+^) undergoes subsequent arene dissociation and chloride abstraction to yield an isolated dichloroborane (Dmp–BCl_2_). Interestingly, the dissociation of mesitylene from [*anti*-**Int2a**]^+^ exhibits an energy barrier of 17.78 kcal/mol, which is higher than that for the coordination of ^*t*^BuCl at the boron center (12.01 kcal/mol, [**TS1**]^+^, kinetic effective molarity = 5.91·10^–5^ M). This energy profile is consistent with the experimental observation that [**2**]^+^ can be fully consumed with one equivalent of ^*t*^BuCl to afford a mesitylene-coordinated [Dmp–B–Cl]^+^ intermediate, which is sufficiently long-lived for spectroscopic detection and fluoride trapping before being transformed into the dichloroborane.

**Figure 6:**
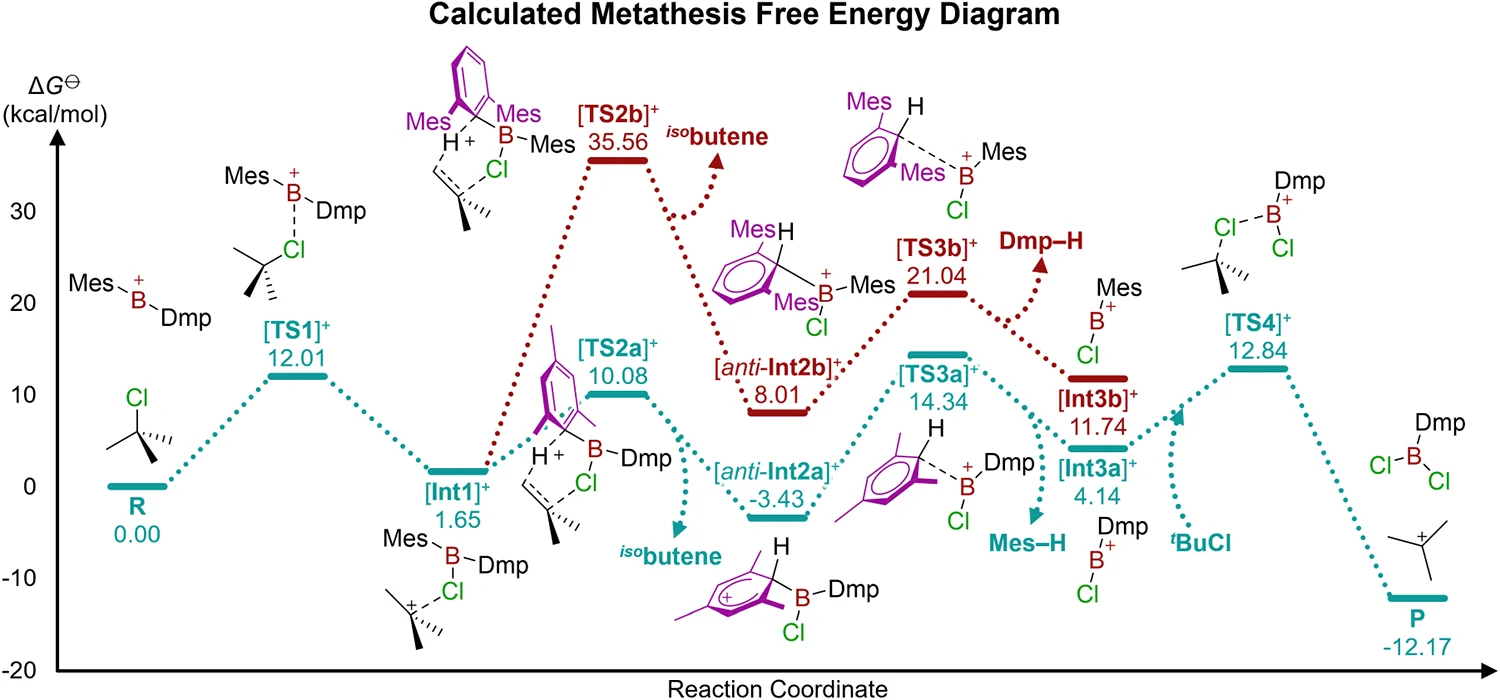
Potential free energy diagram for the metathesis reaction between [**2**]^+^ and ^*t*^BuCl.

The stabilizing effect of the Dmp group on the chloroborinium cation was also investigated computationally. Compared with the linear geometry calculated for [Mes–B–Cl]^+^, a significant distortion of the *m*-terphenyl group toward the boron center was observed in the optimized structure of [Dmp–B–Cl]^+^. The existence of a borinium−π interaction is supported by electron localization function (ELF) analysis,^24^ which reveals a monosynaptic basin on the *ipso*-carbon oriented toward the boron atom (Figure S24). This interaction is further corroborated by the quantum theory of atoms in molecules (QTAIM) analysis,^25^ performed using the Multiwfn program (ver. 2026.1.12).^26^ A bond critical point (BCP) between the boron atom and the *ipso*-carbon of the flanking mesityl ring was identified (Figures S22 and S23). Notably, the relatively high electron density (ρ = 0.09560 e·a_0_^–3^) with a negative Laplacian value (∇^2^ρ = −0.11944 e·a_0_^–5^) suggests the presence of a substantial degree of dative covalent bonding interaction, which accounts for the energy of [Dmp–B–Cl]^+^ being 7.59 kcal/mol lower in energy than that of its mesityl analogue [Mes–B–Cl]^+^.

Finally, the Lewis acidity of [**2**]^+^ and the putative chloroborinium intermediate [**9**]^+^ were evaluated via fluoride (FIA),^27^ hydride (HIA), and chloride ion affinity (CIA)^28^ calculations (Table 1). While [**2**]^+^ possesses slightly lower anion affinities than [**III**]^+^, the incorporation of the chloride substituent significantly enhances the Lewis acidity of the boron center. Furthermore, the observed reactivity between borinium cations and ^*t*^BuCl can also be rationalized by the calculated CIA values. While the CIA of [**2**]^+^ (582.0 kJ/mol) is too low to abstract a chloride from ^*t*^BuCl (CIA of [Me_3_C]^+^ = 662.3 kJ/mol), [**9**]^+^ exhibits a comparable CIA (662.0 kJ/mol) to that of the *tert*-butyl cation. The increased Lewis acidity, coupled with a large gain in entropy, facilitates chloride abstraction from ^*t*^BuCl to yield the observed mixture of Dmp–BCl_2_ and mesitylenium cation.

**Table 1:** Computed Gas-Phase Anion Affinity Values for [III]+, [2]+, and [9]+ (in kJ/mol)

	[**III**]^+^	[**2**]^+^	[**9**]^+^
FIA	834.6	811.1	891.2
HIA	800	776	859
CIA	606	582	662

## Conclusions

In this work, we reported the synthesis and isolation of an *m*-terphenyl-substituted unsymmetrical borinium cation, [Dmp–B–Mes]­[B­(C_6_F_5_)_4_] ([**2**]­[B­(C_6_F_5_)_4_]), and investigated its reduction and metathesis reactions. The neutral diarylboryl radical [Dmp–B–Mes]^•^, generated from the one-electron reduction of [**2**]^+^, undergoes immediate ligand activation to yield partially saturated 9-borafluorene radical **4** ^•^. This carbon-centered radical subsequently dimerizes in arene solvents or abstracts a hydrogen atom from alkane solvents to afford a 9-borafluorene molecule via 1,2-elimination of methane.

Furthermore, treatment of [**2**]^+^ with Et_3_SiH and ^*t*^BuCl promotes a regioselective metathesis reaction, yielding highly Lewis acidic *m*-terphenyl substituted hydrido- and chloroborinium intermediates, respectively. A computational investigation into the *t*BuCl-induced selective B–C_Mes_ bond cleavage suggests that the regioselectivity arises from the steric protection of the Dmp group and stabilization of the resulting chloroborinium cation ([**9**]^+^) through an intramolecular borinium−π interaction. Overall, these studies on *m-*terphenyl-substituted borinium cations unveil a unique chemical transformation of the diaryl borinium cations and provide insight into the chemistry of highly reactive, elusive hydrido- and chloroborinium cations.

## Experimental Section

### General Considerations

All manipulations were carried out with either the Schlenk technique or in a glovebox under a nitrogen or argon atmosphere. Anhydrous toluene, benzene-*d*_6_, mesitylene, *n*-hexane, *n*-pentane, cyclohexane, diethyl ether, and tetrahydrofuran (THF) were dried over Na/K alloy/benzophenone ketyl, distilled under nitrogen, and stored over 4 Å molecular sieves. Acetonitrile (ACN), dichloromethane (DCM), and *ortho*-difluorobenzene (*o*-DFB) were dried over CaH_2_, distilled under nitrogen, and stored over 4 Å molecular sieves. *ortho*-Dichlorobenzene (*o*-DCB), *o*-DCB-*d*_4_, DCM-*d*_2_, and cyclohexane-*d*_12_ were degassed by the freeze–pump–thaw cycle, passed through activated alumina, and stored over 4 Å molecular sieves. Triethylsilane (Et_3_SiH), triphenylmethylium tetrakis­(pentafluorophenyl)­borate ([Ph_3_C]­[B­(C_6_F_5_)_4_]), and decamethylcobaltocene (Cp*_2_Co) were purchased and used without further purification. Potassium graphite (KC_8_), (2,6-dimesitylphenyl)­lithium (Dmp–Li),^12^ dichloromesitylborane,^11^ and arene-coordinated triethylsilylium cation ([Et_3_Si­(mes)]­[B­(C_6_F_5_)_4_])^5,13^ were synthesized according to published procedures. SC-XRD was performed on a Bruker AXS D8 VENTURE at 100 K. NMR spectra were recorded on Bruker AVIII HD-400 (^1^H: 400.13 MHz, ^11^B: 128.38 MHz, ^13^C: 100.61 MHz, ^19^F: 376.50 MHz), Bruker AVIII-500 (^1^H: 500.17 MHz, ^11^B: 160.47 MHz, ^13^C: 125.77 MHz, ^19^F: 470.61 MHz) or Bruker AVIII-800 (^1^H: 800.22 MHz, ^13^C: 201.22 MHz) spectrometers. Chemical shifts (δ) were reported in ppm and were referenced to the signals of residual solvents for ^1^H (C_6_D_5_H: 7.16 ppm; *ortho*-C_6_D_3_HCl_2_: 7.19 and 6.93 ppm; C_6_D_11_H: 1.38 ppm), deuterated solvents for ^13^C (C_6_D_6_: 128.06 ppm; *o*-DCB-*d*_4_: 132.28, 130.03, and 127.28 ppm; C_6_D_12_: 26.43 ppm), and external standards for ^11^B and ^19^F (vs. BF_3_•OEt_2_ and CFCl_3_ = 0 ppm, respectively). EPR spectra were recorded on a Bruker EMXnano spectrometer (0.3162 mW, attenuation of 25 dB, modulation amplitude of 1.000 G, modulation frequency of 100.00 kHz, time constant of 1.28 ms, and sweep time of 30.0 s). EPR simulation was performed using EasySpin 5.2.36.^29^

### Safety Statement

**Caution!** *Potassium graphite is extremely reactive to moisture in the air and potentially pyrophoric; therefore, it should be handled with care*.

### Synthesis of Chloro­(2,6-dimesitylphenyl)­(mesityl)­borane (**1**)

To a rapidly stirred solution of Dmp–Li (2.20 g, 6.87 mmol) in toluene (100 mL), dichloromesitylborane (1.50 g, 7.47 mmol, 1.09 equiv) was added dropwise at room temperature. The resulting white suspension was stirred for 24 h before filtration. The filtrate was concentrated *in vacuo*, and the solid residue was washed with cold *n*-pentane (10 mL, −35 °C) to afford **1** as a white powder in 72% yield (2.36 g, 4.93 mmol). Single crystals of **1** suitable for SC-XRD analysis were obtained by the slow evaporation of an *n*-hexane solution of borane at room temperature under argon.

#### ^1^H NMR (500.17 MHz, Benzene-*d*_6_, 298 K)

δ (ppm) 7.19 (t, ^3^ *J*_H,H_ = 7.6 Hz, 1H; Dmp-*para*-C_6_ ***H***_3_), 6.94 (d, ^3^ *J*_H,H_ = 7.6 Hz, 2H; Dmp-*meta*-C_6_ ***H***_3_), 6.70 (s, 4H; Dmp-Mes-*meta*-C_6_ ***H***_2_), 6.38 (s, 2H, B-Mes-*meta*-C_6_ ***H***_2_), 2.17 (s, 6H; Dmp-Mes-*para*-C ***H***_3_), 2.09 (s, 12H; Dmp-Mes-*ortho*-C ***H***_3_), 2.05 (s, 3H; B-Mes-*para*-C ***H***_3_), 2.03 (s, 6H; B-Mes-*ortho*-C ***H***_3_).

#### ^11^B NMR (160.47 MHz, Benzene-*d*_6_, 298 K)

δ (ppm) 70.9 (br).

#### ^13^C­{^1^H} NMR (125.77 MHz, Benzene-*d*_6_, 298 K)

δ (ppm) 149.28 (Dmp-*ortho-* **C**_6_H_3_), 140.85 (br, B-Mes-*ipso-* **C**_6_H_2_), 140.61 (Dmp-Mes-*ipso-* **C**_6_H_2_), 140.09 (br, Dmp-*ipso-* **C**_6_H_3_), 138.43 (B-Mes-*ortho-* **C**_6_H_2_), 137.79 (B-Mes-*para-* **C**_6_H_2_), 136.27 (Dmp-Mes-*para-* **C**_6_H_2_), 135.98 (Dmp-Mes-*ortho-* **C**_6_H_2_), 131.99 (Dmp-*para-* **C**_6_H_3_), 130.11 (Dmp-*meta-* **C**_6_H_3_), 128.32 (Dmp-Mes-*meta-* **C**_6_H_2_), 127.89 (B-Mes-*meta-* **C**_6_H_2_), 24.26 (B-Mes-*ortho-* **C**H_3_), 21.67 (Dmp-Mes-*ortho-* **C**H_3_), 21.09 (Dmp-Mes-*para-* **C**H_3_ and B-Mes-*para-* **C**H_3_).

### Synthesis of the Borinium Salt [**2**]­[B­(C_6_F_5_)_4_]

To a rapidly stirred solution of **1** (0.0958 g, 0.200 mmol) in *o*-DCB (0.5 mL), a solution of [Et_3_Si­(mes)]­[B­(C_6_F_5_)_4_] (0.1830 g, 0.200 mmol) in *o*-DCB (0.5 mL) was slowly added at room temperature. The resulting golden-yellow solution was immediately subjected to dynamic vacuum for 15 min to remove toluene and chlorotriethylsilane. The addition of *n*-pentane (20 mL) to the orange oily crude product, followed by manual stirring with a spatula, induced the gradual precipitation of [**2**]­[B­(C_6_F_5_)_4_] as yellow chunks. The crude solid was washed thoroughly with cyclohexane (15 mL) and then with *n*-pentane (15 mL × 2) and dried *in vacuo* to give the purified borinium salt in 86% yield (0.192 g, 0.171 mmol). Notably, [**2**]­[B­(C_6_F_5_)_4_], isolated as a bright-yellow powder, showed signs of decomposition in *o*-DCB solution after 2 h at room temperature and decomposed completely overnight. Single crystals of [**2**]­[B­(C_6_F_5_)_4_] suitable for SC-XRD analysis were obtained by the slow diffusion of *n*-pentane into an *o*-DCB solution of the borinium salt at −35 °C under argon for 2 d.

#### ^1^H NMR (500.17 MHz, *o*-DCB-*d*_4_, 263 K)

δ (ppm) 7.94 (t, ^3^ *J*_H,H_ = 7.7 Hz, 1H; Dmp-*para*-C_6_ ***H***_3_), 7.33 (d, ^3^ *J*_H,H_ = 7.7 Hz, 2H; Dmp-*meta*-C_6_ ***H***_3_), 6.69 (s, 4H; Dmp-Mes-*meta*-C_6_ ***H***_2_), 6.25 (s, 2H; B-Mes-*meta*-C_6_ ***H***_2_), 2.04 (s, 6H; Dmp-Mes-*para*-C ***H***_3_), 2.01 (s, 18H; B-Mes-*ortho*-C ***H***_3_, and Dmp-Mes-*ortho*-C ***H***_3_), 1.64 (s, 3H; B-Mes-*para*-C ***H***_3_).

#### ^11^B NMR (128.38 MHz, *o*-DCB-*d*_4_, 298 K)

δ (ppm) 94.5 (br, [Dmp ***B*** Mes]^+^), −16.2 (s; [***B*** (C_6_F_5_)_4_]^−^).

#### ^13^C­{^1^H} NMR (125.77 MHz, *o*-DCB-*d*_4_, 263 K)

δ (ppm) 158.19 (Dmp-*ortho-* **C**_6_H_3_), 157.13 (B-Mes-*para-* **C**_6_H_2_), 154.56 (B-Mes-*ortho-* **C**_6_H_2_), 148.66 (d, ^1^ *J*_C,F_ = 240 Hz; *ortho-* **C**_6_F_5_), 143.66 (Dmp-*para-* **C**_6_H_3_), 140.56 (Dmp-Mes-*para-* **C**_6_H_2_), 138.57 (dt, ^1^ *J*_C,F_ = 234 Hz, ^2^ *J*_C,F_ = 12.1 Hz; *para-* **C**_6_F_5_), 136.63 (d, ^1^ *J*_C,F_ = 234 Hz; *meta-* **C**_6_F_5_), 135.69 (Dmp-Mes-*ortho-* **C**_6_H_2_), 133.54 (Dmp-Mes-*ipso-* **C**_6_H_2_), 130.27 (Dmp-*meta-* **C**_6_H_3_), 129.65 (B-Mes-*meta-* **C**_6_H_2_), 129.44 (Dmp-Mes-*meta-* **C**_6_H_2_), 125.39 (Dmp-*ipso-* **C**_6_H_3_), 124.41 (br; *ipso-* **C**_6_F_5_), 117.46 (B-Mes-*ipso-* **C**_6_H_2_), 23.00 (B-Mes-*ortho-* **C**H_3_), 22.49 (B-Mes-*para-* **C**H_3_), 20.99 (Dmp-Mes-*para-* **C**H_3_), 20.74 (Dmp-Mes-*ortho-* **C**H_3_).

#### ^19^F­{^1^H} NMR (470.63 MHz, *o*-DCB-*d*_4_, 263 K)

δ (ppm) −131.89 (m, 2F; *ortho*-C_6_ ***F***_5_), −161.67 (t, ^3^ *J*_F,F_ = 20.8 Hz, 1F; *para*-C_6_ ***F***_5_), −165.57 (m, 2F; *meta*-C_6_ ***F***_5_).

### Synthesis of **5** from the Reduction of [**2**]­[B­(C_6_F_5_)_4_] in an Aromatic Solvent

A rapidly stirred suspension of [**2**]­[B­(C_6_F_5_)_4_] (0.0780 g, 0.0695 mmol) in arene solvent (benzene, benzene-*d*_6_, mesitylene, or toluene; 1.0 mL) was treated with an equimolar amount of Cp*_2_Co (0.0228 g, 0.0696 mmol) or excess KC_8_ (0.0260 g, 0.192 mmol). The reaction mixture was stirred for 1 h and then filtered. All volatiles in the yellow filtrate were removed *in vacuo*, and the light-yellow solid residue was washed with acetonitrile (1 mL × 3) and redissolved in *n*-hexane. Removal of the solvent *in vacuo* afforded a mixture of isomers of **5** in 63% yield (0.0194 g, 0.0219 mmol) as a white powder. A small amount of crystalline *rac*-**5a** suitable for SC-XRD analysis was obtained by the slow evaporation of an *n*-pentane solution at room temperature. Although the two isomers exhibited different solubilities in acetonitrile and their ratios varied during purification, all attempts to completely separate the mixture and to obtain crystalline *meso*-**5b** were unsuccessful.

#### ^1^H NMR (500.17 MHz, Benzene-*d*_6_, 298 K)

δ (ppm) first isomer: 7.71 (d, ^3^ *J*_H,H_ = 7.8 Hz, 2H), 7.39 (t, ^3^ *J*_H,H_ = 7.6 Hz, 2H), 6.93 (d, ^3^ *J*_H,H_ = 7.2 Hz, 2H), 6.74 (s, 2H), 6.66 (s, 2H), 6.47 (s, 2H), 6.36 (s, 2H), 5.89 (s, 2H), 3.28 (s, 2H), 2.29 (s, 6H), 2.16 (s, 6H), 2.12 (s, 6H), 2.01 (s, 6H), 1.96 (s, 6H), 1.88 (s, 6H), 1.83 (s, 6H), 1.75 (s, 6H), 1.59 (s, 6H); second isomer: 7.61 (d, ^3^ *J*_H,H_ = 7.8 Hz, 2H), 7.40 (t, ^3^ *J*_H,H_ = 7.9 Hz, 2H), 6.96 (d, ^3^ *J*_H,H_ = 7.6 Hz, 2H), 6.76 (s, 2H), 6.66 (s, 2H), 6.45 (s, 2H), 6.37 (s, 2H), 5.84 (s, 2H), 3.27 (s, 2H), 2.30 (s, 6H), 2.16 (s, 6H), 2.13 (s, 6H), 2.06 (s, 6H), 2.02 (s, 6H), 1.83 (s, 6H), 1.79 (s, 6H), 1.76 (s, 6H), 1.51 (s, 6H).

#### ^11^B NMR (128.38 MHz, Benzene-*d*_6_, 298 K)

δ (ppm) 80.1 (br).

### Synthesis of **6** from the Reduction of [**2**]­[B­(C_6_F_5_)_4_] in *n*-Pentane/Cyclohexane

A rapidly stirred suspension of [**2**]­[B­(C_6_F_5_)_4_] (0.0600 g, 0.0535 mmol) in *n*-pentane/cyclohexane (1:1 v/v, 4.0 mL) was treated with excess KC_8_ (0.0220 g, 0.163 mmol). The reaction mixture was stirred for 10 d before filtration. All volatiles were removed *in vacuo*, and the residue was redissolved in toluene (0.05 mL). Crystals of borafluorene **6** suitable for SC-XRD analysis were obtained by cooling the toluene solution at −35 °C under argon, affording **6** in 73% crystalline yield (0.0167 g, 0.0390 mmol).

#### ^1^H NMR (500.17 MHz, Benzene-*d*_6_, 298 K)

δ (ppm) 7.40 (dd, ^3^ *J*_H,H_ = 7.6 Hz, ^4^ *J*_H,H_ = 0.7 Hz, 1H; borafluorene-C4-***H***), 7.16 (t, ^3^ *J*_H,H_ = 7.6, 1H; borafluorene-C3-***H***), 7.07 (br, 1H; borafluorene-C8-***H***), 6.78 (dd, ^3^ *J*_H,H_ = 7.6 Hz, ^4^ *J*_H,H_ = 0.7 Hz, 1H; borafluorene-C2-***H***), 6.70 (m, 1H; borafluorene-C6-***H***), 6.56 (s, 2H; B-Mes-*meta*-C_6_ ***H***_2_), 6.53 (s, 2H; borafluorene-C1-Mes-*meta*-C_6_ ***H***_2_), 2.30 (s, 3H; borafluorene-C5-C ***H***_3_), 2.17 (s, 3H; B-Mes-*para*-C ***H***_3_), 2.08 (s, 3H; borafluorene-C1-Mes-*para*-C ***H***_3_), 2.04 (s, 12H; B-Mes-*ortho*-C ***H***_3_ and borafluorene-C1-Mes-*ortho*-C ***H***_3_), 1.92 (s, 3H; borafluorene-C7-C ***H***_3_).

#### ^11^B NMR (128.38 MHz, Benzene-*d*_6_, 298 K)

δ (ppm) 71.6 (br).

#### ^13^C­{^1^H} NMR (125.77 MHz, Benzene-*d*_6_, 298 K)

δ (ppm) 156.98 (borafluorene-***C*** 4a), 150.36 (borafluorene-***C*** 1), 148.44 (borafluorene-***C*** 4b), 144.60 (br, borafluorene-***C*** 8a), 142.74 (br, borafluorene-***C*** 9a), 138.94 (borafluorene-***C*** 6), 138.45 (borafluorene-C1-Mes-*ipso-* **C**_6_H_2_), 138.07 (br, B-Mes-*ipso-* **C**_6_H_2_), 137.96 (borafluorene-***C*** 7), 137.31 (B-Mes-*ortho-* **C**_6_H_2_), 136.47 (B-Mes-*para-* **C**_6_H_2_), 135.97 (borafluorene-C1-Mes-*para-* **C**_6_H_2_), 135.07 (borafluorene-C1-Mes-*ortho-* **C**_6_H_2_), 134.89 (borafluorene-***C*** 3), 134.29 (borafluorene-***C*** 8), 132.73 (borafluorene-***C*** 5), 129.95 (borafluorene-***C*** 2), 127.61 (borafluorene-C1-Mes-*meta-* **C**_6_H_2_), 126.75 (B-Mes-*meta-* **C**_6_H_2_), 122.40 (borafluorene-***C*** 4), 23.36 (B-Mes-*ortho-* **C**H_3_), 21.35 (borafluorene-C5-***C*** H_3_), 21.28 (B-Mes-*para-* **C**H_3_), 21.24 (borafluorene-C1-Mes-*ortho-* **C**H_3_), 21.02 (borafluorene-C1-Mes-*para-* **C**H_3_), 20.74 (borafluorene-C7-***C*** H_3_).

### Synthesis of **7** and [Et_3_Si­(mes)]­[B­(C_6_F_5_)_4_] from the Reaction of [**2**]­[B­(C_6_F_5_)_4_] and Triethylsilane

A solution of [**2**]­[B­(C_6_F_5_)_4_] (45.0 mg, 0.0401 mmol) in *o*-DCB-*d*_4_ or *o*-DFB (1 mL) was treated with an excess of triethylsilane (64.0 μL, 10 equiv) and stirred for 30 min. The reaction mixture was then placed *in vacuo* to remove all volatiles and extracted with *n*-pentane. The collected *n*-pentane solution was filtered and concentrated to afford [Dmp–BH­(μ-H)]_2_ (**7**) as an off-white solid in 82% yield (10.7 mg, 0.0328 mmol). The remaining solid residue was washed with mesitylene and *n*-pentane to afford [Et_3_Si­(mes)]­[B­(C_6_F_5_)_4_] as an off-white powder in 41% yield (15.0 mg, 0.0164 mmol).

#### [Dmp–BH­(μ-H)]_2_ (**7**)

##### ^1^H NMR (400.13 MHz, Benzene-*d*_6_, 298 K)

δ (ppm) 7.15 (t, ^3^ *J*_H,H_ = 7.6 Hz, 2H; *para*-C_6_ ***H***_3_), 6.85 (d, ^3^ *J*_H,H_ = 7.6 Hz, 4H; *meta*-C_6_ ***H***_3_), 6.83 (s, 8H; Dmp-Mes-*meta*-Ar-***H***), 3.41 (br, 2H; terminal B-***H***), 2.24 (s, 12H; Dmp-Mes-*para*-C ***H***_3_), 1.81 (s, 24H; Dmp-Mes-*ortho*-C ***H***_3_); the μ-H signal was covered by trace impurities.

##### ^11^B NMR (128.38 MHz, Benzene-*d*_6_, 298 K)

δ (ppm) 18.0 (s, br).

#### [Et_3_Si­(mesitylene)]­[B­(C_6_F_5_)_4_]

##### ^1^H NMR (500.17 MHz, *o*-DCB-*d*_4_, 298 K)

δ (ppm) 6.55 (s, 3H; mesitylene-C ***H***), 2.19 (s, 9H; mesitylene-C ***H***_3_), 0.72 (t, ^3^ *J*_H,H_ = 7.4 Hz, 9H; SiCH_2_C ***H***_3_), 0.58 (q, ^3^ *J*_H,H_ = 7.4 Hz, 6H; SiC ***H***_2_CH_3_).

### Synthesis of **8** and [MesH_2_]­[B­(C_6_F_5_)_4_] from the Reaction of [**2**]­[B­(C_6_F_5_)_4_] and *tert*-Butyl Chloride

A solution of [**2**]­[B­(C_6_F_5_)_4_] (45.0 mg, 0.0401 mmol) in *o*-DCB-*d*_4_ or *o*-DFB (1 mL) was added to 2 equiv of *tert*-butyl chloride (9.0 μL, 0.083 mmol) and stirred for 30 min. The reaction mixture was then placed *in vacuo* to remove all volatiles and extracted with *n*-pentane. The collected *n*-pentane solution was filtered and concentrated to afford Dmp–BCl_2_ (**8**) as a white solid in 98% yield (15.6 mg, 0.0395 mmol). The remaining solid residue was redissolved in *o*-DFB and layered with *n*-pentane at −35 °C to afford crystalline [MesH_2_]­[B­(C_6_F_5_)_4_] in 52% yield (16.8 mg, 0.0210 mmol).

#### Dmp–BCl_2_ (**8**)

##### ^1^H NMR (400.13 MHz, Benzene-*d*_6_, 298 K)

δ (ppm) 7.20 (t, ^3^ *J*_H,H_ = 7.6 Hz, 1H; *para*-C_6_ ***H***_3_), 6.91 (d, ^3^ *J*_H,H_ = 7.6 Hz, 2H; *meta*-C_6_ ***H***_3_), 6.82 (s, 4H; Dmp-Mes-*meta*-Ar-***H***), 2.15 (s, 12H; Dmp-Mes-*ortho*-C ***H***_3_), 2.14 (s, 6H; Dmp-Mes-*para*-C ***H***_3_).

##### ^11^B NMR (128.38 MHz, Benzene-*d*_6_, 298 K)

δ (ppm) 58.6 (s, br).

#### [MesH_2_]­[B­(C_6_F_5_)_4_]

##### ^1^H NMR (400.13 MHz, DCM-*d*_2_, 298 K)

δ (ppm) 7.44 (s, br, 2H; C ***H***), 4.44 (s, br, 2H; C ***H***_2_), 2.68 (s, br, 9H; C ***H***_3_).

### Generation of [**9**]^+^ from the Reaction of [**2**]­[B­(C_6_F_5_)_4_] and *tert*-Butyl Chloride

A solution of [**2**]­[B­(C_6_F_5_)_4_] (35.5 mg, 0.0316 mmol) in *o*-DCB-*d*_4_ (0.5 mL) was cooled to −15 °C before the addition of a precooled solution of *tert*-butyl chloride (3.44 μL, 0.0316 mmol) in *o*-DCB-*d*_4_ (0.5 mL) in a J. Young’s tube. The resulting dark-red solution was analyzed with NMR spectroscopy immediately. The ^11^B NMR signal corresponding to the chloroborinium cation moiety of [**9**]^+^ was not observed.

#### ^1^H NMR (400.13 MHz, *o*-DCB-*d*_4_, 298 K)

δ (ppm) 7.49 (t, 1H), 7.09 (d, 2H), 6.75 (s), 6.59 (s, br, 4H), 2.20 (s), 2.06 (s), 2.05 (s).

### Synthesis of **10** from the Reaction of [**2**]­[B­(C_6_F_5_)_4_] and [^*n*^Bu_4_N]­[PF_6_]

A solution of [**2**]­[B­(C_6_F_5_)_4_] (78.0 mg, 0.0695 mmol) in *o*-DFB (1 mL) was cooled to −35 °C before the dropwise addition of a precooled solution of [^*n*^Bu_4_N]­[PF_6_] (27.0 mg, 0.0697 mmol) in *o*-DFB (1 mL). The reaction mixture was stirred for 30 min, allowed to warm to room temperature slowly, and then placed *in vacuo* to remove all volatiles. The residue was extracted with *n*-hexane. The collected *n*-hexane solution was then concentrated to afford Dmp­(fluoro)­(mesityl)­borane (**10**) as a light-yellow solid in 87% yield (27.8 mg, 0.0611 mmol). Single crystals of **10** suitable for SC-XRD analysis were obtained by the slow evaporation of an *n*-hexane solution of **10** at room temperature under argon.

#### ^1^H NMR (500.17 MHz, Benzene-*d*_6_, 298 K)

δ (ppm) 7.25 (t, ^3^ *J*_H,H_ = 7.6 Hz, 1H; *para*-C_6_ ***H***_3_), 6.99 (d, ^3^ *J*_H,H_ = 7.6 Hz, 2H; *meta*-C_6_ ***H***_3_), 6.72 (s, 4H; Dmp-Mes-*meta*-C_6_ ***H***_2_), 6.39 (s, 2H, B-Mes-*meta*-C_6_ ***H***_2_), 2.15 (s, 18H; Dmp-Mes-C ***H***_3_), 2.07 (s, 6H; B-Mes-*ortho*-C ***H***_3_), 2.00 (s, 3H; B-Mes-*para*-C ***H***_3_).

#### ^11^B NMR (160.47 MHz, Benzene-*d*_6_, 298 K)

δ (ppm) 52.7 (s, br).

#### ^13^C­{^1^H} NMR (125.77 MHz, Benzene-*d*_6_, 298 K)

δ (ppm) 147.35 (Dmp-*ortho-* **C**_6_H_3_), 147.33 (Dmp-*ortho-* **C**_6_H_3_), 141.71 (B-Mes-*ortho-* **C**_6_H_2_), 141.69 (B-Mes-*ortho-* **C**_6_H_2_), 139.58 (Dmp-Mes-*ipso-* **C**_6_H_2_), 139.00 (B-Mes-*para-* **C**_6_H_2_), 138.82 (br, Dmp-*ipso-* **C**_6_H_3_), 136.37 (Dmp-Mes-*para-* **C**_6_H_2_), 136.17 (Dmp-Mes-*ortho-* **C**_6_H_2_), 134.29 (br, B-Mes-*ipso-* **C**_6_H_2_), 130.70 (Dmp-*para-* **C**_6_H_3_), 129.15 (Dmp-*meta-* **C**_6_H_3_), 128.33 (Dmp-Mes-*meta-* **C**_6_H_2_), 127.74 (B-Mes-*meta-* **C**_6_H_2_), 23.36 (B-Mes-*ortho-* **C**H_3_), 21.38 (Dmp-Mes-*ortho-* **C**H_3_), 21.18 (B-Mes-*para-* **C**H_3_), 21.06 (Dmp-Mes-*para-* **C**H_3_).

#### ^19^F­{^1^H} NMR (470.63 MHz, Benzene-*d*_6_, 298 K)

δ (ppm) −9.84 (s, br).

## Computational Sections

All quantum chemical calculations were performed with ORCA 6.1.0 according to the following procedure.^18^ The geometries were optimized using the r^2^SCAN0-D4-gCP/def2-TZVP level.^19^ Harmonic frequency calculations at the same level of theory were carried out to verify that the optimized structures correspond to either local minima or transition states. The transition state and intermediate searches were carried out with the climbing image nudged elastic band method,^30^ and the former was confirmed to be the transition state connecting both the educt and the product by intrinsic reaction coordinate (IRC) analysis.^20^ No additional intermediates (other than rotamers) were found in 50 IRC steps in both directions. The quasi rigid-rotor harmonic oscillator model (QRRHO) with Grimme’s entropy interpolation method was applied for low frequencies (<100 cm^–1^) to calculate the zero-point energies (ZPE) and thermal corrections (*G*_corr_ = *G* – EE for the Gibbs free energy correction, and *H*_corr_ = *H* – EE for the enthalpy correction) at 1.00 atm and 298.15 K using the ideal gas relation without a frequency scaling factor.^31^ Rotational symmetry factors are manually assigned according to the point groups of the optimized geometries. Higher accuracy single-point (SP) electronic energies were obtained on the optimized geometry using the DLPNO-CCSD­(T)/cc-pVTZ method with a TightPNO threshold.^21,22^ Solvation free energies in *o*-DCB were obtained from the COSMO-RS open source implementation with the default BP86/def2-TZVPD level.^23^ All calculations were performed with a DefGrid3 integration grid, and both the SCF and geometry optimization convergence thresholds were tightened (VeryTightSCF and TightOpt) to ensure reliable prediction. No orbitals were frozen during the post-SCF calculations. The total free energies of each compound were calculated as the sum of the single-point, *G*_corr_, and solvation energies. For the prediction of hyperfine coupling constants of **4** ^•^, see SI for further details.^32–34^

## Supplementary Material





## Notes

Synthesis, characterization, computational details, and NMR spectra (PDF)
Optimized geometries (XYZ)
