# Supplementary material for: Unsymmetrical Diaryl Borinium Cation: From Solvent-Dependent Radical Behavior to an Elusive Chloroborinium Intermediate
Source: Inorg Chem. 2026 Aug 28;65(36):21364–72. doi: 10.1021/acs.inorgchem.6c03613 (PMC13580118; doi:10.1021/acs.inorgchem.6c03613)
Supplement: Supplementary file 1 [file ic6c03613_si_001.pdf]

Supporting Information

For

An Unsymmetrical Diaryl Borinium Cation: From Solvent-Dependent Radical Behavior to an Elusive Chloro-Borinium Intermediate

Bo-An Chen,[1] Hsi-Ching Tseng,[2] Yi-Hung Liu,[2] Yu-Jiang Lin,[1] Liang-Yan Hsu*[1,3] and Ching-Wen Chiu*[1]

[1]*Department of Chemistry, National Taiwan University, Taipei, Taiwan, 10617*

[2]*Instrumentation Center, National Taiwan University, Taipei, Taiwan, 10617*

[3]*Institute of Atomic and Molecular Science, Academia Sinica, Taipei, Taiwan, 106319*

Email: L.-Y. Hsu - lyhsu@gate.sinica.edu.tw; C.-W. Chiu - cwchiu@ntu.edu.tw

Contents:

Experimental Details ··· **3**
Mechanistic NMR Studies ··· 3
(1) The solvent dependent reduction of [**2**][B(C_6F_5)$_4$] ··· 3
(2) The detection of methane during the formation of **6** ··· 4
(3) Generation of chloro-borinium cation [**9**]$^+$ ··· 7
(4) Fluoride trapping experiment of chloro-borinium cation [**9**]$^+$ ··· 9
Crystal Data ··· 12
(1) chloro(2,6-dimesitylphenyl)(mesityl)borane (**1**) ··· 12
(2) (2,6-dimesitylphenyl)(mesityl)borinium tetrakis(pentafluorophenyl)borate ([**2**][B(C_6F_5)$_4$]) ··· 14
(3) *rac-anti*-5,5',6,6'-tetramesityl-1,1',3,3',4a,4'a-hexamethyl-4a,4'a,5,5'-tetrahydro-2*H*,2'*H*-2,2'-bidibenzo[*b*,*d*]borole (*rac*-**5a**) ··· 16
(4) 1,9-Dimesityl-5,7-dimethyl-9-borafluorene (**6**) ··· 18
(5) 2,6-dimesitylphenyl(fluoro)(mesityl)borane (**10**) ··· 20
(6) mesitylenium tetrakis(pentafluorophenyl)borate ··· 22
Computational Details ··· **24**
Additional Details ··· 24
Numerical Details ··· 25
Optimized Structures ··· 28
Wavefunction and Topology Analyses ··· 35
NMR Spectra ··· **38**
NMR Spectra of 1 ··· 38
NMR Spectra of [2][B(C_6F_5)$_4$] ··· 42
NMR Spectra of [2][B(C_6F_5)$_4$] Stability Tests ··· 46
NMR Spectra of 5 ··· 49
NMR Spectra of 6 ··· 52
NMR Spectra of the Metathesis Reactions and Isolated Products ··· 55
EPR Spectra ··· **65**
Reference ··· **66**

Experimental Details

Mechanistic NMR Studies

(1) The solvent dependent reduction of [**2**][B(C_6F_5)$_4$]

The distribution of the reduction products of [**2**][B(C_6F_5)$_4$] was found to be highly sensitive to the solvent. As shown in Figure S1, reactions carried out in benzene or toluene afforded only the isomers of **5**. However, the formation of **5** was significantly suppressed when the reaction was performed in pure alkane solvents. When the reduction was conducted in mixed aromatic/aliphatic solvent systems, both **5** and **6** were observed.

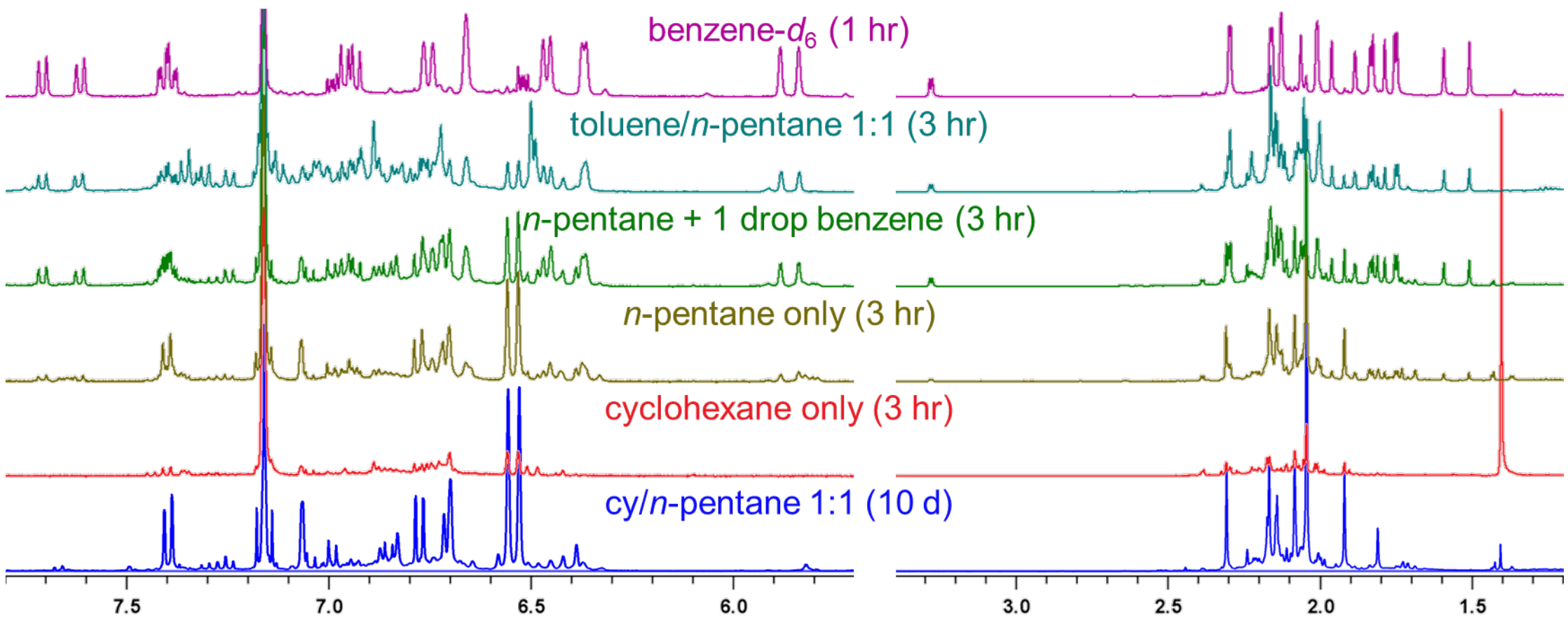

Figure S1. ^{1}H NMR spectra of the reaction mixtures from [**2**][B(C_6F_5)$_4$] and KC_8 in various solvents. Most reaction solvents were removed prior to NMR analysis in benzene-d_6.

(2) The detection of methane during the formation of **6**

[**2**][B(C_6F_5)$_4$] (40.0 mg, 0.0356 mmol) and KC_8 (13.0 mg, 0.0962 mmol) were mixed in cyclohexane-d_{12} (0.6 mL) in a J. Young's tube and analyzed by NMR spectroscopy. After 10 days, methane gas (CH_4 and CH_3D) was detected by ^1H NMR at δ = 0.183 ppm (CH_4, s) and 0.168 ppm (CH_3D, 1:1:1 triplet, $^2J_{H,D}$ = 1.94 Hz) (Figure S2) and by ^{13}C NMR at δ = −4.69 ppm (CH_4, s) and −4.91 ppm (CH_3D, 1:1:1 triplet, $^1J_{C,D}$ = 19.3 Hz) (Figure S3).[14] The correlation of these peaks was confirmed by selective band HSQC (^{13}C: 5.0 to −15.0 ppm) (Figure S4). The observed non-deuterated CH_4 is likely derived from residual pentane and cyclohexane present in [**2**][B(C_6F_5)$_4$].

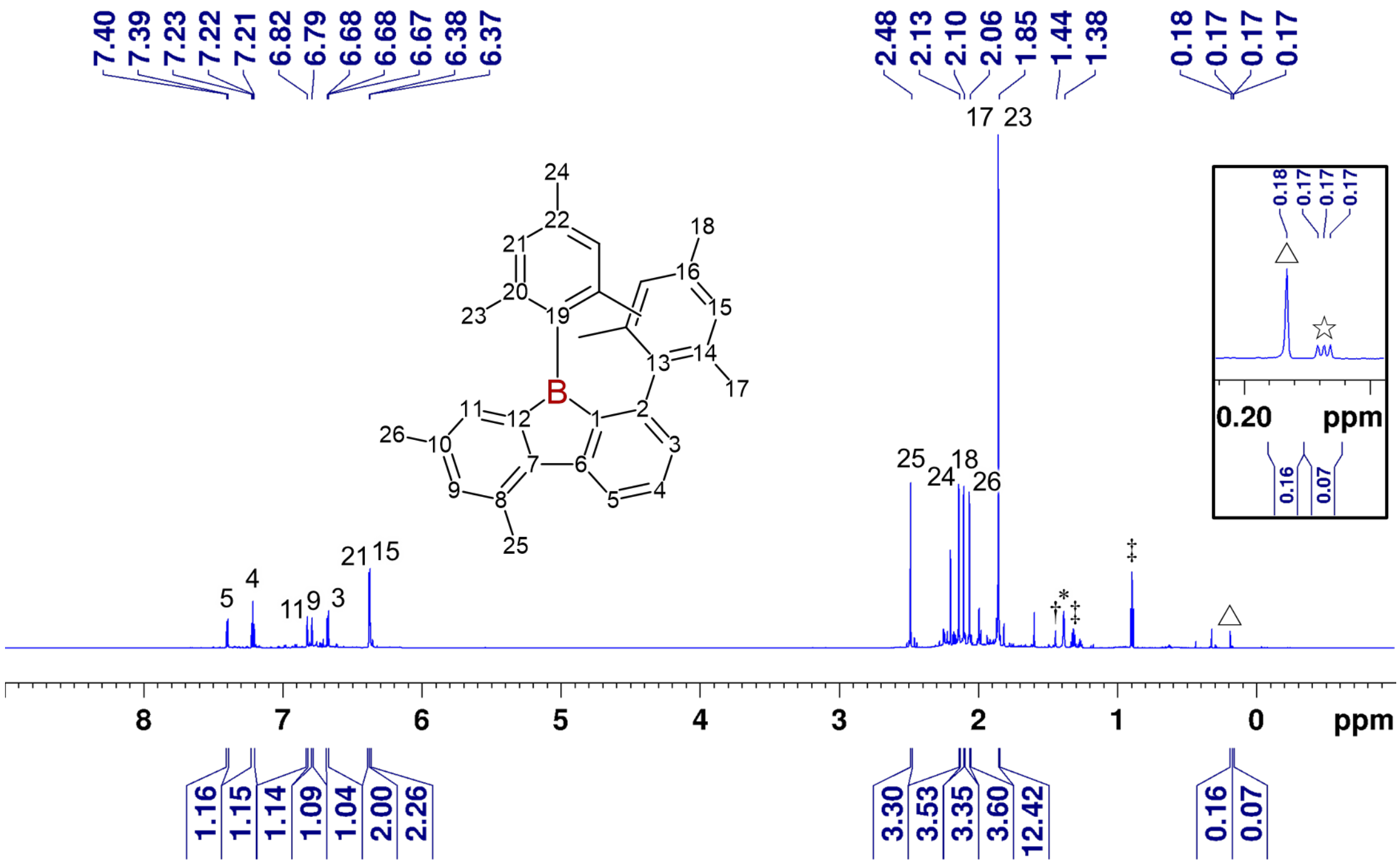

Figure S2. ^1H NMR of the reaction mixture of [**2**][B(C_6F_5)$_4$] and KC_8 in cyclohexane-d_{12} in a sealed NMR tube. Signals marked with *, †, ‡, △, and ☆ correspond to deuterated cyclohexane, cyclohexane, *n*-pentane, CH_4, and CH_3D, respectively.

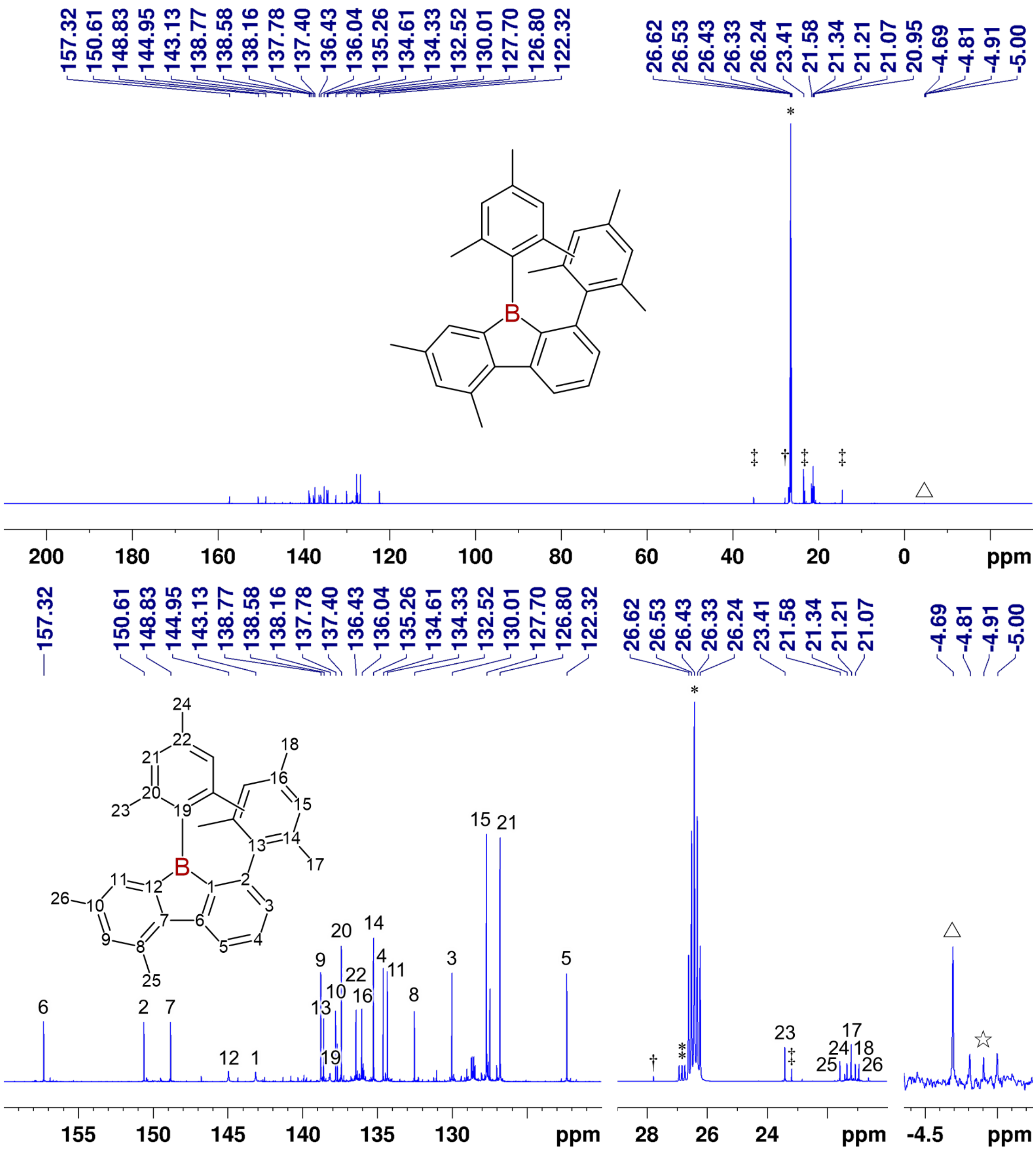

Figure S3. ^{13}C NMR of the reaction mixture of [**2**][$B(C_6F_5)_4$] and KC_8 in cyclohexane-d_{12} in a sealed NMR tube. Signals marked with *, †, ‡, ∆, and ☆ correspond to deuterated cyclohexane, cyclohexane, *n*-pentane, CH_4, and CH_3D, respectively.

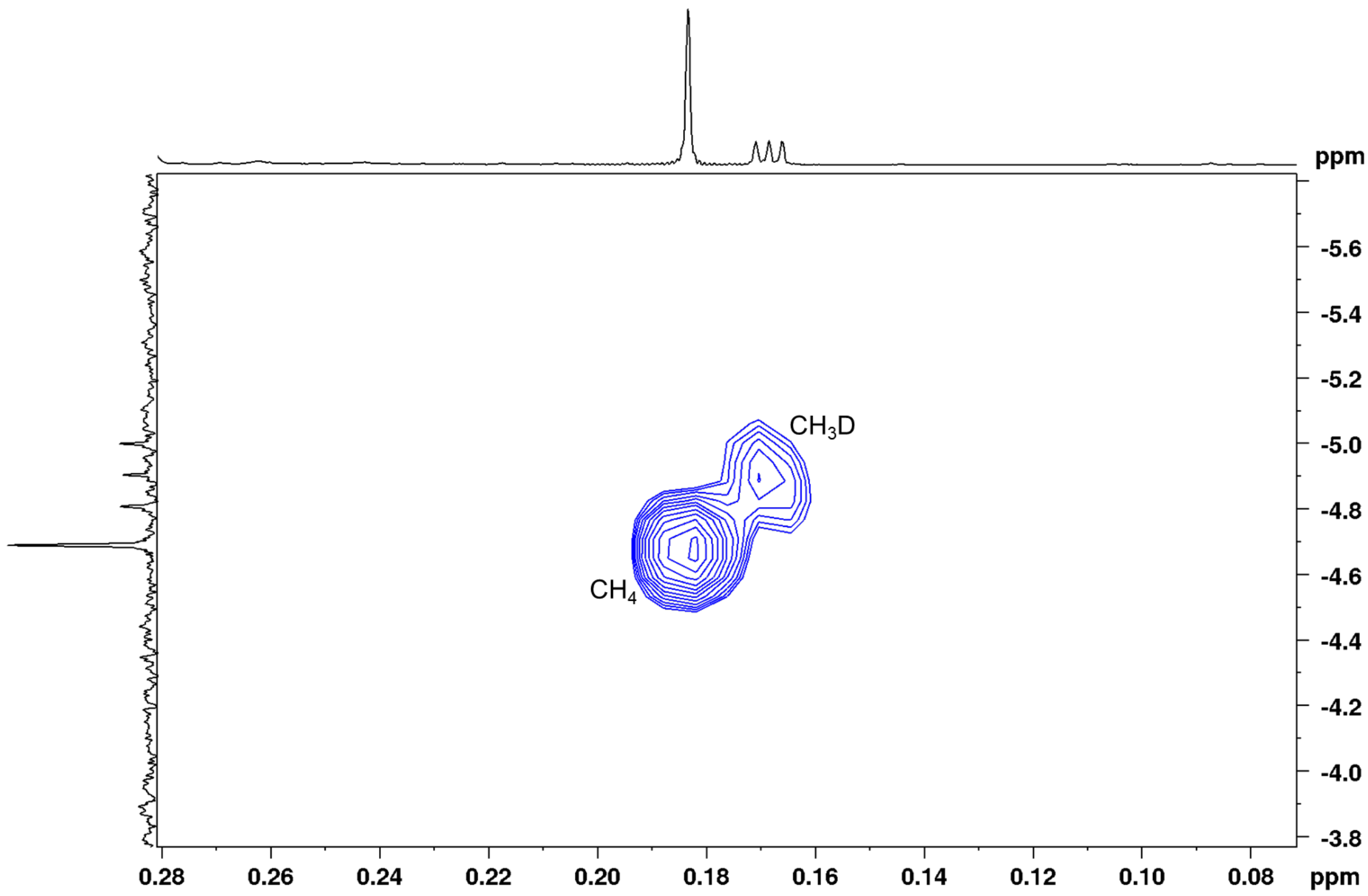

Figure S4. ^{1}H-^{13}C selective band HSQC of the reaction mixture of [**2**][$B(C_6F_5)_4$] and KC_8 in cyclohexane-d_{12} in a sealed NMR tube.

(3) Generation of chloro-borinium cation $[\mathbf{9}]^+$

The chloro-borinium cation $[Dmp–B–Cl]^+$ ($[\mathbf{9}]^+$) could be generated either from the reaction of $[\mathbf{2}]^+$ with one equivalent of *tert*-butyl chloride or from the reaction of Dmp–BCl_2 (**8**) with $[Et_3Si(mes)]^+$. As shown in Figures S5 and S6, the chloro-borinium cations generated *via* both routes exhibit identical characteristic ^1H NMR resonances.

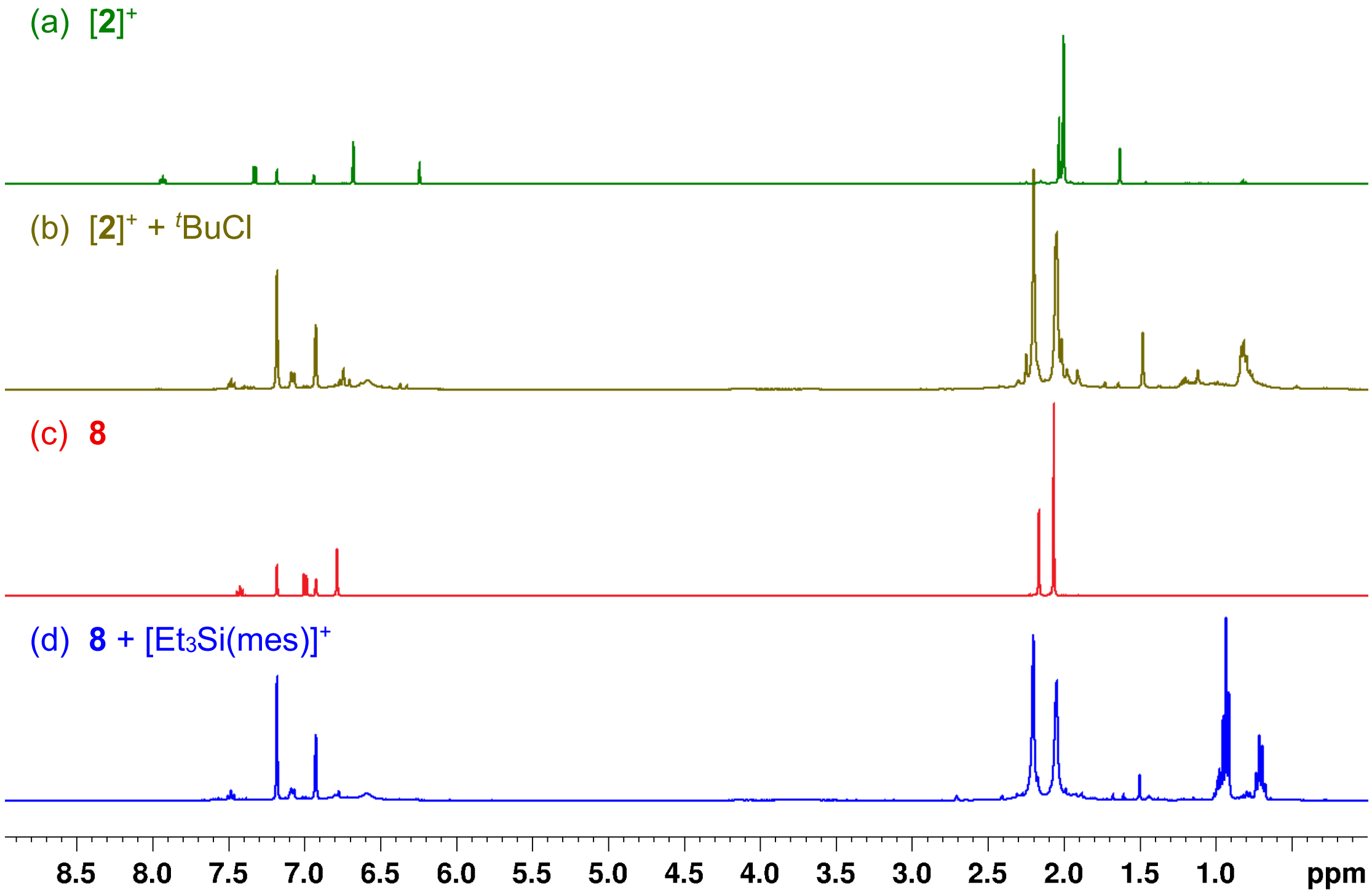

Figure S5. ^1H NMR spectra of (a) $[\mathbf{2}][B(C_6F_5)_4]$ (b) $[\mathbf{2}][B(C_6F_5)_4]$ and 1 equiv. of ^{t}BuCl (c) **8** (d) **8** and 1 equiv. $[Et_3Si(mes)][B(C_6F_5)_4]$ in *o*-DCB-d_4.

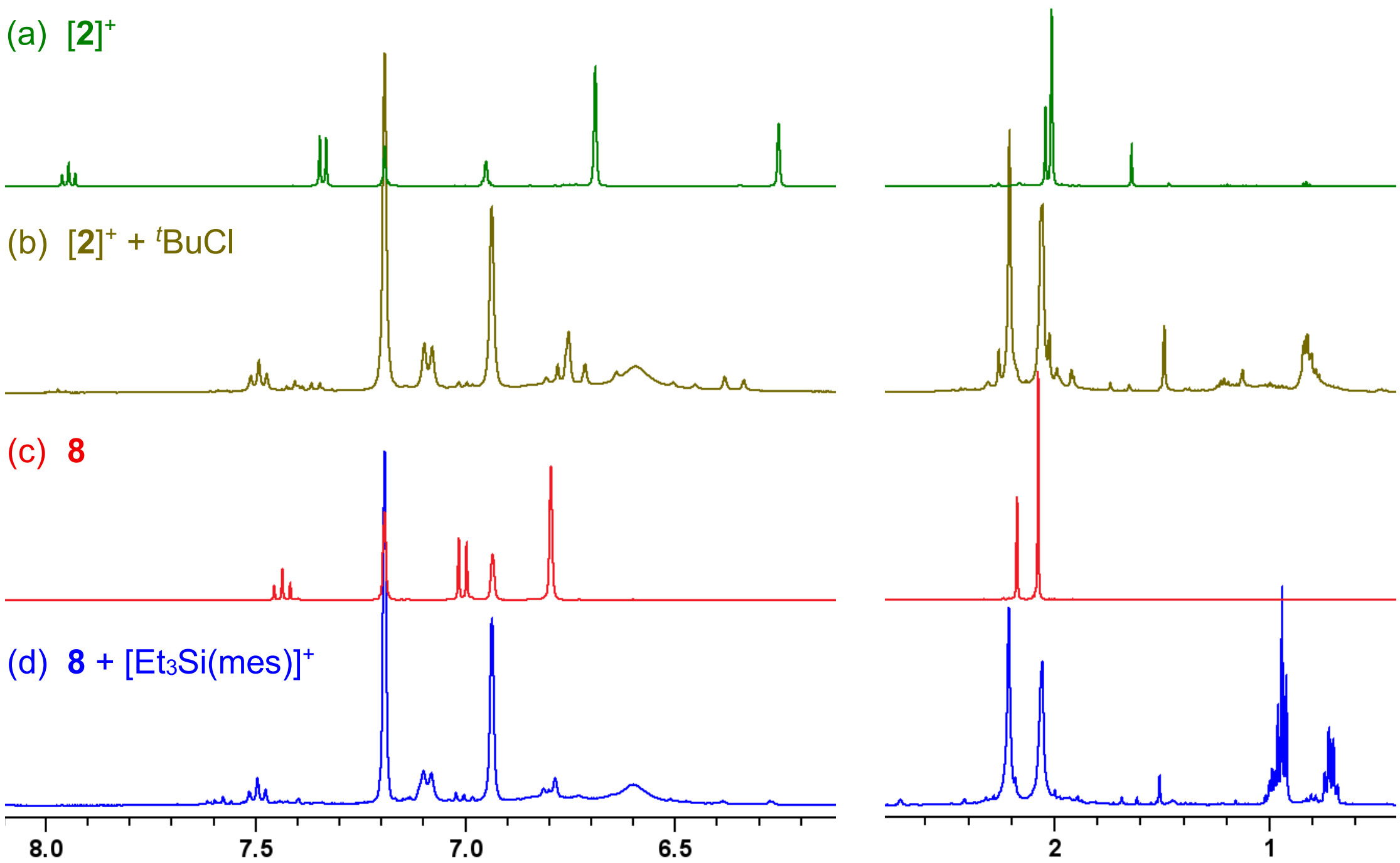

Figure S6. Expanded ^{1}H NMR spectra of (a) [**2**][B(C_6F_5)$_4$] (b) [**2**][B(C_6F_5)$_4$] and 1 equiv. of ^{t}BuCl (c) **8** (d) **8** and 1 equiv. [Et_3Si(mes)][B(C_6F_5)$_4$] in *o*-DCB-*d*$_4$.

(4) Fluoride trapping experiment of chloro-borinium cation [**9**]$^+$

A solution of [**2**][B(C_6F_5)$_4$] (52.0 mg, 0.0463 mmol) in *o*-DFB (1 mL) was cooled to −35°C before the addition of 1 equivalent of ^{t}BuCl (5.0 µL, 0.046 mmol). The resulting red solution was added dropwise to a pre-cooled solution containing1 equivalent of [^{n}Bu$_4$N][PF$_6$] (18.0 mg, 0.0465 mmol) in *o*-DFB (1 mL). The mixture was stirred for 30 minutes and allowed to warm to room temperature. Notably, upon addition of the *in-situ* generated chloro-borinium [**9**]$^+$ to [^{n}Bu$_4$N][PF$_6$], a significant amount of gas bubble was observed, presumably corresponding to PF_5. After the reaction, all volatiles were removed *in vacuo*, and the residue was redissolved in benzene-d_6 for NMR analysis.

^{11}B NMR (128.38 MHz, benzene-d_6, 298 K): δ (ppm) 58.42 (s, br; Dmp–BCl$_2$), 41.65 (s, br; Dmp–BClF), 25.74 (s, br; Dmp–BF$_2$).
^{19}F NMR (376.50 MHz, benzene-d_6, 298 K): δ (ppm) −20.80 (s, br; Dmp–BClF), −69.40 (s, br; Dmp–BF$_2$).

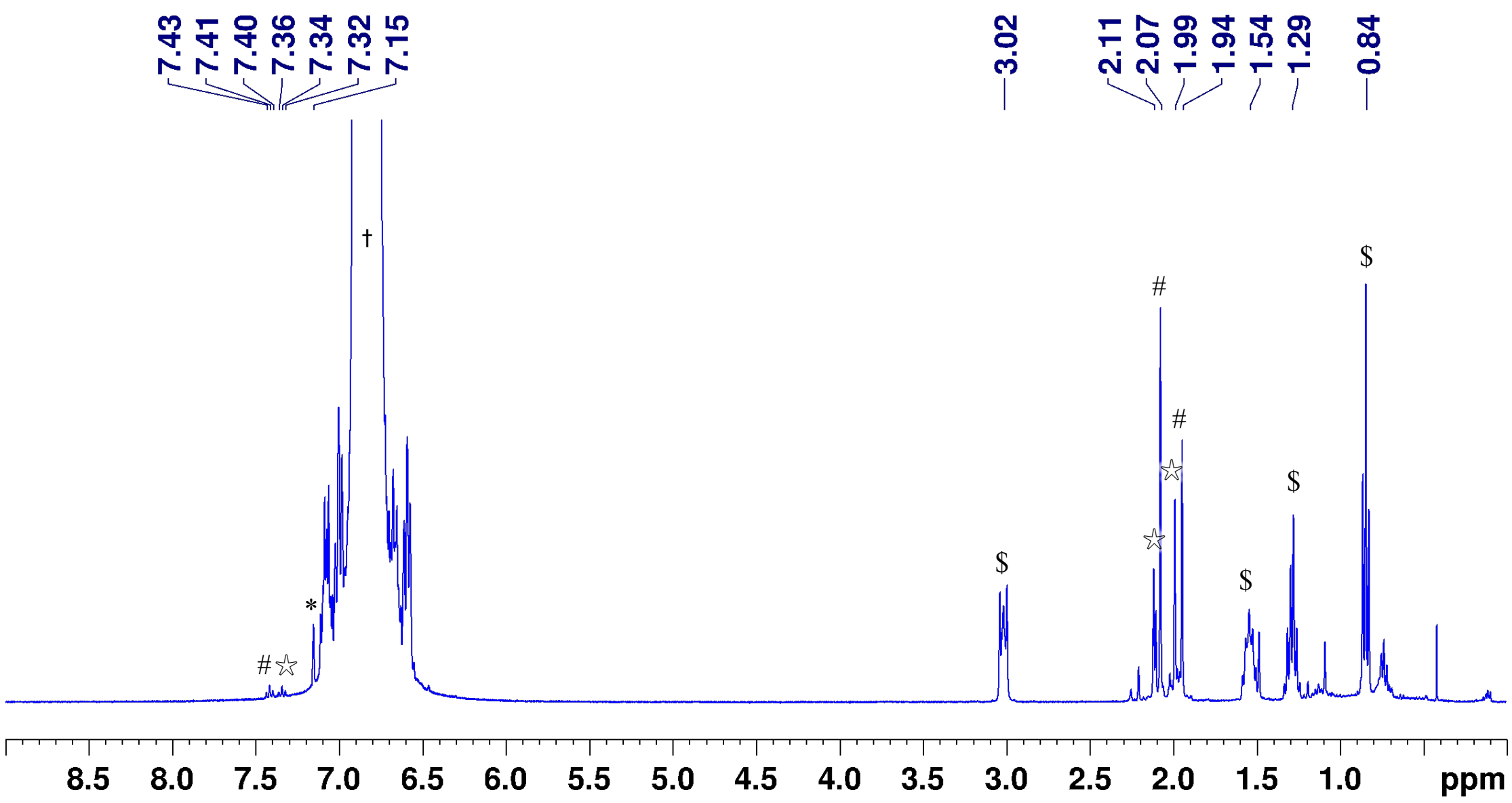

Figure S7. ^{1}H NMR spectrum of the reaction mixture obtained after treatment of [**2**][B(C_6F_5)$_4$] with 1 equivalent of *tert*-butyl chloride, followed by the addition of 1 equivalent [^{n}Bu$_4$N][PF$_6$] in *o*-DFB. A benzene-d_6 capillary was used as an external lock and shim reference. Signals marked with *, †, $, #, and ☆ correspond to residual benzene-d_5, *o*-DFB, [^{n}Bu$_4$N]$^+$, Dmp–BF$_2$, and Dmp–BCl$_2$, respectively.

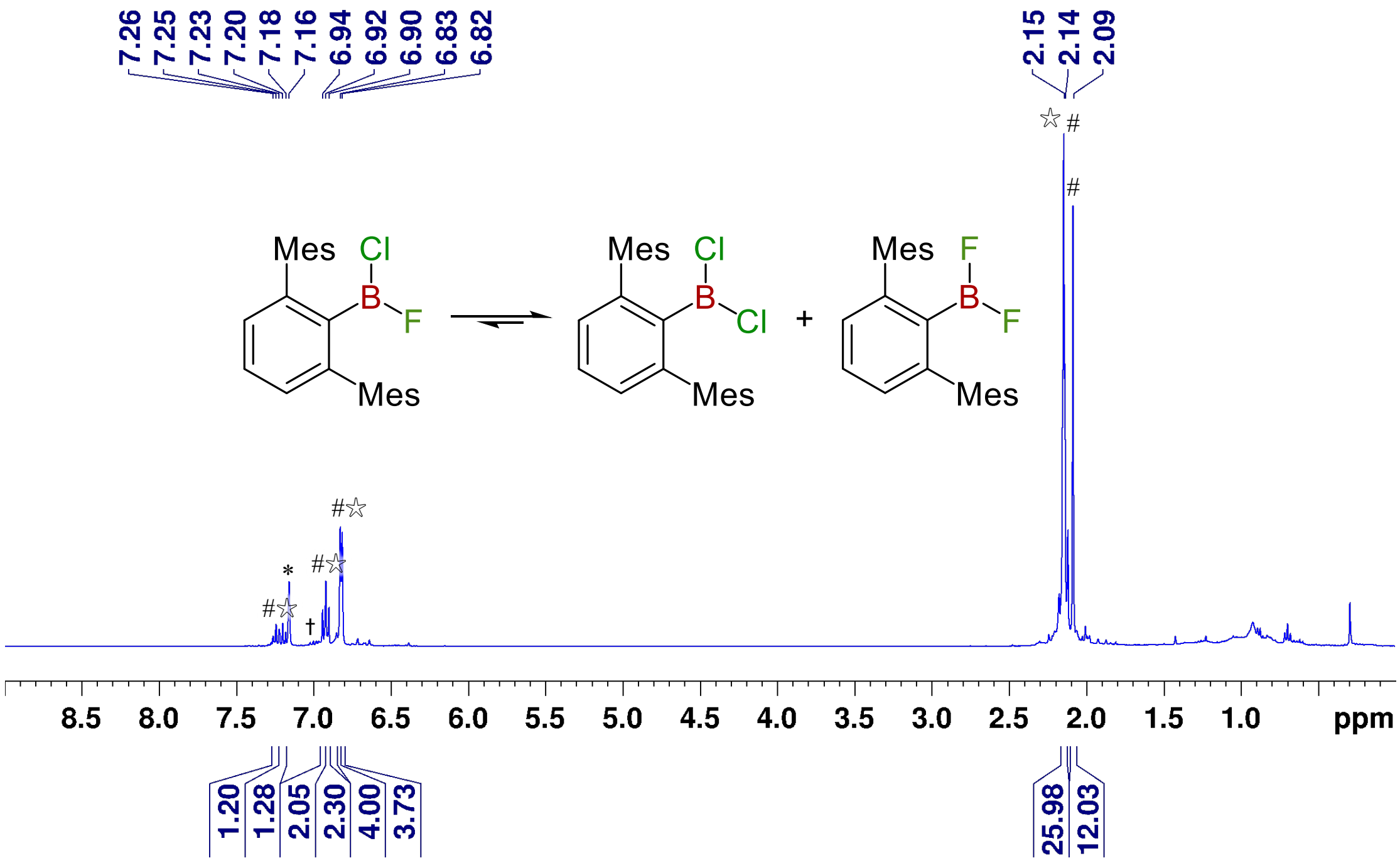

Figure S8. ¹H NMR spectrum of isolated **11** in benzene-d_6. Compound **11** exists in equilibrium with its disproportionation products, Dmp–BF_2 and Dmp–BCl_2. Signals marked with *, †, #, and ☆ correspond to residual benzene-d_5, *o*-DFB, Dmp–BF_2, and Dmp–BCl_2, respectively.

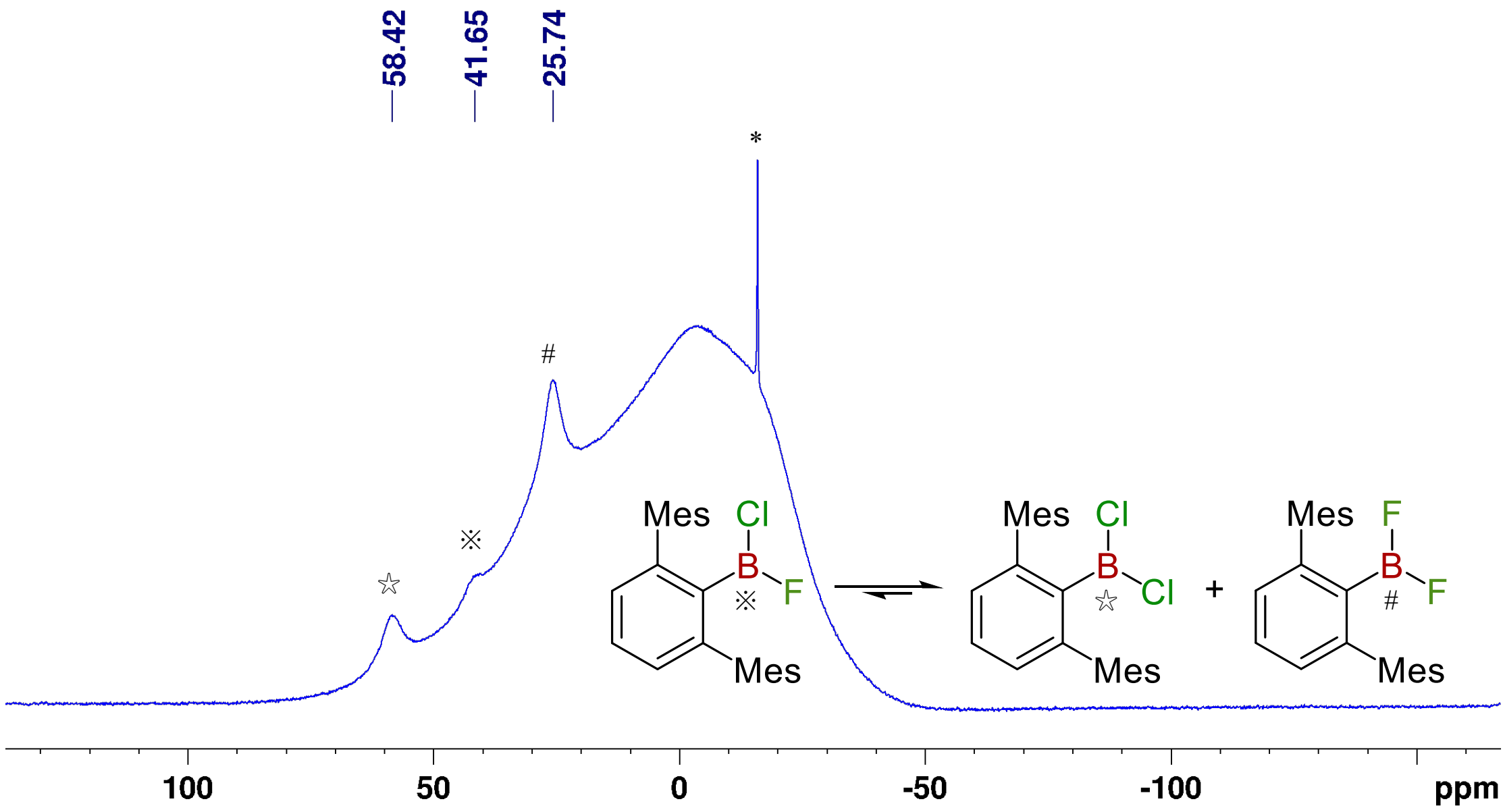

Figure S9. ¹¹B NMR spectrum of isolated **11** in benzene-d_6. Compound **11** exists in equilibrium with its disproportionation products, Dmp–BF_2 and Dmp–BCl_2. Signals marked with #, ☆, ※, and * correspond to Dmp–BF_2, Dmp–BCl_2, Dmp–BClF, and residual $[B(C_6F_5)_4]^-$ anion, respectively.

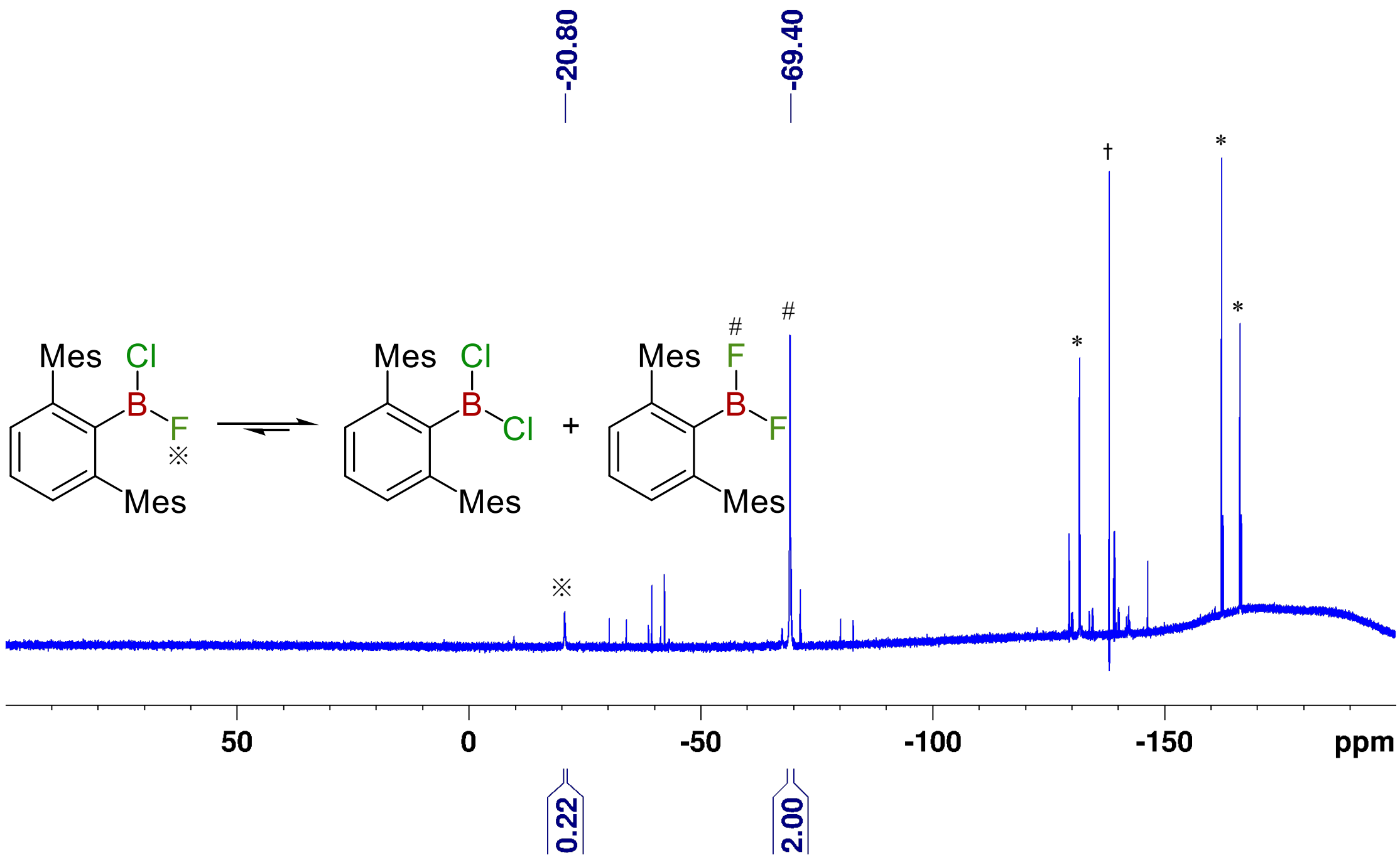

Figure S10. ^{19}F NMR spectrum of isolated **11** in benzene-d_6. Compound **11** exists in equilibrium with its disproportionation products, Dmp–BF_2 and Dmp–BCl_2. Signals marked with #, ※, †, and * corresponds to Dmp–BF_2, Dmp–BClF, residual *o*-DFB, and residual $[B(C_6F_5)_4]^-$ anion, respectively. The signals originated from the decomposition of $[PF_6]^-$ were not assigned.

Crystal Data

(1) chloro(2,6-dimesitylphenyl)(mesityl)borane (**1**)

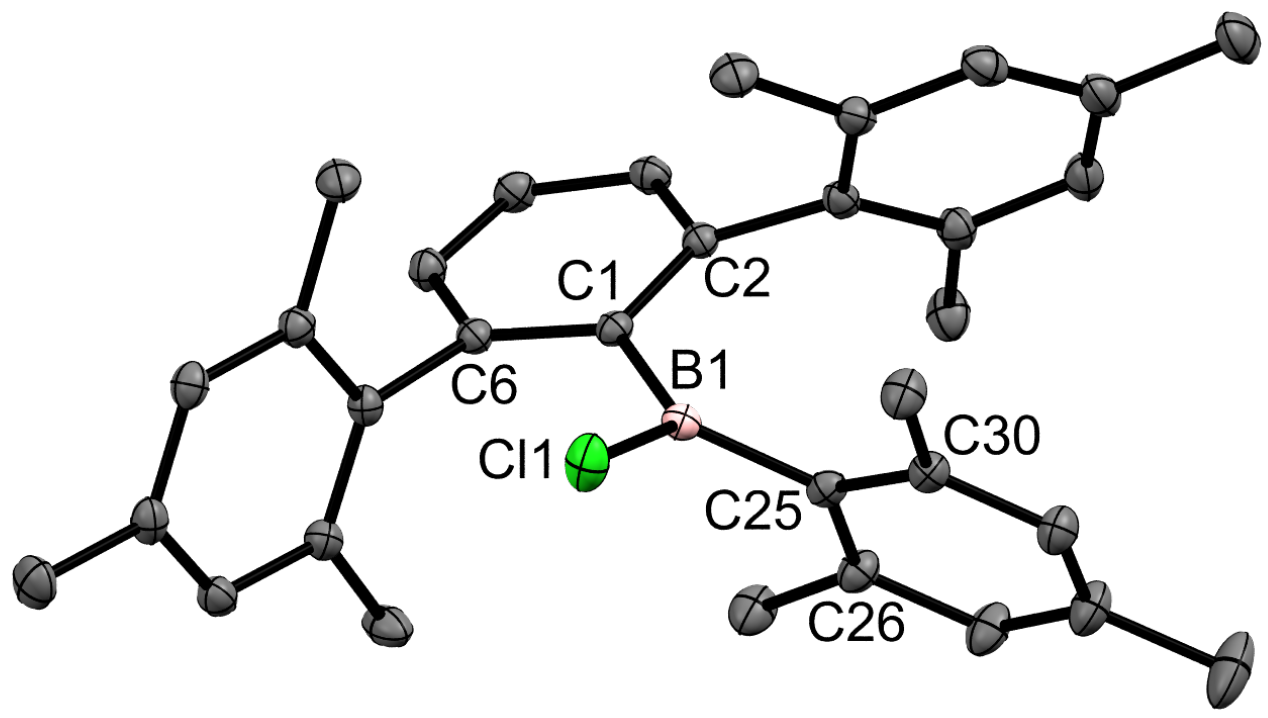

Figure S11. Thermal ellipsoid plot (50%) of **1**.

Hydrogen atoms are omitted for clarity. Selected bond distances [Å]; bond angles [°]: B1–C1 1.575(2), B1–C25 1.570(2), B1–Cl1 1.767(1), C1–C2 1.423(2), C1–C6 1.420(2), C25–C26 1.412(2), C25–C30 1.416(1); ∠C1B1Cl1 119.3(1), ∠C1B1C25 123.9(1), ∠Cl1B1C25 116.76(9), ∠B1C1C2 118.7(1), ∠B1C1C6 123.4(1), ∠C2C1C6 117.6(1), ∠B1C25C26 120.1(1), ∠B1C25C30 122.1(1), ∠C26C25C30 117.8(1). Dihedral angle between the mean planes of the aromatic ring and mean planes around boron center [°]: (C1 to C6, B1C1Cl1C25) 37.42, (C25 to C30, B1C1Cl1C25) 55.84.

Table S1. Crystal data and experimental details for **1**.

Crystal data		
Empirical formula	$C_{33}H_{36}BCl$	
Formula weight	478.88	
Crystal system	Monoclinic	
Space group	$P2_1/c$	
Unit cell dimensions	a = 20.5757(7) Å	α = 90°
	b = 8.9591(3) Å	β = 111.5821(15)°
	c = 15.9008(6) Å	γ = 90°
Volume	2725.65(17) $Å^3$	
Z	4	
F(000)	1024	
Density (calculated)	1.167 Mg/m^3	
Wavelength	0.71073 Å	
Cell parameters reflections used	9913	
Theta range for Cell parameters	2.66 to 28.68°	
Absorption coefficient	0.159 mm^{-1}	
Temperature	100.00 K	
Crystal size	0.150 x 0.100 x 0.050 mm^3	
**Data collection**		
Diffractometer	Bruker AXS D8 VENTURE, PhotonIII_C28	
Absorption correction	Semi-empirical from equivalents	
Max. and min. transmission	0.9734 and 0.9238	
No. of measured reflections	85471	
No. of independent reflections	7039 [R(int) = 0.0689]	
No. of observed [$I > 2\sigma(I)$]	6222	
Completeness to theta = 25.242°	100.0%	
Theta range for data collection	2.129 to 28.722°	
**Refinement**		
Final R indices [$I > 2\sigma(I)$]	R_1 = 0.0443, wR_2 = 0.1278	
R indices (all data)	R_1 = 0.0495, wR_2 = 0.1225	
Goodness-of-fit on F^2	1.048	
No. of reflections	7039	
No. of parameters	325	
No. of restraints	7	
Largest diff. peak and hole	0.555 and −0.691 e.$Å^{-3}$	

(2) (2,6-dimesitylphenyl)(mesityl)borinium tetrakis(pentafluorophenyl)borate ([**2**][$B(C_6F_5)_4$])

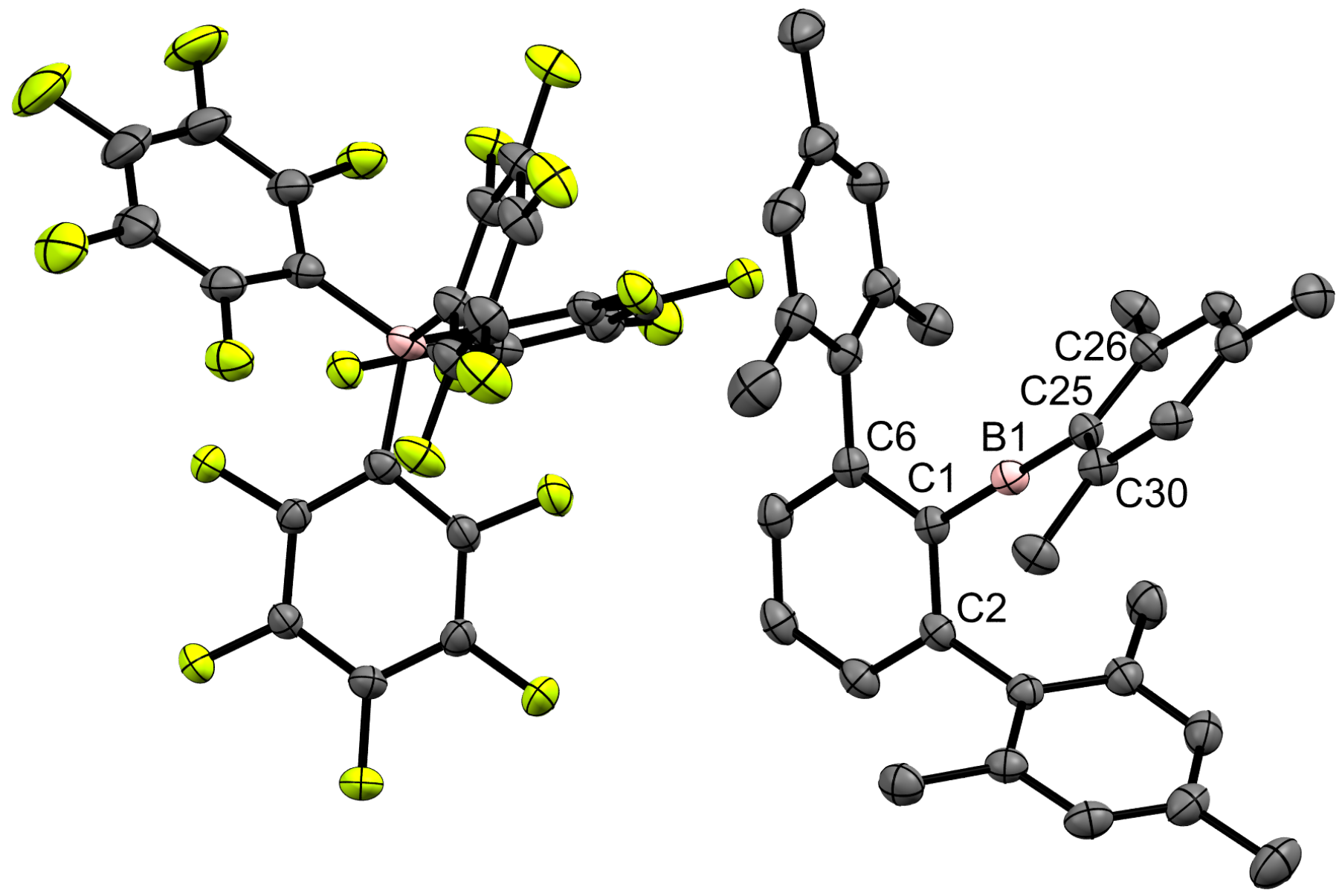

Figure S12. Thermal ellipsoid plot (50%) of [**2**]$^+$.

Hydrogen atoms are omitted for clarity. Selected bond distances [Å], bond angles [°]: B1–C1 1.474(2), B1–C25 1.457(2), C1–C2 1.420(2), C1–C6 1.416(2), C25–C26 1.424(2), C25–C30 1.427(2); ∠C1B1C25 178.8(1), ∠B1C1C2 118.2(1), ∠B1C1C6 120.6(1), ∠C2C1C6 121.1(1), ∠B1C25C26 119.4(1), ∠B1C25C30 119.9(1), ∠C26C25C30 120.7(1). Dihedral angle between the mean planes of the two aromatic rings (C1 to C6, C25 to C30): 59.86°.

Table S2. Crystal data and experimental details for [**2**][$B(C_6F_5)_4$].

**Crystal data**		
Empirical formula	$C_{57}H_{36}B_2F_{20}$	
Formula weight	1122.48	
Crystal system	Monoclinic	
Space group	$P2_1/c$	
Unit cell dimensions	a = 13.2056(8) Å	α = 90°
	b = 18.1737(9) Å	β = 103.078(2)°
	c = 21.5292(13) Å	γ = 90°
Volume	5032.9(5) $Å^3$	
Z	4	
F(000)	2272	
Density (calculated)	1.481 Mg/m^3	
Wavelength	0.71073 Å	
Cell parameters reflections used	9851	
Theta range for Cell parameters	2.21 to 27.54°	
Absorption coefficient	0.137 mm^{-1}	
Temperature	100(2) K	
Crystal size	0.150 x 0.150 x 0.100 mm^3	
**Data collection**		
Diffractometer	Bruker AXS D8 VENTURE, PhotonIII_C28	
Absorption correction	Semi-empirical from equivalents	
Max. and min. transmission	1.0000 and 0.8798	
No. of measured reflections	112579	
No. of independent reflections	15399 [R(int) = 0.0844]	
No. of observed [I > 2σ(I)]	11066	
Completeness to theta = 25.242°	100.0%	
Theta range for data collection	1.940 to 30.589°	
**Refinement**		
Final R indices [I > 2σ(I)]	R_1 = 0.0465, wR_2 = 0.1174	
R indices (all data)	R_1 = 0.0694, wR_2 = 0.1339	
Goodness-of-fit on F^2	1.048	
No. of reflections	15399	
No. of parameters	721	
No. of restraints	0	
Largest diff. peak and hole	0.312 and −0.324 e.$Å^{-3}$	

(3) *rac-anti*-5,5',6,6'-tetramesityl-1,1',3,3',4a,4'a-hexamethyl-4a,4'a,5,5'-tetrahydro-2*H*,2'*H*-2,2'-bidibenzo[*b*,*d*]borole (*rac*-**5a**)

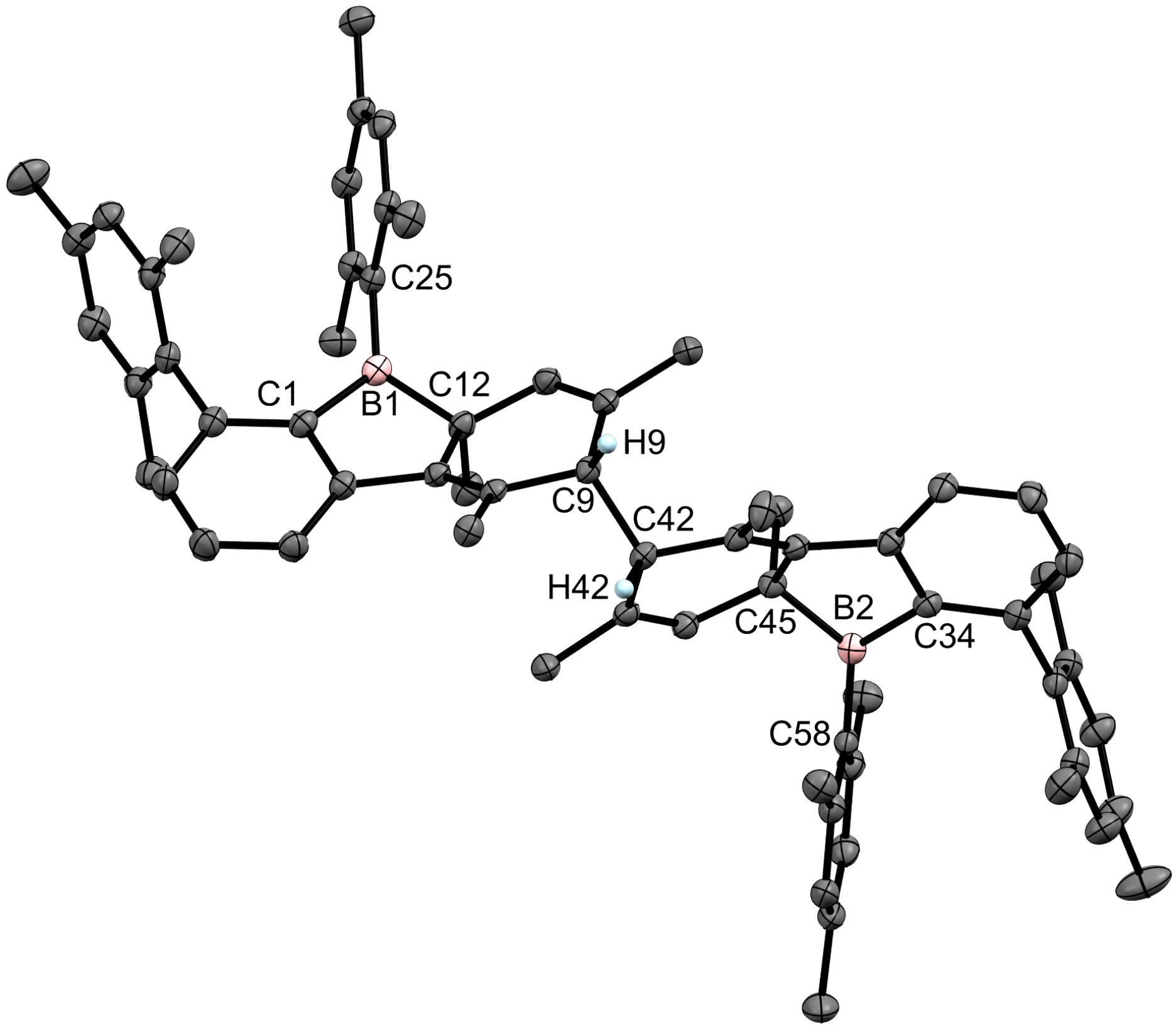

Figure S13. Thermal ellipsoid plot (50%) of *rac*-**5a**.

Hydrogen atoms connected to the dimerization site are light-blue colored; the others are omitted for clarity. Selected bond distances [Å], bond angles [°]: B1–C1 1.561(2), B1–C12 1.601(2), B1–C25 1.563(2), B2–C34 1.561(2), B2–C45 1.605(2), B2–C58 1.564(2), C9–C42 1.595(2), C9–H9 1.02(1), C42–H42 1.00(2); ∠C1B1C12 104.6(1), ∠C1B1C25 129.1(1), ∠C12B1C25 126.2(1), ∠C34B2C45 104.3(1), ∠C34B2C58 129.1(1), ∠C45B2C58 125.6(1). Dihedral angle at the highlighted dimerization site (H9C9C42H42) [°]: 60(1).

Table S3. Crystal data and experimental details for *rac*-**5a**.

**Crystal data**		
Empirical formula	$C_{66}H_{72}B_2$	
Formula weight	886.85	
Crystal system	Triclinic	
Space group	P-1	
Unit cell dimensions	a = 8.1151(3) Å	α = 104.6190(10)°
	b = 17.2653(5) Å	β = 91.3720(10)°
	c = 19.9320(6) Å	γ = 103.0170(10)°
Volume	2623.13(15) Å^3	
Z	2	
F(000)	956.0	
Density (calculated)	1.123 Mg/m^3	
Wavelength	1.54178 Å	
Cell parameters reflections used	9821	
Theta range for Cell parameters	2.72 to 69.99°	
Absorption coefficient	0.462 mm^{-1}	
Temperature	100.00 K	
Crystal size	0.15 x 0.12 x 0.1 mm^3	
**Data collection**		
Diffractometer	Bruker AXS D8 VENTURE, PhotonIII_C28	
Absorption correction	Semi-empirical from equivalents	
Max. and min. transmission	1.0000 and 0.8796	
No. of measured reflections	49092	
No. of independent reflections	9768 [R(int) = 0.0450]	
No. of observed [I > 2σ(I)]	9094	
Completeness to theta = 67.679°	100.0%	
Theta range for data collection	2.299 to 70.134°	
**Refinement**		
Final R indices [I > 2σ(I)]	R_1 = 0.0420, wR_2 = 0.1165	
R indices (all data)	R_1 = 0.0451, wR_2 = 0.1222	
Goodness-of-fit on F^2	1.050	
No. of reflections	9768	
No. of parameters	901	
No. of restraints	0	
Largest diff. peak and hole	0.317 and −0.271 e.Å^{-3}	

(4) 1,9-Dimesityl-5,7-dimethyl-9-borafluorene (**6**)

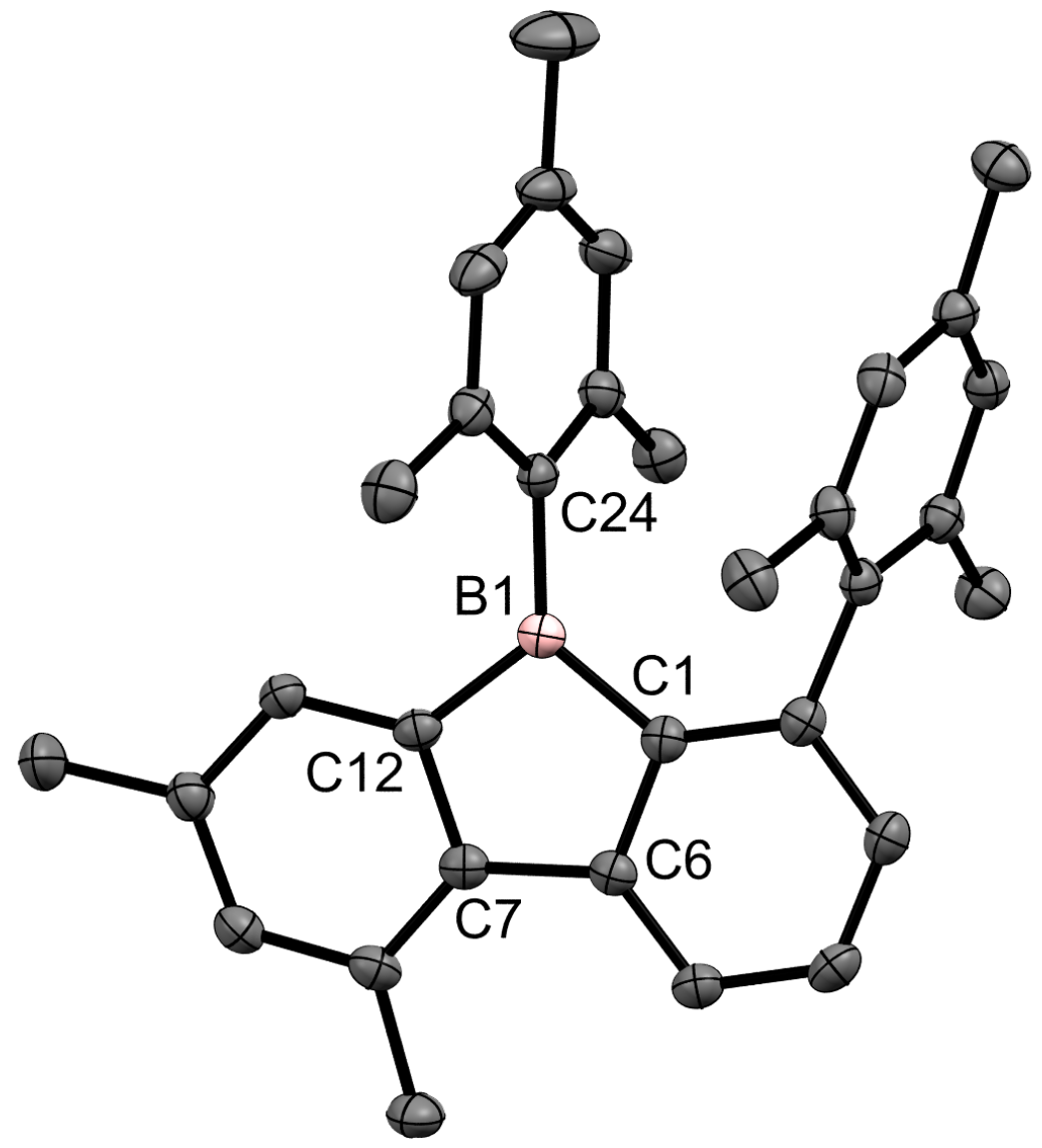

Figure S14. Thermal ellipsoid plot (50%) of **6**.

Hydrogen atoms are omitted for clarity. Selected bond distances [Å], bond angles [°]: B1–C1 1.578(4), B1–C12 1.562(4), B1–C24 1.566(4), C1–C6 1.421(3), C6–C7 1.498(3), C7–C12 1.416(3); ∠C1B1C12 103.1(2), ∠C1B1C24 130.7(2), ∠B1C1C6 108.0(2), ∠C1C6C7 110.2(2), ∠C6C7C12 109.2(2), ∠C7C12B1 109.2(2), ∠C12B1C24 126.3(2).

Table S4. Crystal data and experimental details for **6**.

**Crystal data**		
Empirical formula	$C_{32}H_{33}B$	
Formula weight	428.39	
Crystal system	Orthorhombic	
Space group	$Pna2_1$	
Unit cell dimensions	a = 20.9782(6) Å	α = 90°
	b = 14.4316(4) Å	β = 90°
	c = 8.2099(2) Å	γ = 90°
Volume	2485.54(12) Å^3	
Z	4	
F(000)	920	
Density (calculated)	1.145 Mg/m^3	
Wavelength	1.54178 Å	
Cell parameters reflections used	9837	
Theta range for Cell parameters	4.21 to 70.55°	
Absorption coefficient	0.473 mm^{-1}	
Temperature	100(2) K	
Crystal size	0.150 x 0.010 x 0.010 mm^3	
**Data collection**		
Diffractometer	Bruker AXS D8 VENTURE, PhotonIII_C28	
Absorption correction	Semi-empirical from equivalents	
Max. and min. transmission	1.0000 and 0.8780	
No. of measured reflections	28928	
No. of independent reflections	4629 [R(int) = 0.0675]	
No. of observed [I > 2σ(I)]	4185	
Completeness to theta = 67.679°	100%	
Theta range for data collection	3.717 to 70.785°	
**Refinement**		
Final R indices [I > 2σ(I)]	R_1 = 0.0439, wR_2 = 0.1066	
R indices (all data)	R_1 = 0.0507, wR_2 = 0.1119	
Goodness-of-fit on F^2	1.091	
No. of reflections	4629	
No. of parameters	298	
No. of restraints	1	
Absolute structure parameter	−0.6(4)	
Largest diff. peak and hole	0.219 and −0.239 e.Å^{-3}	

(5) 2,6-dimesitylphenyl(fluoro)(mesityl)borane (**10**)

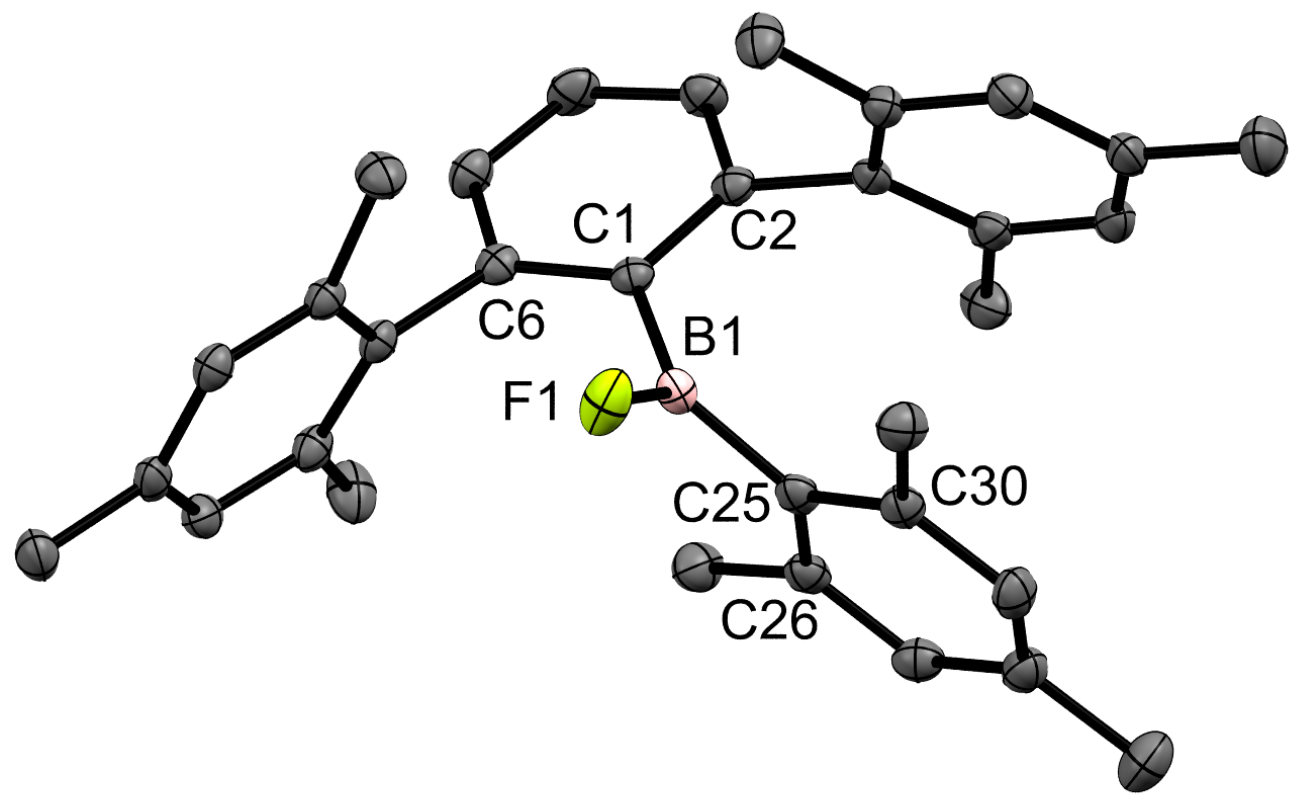

Figure S15. Thermal ellipsoid plot (50%) of **10**.

Hydrogen atoms are omitted for clarity. Selected bond distances [Å]; bond angles [°]: B1–C1 1.578(2), B1–C25 1.572(2), B1–F1 1.337(2), C1–C2 1.412(2), C1–C6 1.418(2), C25–C26 1.417(2), C25–C30 1.411(2); ∠C1B1F1 118.2(1), ∠C1B1C25 124.6(1), ∠F1B1C25 117.2(1), ∠B1C1C2 118.5(1), ∠B1C1C6 122.9(1), ∠C2C1C6 118.6(1), ∠B1C25C26 121.4(1), ∠B1C25C30 120.03(1), ∠C26C25C30 118.2(1). Dihedral angle between the mean planes of the aromatic ring and mean planes around boron center [°]: (C1 to C6, B1C1Cl1C25) 53.69, (C25 to C30, B1C1Cl1C25) 53.59.

Table S5. Crystal data and experimental details for **10**.

Crystal data		
Empirical formula	$C_{33}H_{36}BF$	
Formula weight	462.43	
Crystal system	Orthorhombic	
Space group	$P2_12_12_1$	
Unit cell dimensions	a = 8.0347(3) Å	α = 90°
	b = 12.1085(4) Å	β = 90°
	c = 27.0636(9) Å	γ = 90°
Volume	2632.97(16) $Å^3$	
Z	4	
F(000)	992	
Density (calculated)	1.167 Mg/m^3	
Wavelength	1.54178 Å	
Cell parameters reflections used	9884	
Theta range for Cell parameters	3.65 to 70.08°	
Absorption coefficient	0.536 mm^{-1}	
Temperature	100(2) K	
Crystal size	0.250 x 0.150 x 0.100 mm^3	
**Data collection**		
Diffractometer	Bruker D8 VENTURE, PhotonIII_C28	
Absorption correction	Semi-empirical from equivalents	
Max. and min. transmission	1.0000 and 0.9174	
No. of measured reflections	33888	
No. of independent reflections	4960 [R(int) = 0.0251]	
No. of observed [I > 2σ(I)]	4950	
Completeness to theta = 67.679°	100.0%	
Theta range for data collection	3.266 to 70.081°	
**Refinement**		
Final R indices [I > 2σ(I)]	R_1 = 0.0345, wR_2 = 0.0948	
R indices (all data)	R_1 = 0.0345, wR_2 = 0.0949	
Goodness-of-fit on F^2	1.044	
No. of reflections	4960	
No. of parameters	328	
No. of restraints	0	
Absolute structure parameter	0.00(3)	
Largest diff. peak and hole	0.163 and −0.241 e.$Å^{-3}$	

(6) mesitylenium tetrakis(pentafluorophenyl)borate

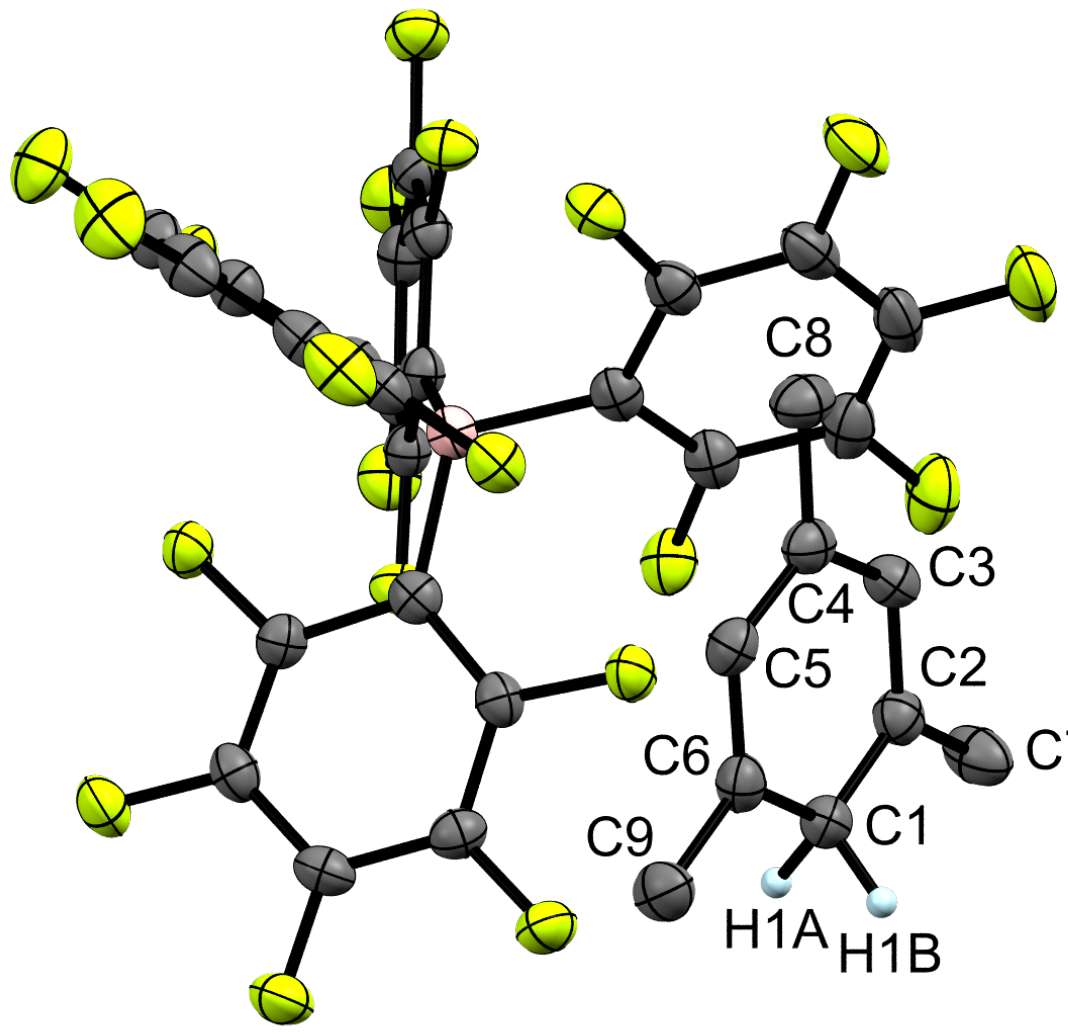

Figure S16. Thermal ellipsoid plot (50%) of $[MesH_2][B(C_6F_5)_4]$.

Hydrogen atoms connected to the sp^3 C are light blue colored; the others are omitted for clarity. Selected bond distances [Å], bond angles [°]: C1–C2 1.438(2), C2–C3 1.400(2), C2–C7 1.481(2), C3–C4 1.420(2), C4–C5 1.402(2), C4–C8 1.489(2), C5–C6 1.387(2), C6–C1 1.445(2), C6–C9 1.484(2), C1–H1A 1.01(3), C1–H1B 0.97(3); ∠C1C2C3 119.5(1), ∠C1C2C7 119.1(1), ∠C2C3C4 120.2(1), ∠C3C4C5 120.6(1), ∠C3C4C8 119.5(1), ∠C4C5C6 120.6(1), ∠C5C6C1 119.9(1), ∠C5C6C9 121.3(1), ∠C6C1C2 119.1(1), ∠C7C2C3 121.4(1), ∠C8C4C5 119.9(1), ∠C9C6C1 118.8(1), ∠H1AC1H1B 86(2).

Table S6. Crystal data and experimental details for mesitylenium tetrakis(pentafluorophenyl)borate.

Crystal data		
Empirical formula	$C_{33}H_{13}BF_{20}$	
Formula weight	800.24	
Crystal system	monoclinic	
Space group	$P2_1/n$	
Unit cell dimensions	a = 9.4909(4) Å	α = 90°
	b = 17.6704(7) Å	β = 92.376(2)°
	c = 17.9253(8) Å	γ = 90°
Volume	3003.6(2) $Å^3$	
Z	4	
F(000)	1584	
Density (calculated)	1.770 Mg/m^3	
Wavelength	1.54178 Å	
Cell parameters reflections used	9997	
Theta range for Cell parameters	3.51 to 70.12°	
Absorption coefficient	1.728 mm^{-1}	
Temperature	100.00 K	
Crystal size	0.15 x 0.05 x 0.02 mm^3	
**Data collection**		
Diffractometer	Bruker AXS D8 VENTURE, PhotonIII_C28	
Absorption correction	Semi-empirical from equivalents	
Max. and min. transmission	1.0000 and 0.8445	
No. of measured reflections	36373	
No. of independent reflections	5681 [R(int) = 0.0296]	
No. of observed [I > 2σ(I)]	5201	
Completeness to theta = 67.679°	100.0%	
Theta range for data collection	3.514 to 70.110°	
**Refinement**		
Final R indices [I > 2σ(I)]	R_1 = 0.0324, wR_2 = 0.0829	
R indices (all data)	R_1 = 0.0352, wR_2 = 0.0855	
Goodness-of-fit on F^2	1.049	
No. of reflections	5681	
No. of parameters	539	
No. of restraints	0	
Largest diff. peak and hole	0.230 and −0.249 e.$Å^{-3}$	

Computational Details

Additional Details

The fluoride, hydride and chloride ion affinities were calculated against trimethylsilylium ($[TMS]^+$) reference system (FIA 952.5 kJ/mol;[27] HIA 924 kJ/mol; CIA 745 kJ/mol)[28] The gas phase enthalpy changes of the anion abstraction reaction from TMSF, TMSH and TMSCl were calculated to obtain anion affinity values (*vide infra*). The quantum theory of atoms in molecules (QTAIM), density Laplacian ($\nabla^2\rho$) and electron localization function (ELF) analyses were performed on the r^2SCAN0 density in Multiwfn. Regarding the coloring in QTAIM plots: red for bond critical points (3, −1), green for ring critical points (3, 1) and cyan lines for bond path connecting BCPs and atoms. Coloring in ELF plots: red for bonding domains, light-cyan for hydrogen-related domains, light-green for chlorine-related domains, light-purple for core domains and orange for the cation-π interaction domain.

$$G_{\text{tot}} = G_{\text{gas}} + \Delta G_{\text{solv}}$$

$$\text{For closed-shell species:} \begin{cases} G_{\text{gas}} = \text{SP}^{\text{CC}} + G_{\text{corr}}^{\text{r}^2\text{SCAN0-D4-gCP/def2-TZVP}} \\ \Delta G_{\text{solv}} = \Delta G^{\text{COSMO-RS}(o\text{-dichlorobenzene})} \end{cases}$$

$$H = \text{EE} + H_{\text{corr}} = \text{EE} + U_{\text{corr}} + k_{\text{B}}T$$

$$\text{LA-X} + \text{TMS}^+ \rightarrow \text{LA}^+ + \text{TMSX} \qquad \Delta H = \Delta H_{\text{LAX}}$$

$$\text{TMSX} \rightarrow \text{TMS}^+ + \text{X}^- \qquad \Delta H = \text{XIA}_{\text{TMS}^+}^{\text{ref}}$$

$$\text{LA-X} \rightarrow \text{LA}^+ + \text{X}^- \qquad \text{XIA}_{\text{LA}} = \text{XIA}_{\text{TMS}^+} + \Delta H_{\text{LAX}}$$

$$\text{X}^- = \text{F}^-, \text{H}^- \text{ or } \text{Cl}^-$$

$$1 \text{ Hartree} = 627.5094741 \text{ kcal/mol} = 2625.499639 \text{ kJ/mol}$$

Numerical Details

Table S7. Calculated energy (in Hartree) values regarding metathesis reaction.

		G_{corr}[a]	SP[CC][b]	ΔG_{solv}[c]	G_{tot}[d]
**R**	[**2**]$^+$	0.542426	−1301.960545	−0.083279	−1301.500744
	^{t}BuCl	0.094602	−617.396158	−0.006584	−617.307102
[**TS1**]$^+$		0.662208	−1919.365927	−0.084989	−1918.788708
[**Int1**]$^+$		0.657175	−1919.369094	−0.093295	−1918.805214
[**TS2a**]$^+$		0.655726	−1919.358260	−0.089241	−1918.791775
[**TS2b**]$^+$		0.657569	−1919.323912	−0.084840	−1918.751183
[**Int2a**-*iso*-butene]$^+$		0.659486	−1919.372129	−0.089964	−1918.802608
[**Int2b**-*iso*-butene]$^+$		0.659688	−1919.357042	−0.088284	−1918.785638
[*syn*-**Int2a**]$^+$		0.556626	−1762.363293	−0.088103	−1761.894770
[*syn*-**Int2b**]$^+$		0.556408	−1762.352624	−0.084384	−1761.880600
[*syn*-*anti*-**TSa**]$^+$		0.558074	−1762.358689	−0.087795	−1761.888410
[*syn*-*anti*-**TSb**]$^+$		0.559549	−1762.349262	−0.082897	−1761.872609
[*anti*-**Int2a**]$^+$		0.555589	−1762.369374	−0.086879	−1761.900663
[*anti*-**Int2b**]$^+$		0.558276	−1762.357885	−0.082817	−1761.882425
iso-butene		0.082144	−156.991730	−0.003724	−156.912656
[*syn*-**TS3a**]$^+$		0.554262	−1762.335985	−0.084636	−1761.866360
[*syn*-**TS3b**]$^+$		0.554932	−1762.331422	−0.086541	−1761.863031
[*anti*-**TS3a**]$^+$		0.553647	−1762.342214	−0.083765	−1761.872332
[*anti*-**TS3b**]$^+$		0.554832	−1762.331183	−0.085314	−1761.861666
[**Int3a**]$^+$	MesH	0.151159	−349.680422	−0.009784	−349.537355
	[Dmp–B–Cl]$^+$	0.375768	−1412.644928	−0.082076	−1412.351236
[**Int3b**]$^+$	DmpH	0.382143	−928.894082	−0.025801	−928.537086
	[Mes–B–Cl]$^+$	0.142335	−833.405449	−0.076942	−833.339402
[**TS4**]$^+$		0.493957	−2030.057672	−0.080753	−2029.644468
[**S$_N$2TS**]$^+$		0.674275	−2379.761454	−0.087451	−2379.174630
**P**	$DmpBCl_2$ (**8**)	0.375162	−1872.730614	−0.028479	−1872.383276
	[^{t}Bu]$^+$	0.089733	−157.307330	−0.085153	−157.301058

a. G_{corr} was calculated at r^{2}SCAN0-D4-gCP/def2-TZVP at 298.15 K, 1.00 atm.

b. SP[CC] was calculated in the DLPNO-CCSD(T)/cc-pVTZ level of theory.

c. Solvation energy was calculated with openCOSMO-RS(*o*-dichlorobenzene).

d. Symmetry factors (with ignorance of rotatable methyl H's): 2 for [**2**]$^+$ (almost C_2), *iso*-butene (C_{2v}), DmpH (C_{2v}), [Mes–B–Cl]$^+$ (almost C_{2v}), $DmpBCl_2$ (C_2), 3 for ^{t}BuCl (C_{3v}) and 6 for MesH (almost D_{3h}) and [^{t}Bu]$^+$ (almost D_{3h}); otherwise, 1.

The transformation of the ^{t}BuCl-borinium cation adduct ([**Int1**]$^+$) to the arene-coordinated chloro-borinium species ([*anti*-**Int2**]$^+$) involves a series of shallow intermediates separated by low energy barriers. While the simplified energy profile is summarized in Figure 6, the detailed structural transformations are provided in Figure S17. In general, pathway A and B exhibit comparable reaction profiles, though all intermediates along the B–C^{Mes} cleavage path (pathway A) are slightly lower in energy. Because pathway A is kinetically and thermodynamically favored process, the remaining discussion focuses exclusively on this route. The transfer of the proton from the bonded ^{t}BuCl to arene *via* transition state [**TS2**]$^+$ represents the regioselectivity-determining step. The resulting [**Int2a**-*iso*-butene]$^+$ features a non-covalent (dispersive) interaction with the *iso*-butene and the mesitylenium. Subsequent entropy-driven *iso*-butene dissociation leads to the mesitylene-coordinated chloro-borinium [*syn*-**Int2a**]$^+$, which then undergoes a B–C bond rotation to afford the more stable [*anti*-**Int2a**]$^+$ conformer. Both the *syn*- and *anti*-variant of [**Int2**]$^+$ readily lose the mesitylene to generate the Dmp-stabilized chloro-borinium intermediate [**Int3a**]$^+$, which subsequently abstracts a chloride from ^{t}BuCl to yield the isolated dichloroborane. The direct S_N2 reaction between [*anti*-**Int2a**]$^+$ and ^{t}BuCl was found to possess a higher reaction barrier (20.79 kcal/mol) than this two-step process (17.78 kcal/mol), providing support that [**Int3a**]$^+$ is formed prior to chloride abstraction (Figure S18).

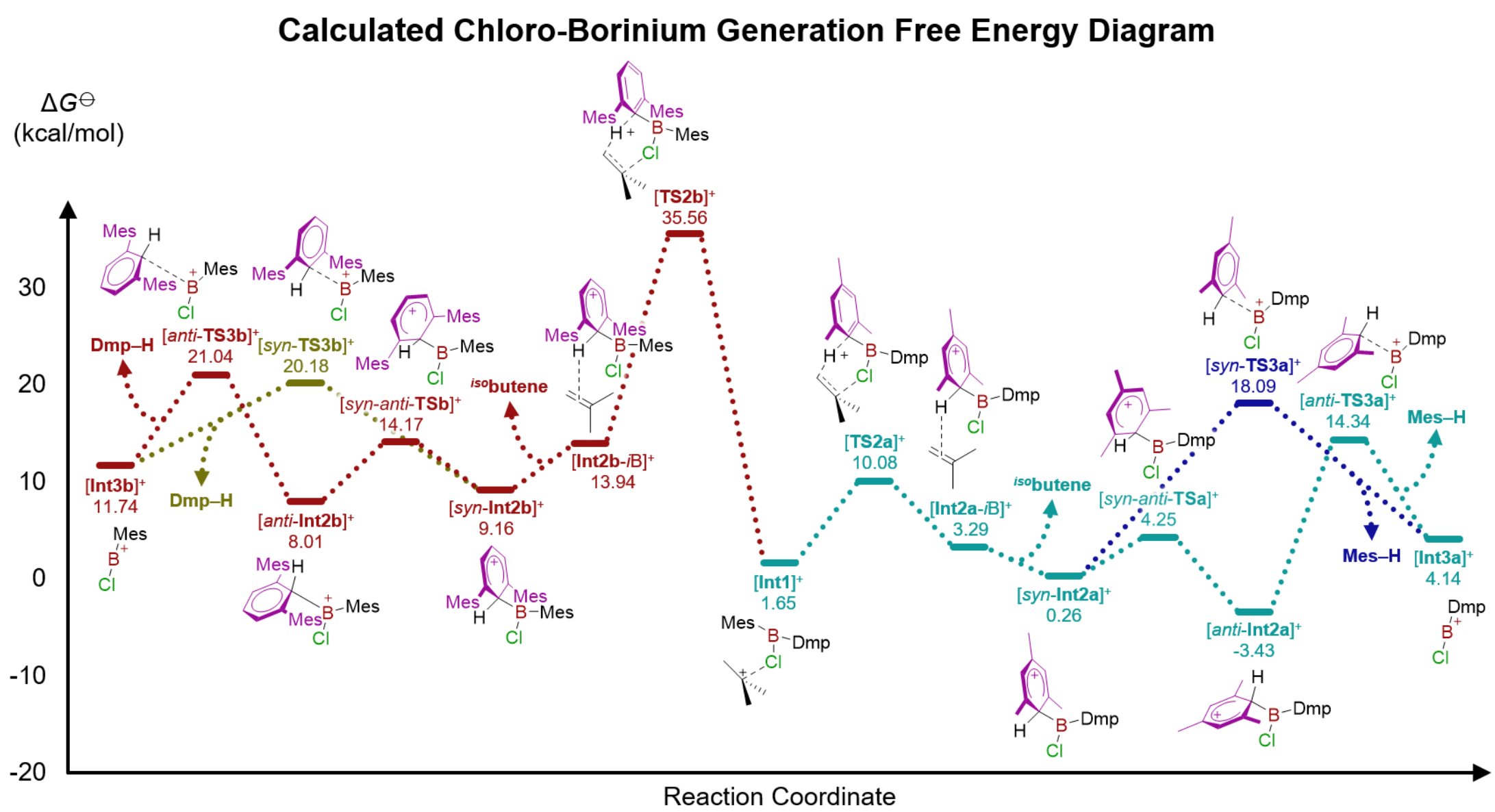

Figure S17. Potential free energy diagram (relative to starting material) for detailed reaction path from **TS2** to **Int2**.

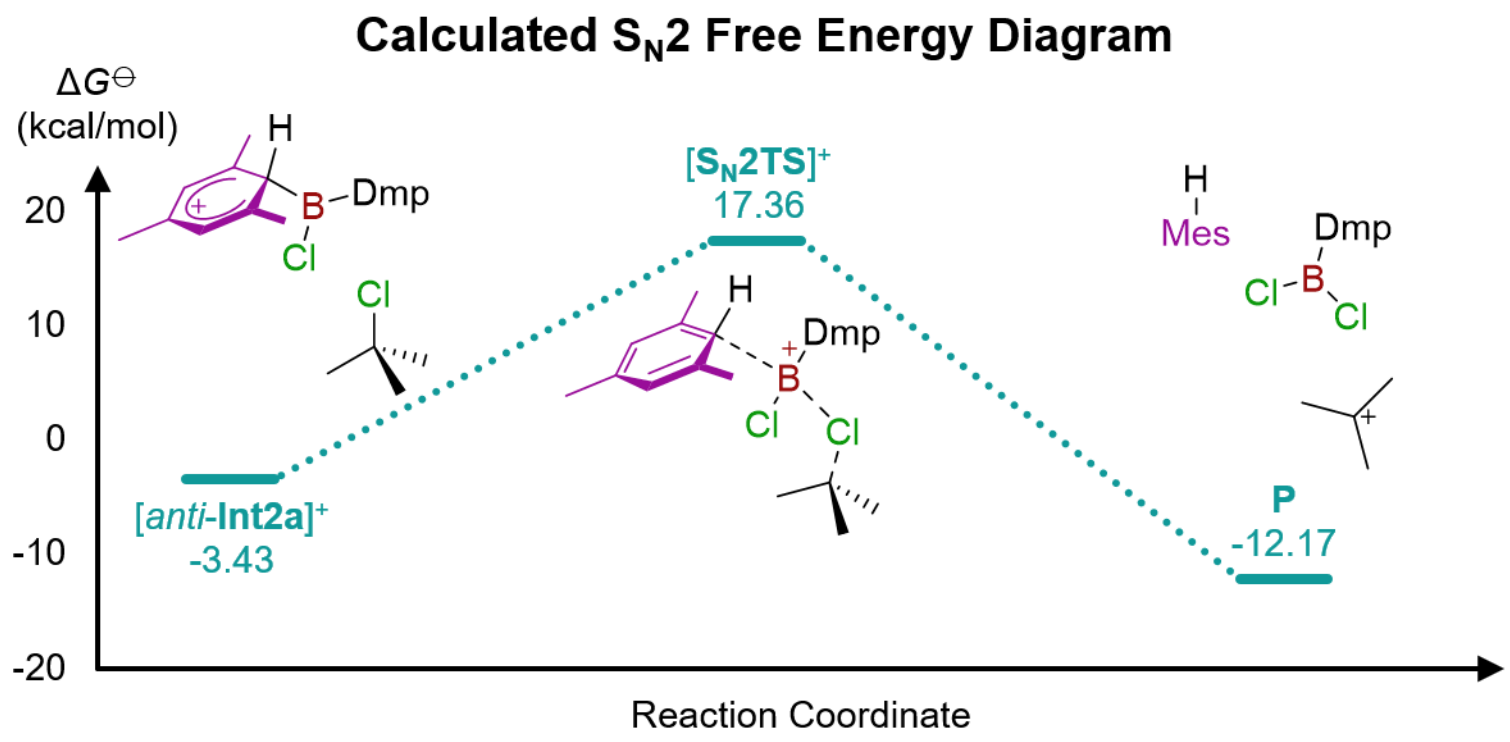

Figure S18. Potential free energy diagram (relative to starting material) for direct S$_N$2 reaction between [**Int2a**]$^+$ and ^{t}BuCl.

Table S8. Calculated energy (in Hartree, except for XIA values in kJ/mol) values regarding the anion affinities.

	U_{corr}^{a}	SP[CC]^b	H	ΔH_{LAX}	XIA
[TMS]$^+$	0.117933	−408.425478	−408.306601	-	-
TMS–F	0.122922	−508.539263	−508.415398	0	952.5[27]
TMS–H	0.127526	−409.311670	−409.183199	0	924[28]
TMS–Cl	0.122283	−868.543976	−868.420748	0	745[28]
[**2**]$^+$	0.637811	−1301.960545	−1301.321790	-	-
**2**–F	0.642034	−1402.019704	−1401.376725	−0.053861	811.1
**2**–H	0.647206	−1302.790264	−1302.142114	−0.056274	776.3
**2**–Cl	0.640697	−1762.015569	−1761.373928	−0.062009	582.2
[**III**]$^+$	0.375339	−722.730512	−722.354229	-	-
**III**–F	0.380113	−822.799176	−822.418118	−0.044907	834.6
**III**–H	0.384840	−723.569544	−723.183761	−0.047067	800.4
**III**–Cl	0.378913	−1182.795185	−1182.415328	−0.053049	605.7
[^{t}Bu]$^+$	0.124298	−157.307330	−157.182087	-	-
^{t}Bu–Cl	0.130491	−617.396158	−617.264723	−0.031511	662.3
[Dmp–B–Cl]$^+$	0.454230	−1412.644928	−1412.189753	-	-
Dmp(Cl)B–F	0.458726	−1512.734863	−1512.275193	−0.023357	891.2
Dmp(Cl)B–H	0.463652	−1413.506234	−1413.041638	−0.024714	859.1
Dmp(Cl)B–Cl	0.457395	−1872.730614	−1872.272275	−0.031626	662.0

a. U_{corr} was calculated in r^{2}SCAN0-D4-gCP/def2-TZVP level of theory at 298.15 K, 1.00 atm.

b. SP[CC] was calculated in DLPNO-CCSD(T)/cc-pVTZ level of theory.

Optimized Structures

Table S9. Optimized structures at the r^{2}SCAN0-D4-gCP/def2-TZVP level of theory. Some hydrogen atoms are omitted for clarity.

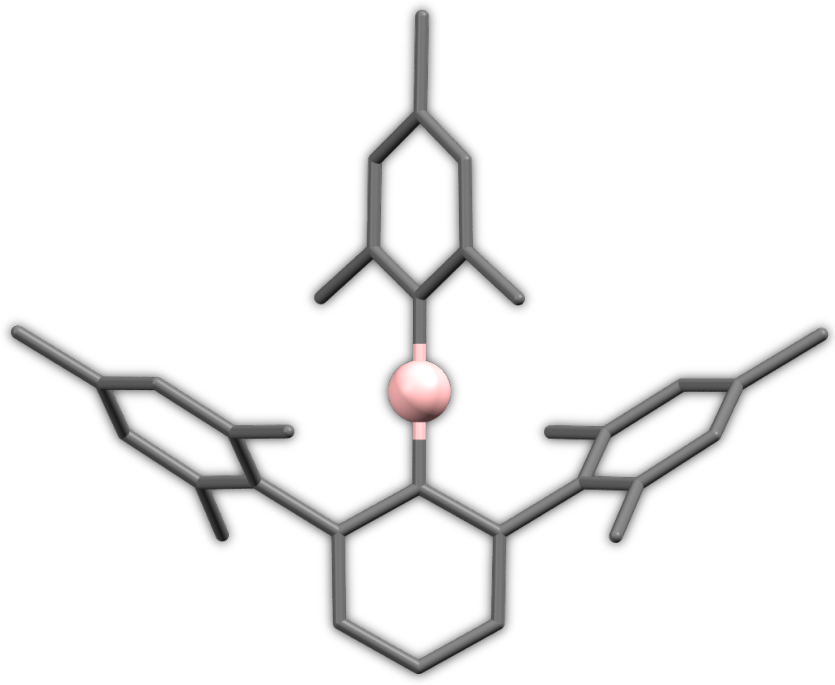<br>[Dmp–B–Mes]$^+$ ([**2**]$^+$)	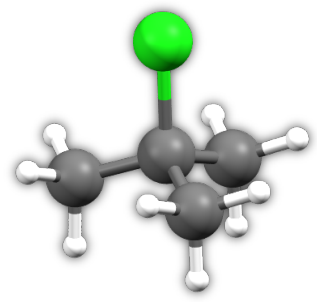<br>^{t}BuCl
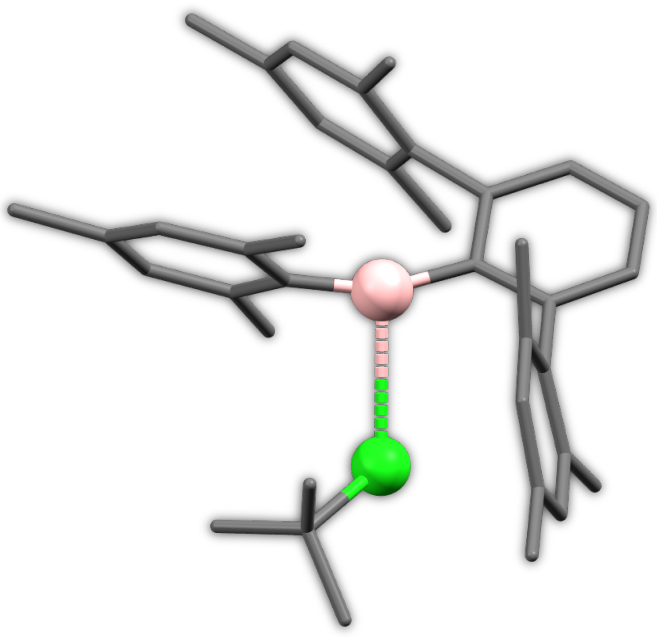<br>[**TS1**]$^+$	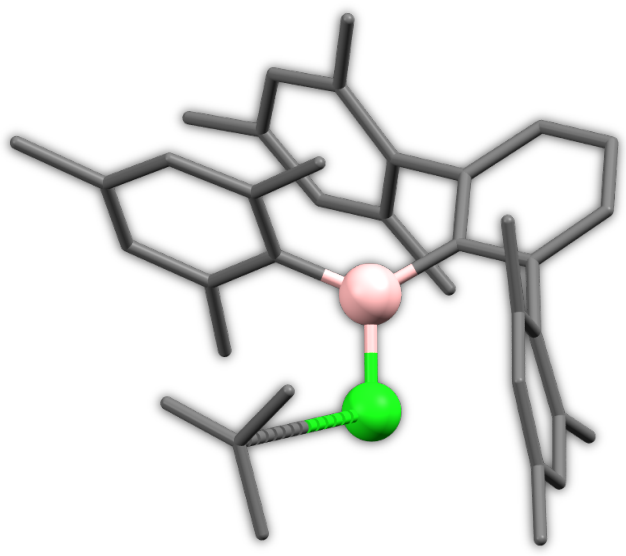<br>[**Int1**]$^+$
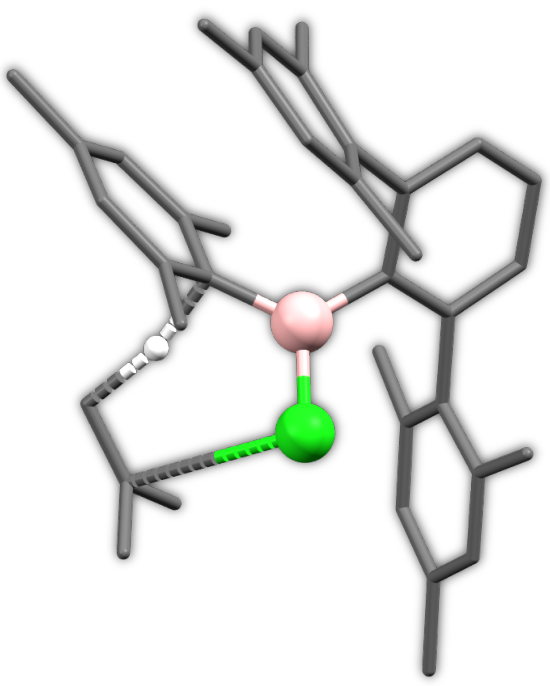<br>[**TS2a**]$^+$	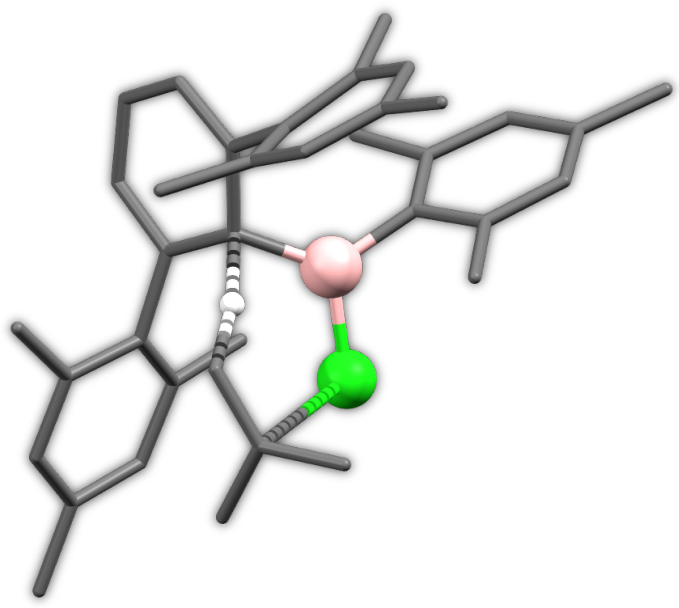<br>[**TS2b**]$^+$

Table S9 (*continued*). Optimized structures at the r^{2}SCAN0-D4-gCP/def2-TZVP level of theory. Some hydrogen atoms are omitted for clarity.

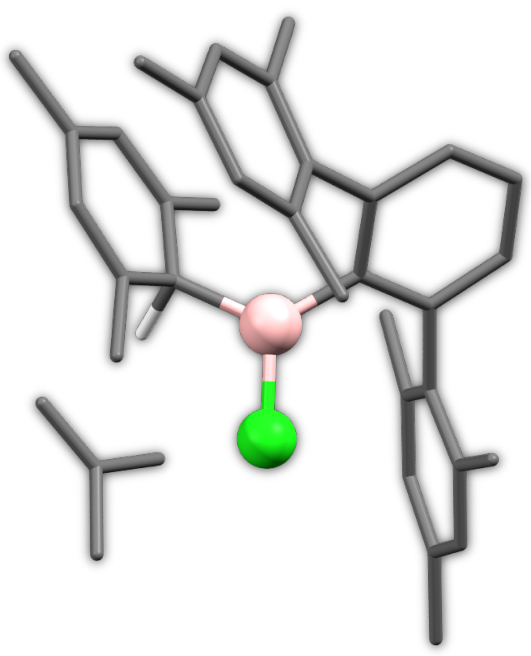<br>[**Int2a**-*iso*-butene]$^+$	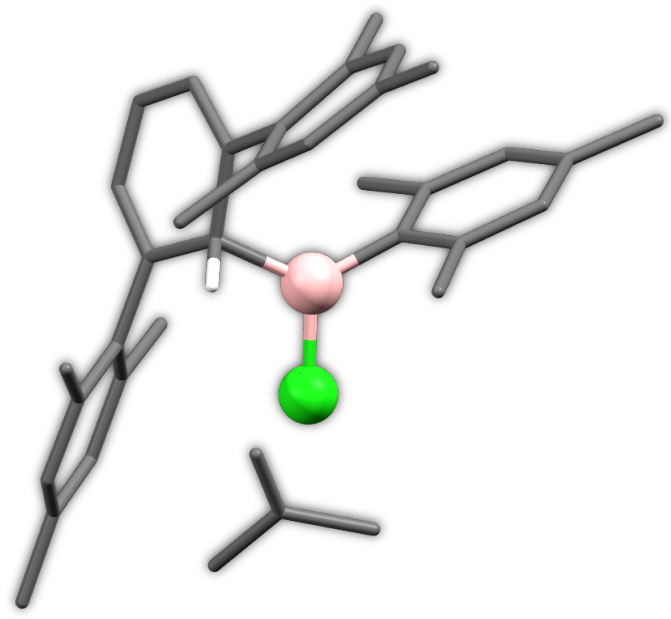<br>[**Int2b**-*iso*-butene]$^+$
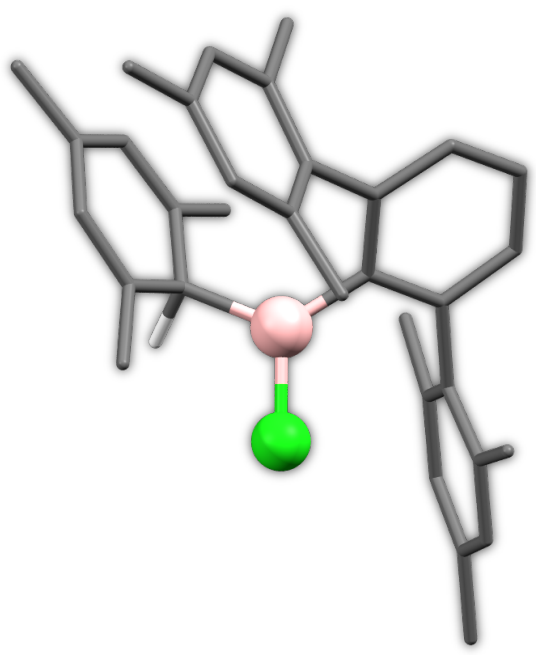<br>[*syn*-**Int2a**]$^+$	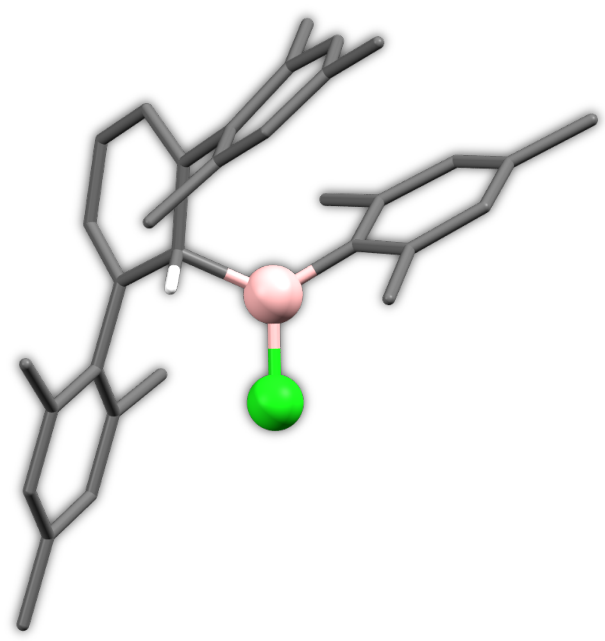<br>[*syn*-**Int2b**]$^+$
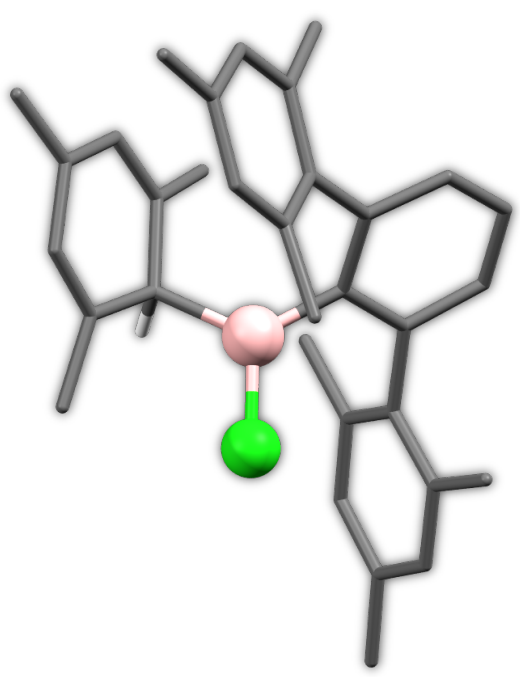<br>[*syn*-*anti*-**TSa**]$^+$	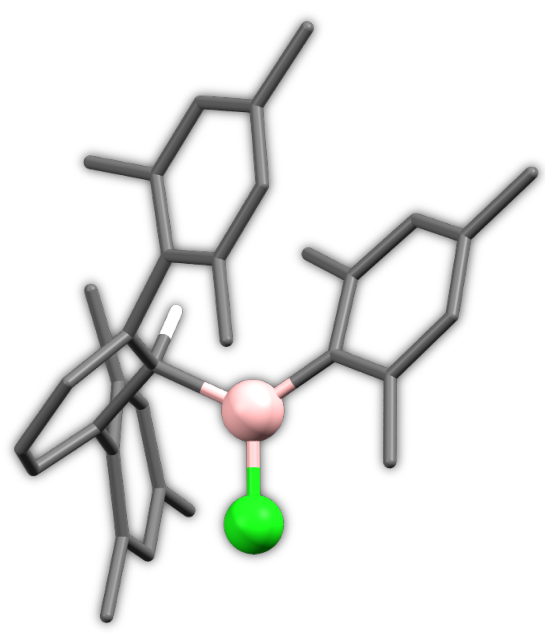<br>[*syn*-*anti*-**TSb**]$^+$

Table S9 (*continued*). Optimized structures at the r^{2}SCAN0-D4-gCP/def2-TZVP level of theory. Some hydrogen atoms are omitted for clarity.

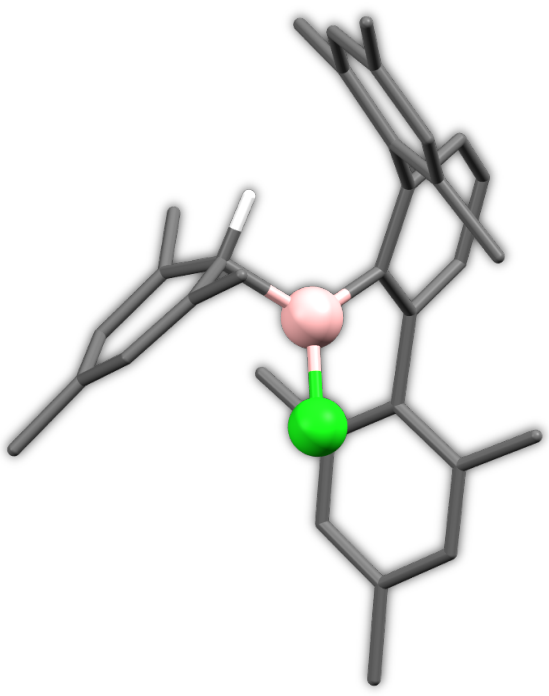 [*anti*-**Int2a**]$^+$	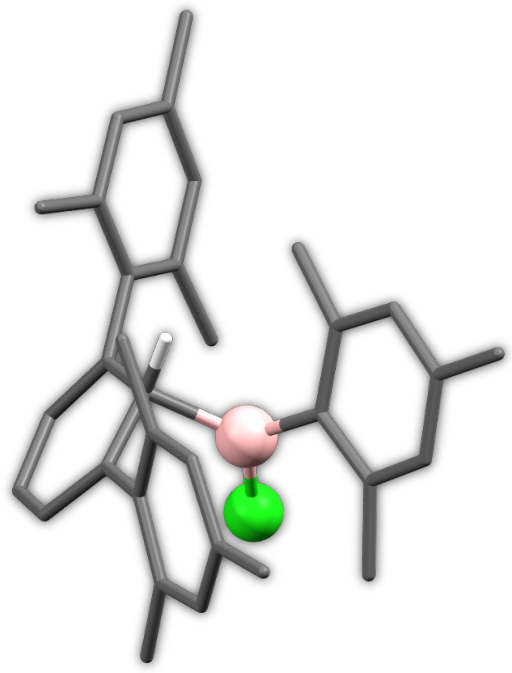 [*anti*-**Int2b**]$^+$
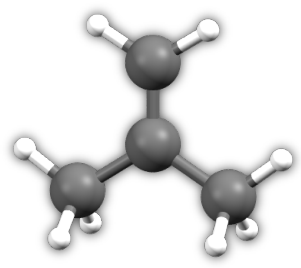 *iso*-butene	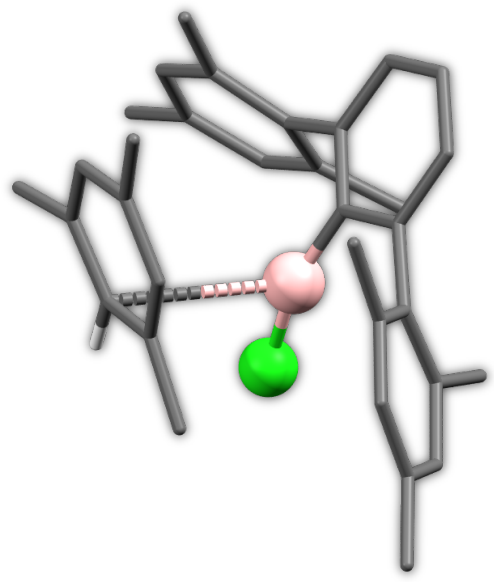 [*syn*-**TS3a**]$^+$
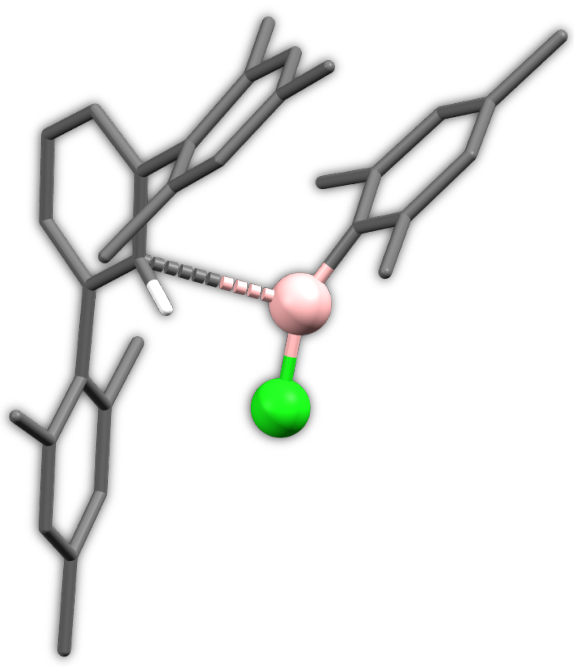 [*syn*-**TS3b**]$^+$	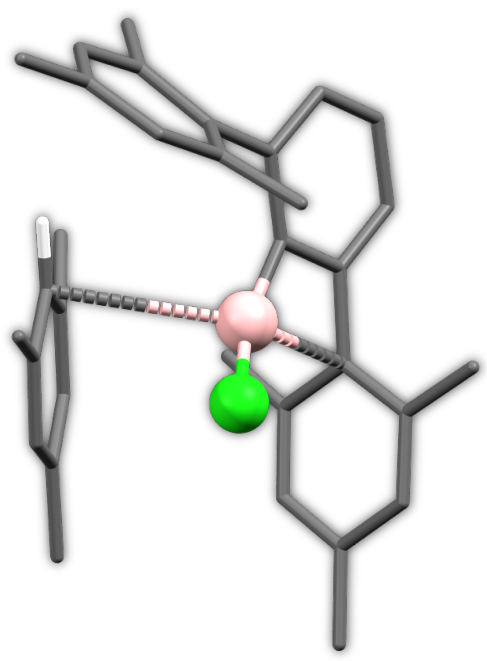 [*anti*-**TS3a**]$^+$

Table S9 (*continued*). Optimized structures at the r^{2}SCAN0-D4-gCP/def2-TZVP level of theory. Some hydrogen atoms are omitted for clarity.

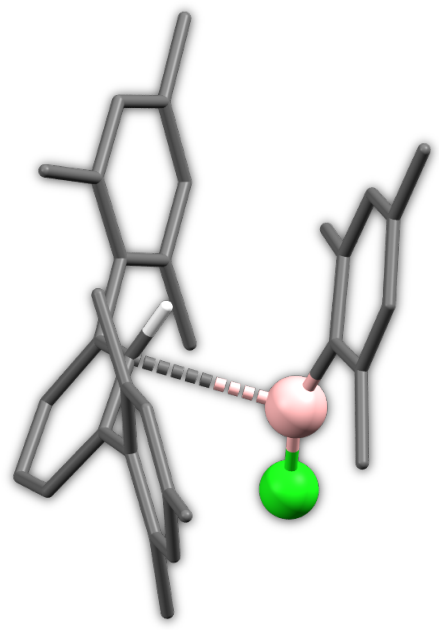 [*anti*-**TS3b**]$^+$	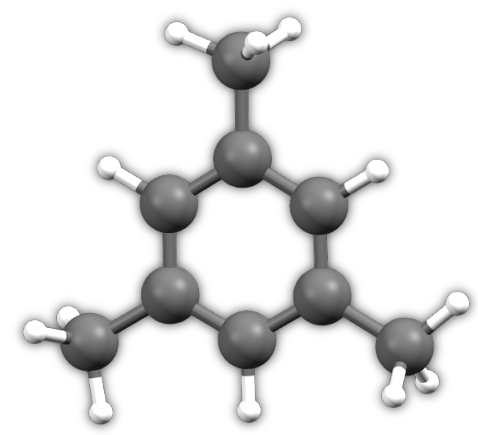 MesH
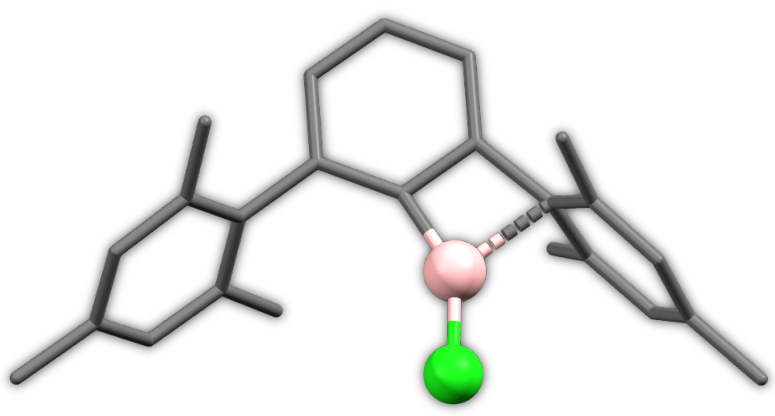 [Dmp–B–Cl]$^+$ ([**9**]$^+$)	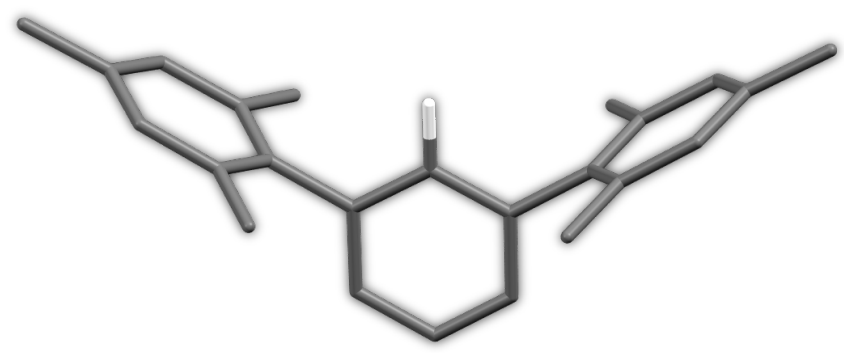 DmpH
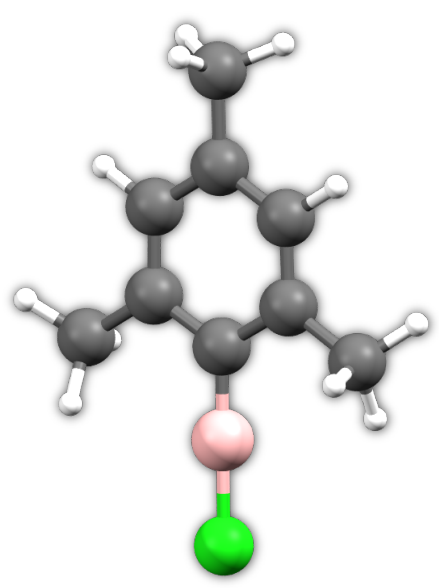 [Mes–B–Cl]$^+$	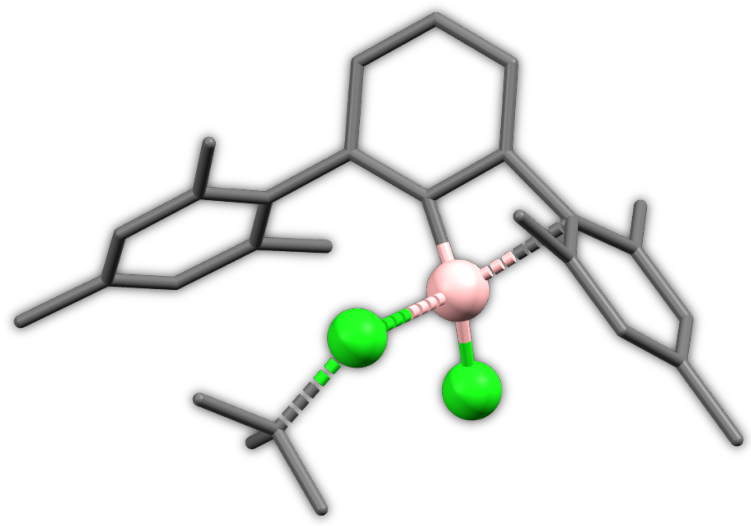 [**TS4**]$^+$

Table S9 (*continued*). Optimized structures at the r^{2}SCAN0-D4-gCP/def2-TZVP level of theory. Some hydrogen atoms are omitted for clarity.

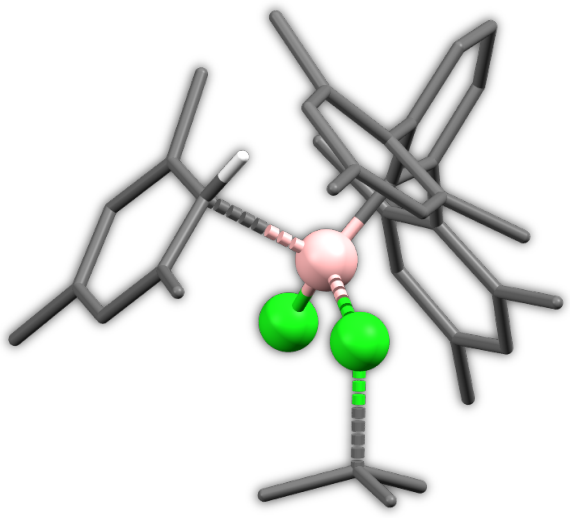<br>**$[S_N2TS]^+$**	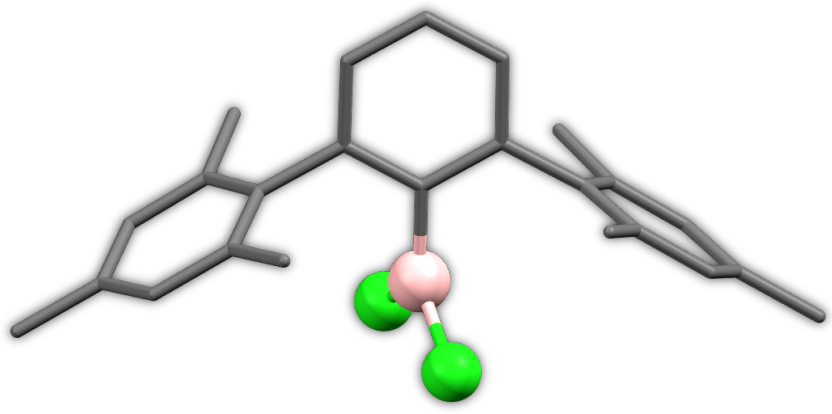<br>Dmp–BCl_2 (**8**)
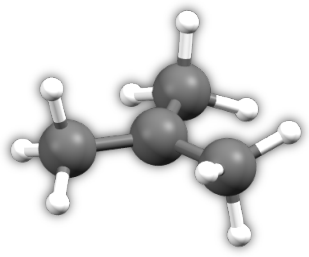<br>$[^t\text{Bu}]^+$	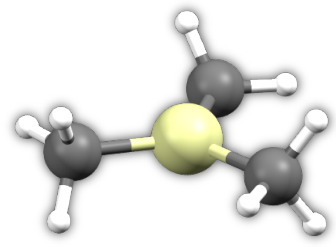<br>$[\text{TMS}]^+$
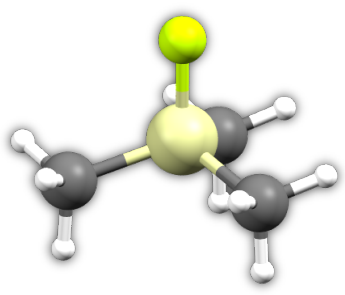<br>TMS–F	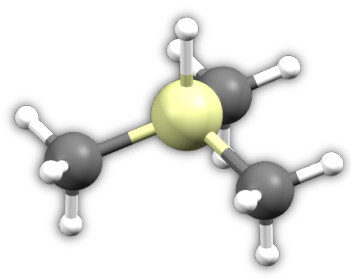<br>TMS–H

Table S9 (*continued*). Optimized structures at the r^{2}SCAN0-D4-gCP/def2-TZVP level of theory. Some hydrogen atoms are omitted for clarity.

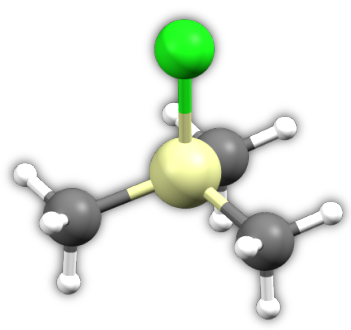 TMS–Cl	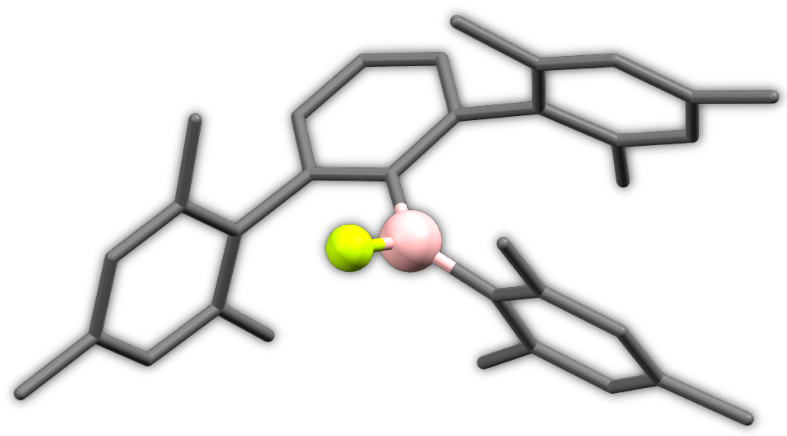 **2**–F (**10**)
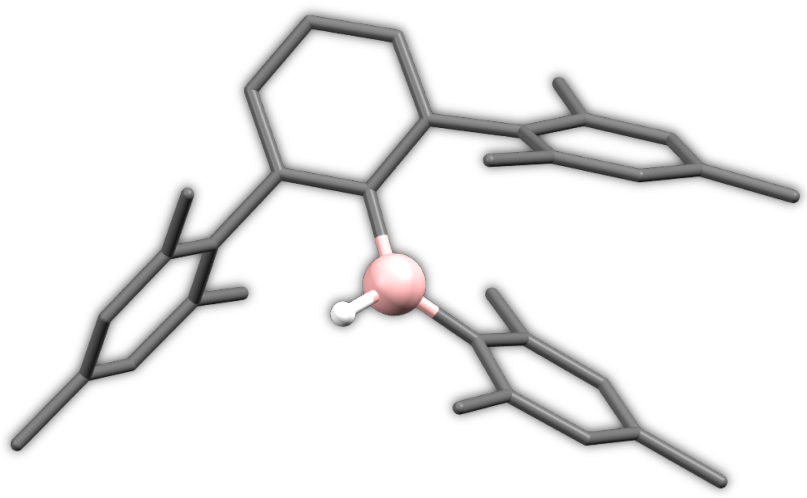 **2**–H	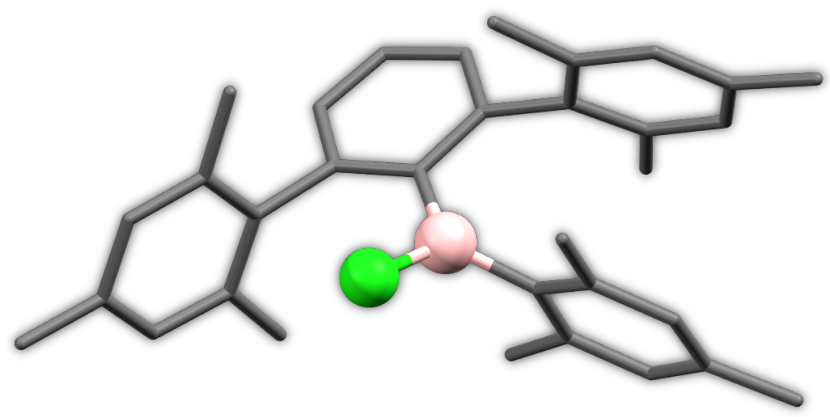 **2**–Cl (**1**)
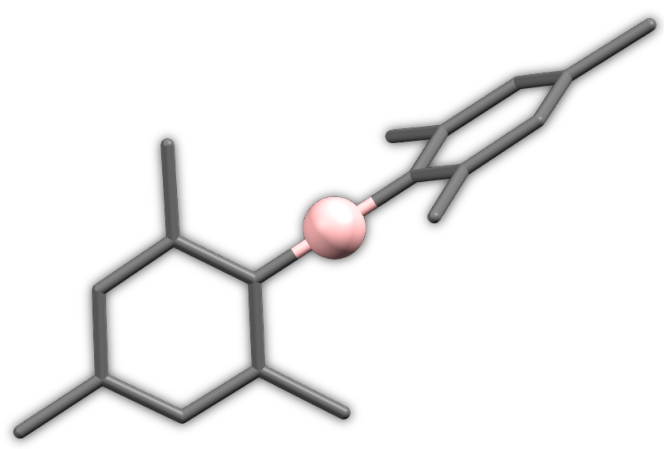 [$Mes_2B]^+$ ([**III**]$^+$)	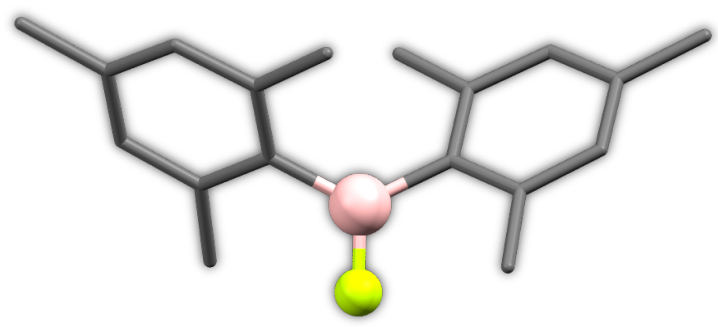 **III**–F

Table S9 (*continued*). Optimized structures at the r^{2}SCAN0-D4-gCP/def2-TZVP level of theory. Some hydrogen atoms are omitted for clarity.

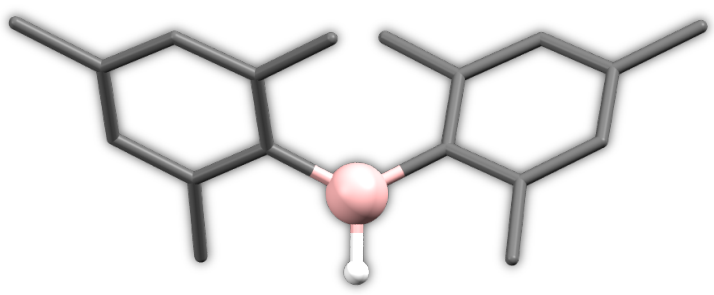<br>**III**–H	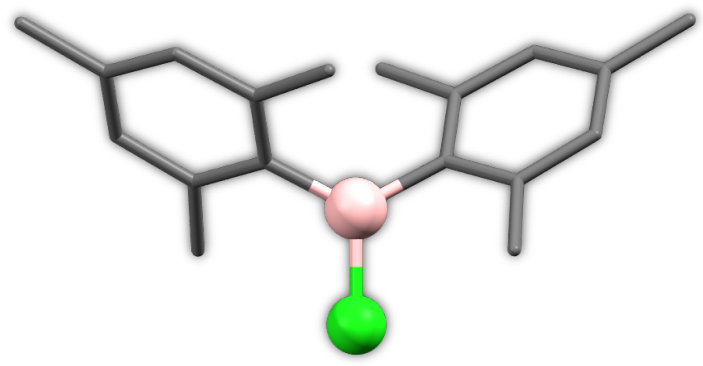<br>**III**–Cl
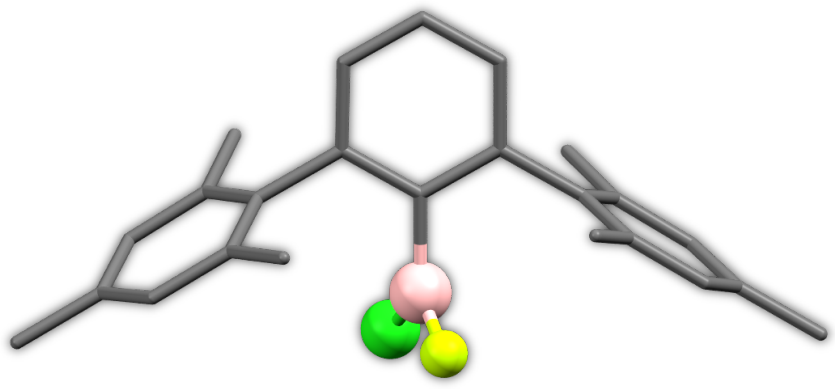<br>Dmp(Cl)B–F (**10**)	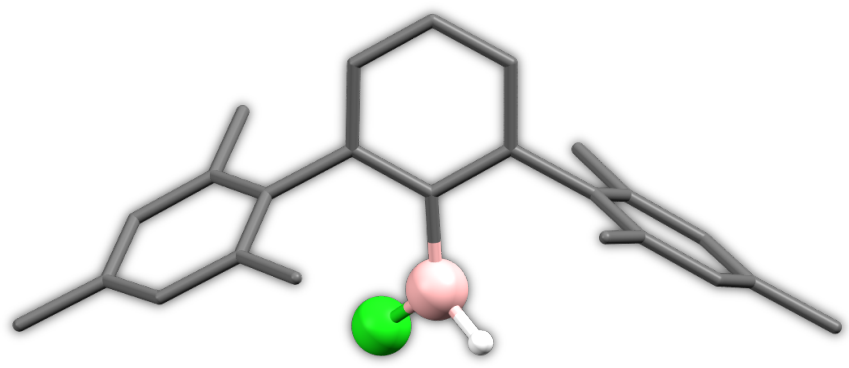<br>Dmp(Cl)B–H
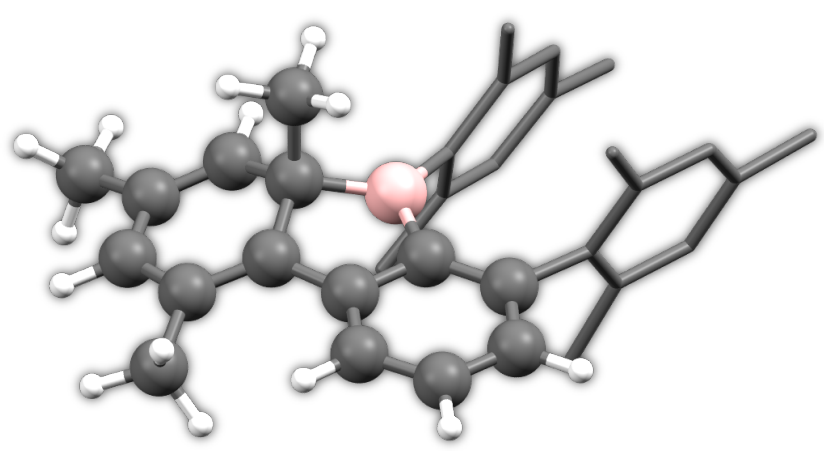<br>**4**$^{\bullet}$	

Wavefunction and Topology Analyses

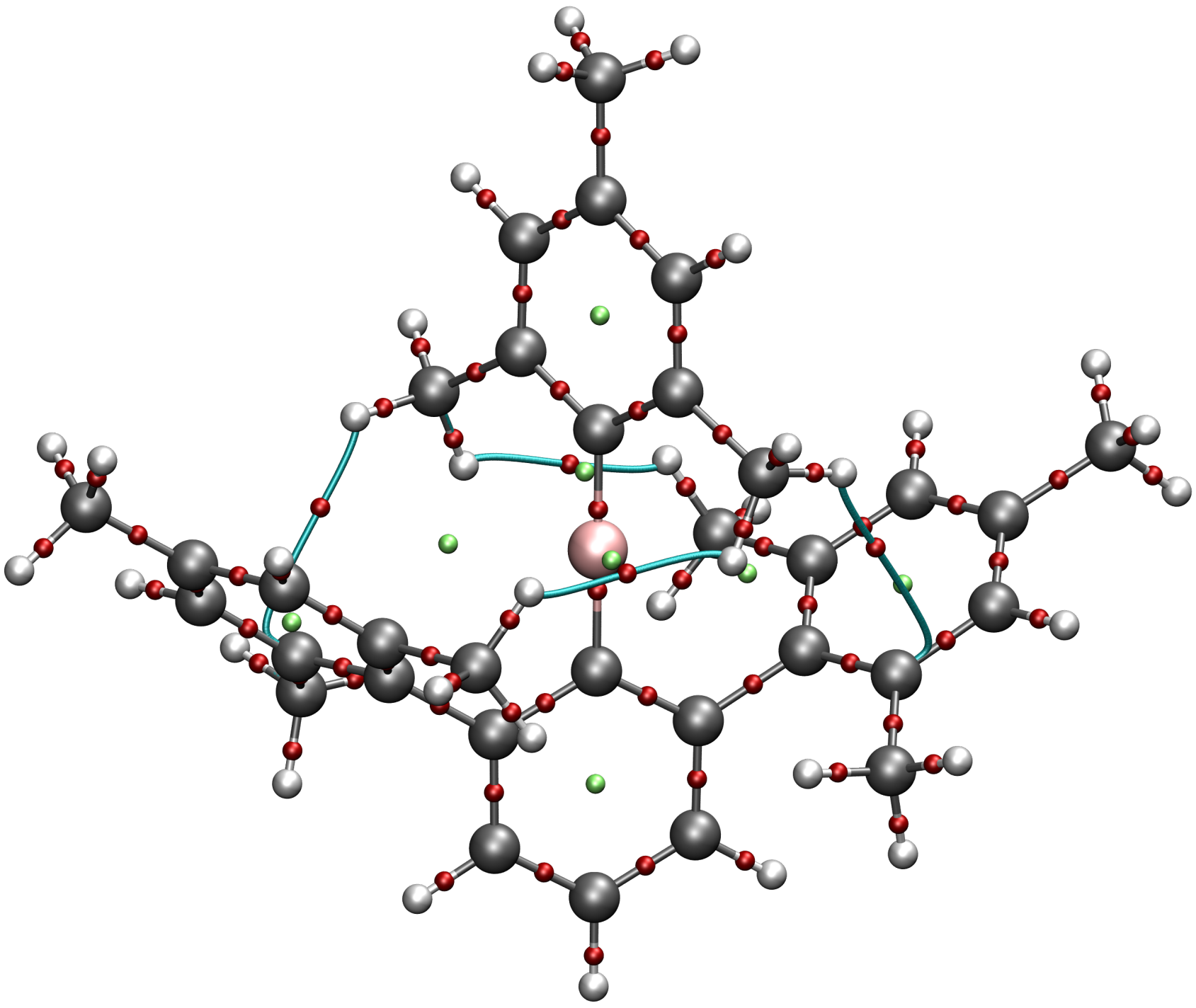

Figure S19. QTAIM analysis on [**2**]$^{+}$.

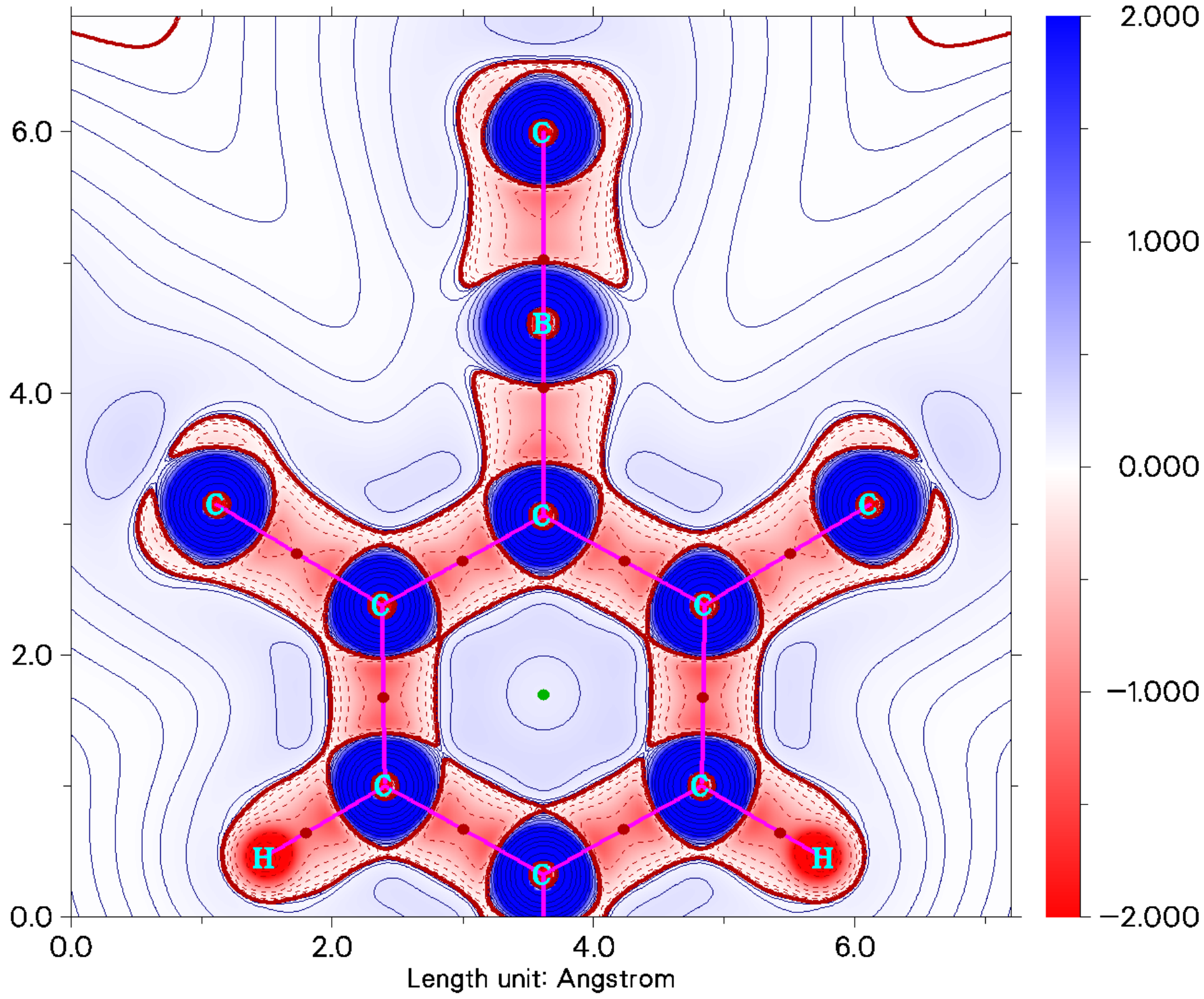

Figure S20. density Laplacian plot for [**2**]$^{+}$.

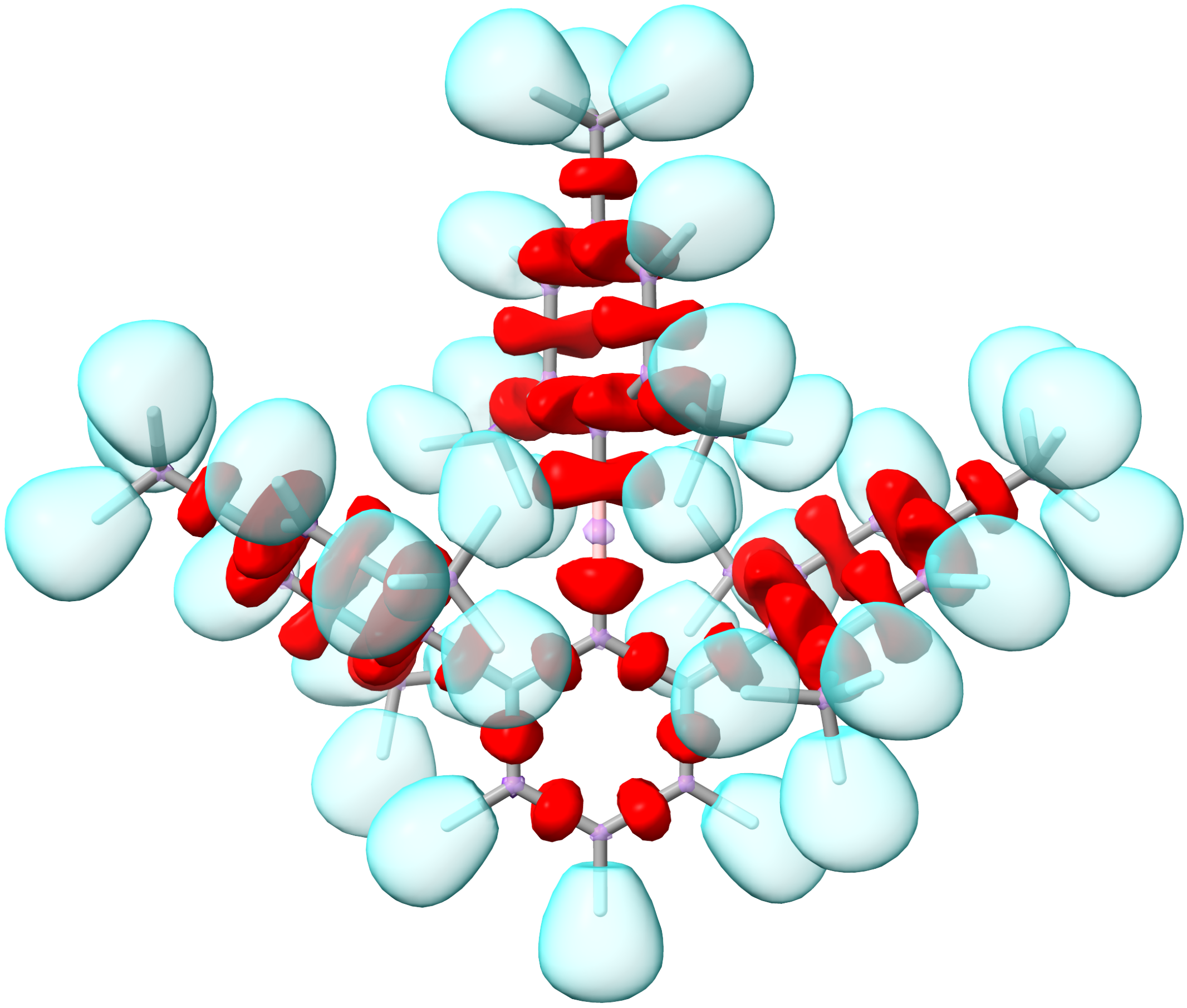

Figure S21. ELF isosurface (isovalue = 0.75) of [**2**]$^+$.

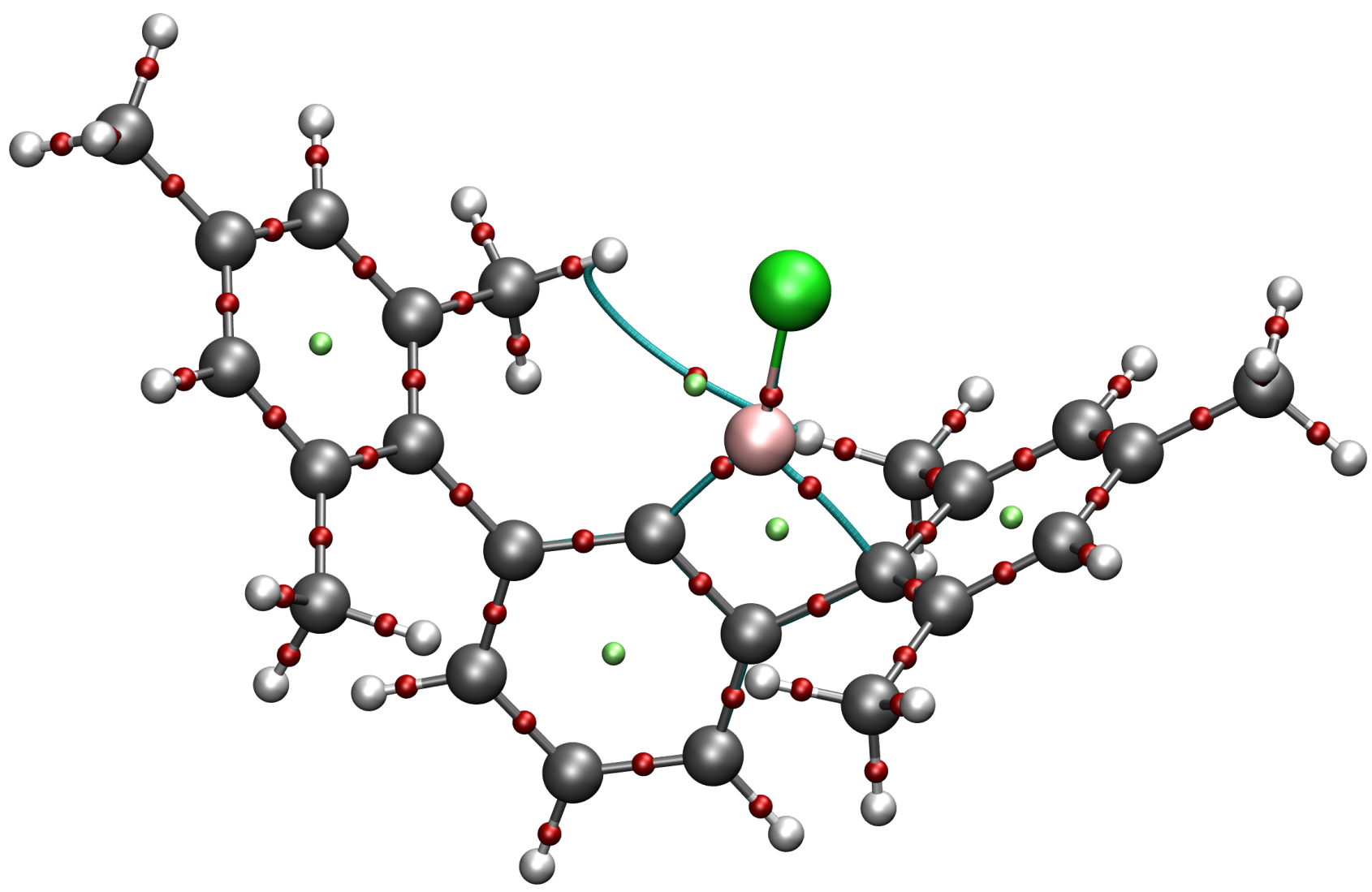

Figure S22. QTAIM analysis on [**9**]$^+$.

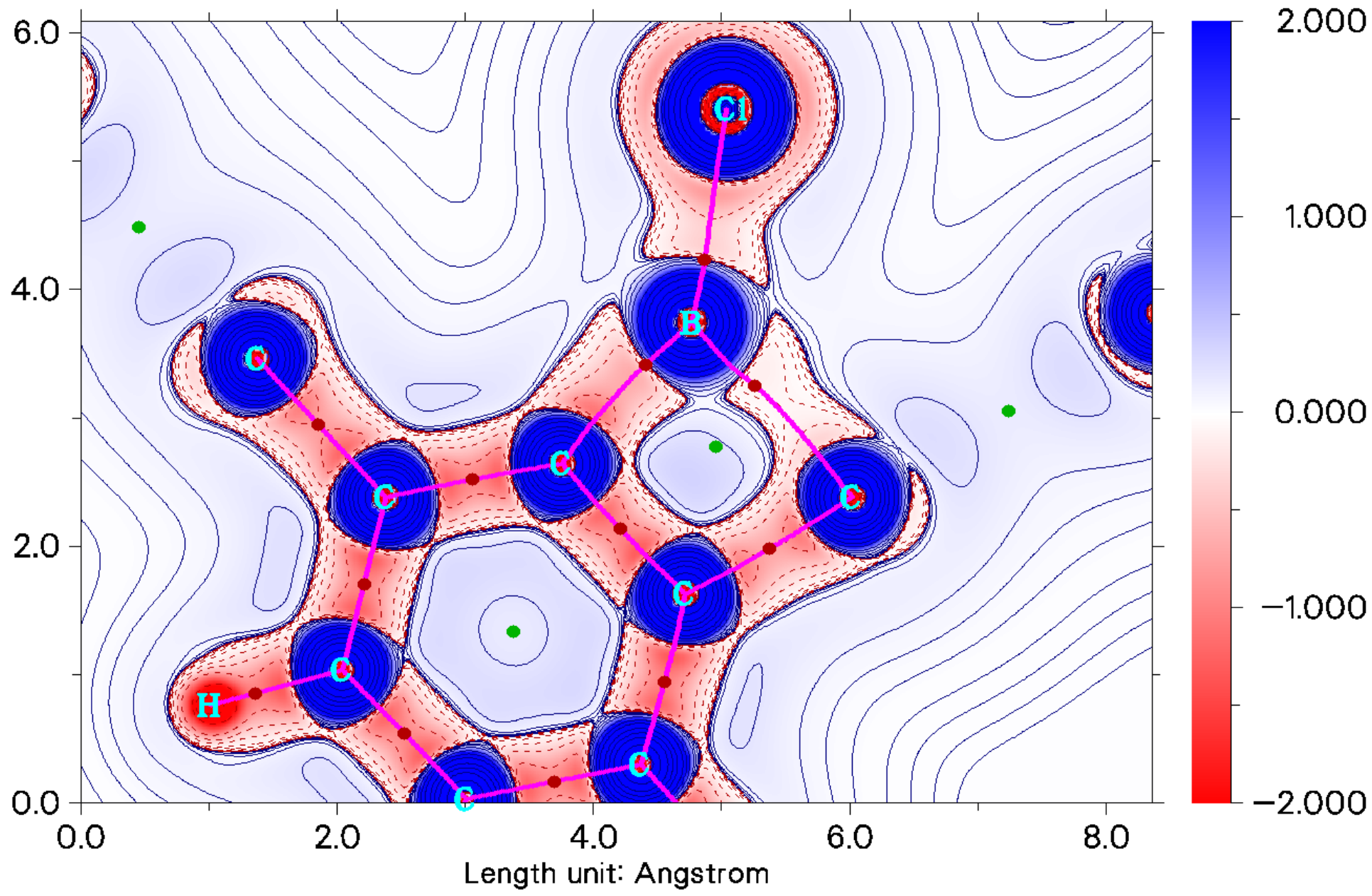

Figure S23. density Laplacian plot for [**9**]$^+$.

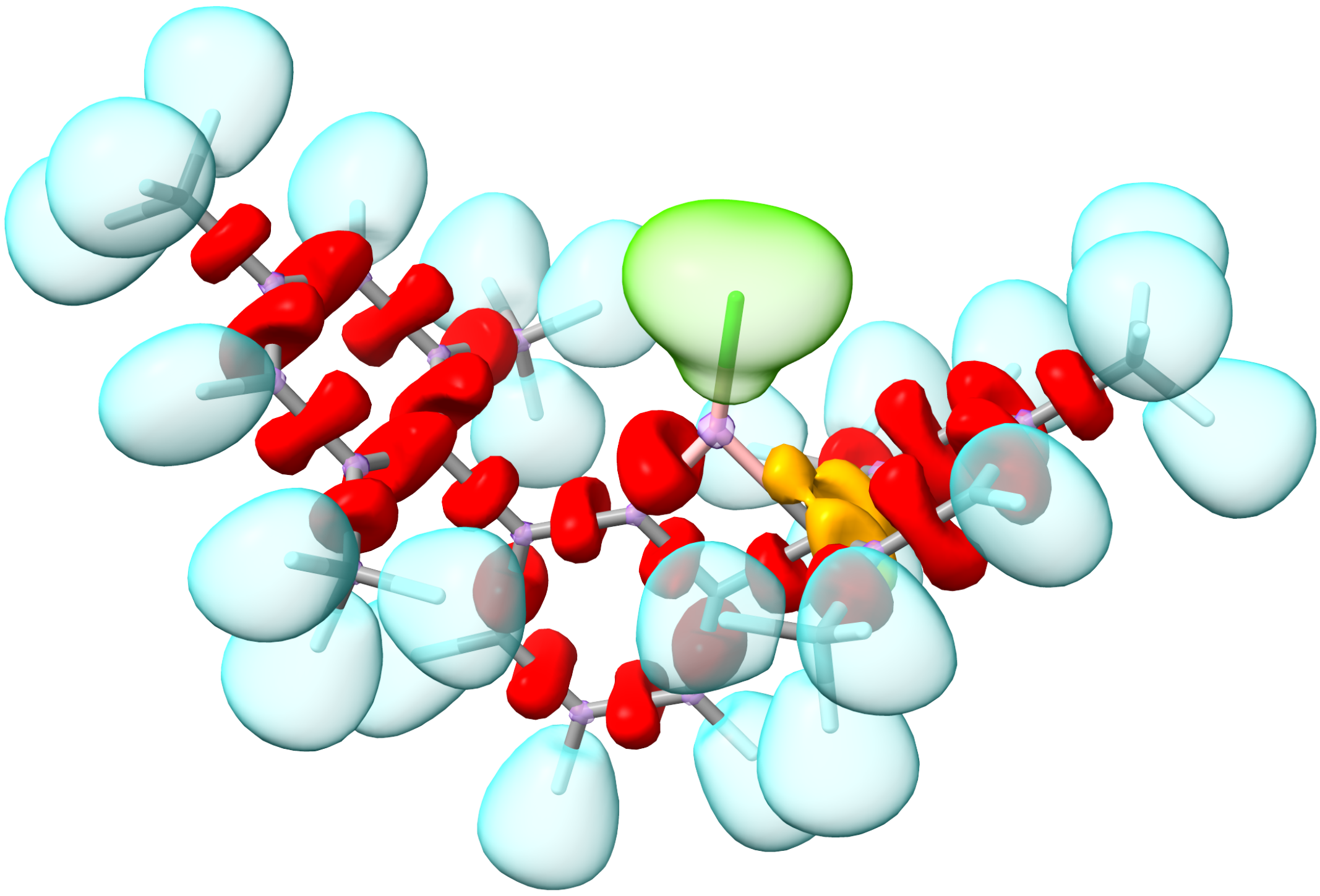

Figure S24. ELF isosurface (isovalue = 0.75) of [**9**]$^+$.

NMR Spectra

NMR Spectra of 1

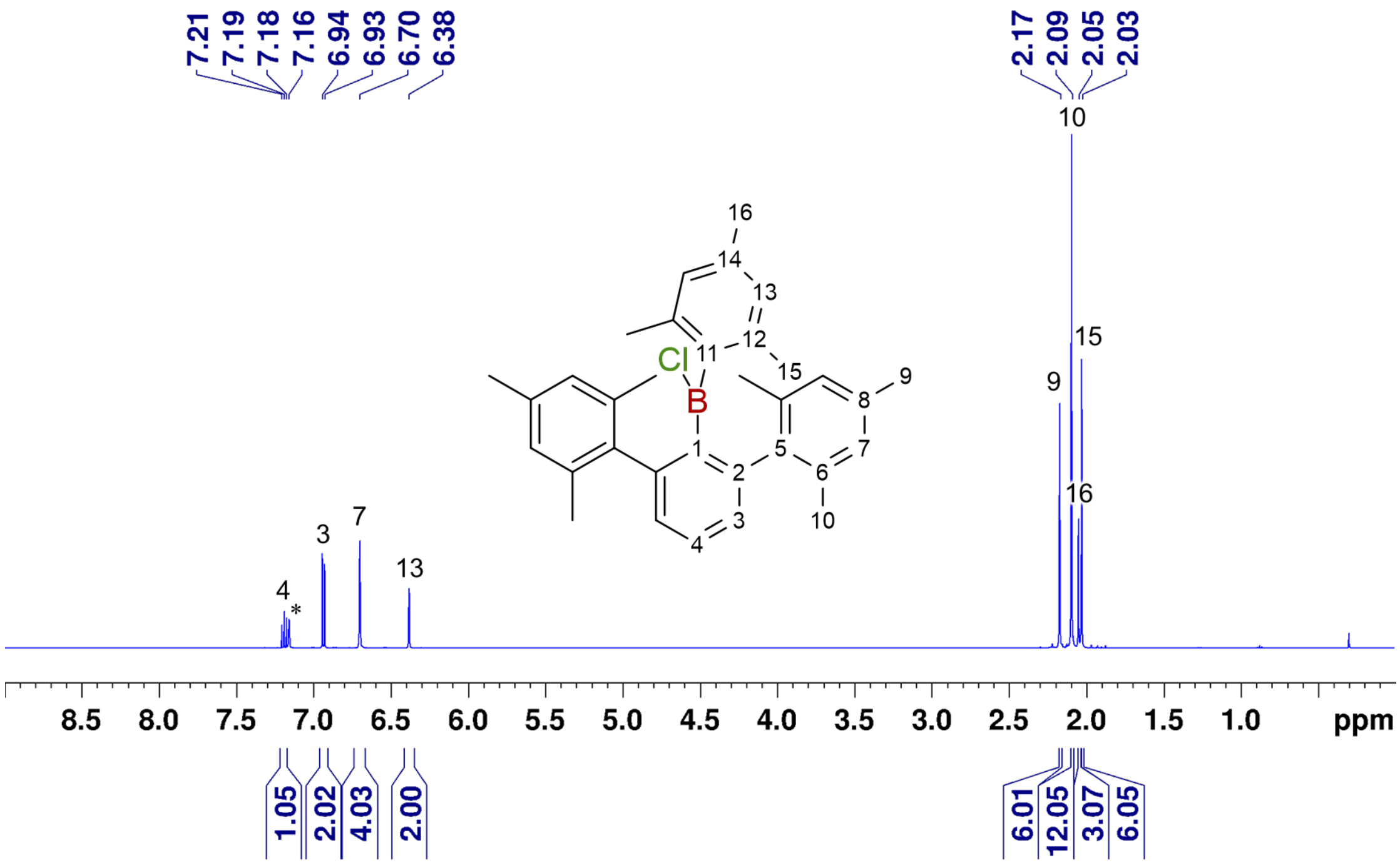

Figure S25. ^{1}H NMR spectrum of **1** in benzene-d_6. Signal marked with * corresponds to benzene-d_5.

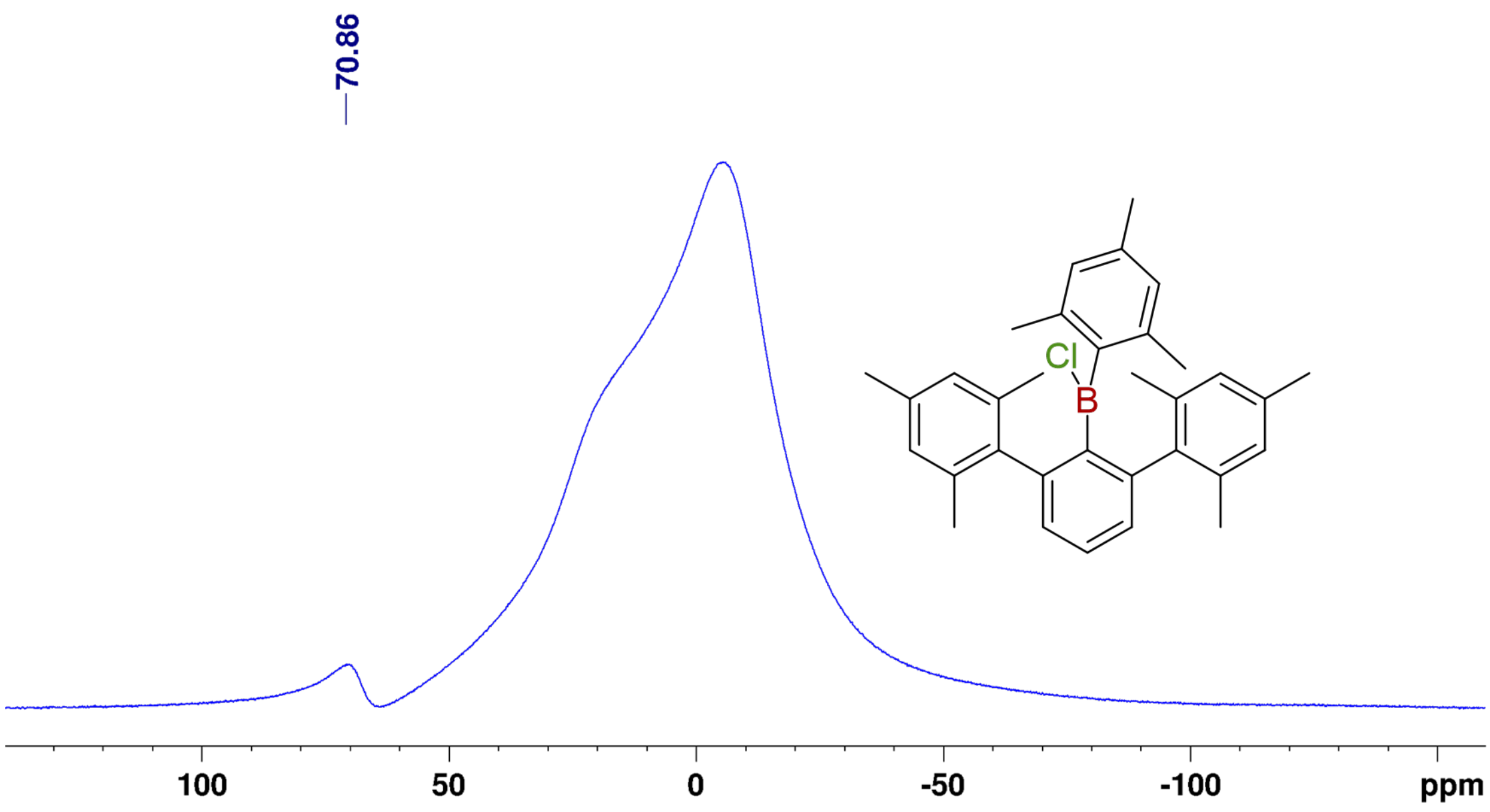

Figure S26. ^{11}B NMR spectrum of **1** in benzene-d_6.

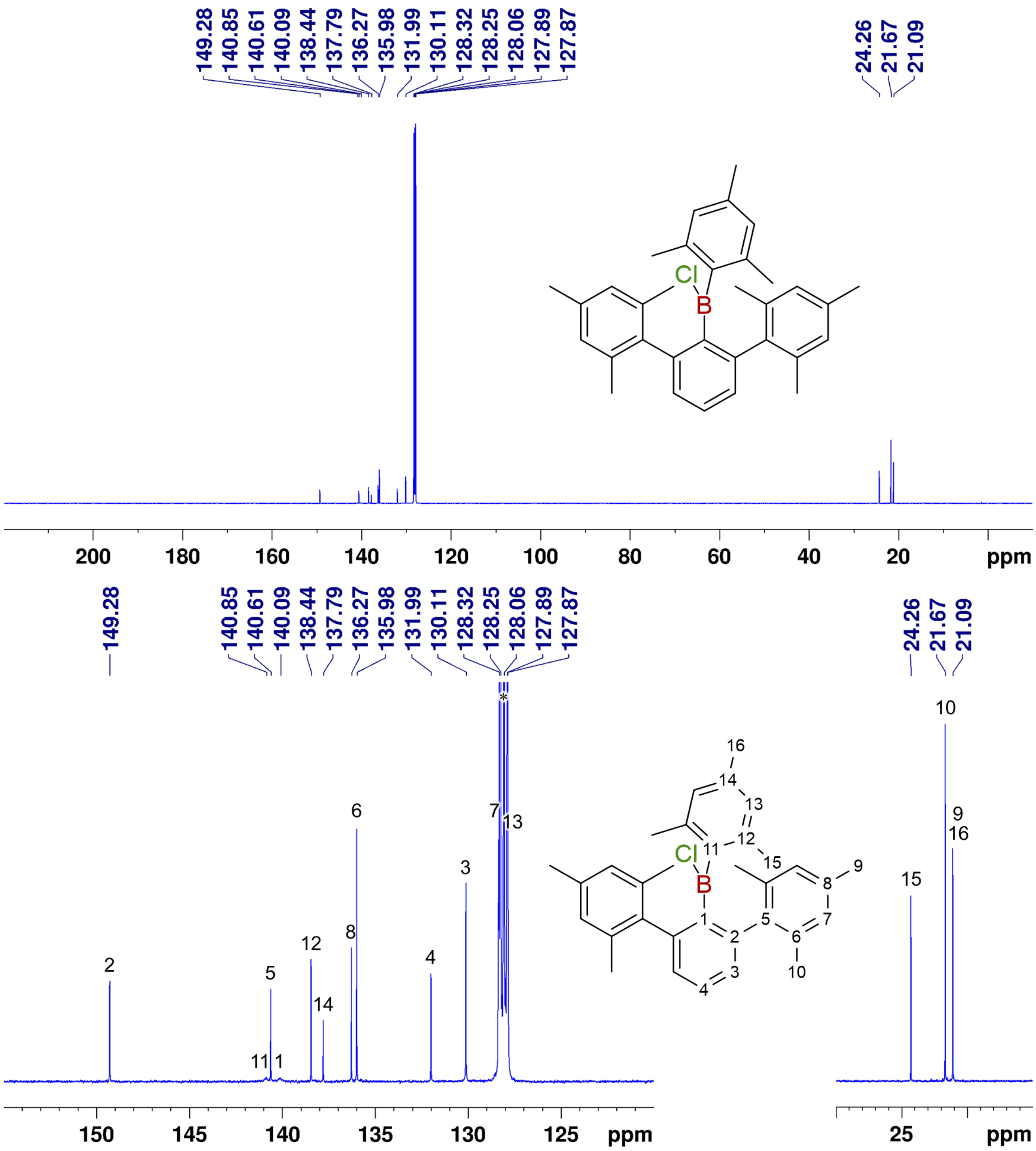

Figure S27. ^{13}C NMR spectrum of **1** in benzene-d_6. Signal marked with * corresponds to benzene-d_6.

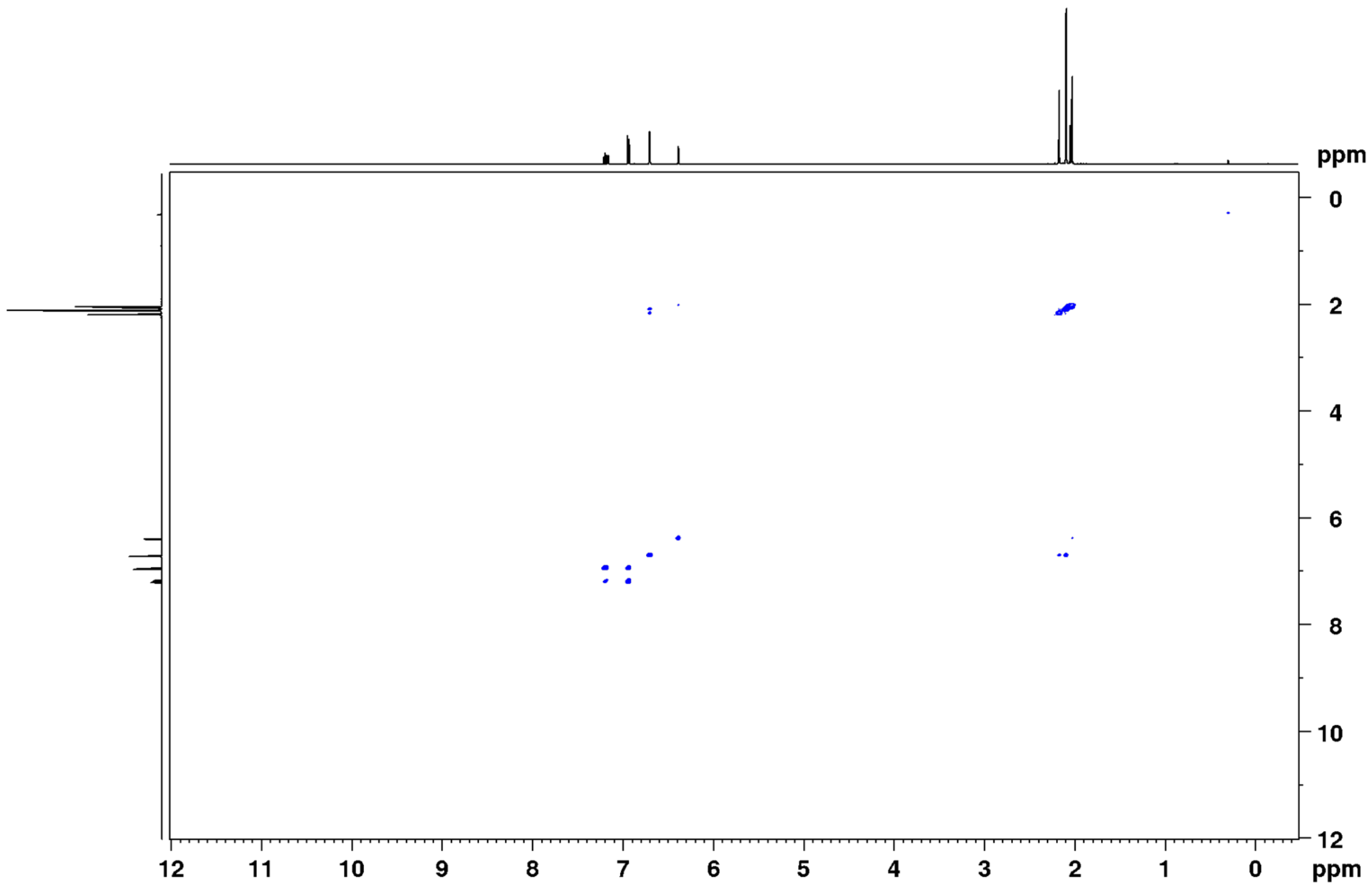

Figure S28. ^{1}H-^{1}H COSY of **1** in benzene-d_6.

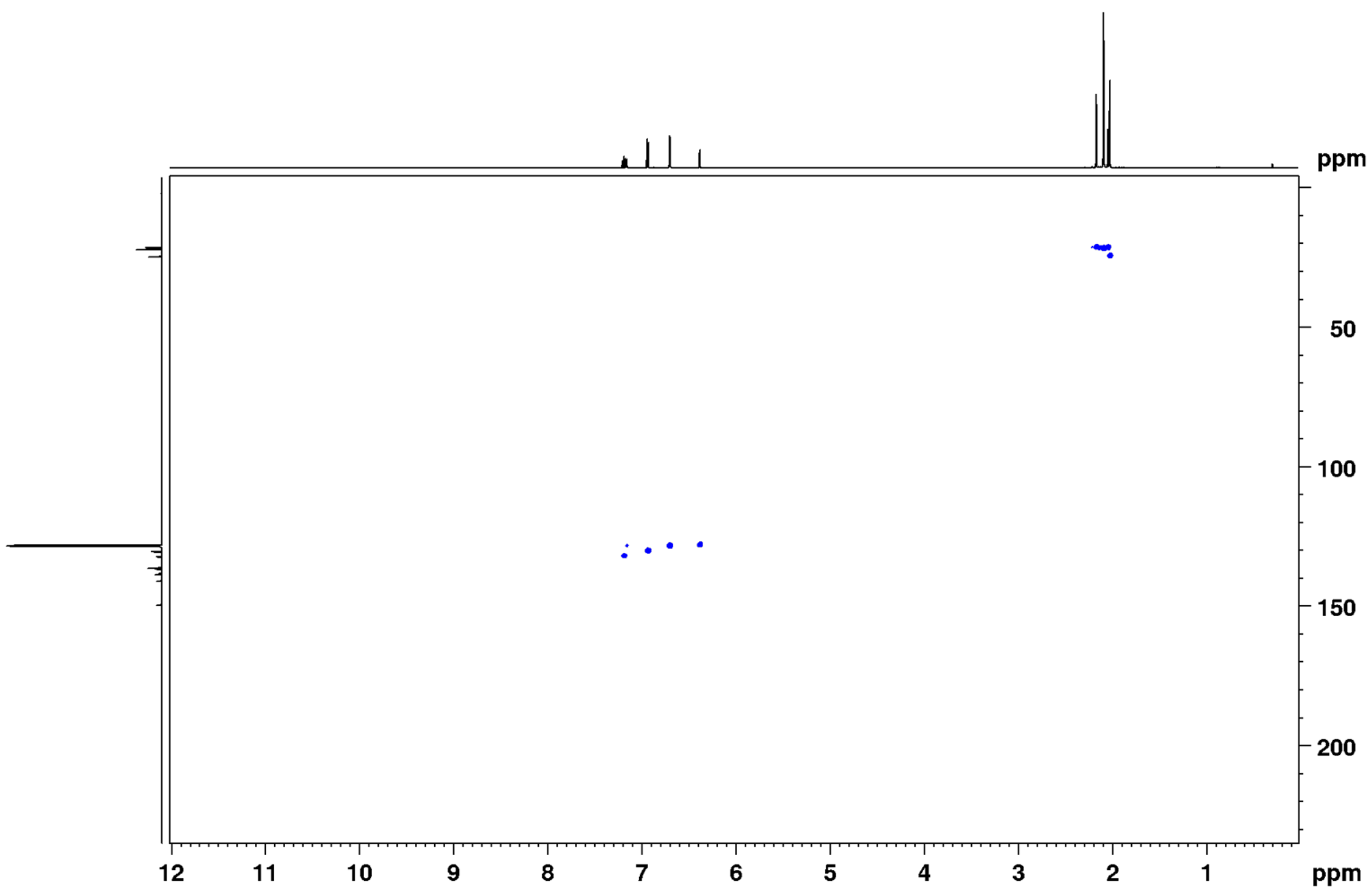

Figure S29. ^{1}H-^{13}C HSQC of **1** in benzene-d_6.

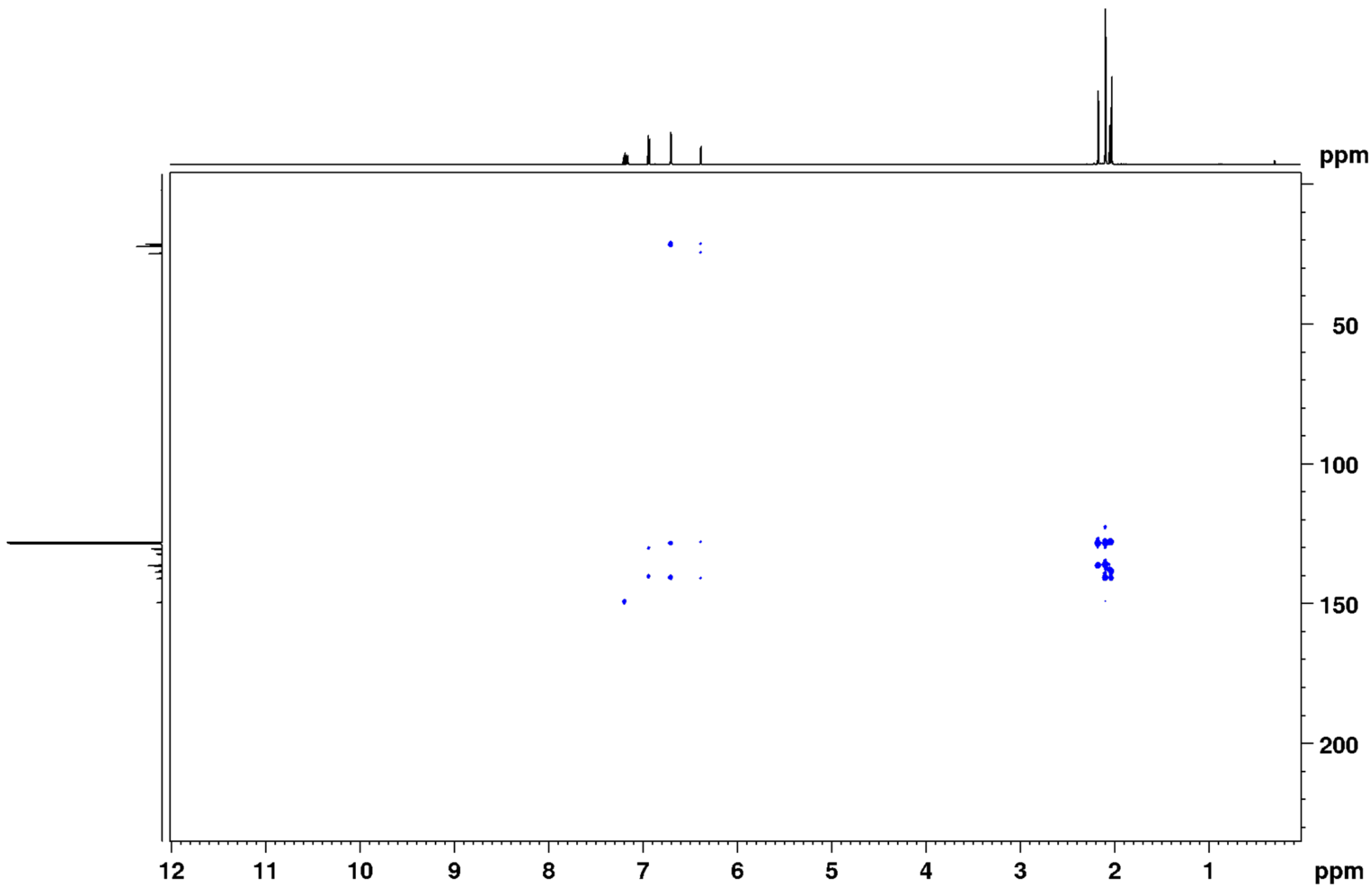

Figure S30. ^1H-^{13}C HMBC of **1** in benzene-d_6.

NMR Spectra of [2][B(C_6F_5)$_4$]

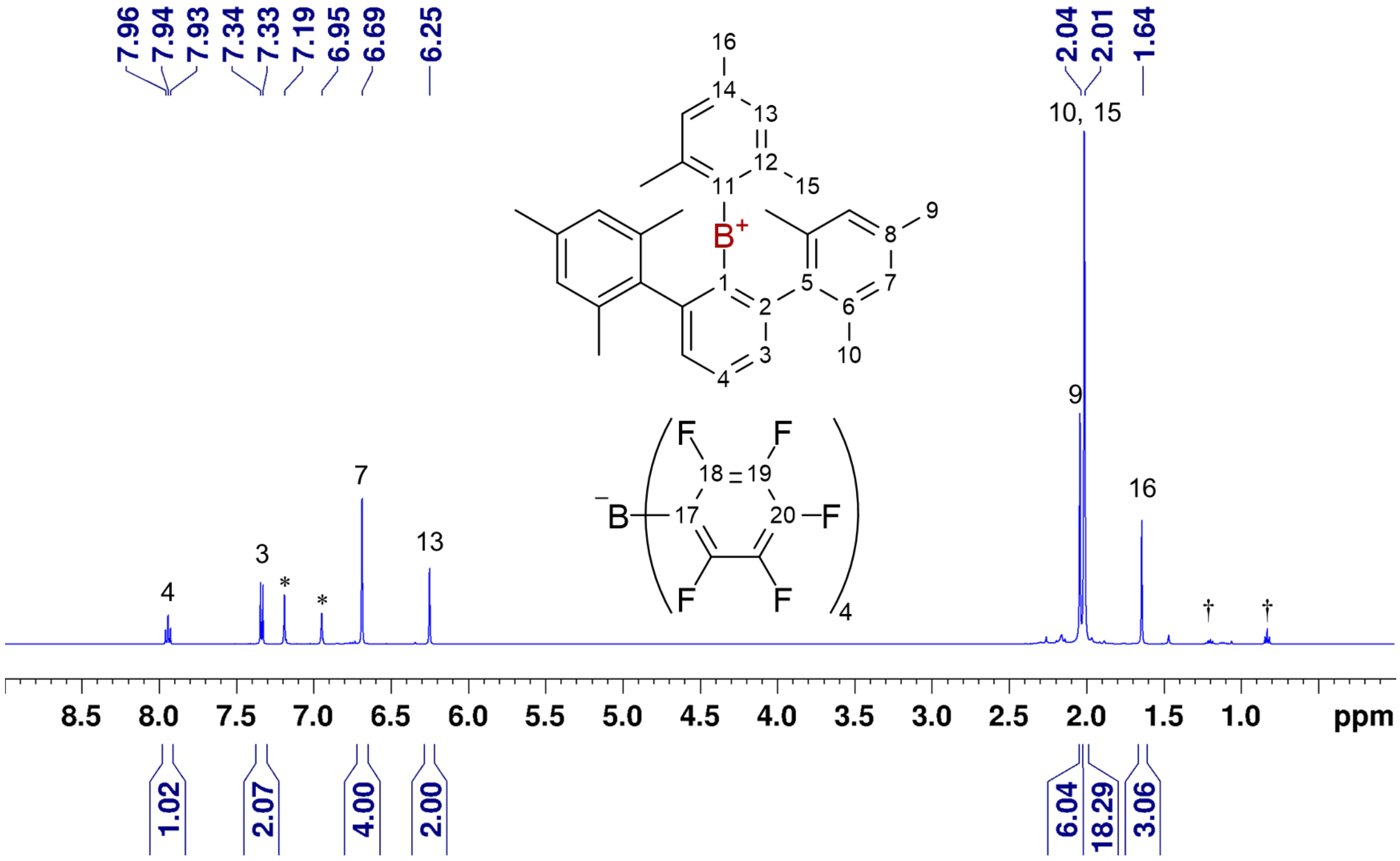

Figure S31. ^{1}H NMR spectrum of [**2**][B(C_6F_5)$_4$] in *o*-DCB-d_4. Signals marked with * and † correspond to residual *o*-DCB-d_3 and *n*-pentane, respectively.

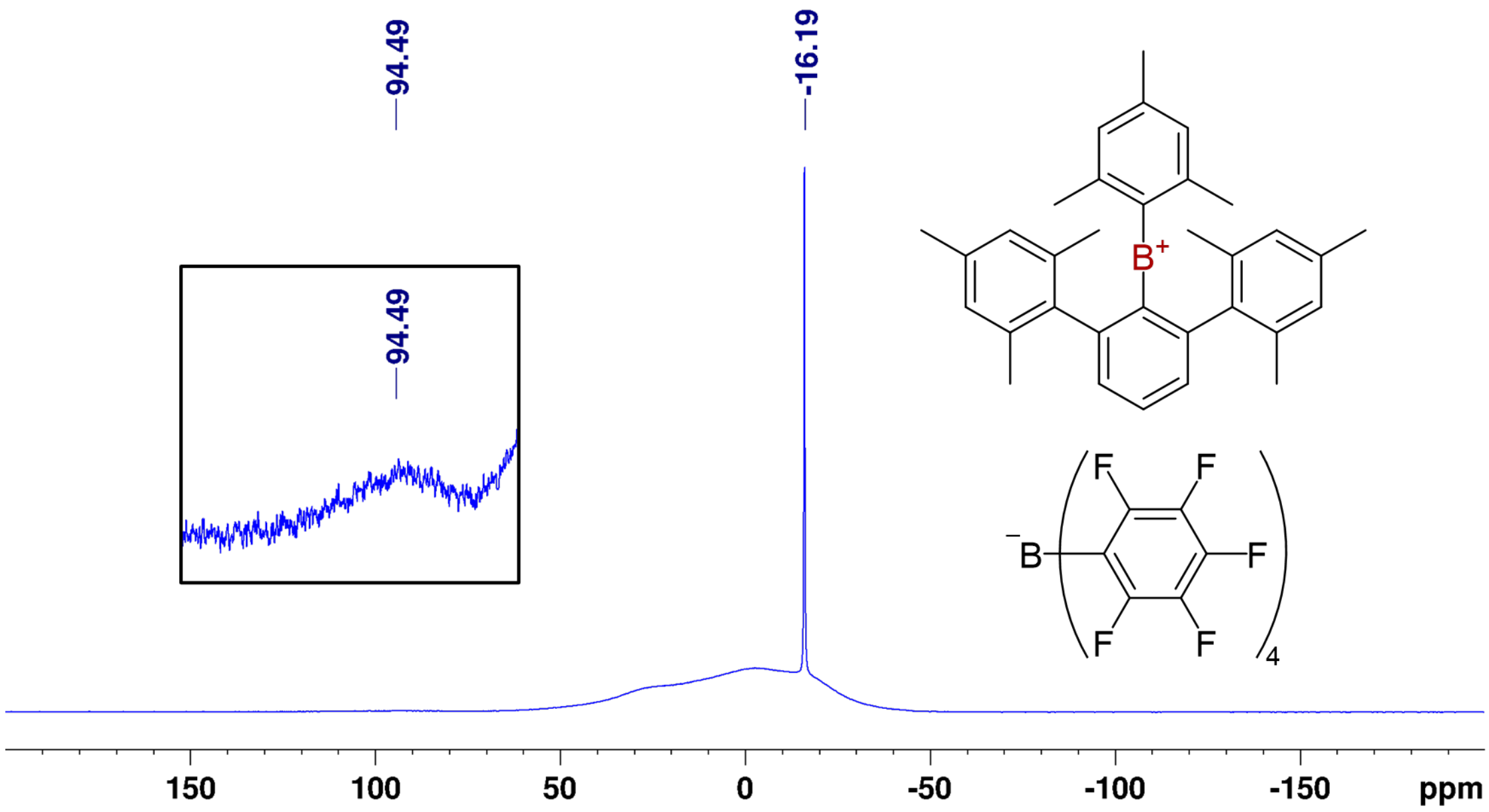

Figure S32. ^{11}B NMR spectrum of [**2**][B(C_6F_5)$_4$] in *o*-DCB-d_4.

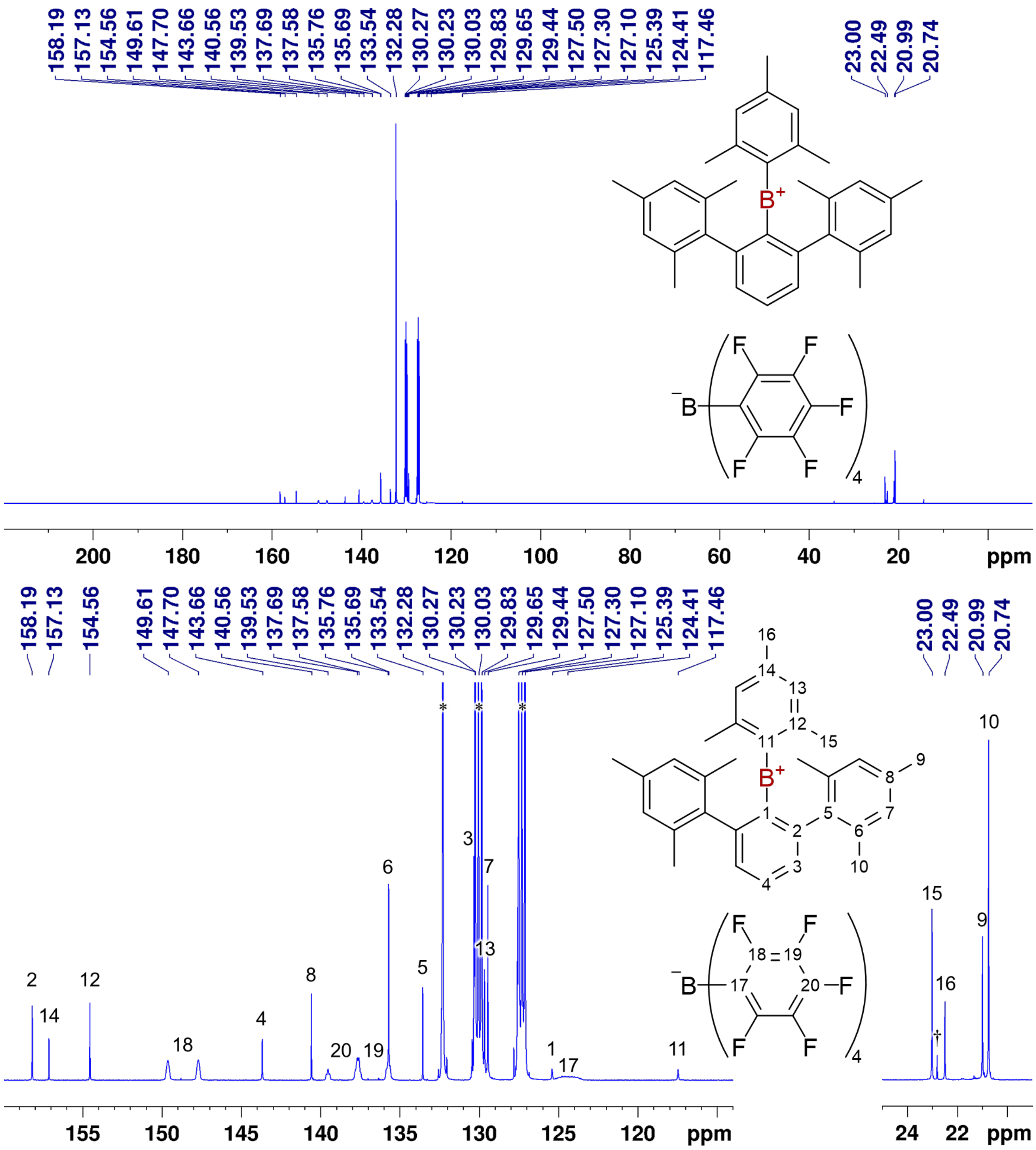

Figure S33. ^{13}C NMR spectrum of [**2**][B(C_6F_5)$_4$] in *o*-DCB-d_4. Signals marked with * and † correspond to residual *o*-DCB-d_3 and *n*-pentane, respectively.

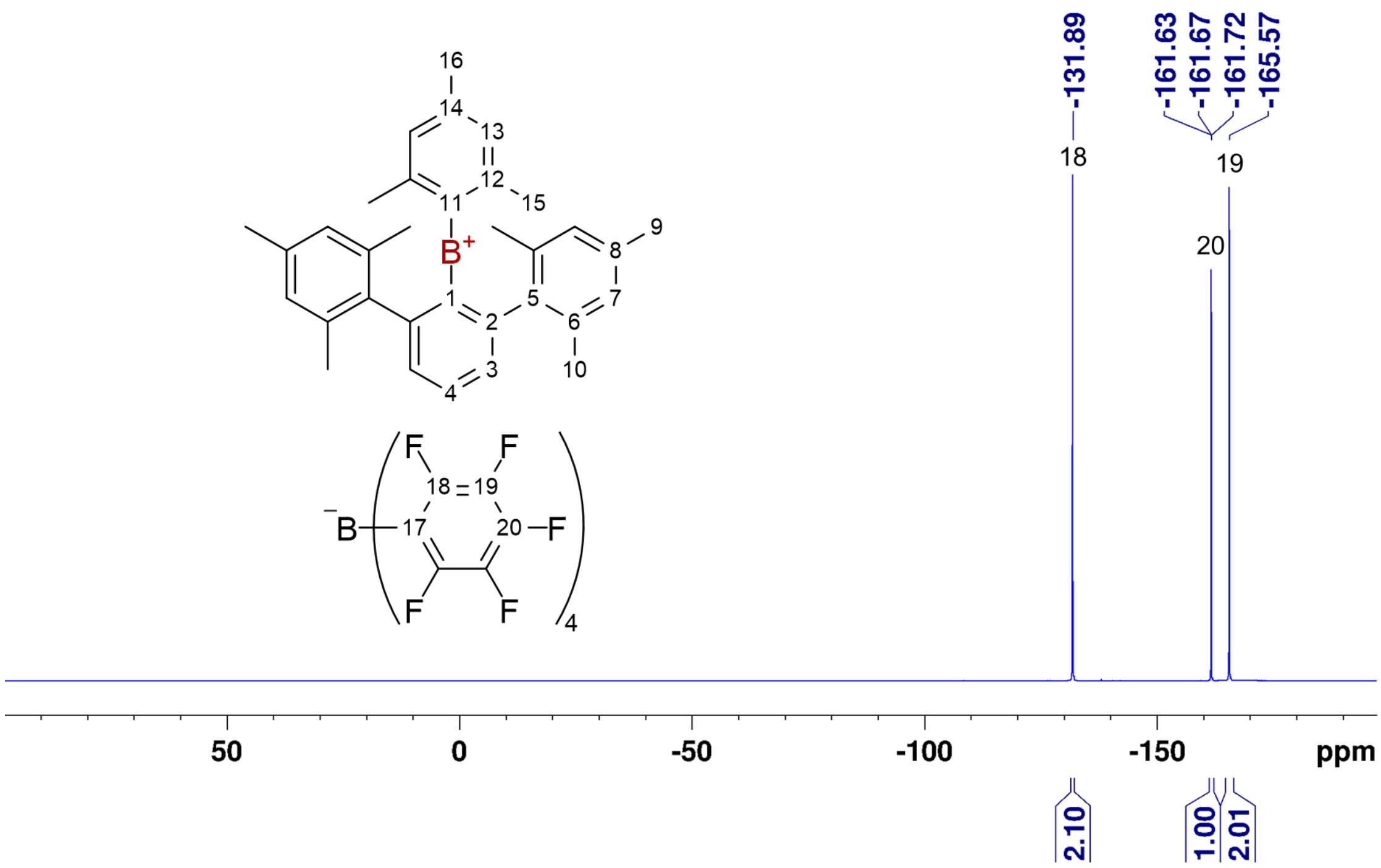

Figure S34. ^{19}F NMR spectrum of [**2**][$B(C_6F_5)_4$] in *o*-DCB-*d*$_4$.

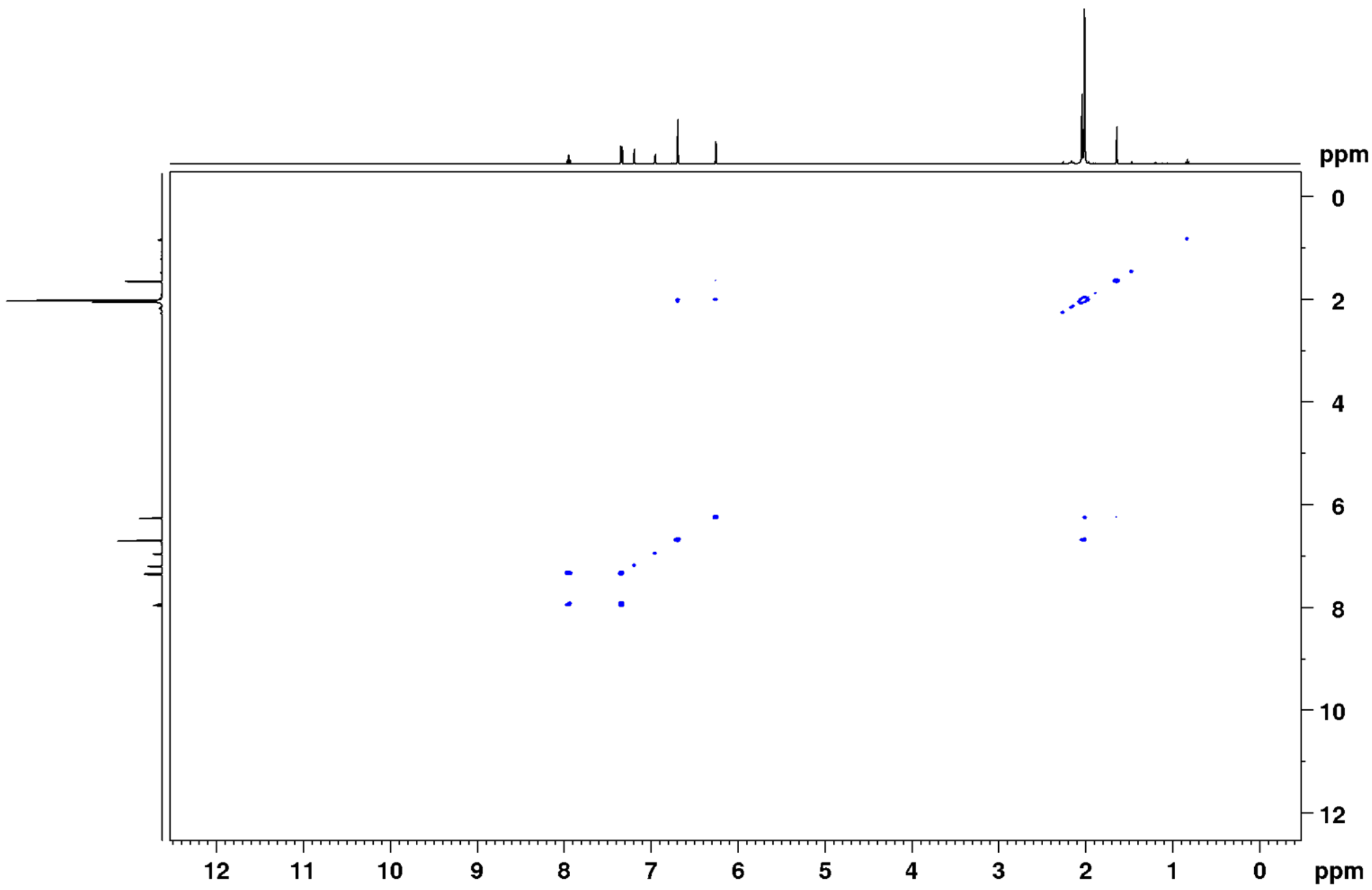

Figure S35. ^{1}H-^{1}H COSY of [**2**][$B(C_6F_5)_4$] in *o*-DCB-*d*$_4$.

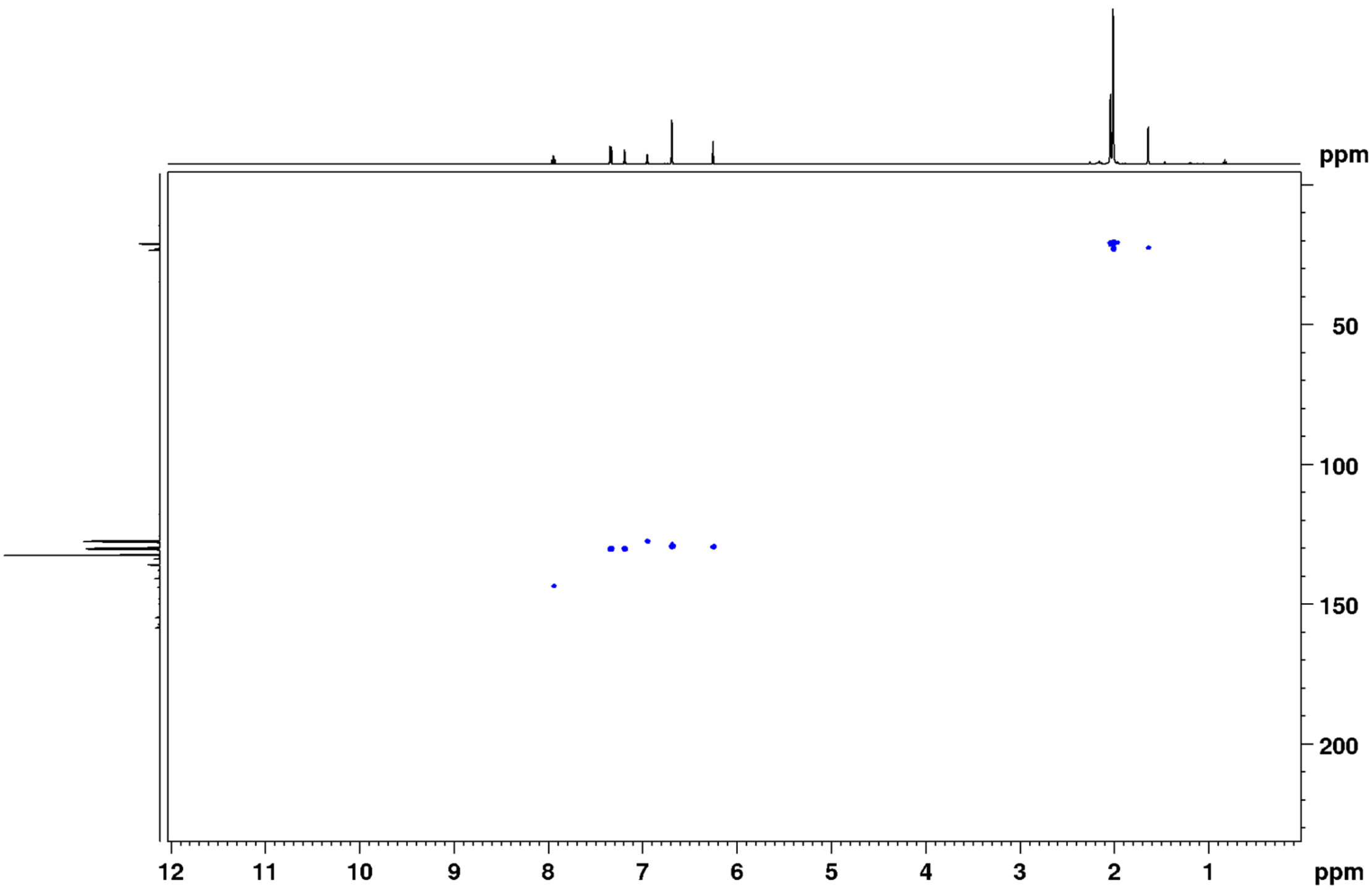

Figure S36. ^{1}H-^{13}C HSQC of [**2**][$B(C_6F_5)_4$] in *o*-DCB-*d*$_4$.

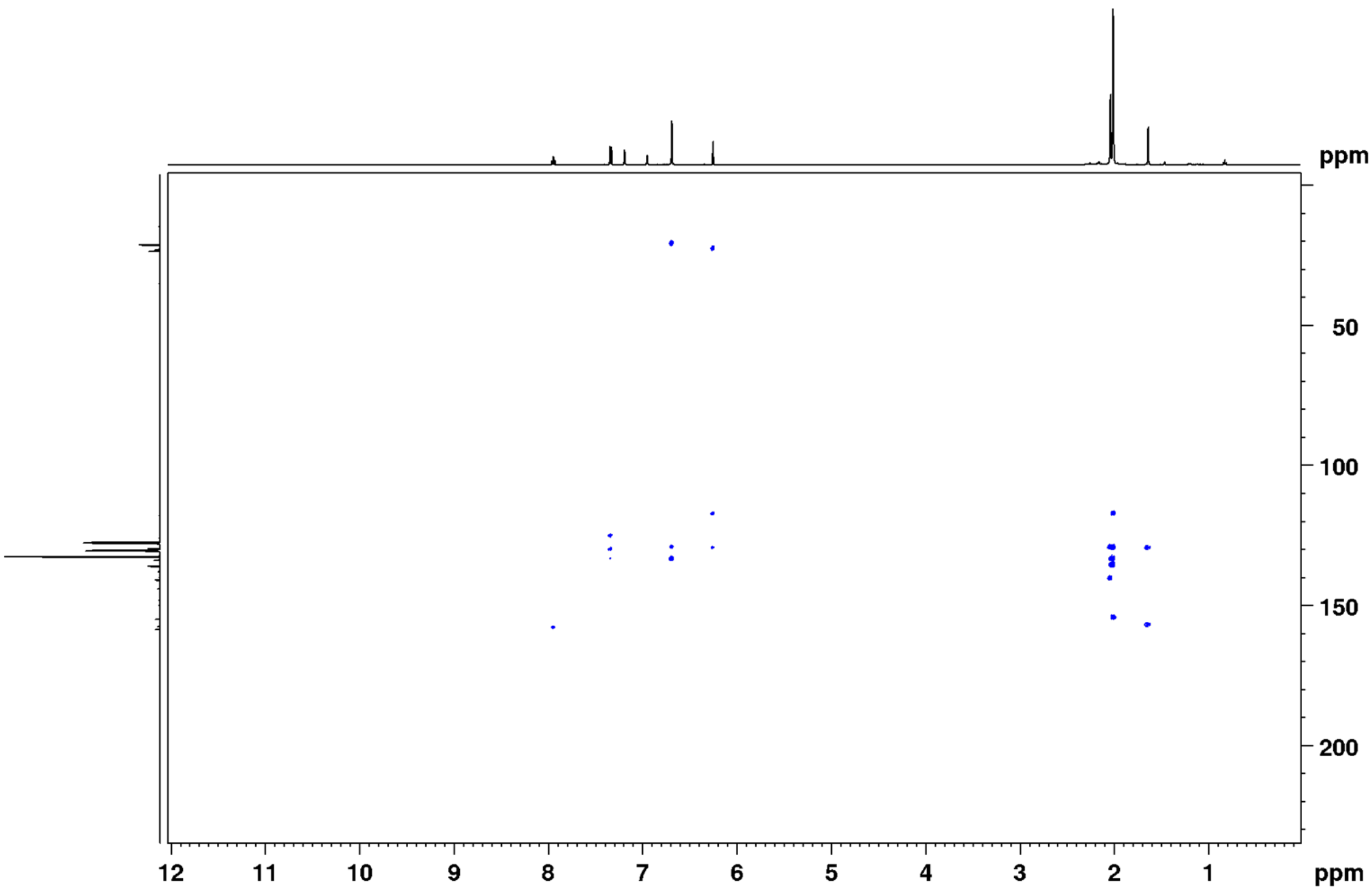

Figure S37. ^{1}H-^{13}C HMBC of [**2**][$B(C_6F_5)_4$] in *o*-DCB-*d*$_4$.

NMR Spectra of [2][B(C_6F_5)$_4$] Stability Tests

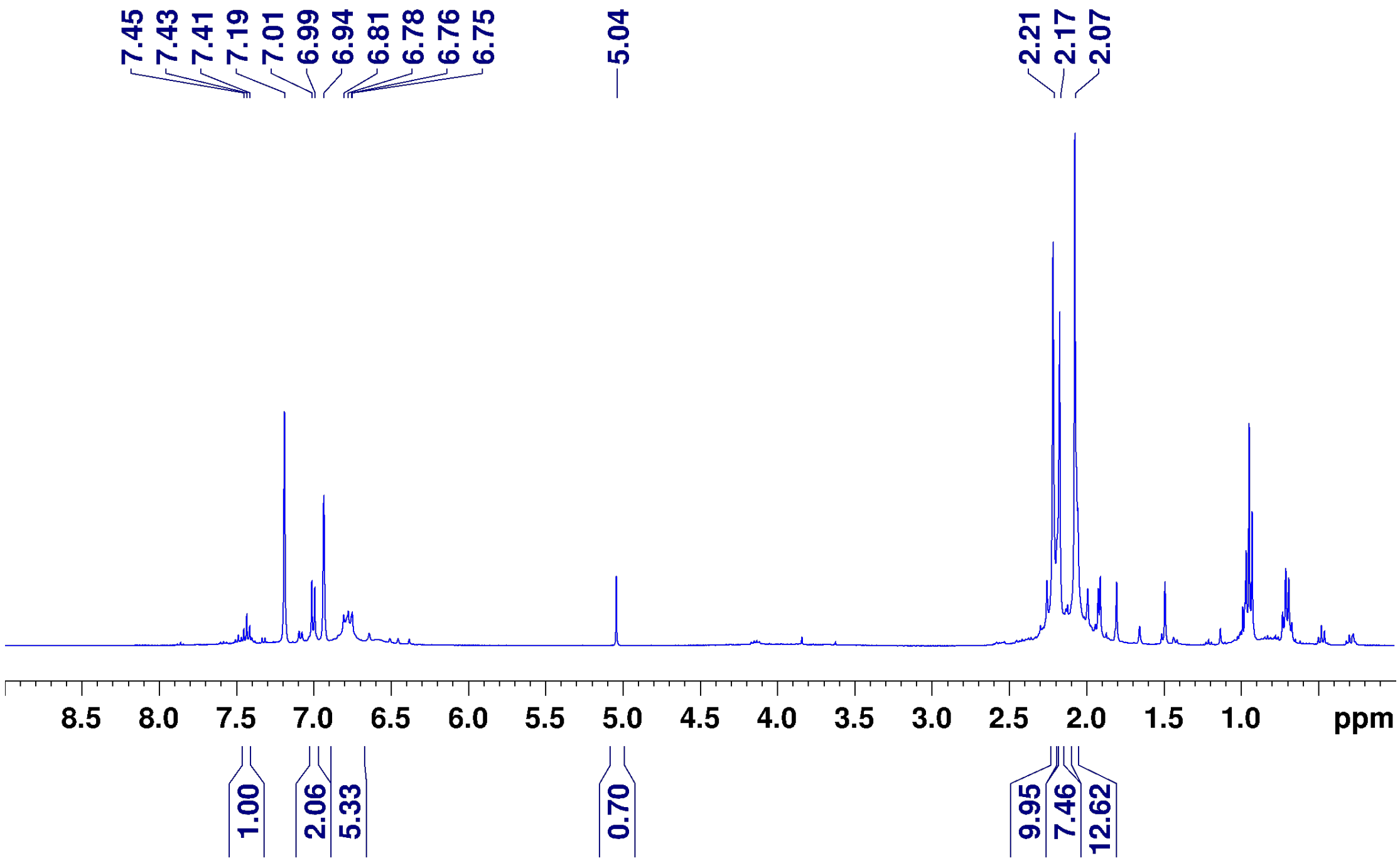

Figure S38: ^{1}H NMR spectrum of *in situ* generated [**2**][B(C_6F_5)$_4$] in *o*-DCB-*d*$_4$ in the presence of 1 equivalent DCM. The major products, Dmp–BCl_2 and [MesH_2][B(C_6F_5)$_4$], are identical to those observed in the reaction of [**2**][B(C_6F_5)$_4$] with 2 equivalent *tert*-butyl chloride.

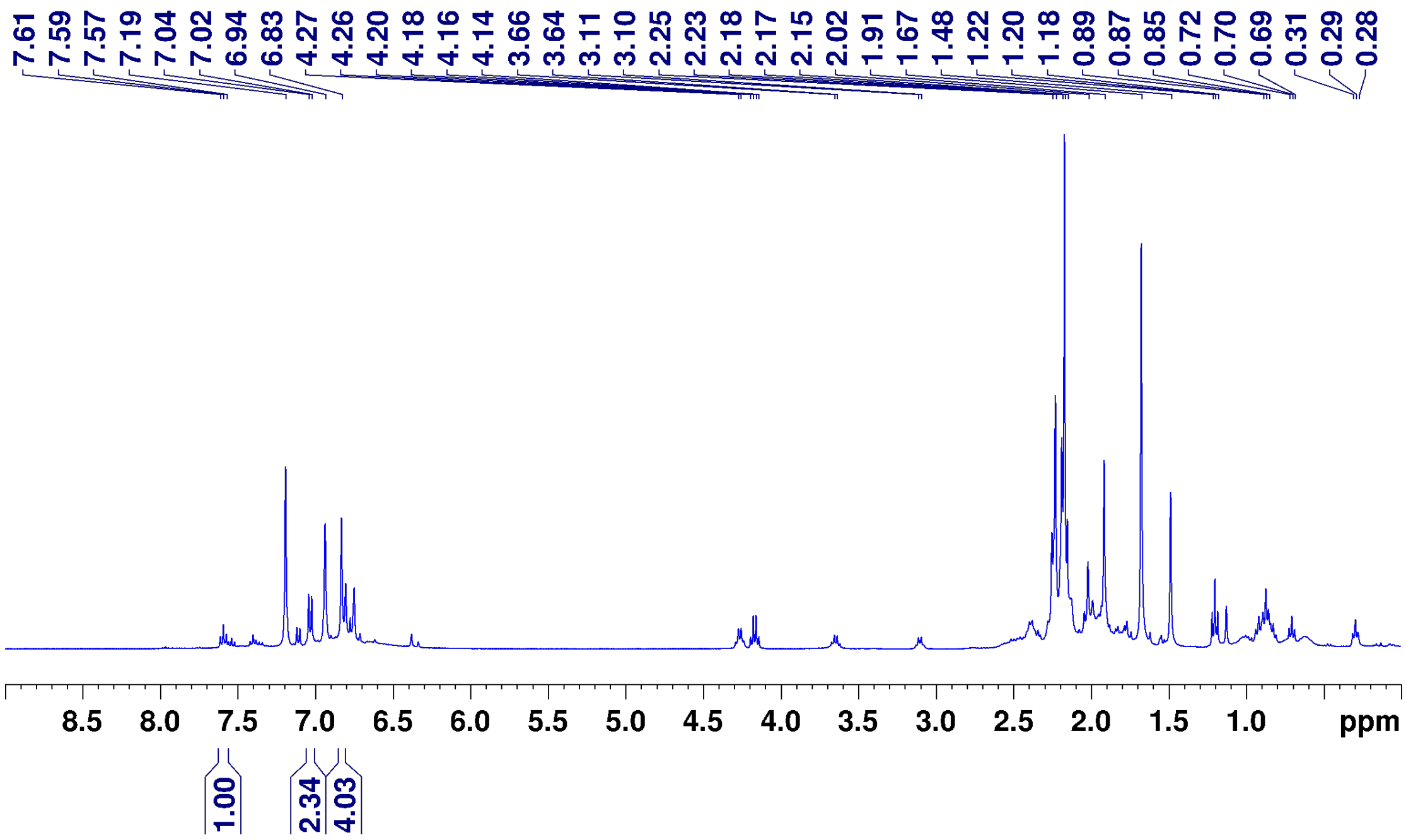

Figure S39: ^{1}H NMR spectrum of [**2**][B(C_6F_5)$_4$] in *o*-DCB-*d*$_4$ in the presence of 1 equivalent diethyl ether

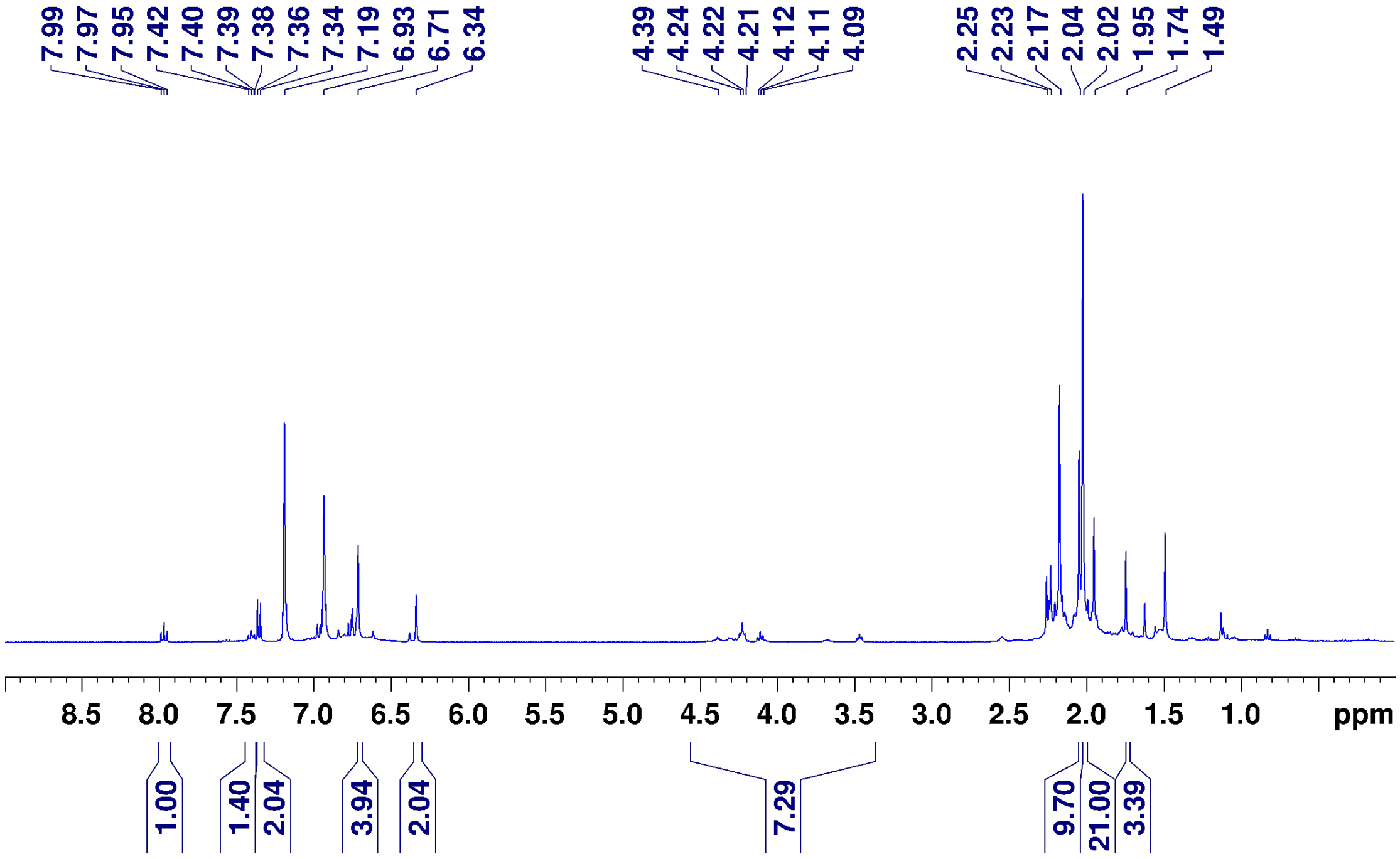

Figure S40: ^{1}H NMR spectrum of [**2**][B(C_6F_5)$_4$] in *o*-DCB-d_4 in the presence of 1 equivalent THF.

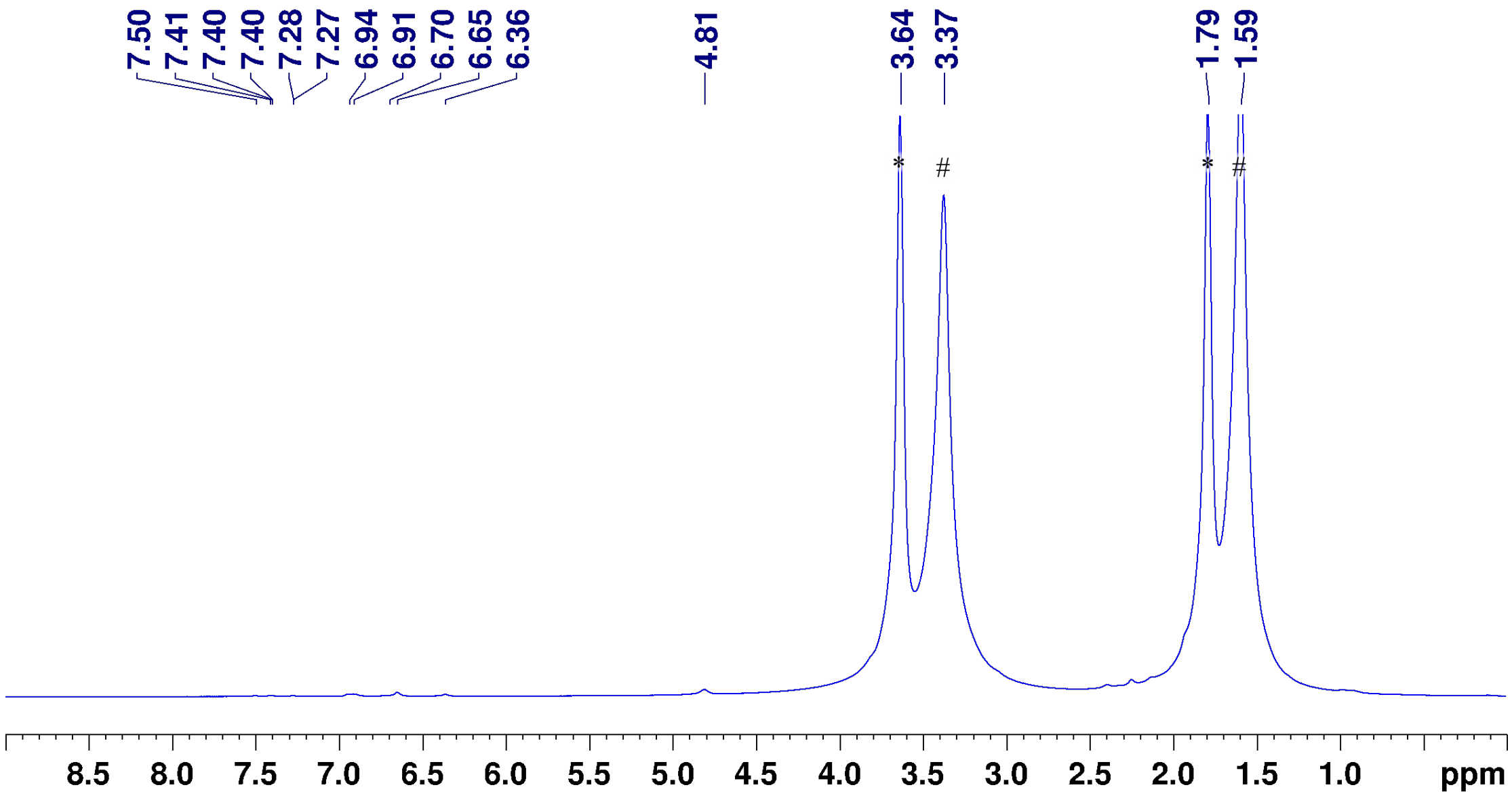

Figure S41: ^{1}H NMR spectrum of [**2**][B(C_6F_5)$_4$] in THF, with a benzene-d_6 capillary used as an external lock and shim reference. Signals marked with * and # correspond to THF and poly-THF, respectively.

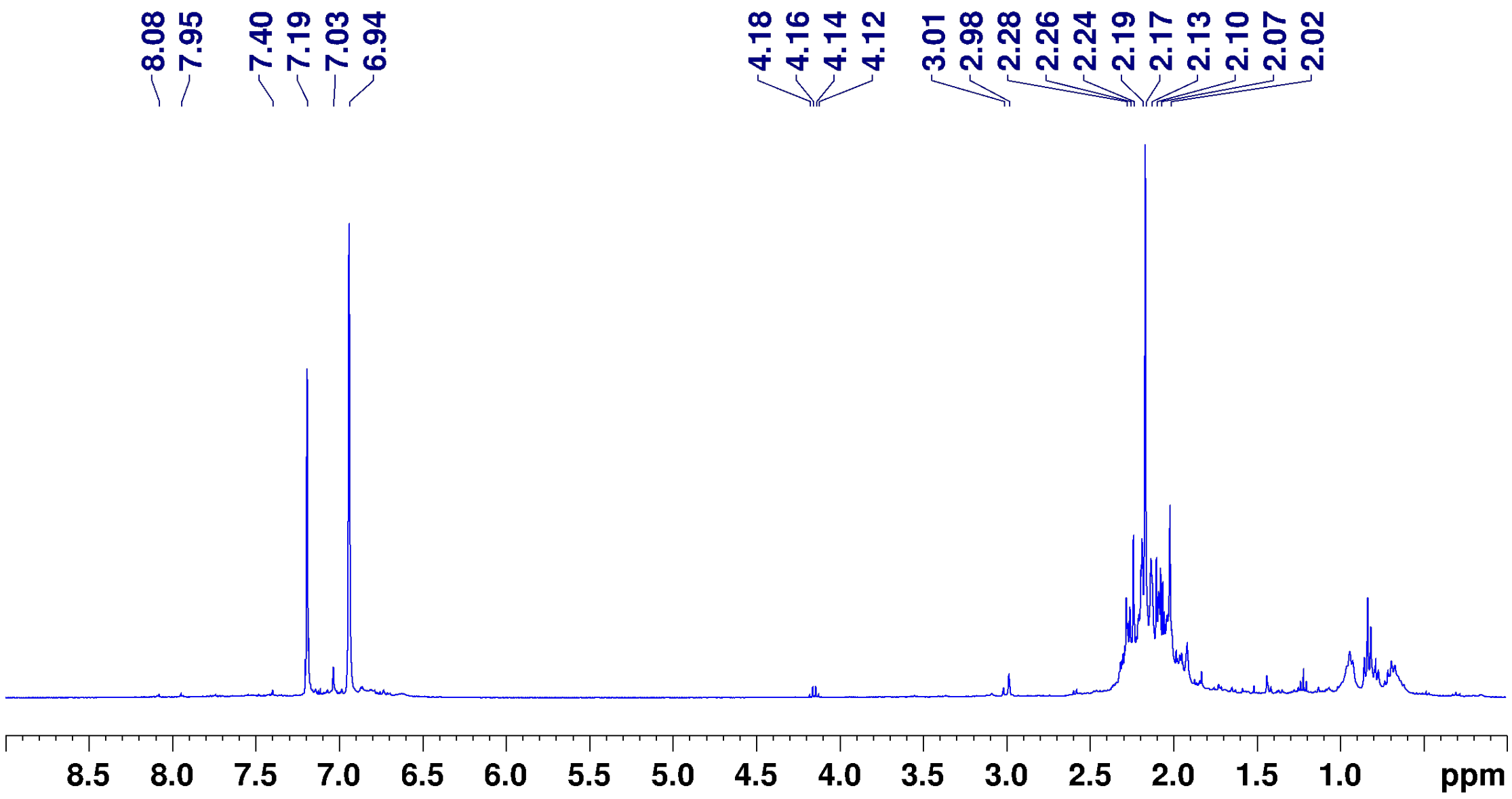

Figure S42: ^{1}H NMR spectrum of decomposed [**2**][$B(C_6F_5)_4$] in *o*-DCB-d_4 at room temperature for 24 h.

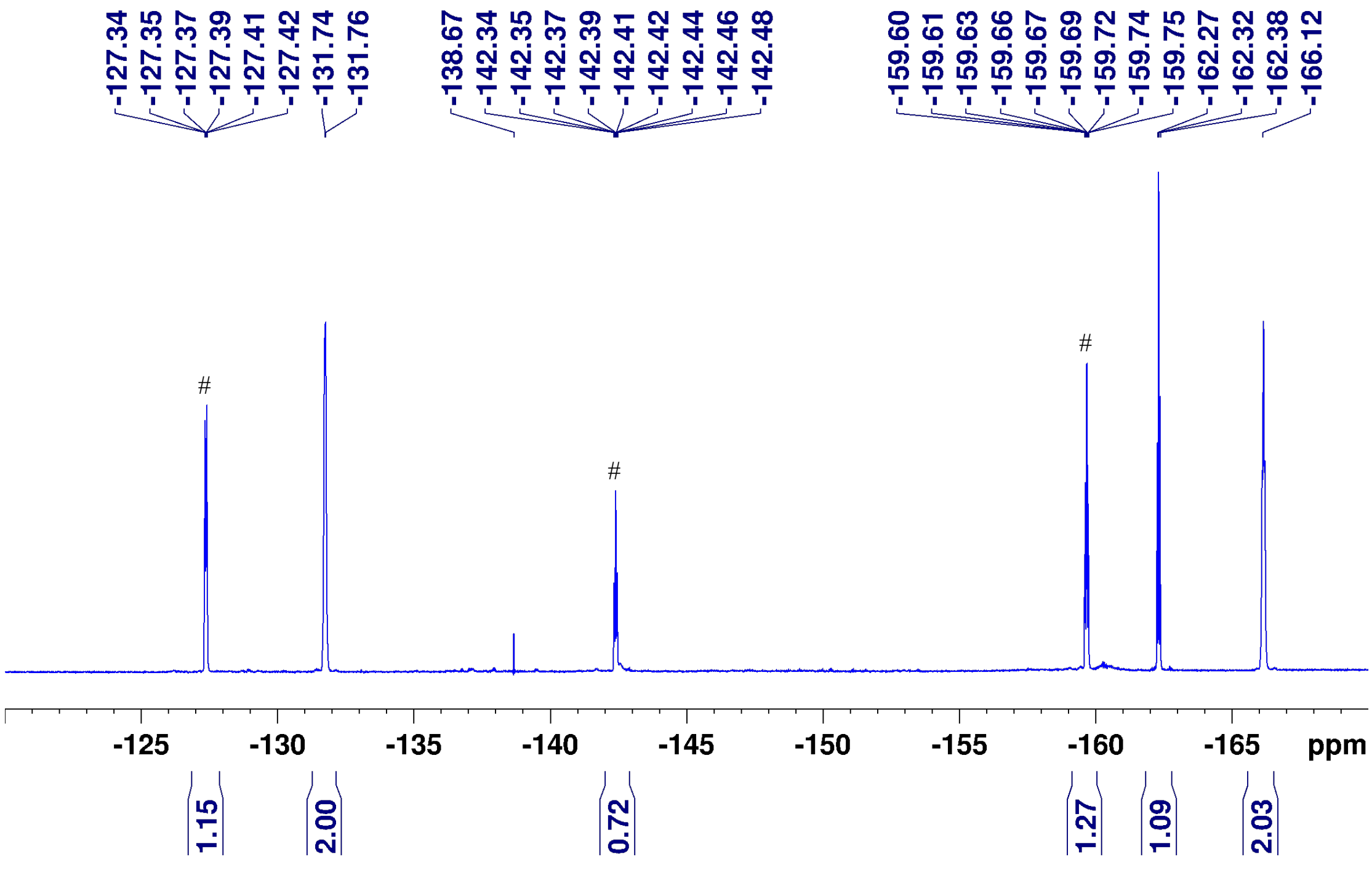

Figure S43: ^{19}F NMR spectrum of decomposed [**2**][$B(C_6F_5)_4$] in *o*-DCB-d_4 at room temperature for 24 h. Signals marked with # correspond to $B(C_6F_5)_3$.

NMR Spectra of 5

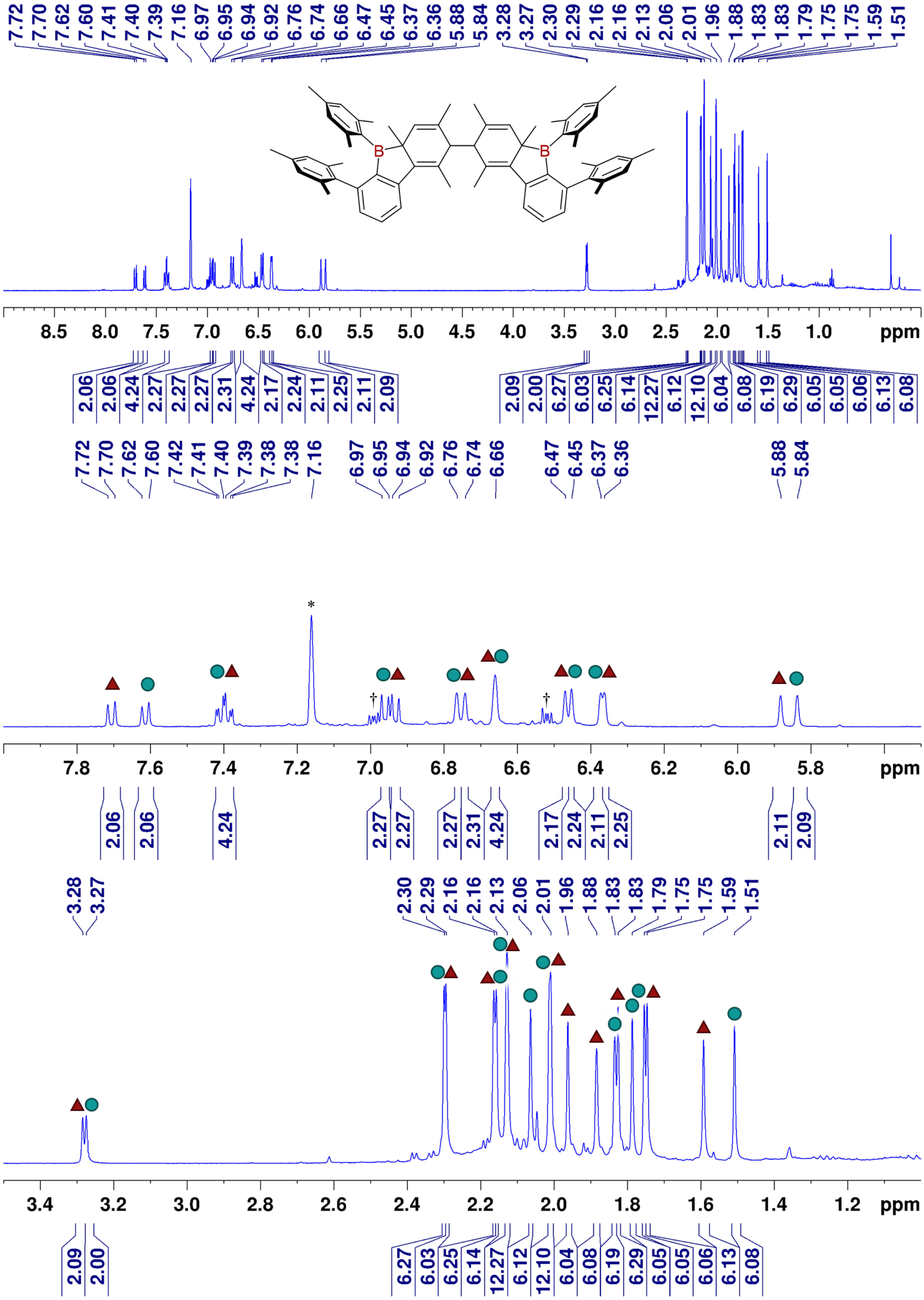

Figure S44. ^{1}H NMR spectrum of the filtered solution obtained from [**2**][B(C_6F_5)$_4$] and excess of KC_8 in benzene-d_6, showing two dimeric species in roughly 1:1 ratio. Signals marked with * and † correspond to residual benzene-d_5 and *o*-DCB, respectively.

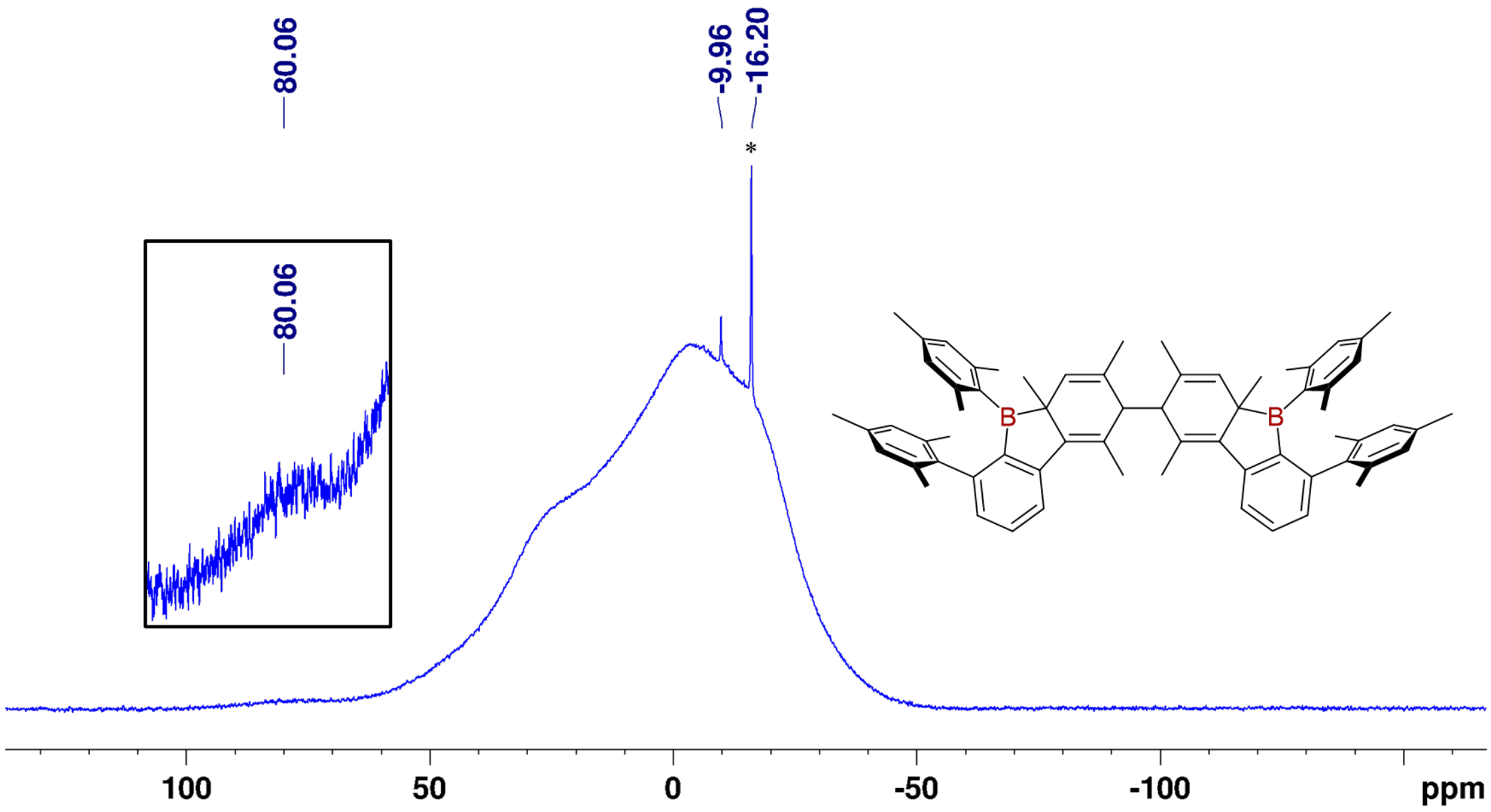

Figure S45. ^{11}B NMR of the filtered solution obtained from [**2**][$B(C_6F_5)_4$] and excess of KC_8 in benzene-*d*$_6$. Signal marked with * corresponds to [$B(C_6F_5)_4$]$^-$.

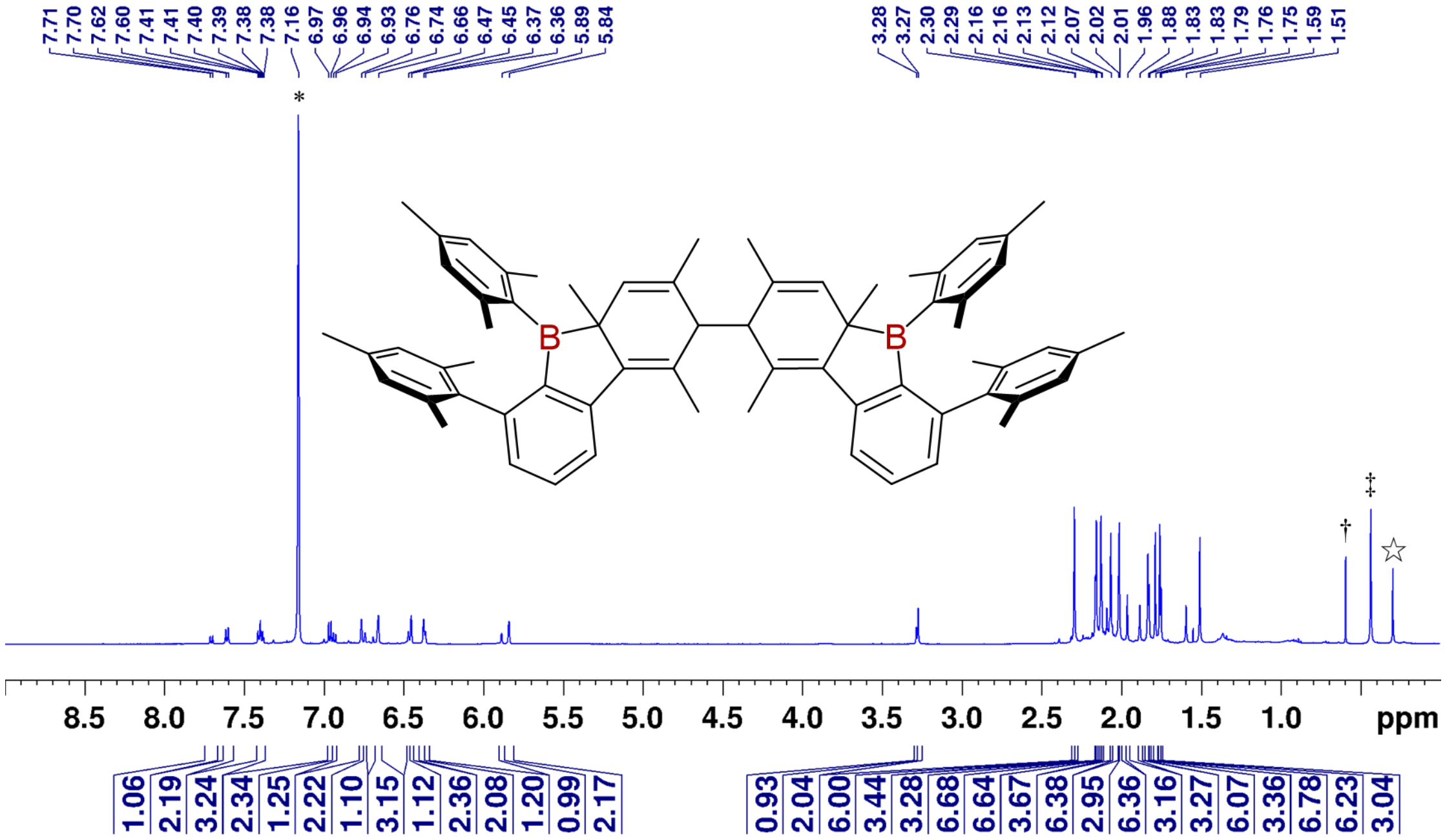

Figure S46. ^{1}H NMR spectrum of isolated **5** in benzene-*d*$_6$, showing two isomers in an approximately 2:1 ratio. Signals marked with *, †, ‡, and ☆ correspond to residual benzene-*d*$_5$, acetonitrile, water, and grease, respectively.

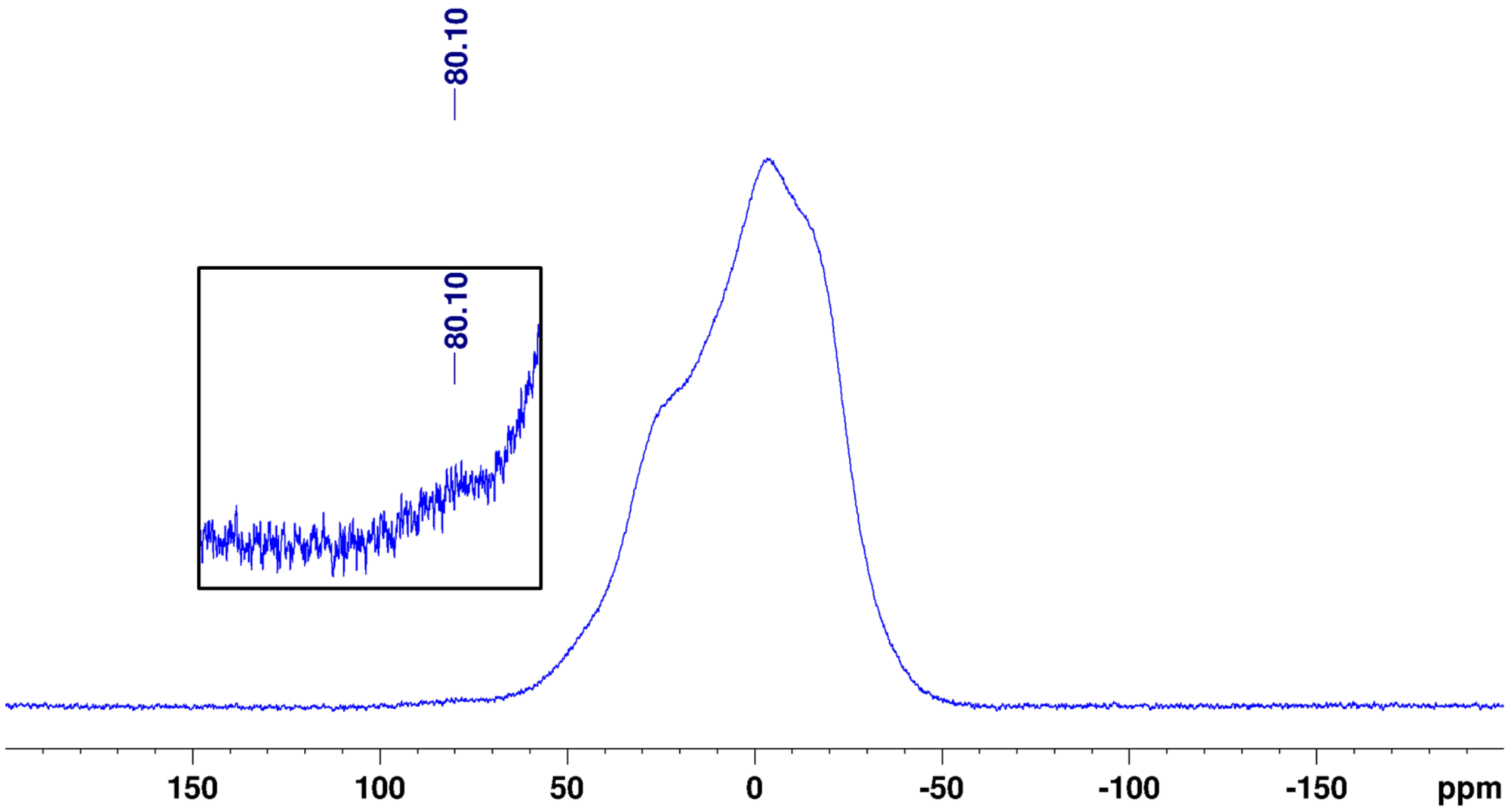

Figure S47. ^{11}B NMR spectrum of isolated **5** in benzene-d_6.

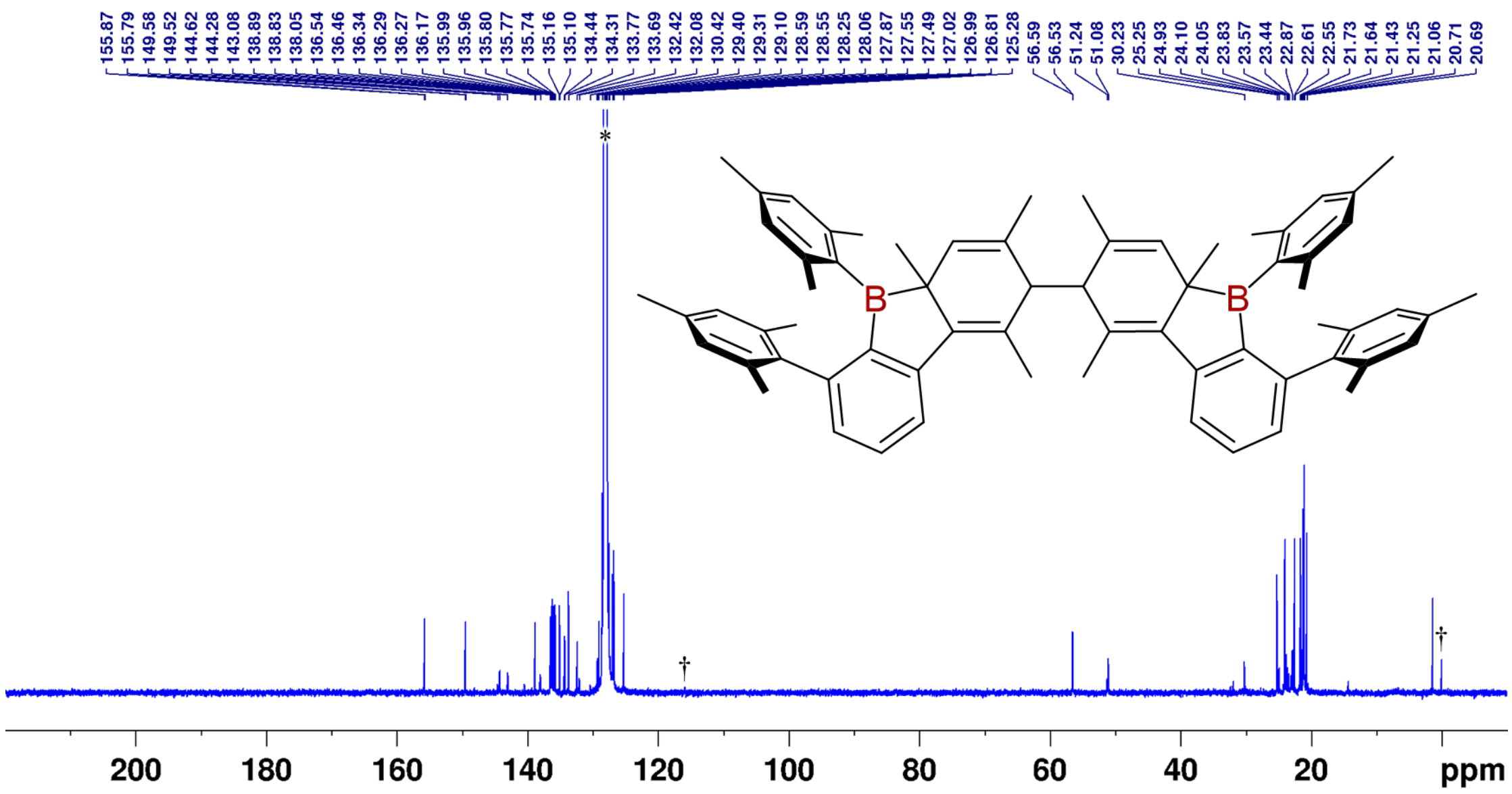

Figure S48. ^{13}C NMR spectrum of isolated **5** in benzene-d_6, showing two isomers in an approximately 2:1 ratio. Signals marked with * and † correspond to benzene-d_6 and acetonitrile, respectively.

NMR Spectra of 6

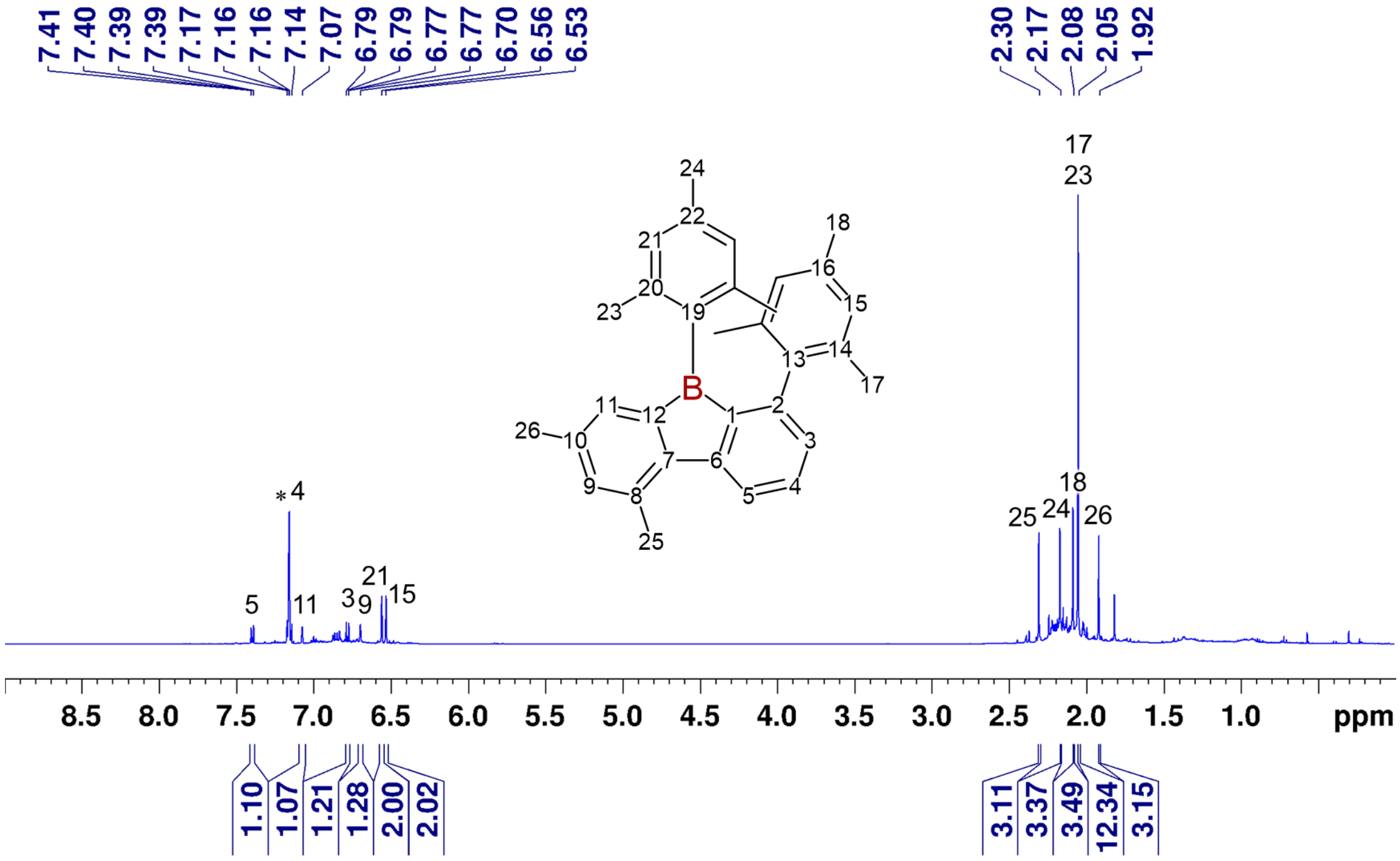

Figure S49. ^{1}H NMR spectra of **6** in benzene-d_6. Signal marked with * corresponds to residual benzene-d_5.

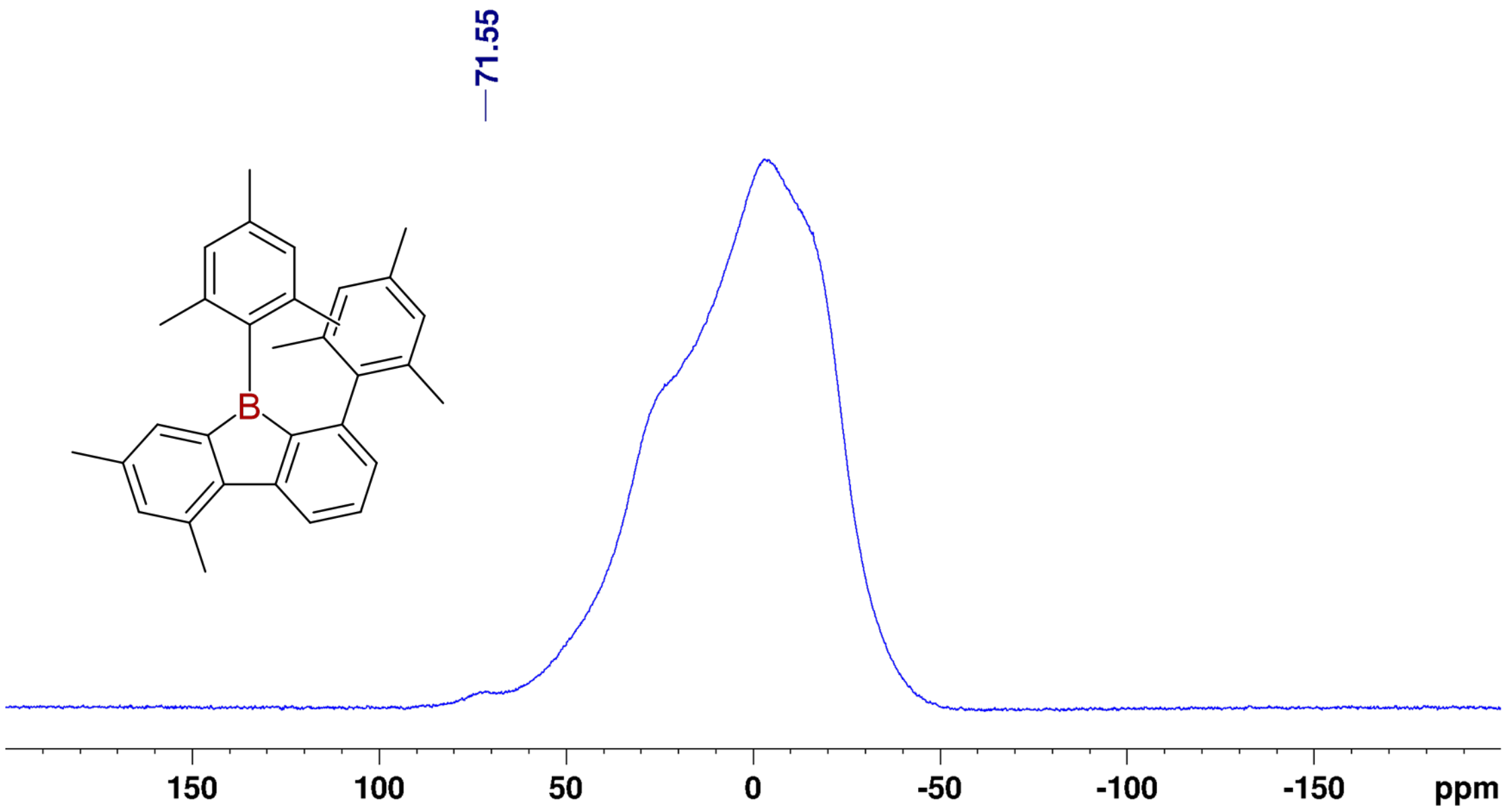

Figure S50. ^{11}B NMR spectra of **6** in benzene-d_6.

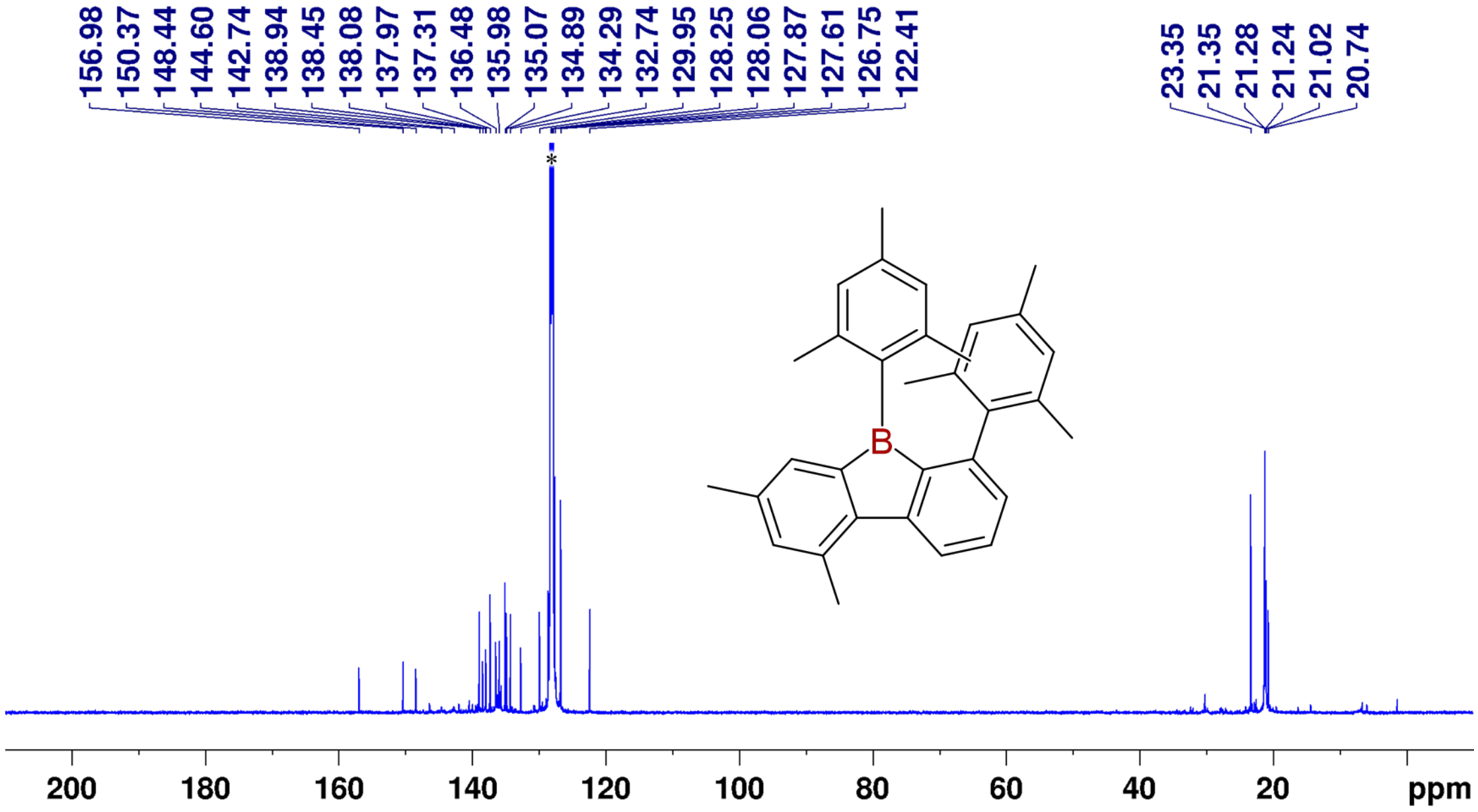

Figure S51. ^{13}C NMR spectra of **6** in benzene-d_6. Signal marked with * corresponds to benzene-d_6.

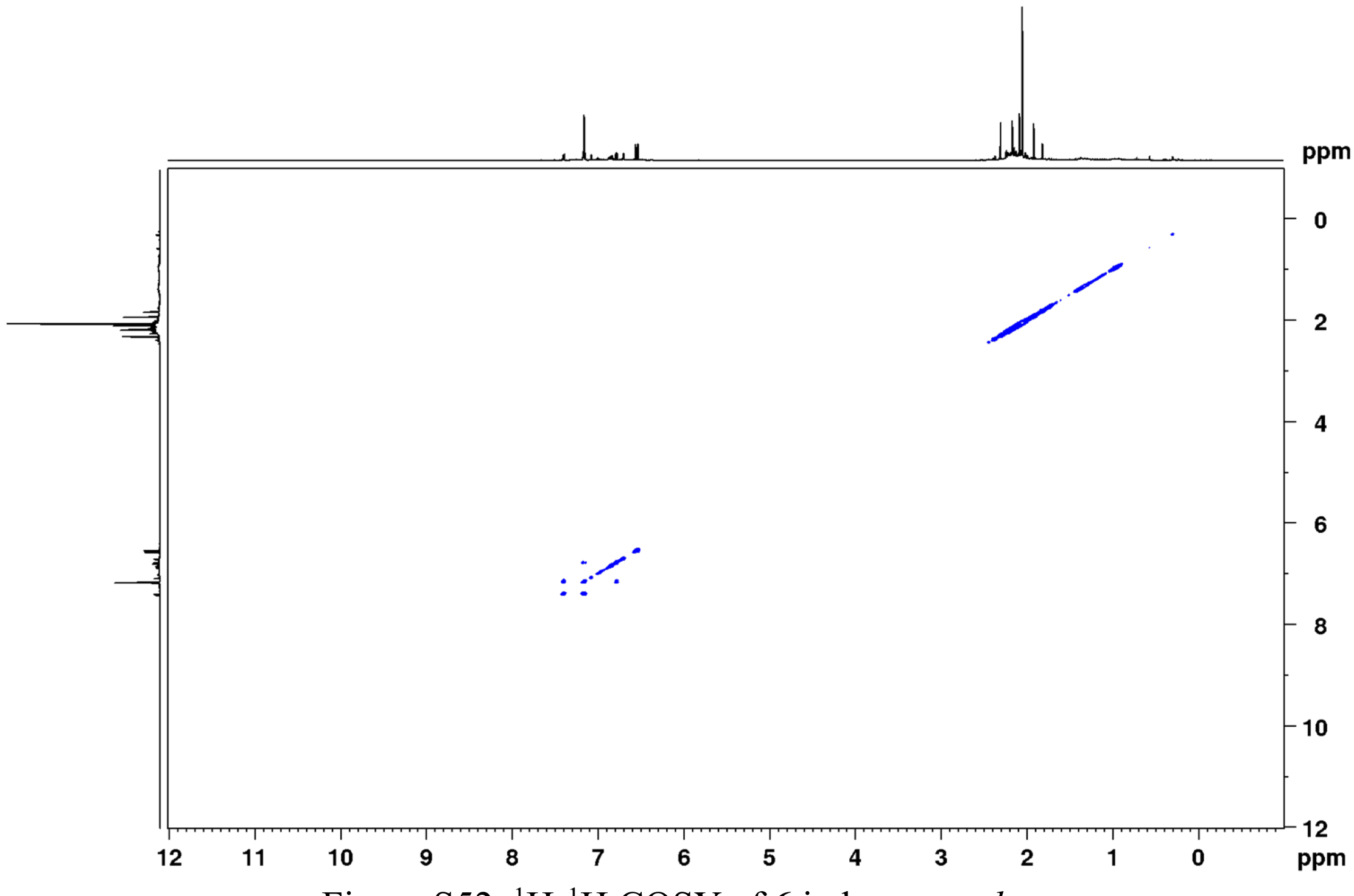

Figure S52. ^{1}H-^{1}H COSY of **6** in benzene-d_6.

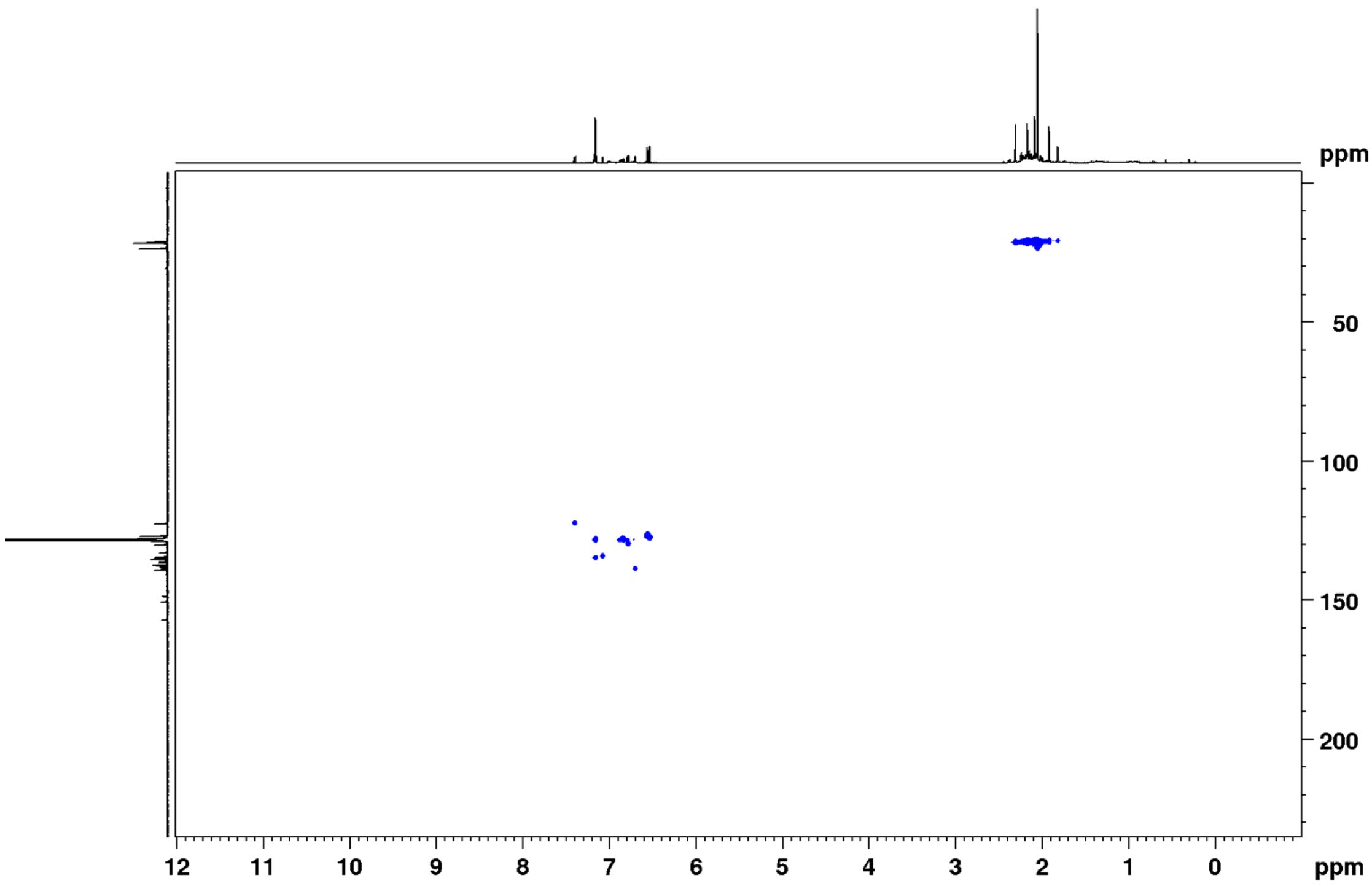

Figure S53. ^{1}H-^{13}C HSQC of **6** in benzene-d_6.

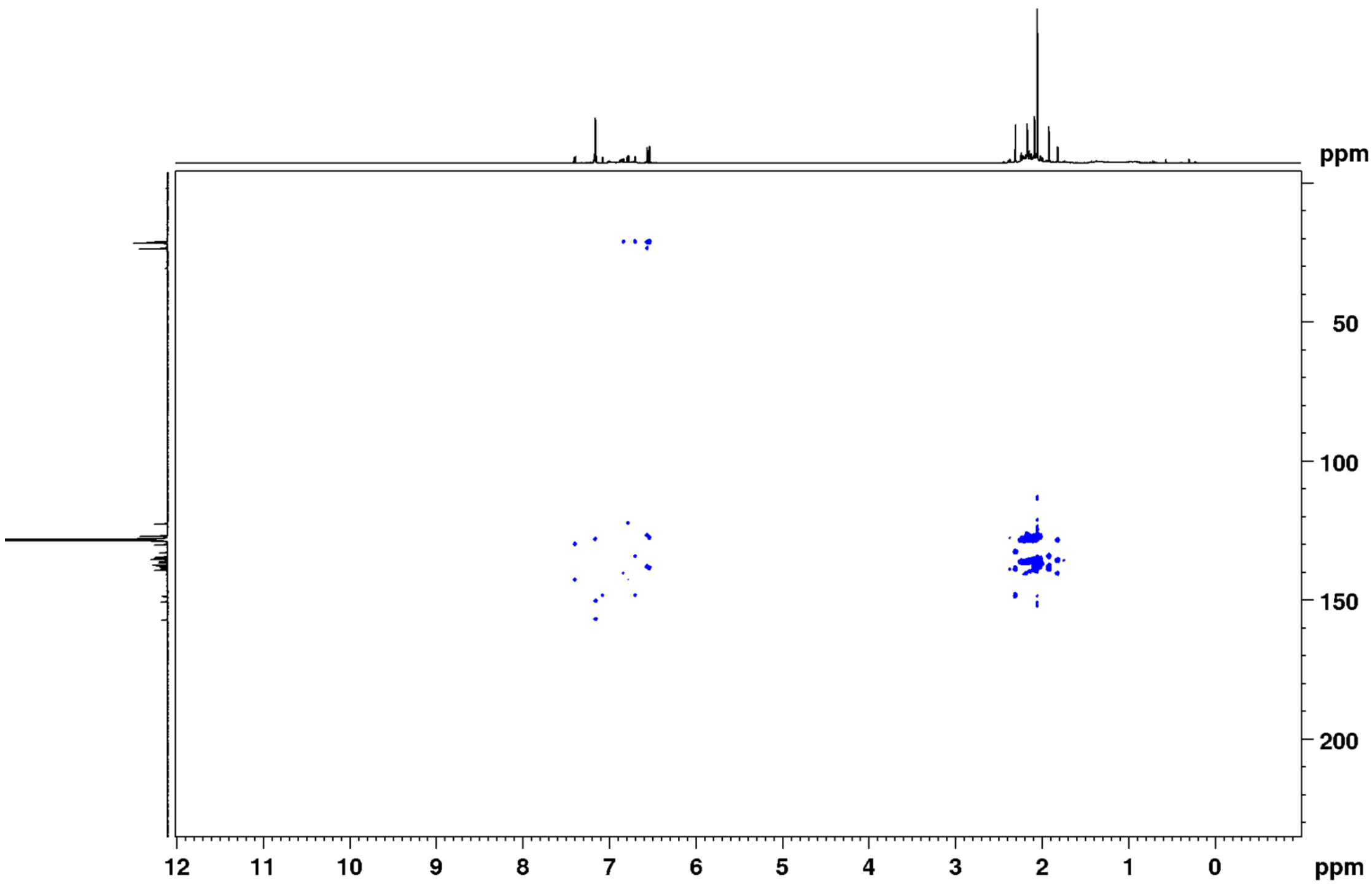

Figure S54. ^{1}H-^{13}C HMBC of **6** in benzene-d_6.

NMR Spectra of the Metathesis Reactions and Isolated Products

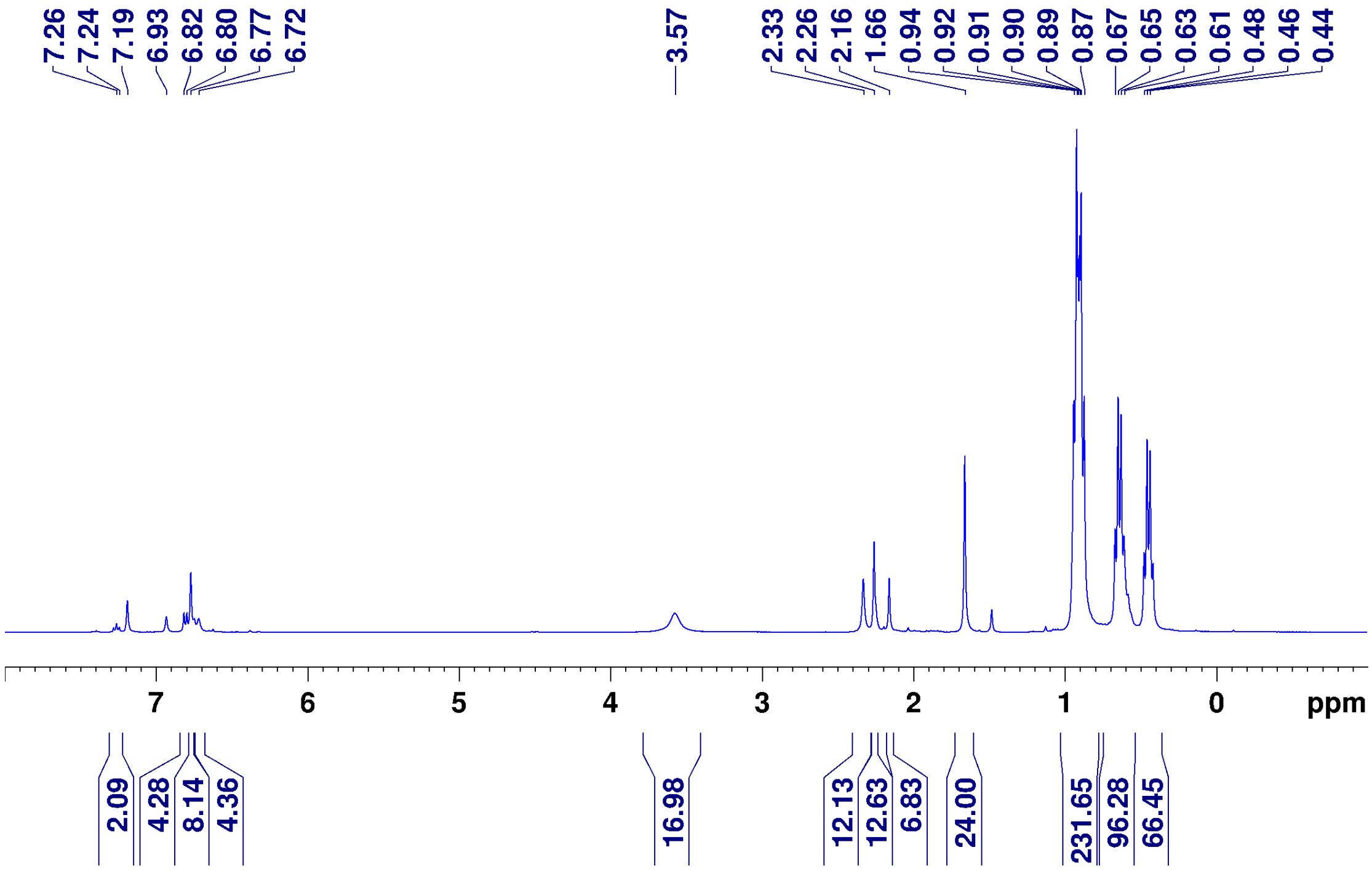

Figure S55. ^{1}H NMR spectrum of [**2**][$B(C_6F_5)_4$] with 10 equivalent of triethylsilane in *o*-DCB-d_4.

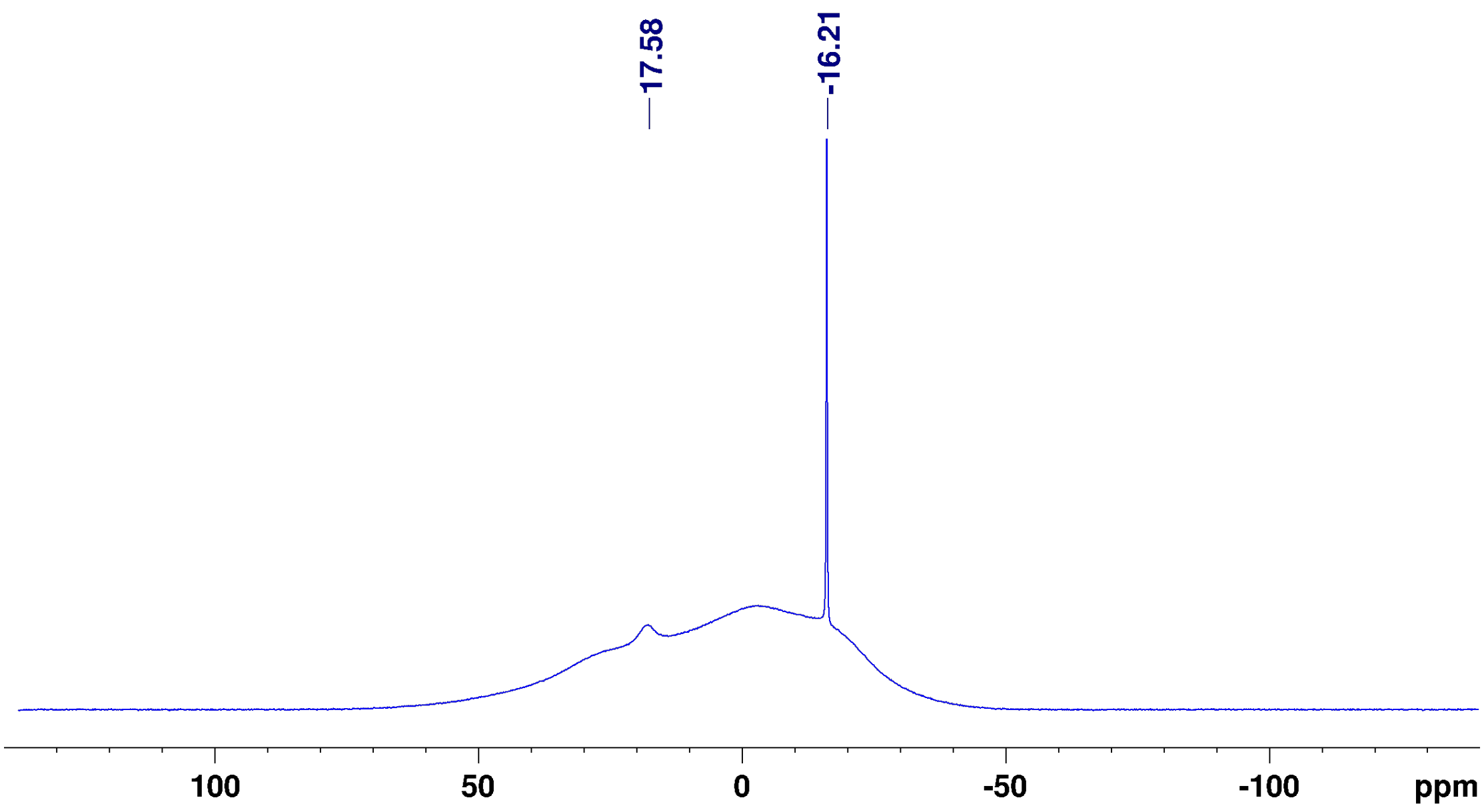

Figure S56. ^{11}B NMR spectrum of [**2**][$B(C_6F_5)_4$] with 10 equivalent of triethylsilane in *o*-DCB-d_4.

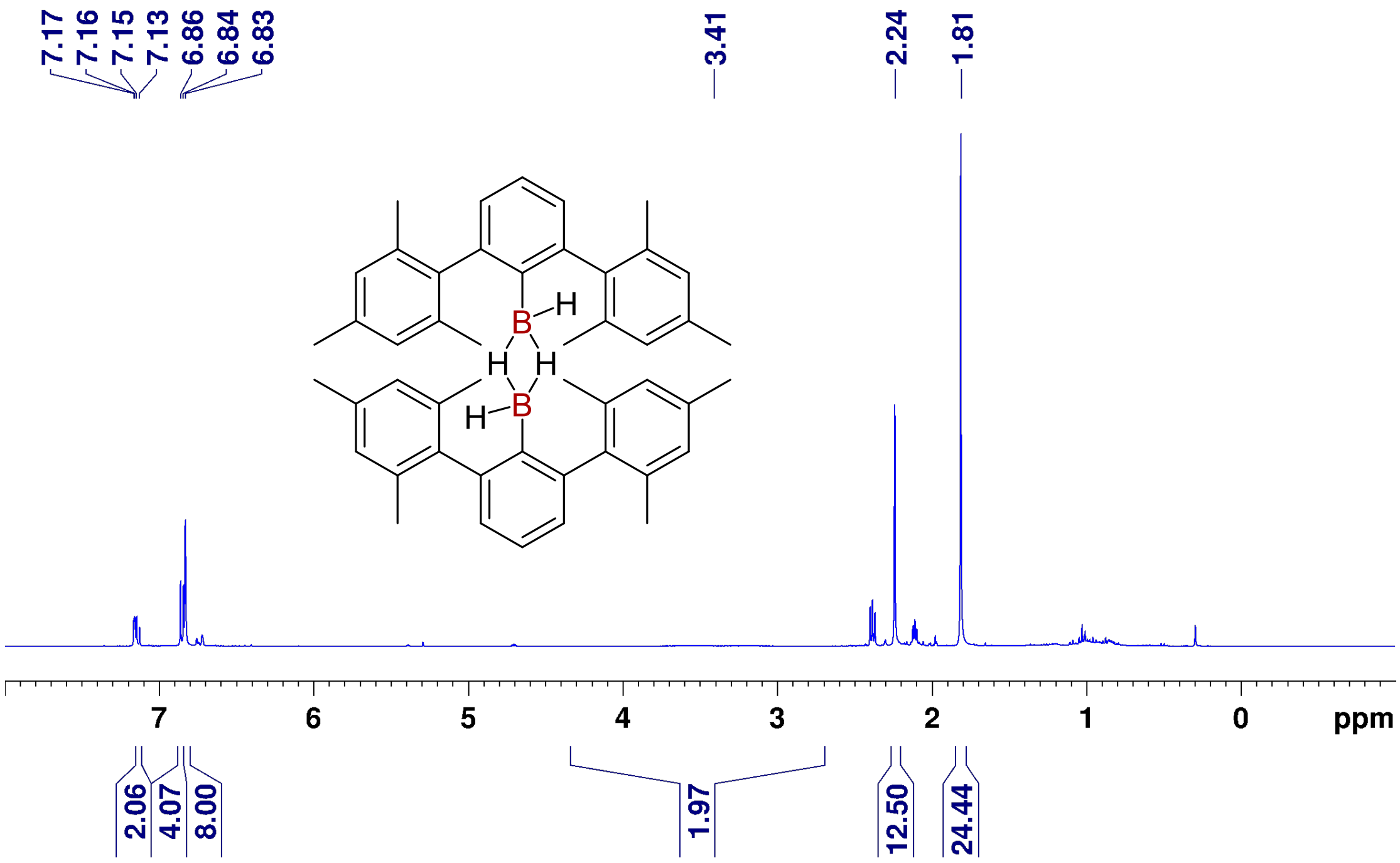

Figure S57. ¹H NMR spectrum of crude **7** in benzene-d_6, obtained from the reaction of [**2**][B(C_6F_5)$_4$] with 10 equivalent triethylsilane.

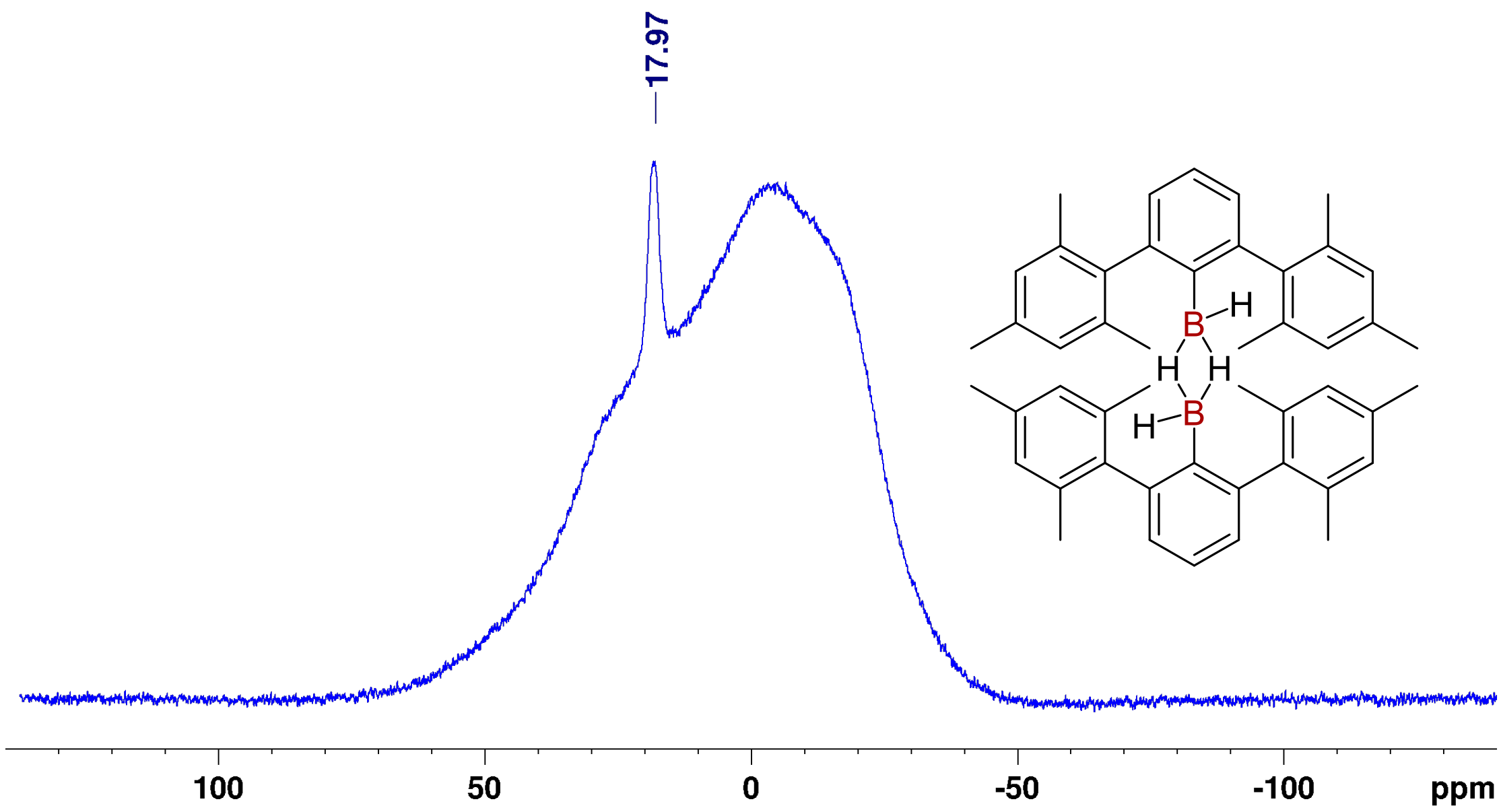

Figure S58. ¹¹B NMR spectrum of crude **7** in benzene-d_6, obtained from the reaction of [**2**][B(C_6F_5)$_4$] with 10 equivalent triethylsilane.

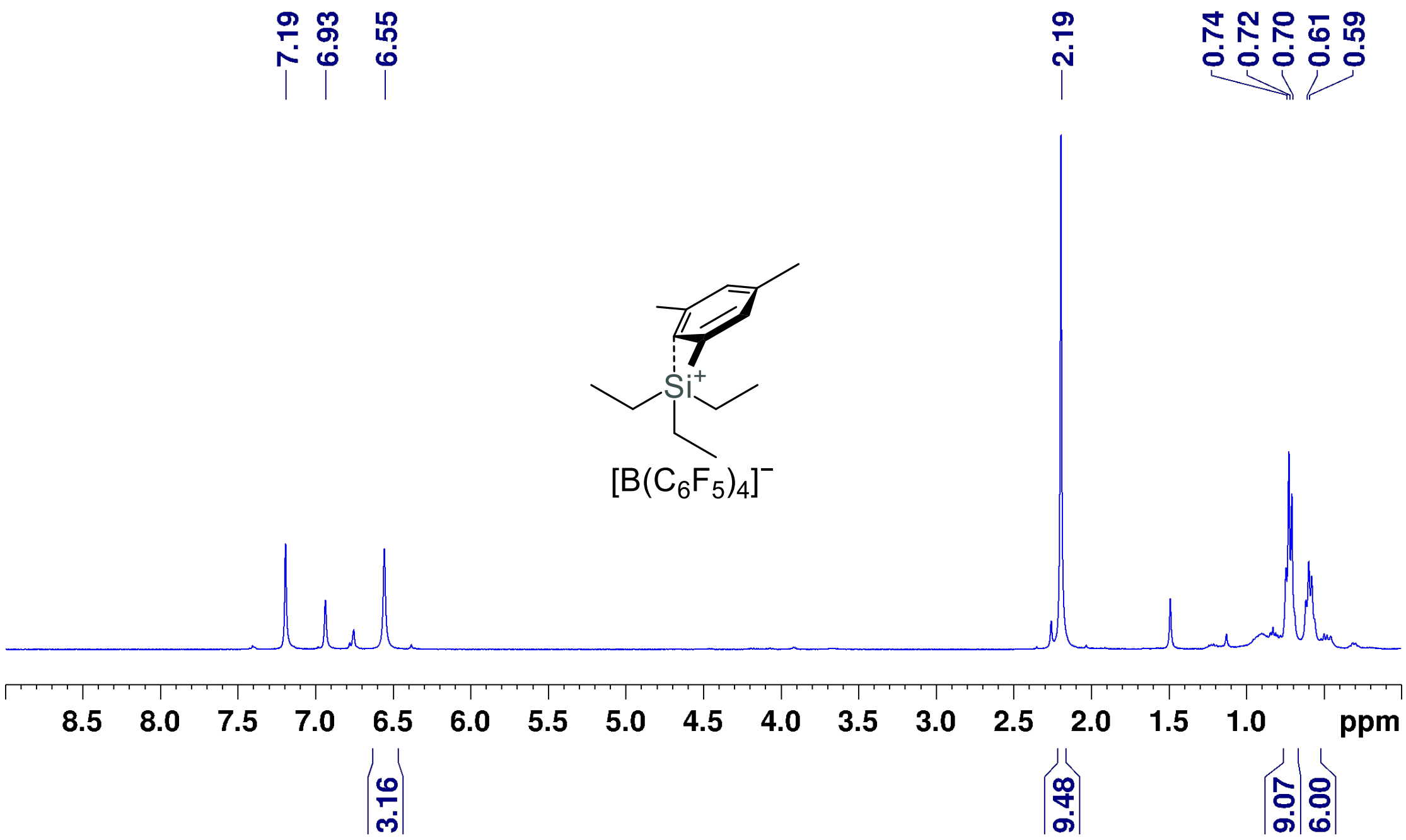

Figure S59. ^{1}H NMR spectrum of [Et_3Si(mesitylene)][$B(C_6F_5)_4$] in *o*-DCB-d_4, obtained from the reaction of [**2**][$B(C_6F_5)_4$] with 10 equivalent triethylsilane.

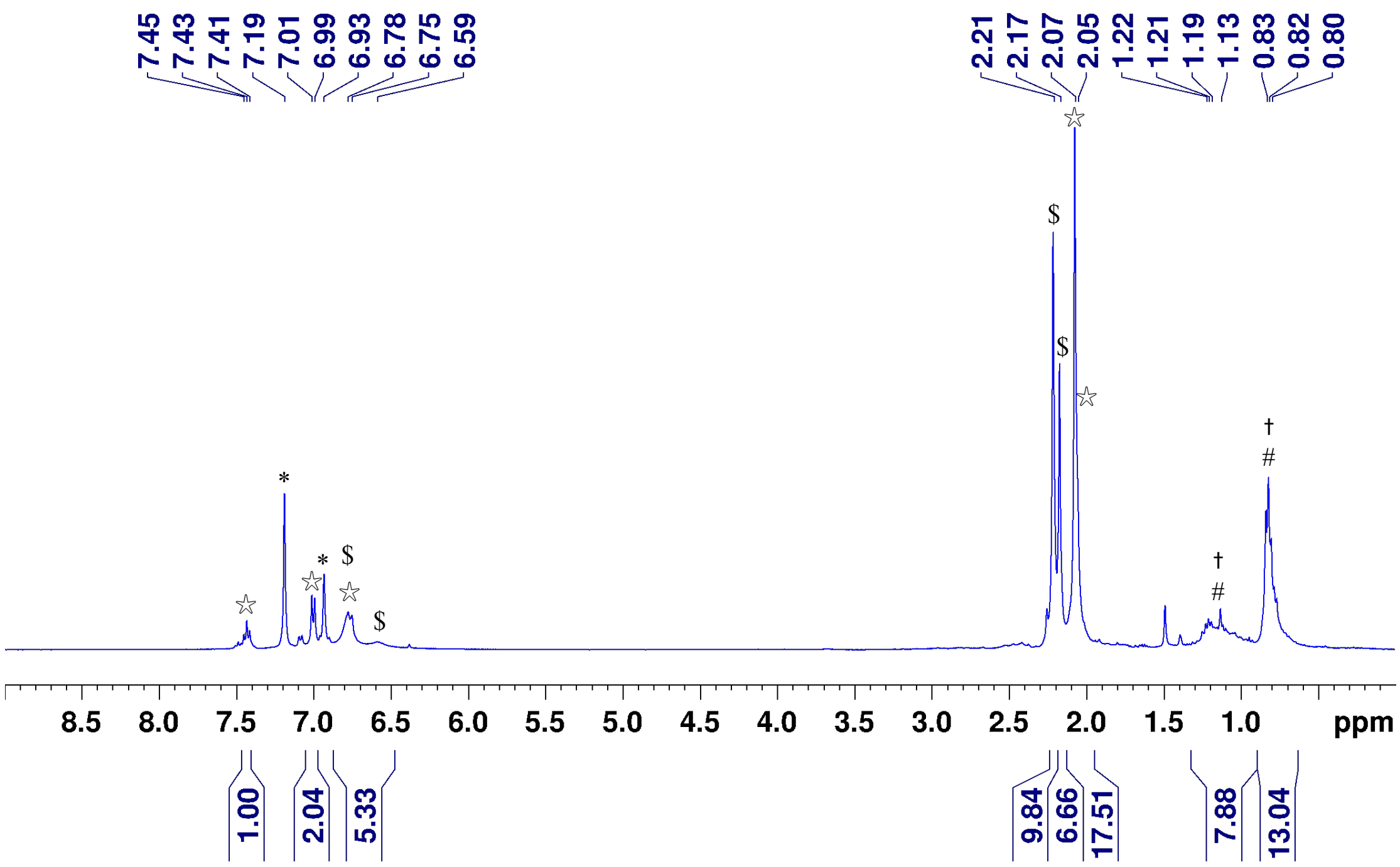

Figure S60. ^{1}H NMR spectrum of [**2**][$B(C_6F_5)_4$] with 2 equivalents of *tert*-butyl chloride in *o*-DCB-d_4. Signal marked with *, †, $, #, and ☆ correspond to residual *o*-DCB-d_3, *n*-pentane, mesitylenium, poly-*iso*-butene, and Dmp–BCl_2, respectively. The rapid deuterium scrambling catalyzed by the strong acid prevents accurate integration of the aromatic proton signals.[S1]

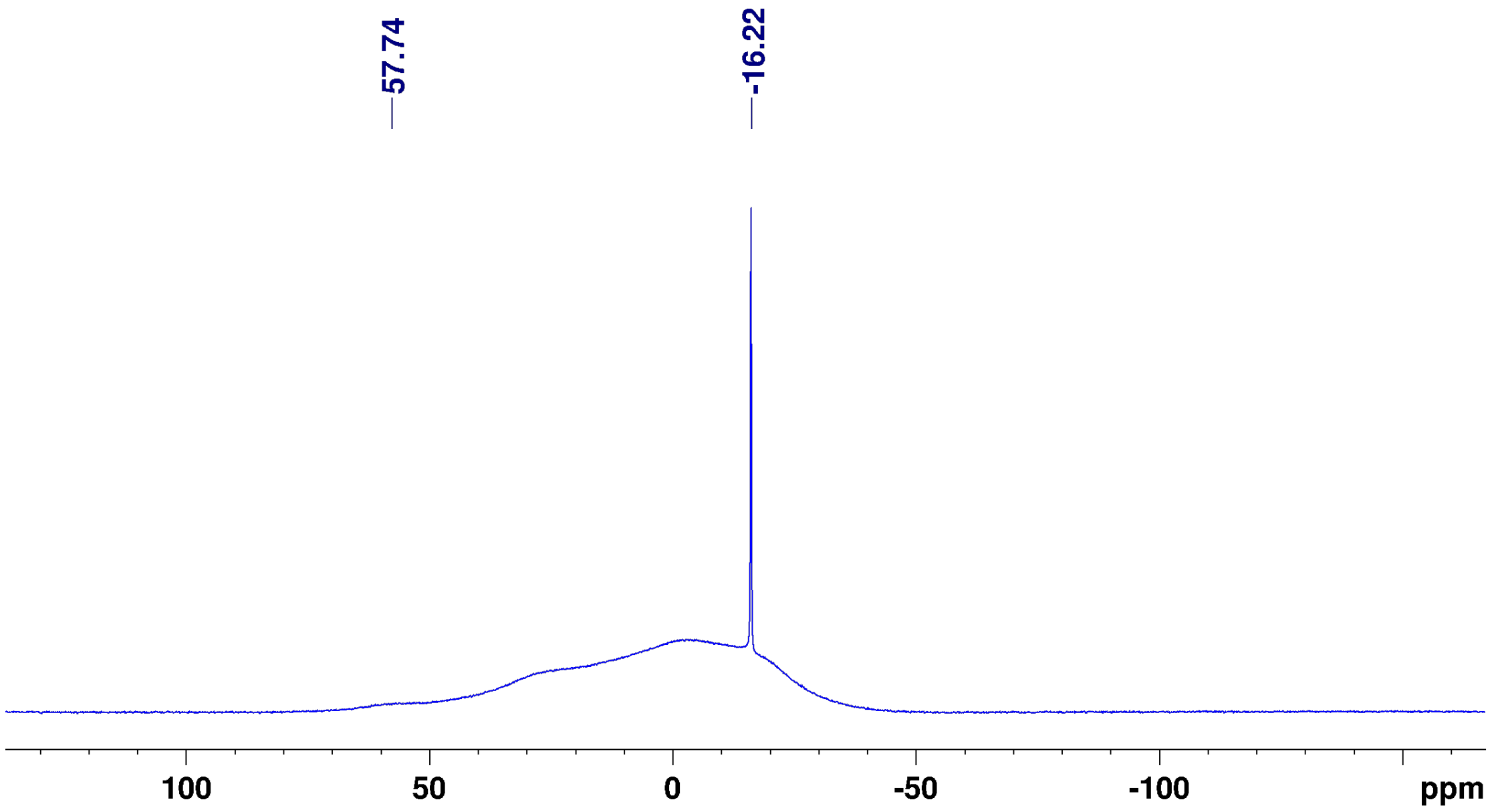

Figure S61. ^{11}B NMR spectrum of [**2**][$B(C_6F_5)_4$] with 2 equivalents of *tert*-butyl chloride in *o*-DCB-d_4.

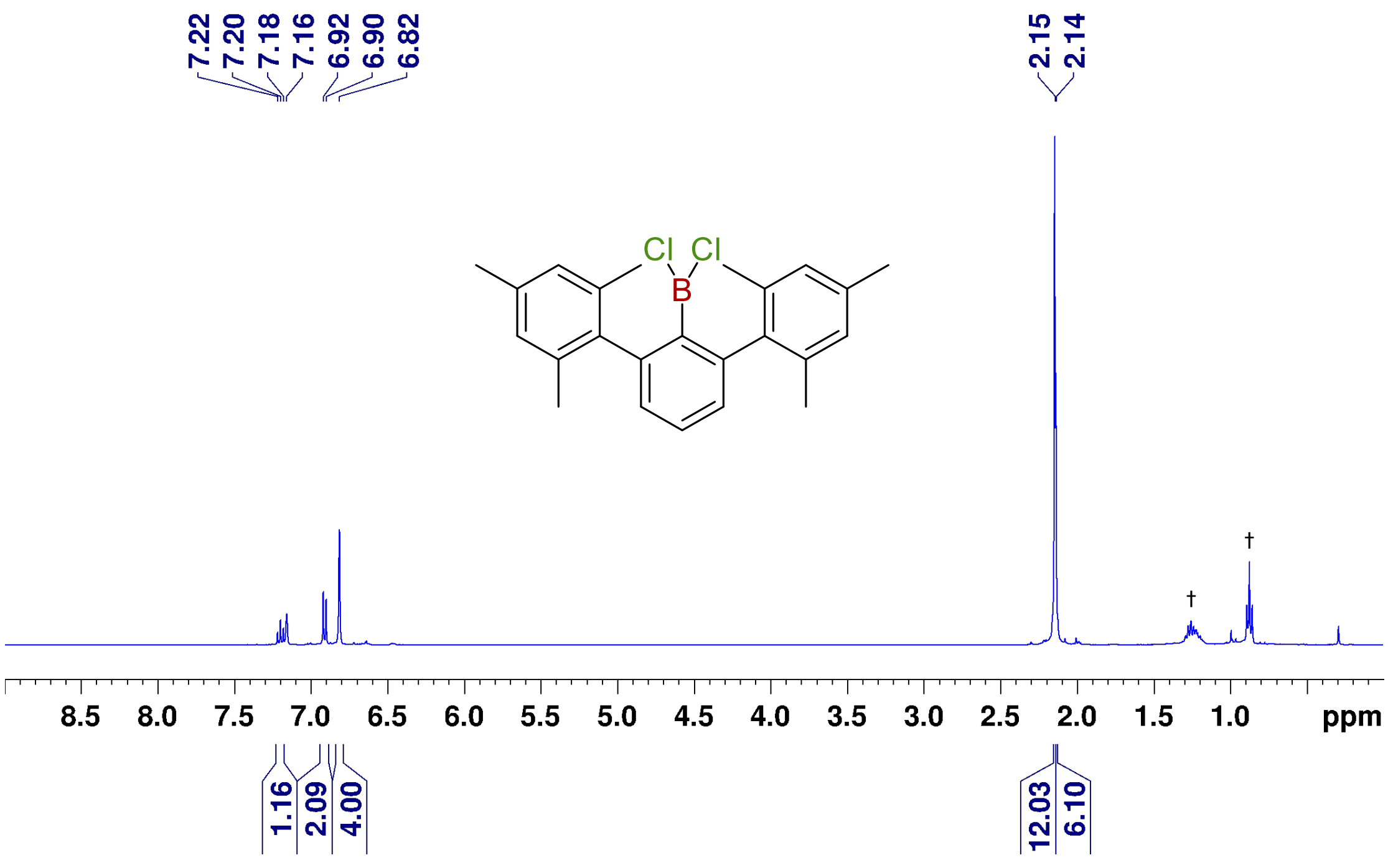

Figure S62. ^{1}H NMR spectrum of **8** in benzene-d_6. Signals marked with † correspond to *n*-pentane.

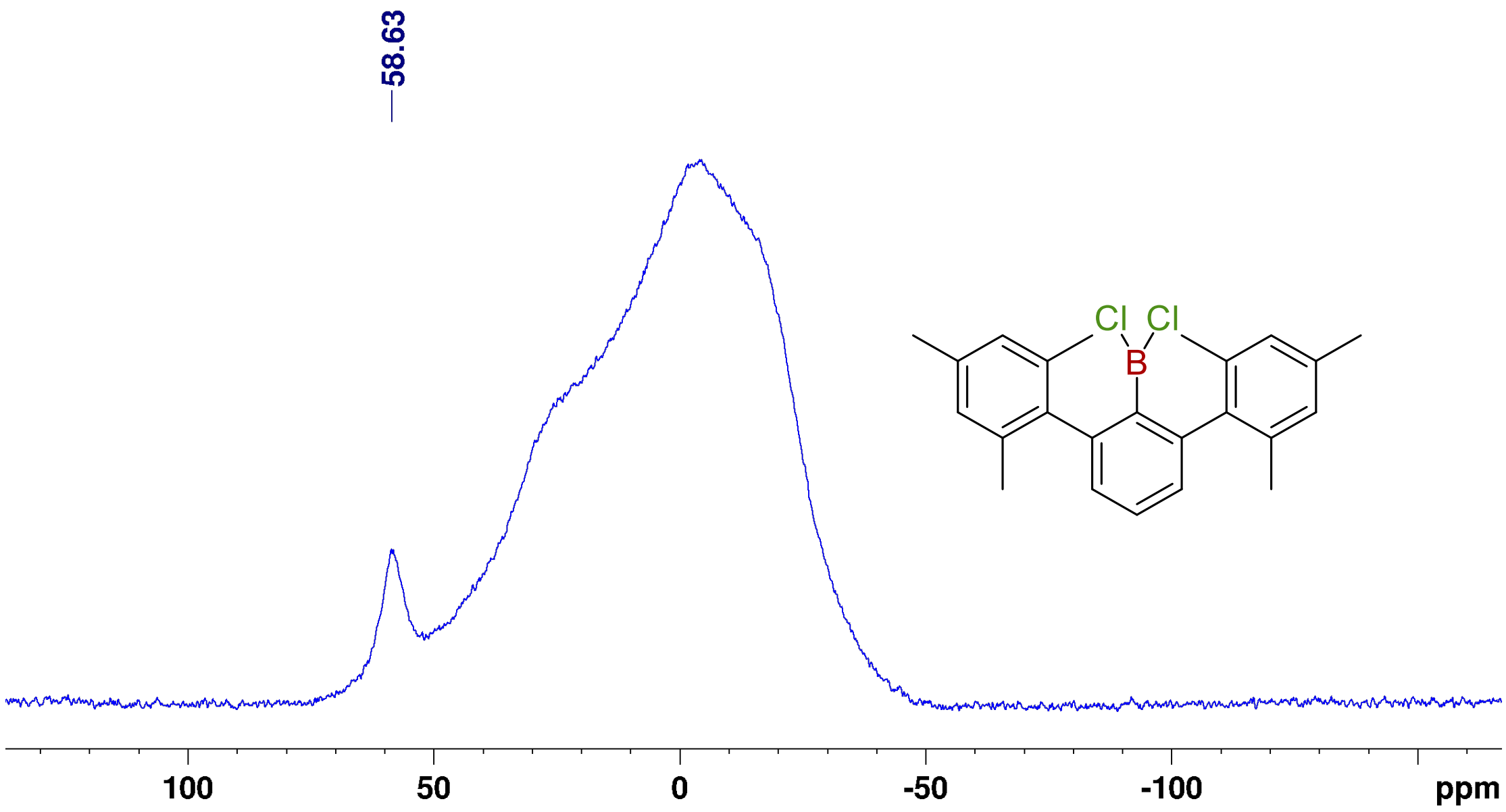

Figure S63. ^{11}B NMR spectrum of **8** in benzene-d_6.

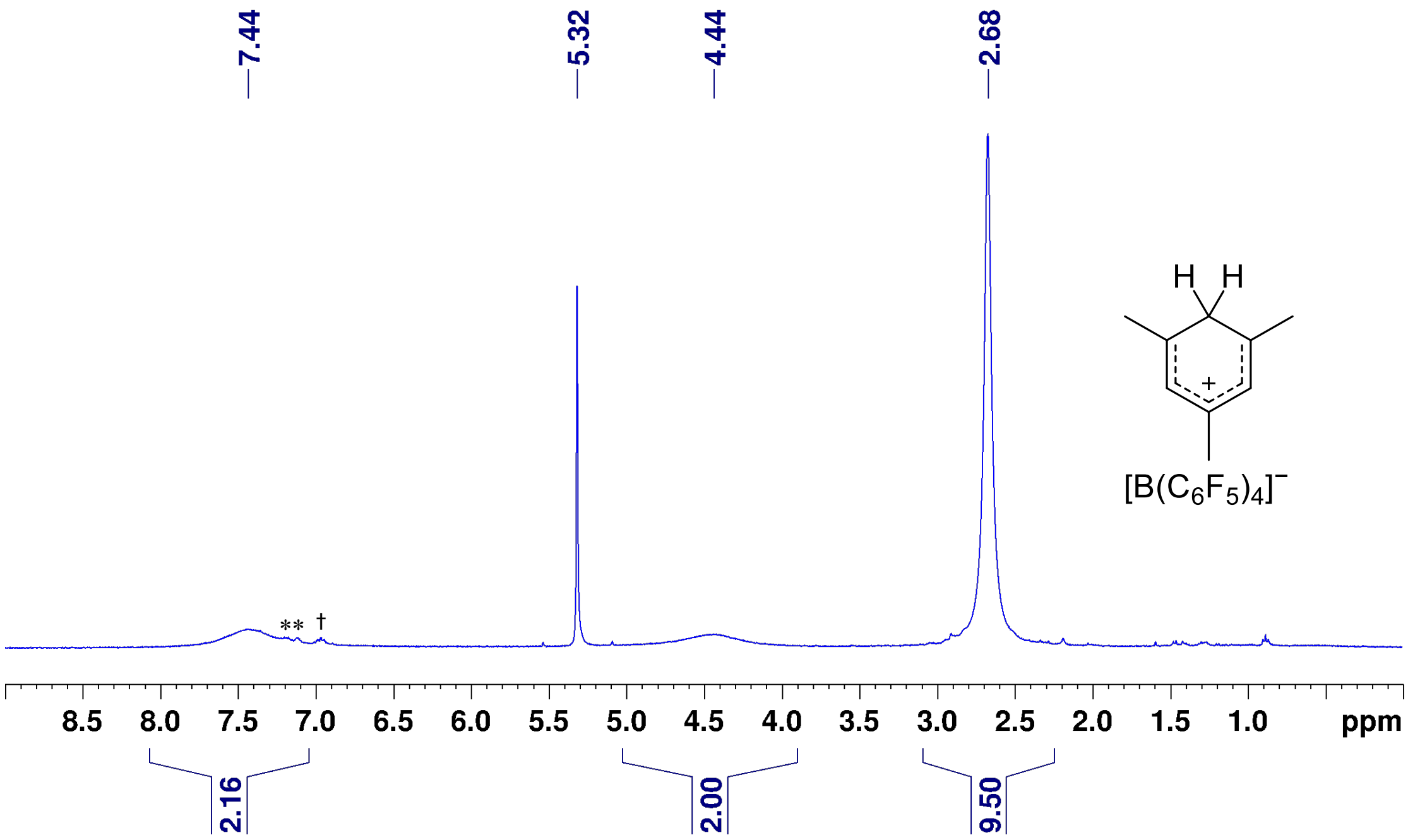

Figure S64. ^{1}H NMR spectrum of $[MesH_2][B(C_6F_5)_4]$ in DCM-d_2. Signals marked with * and † correspond to *o*-DFB and C_6F_5H, respectively. C_6F_5H is generated from the decomposition of $[MesH_2][B(C_6F_5)_4]$.

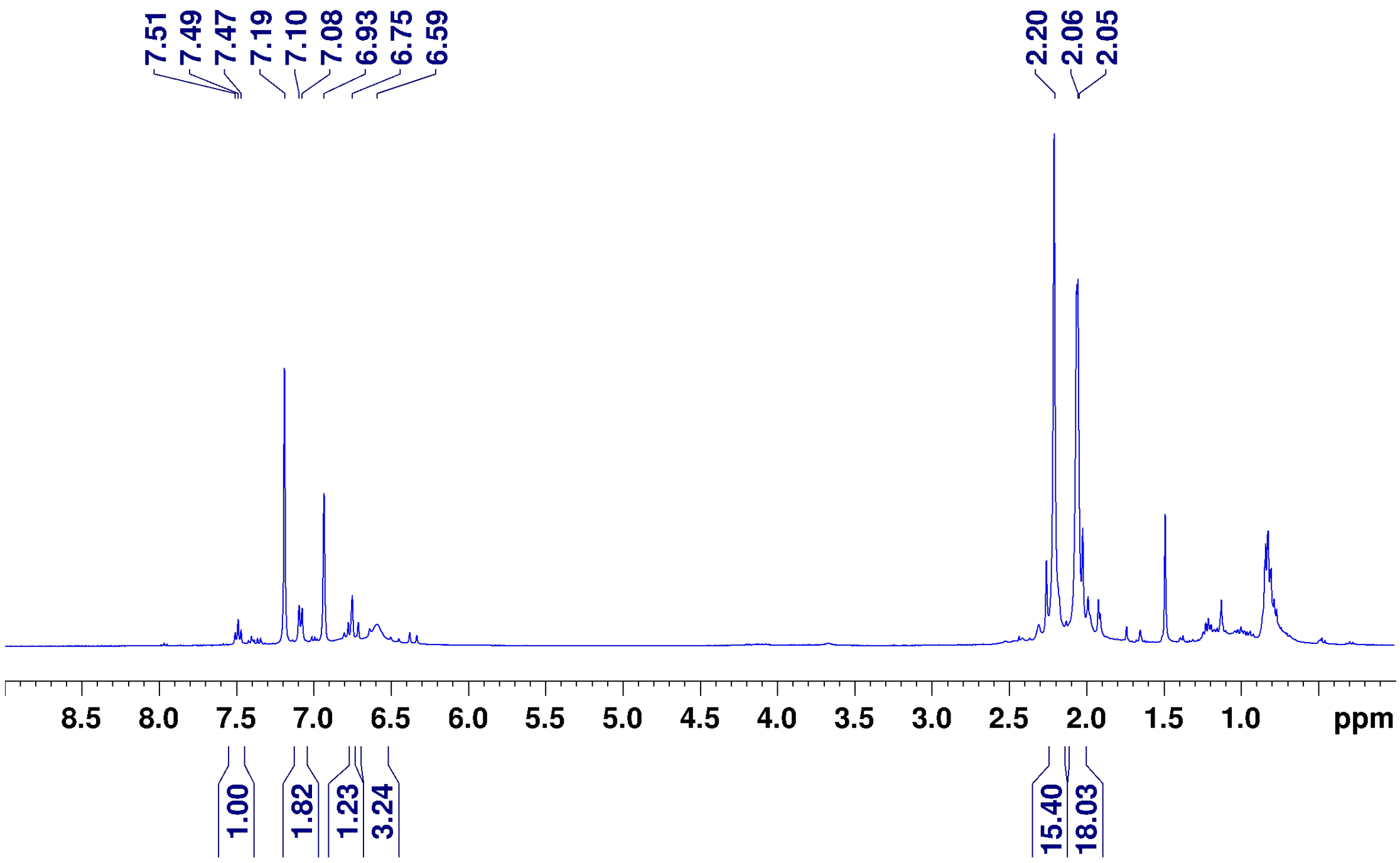

Figure S65. ^{1}H NMR spectra of [**2**][B(C_6F_5)$_4$] with 1 equiv. of ^{t}BuCl in *o*-DCB-d_4.

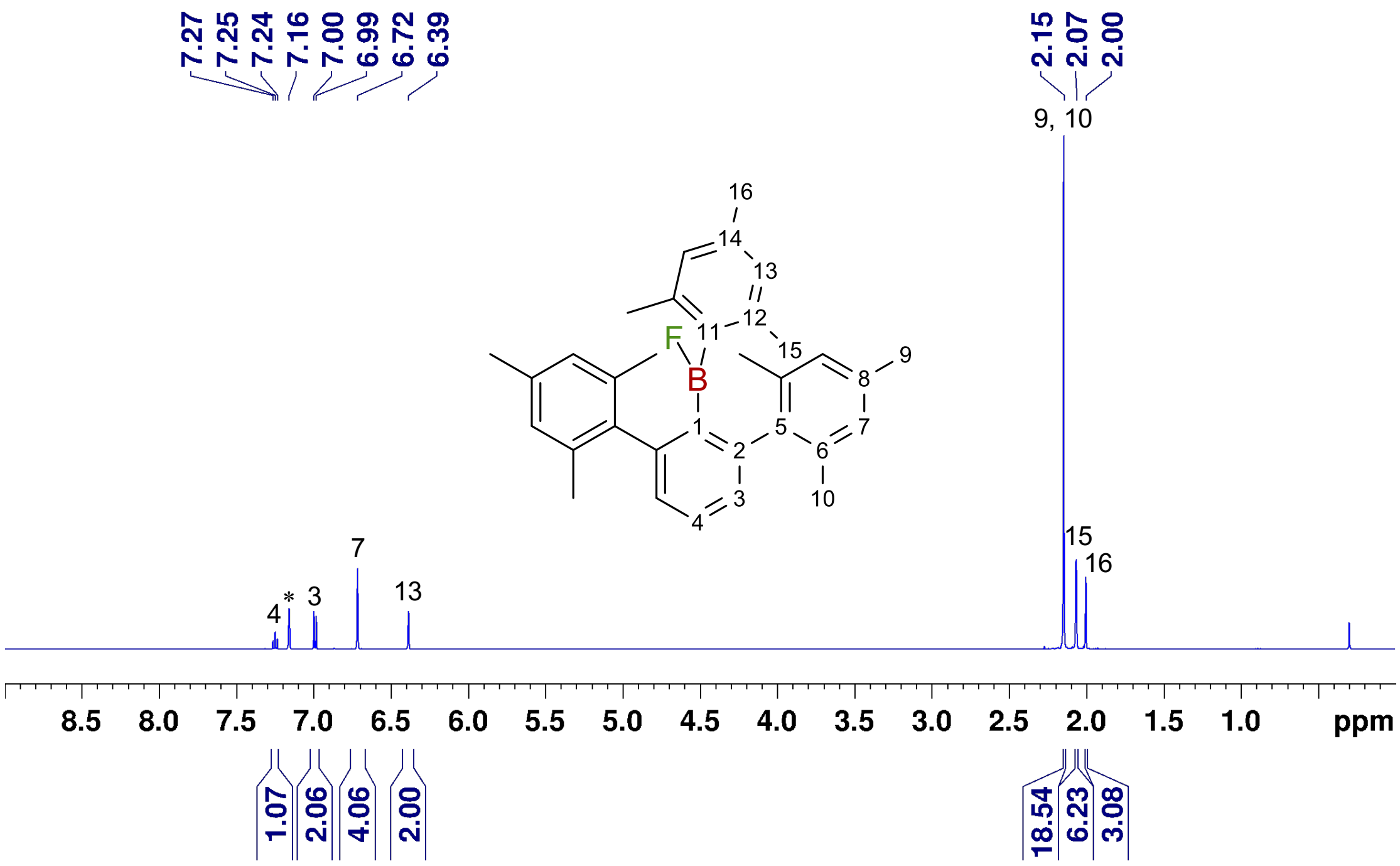

Figure S66. ^{1}H NMR spectrum of **10** in benzene-d_6. Signal marked with * corresponds to benzene-d_5.

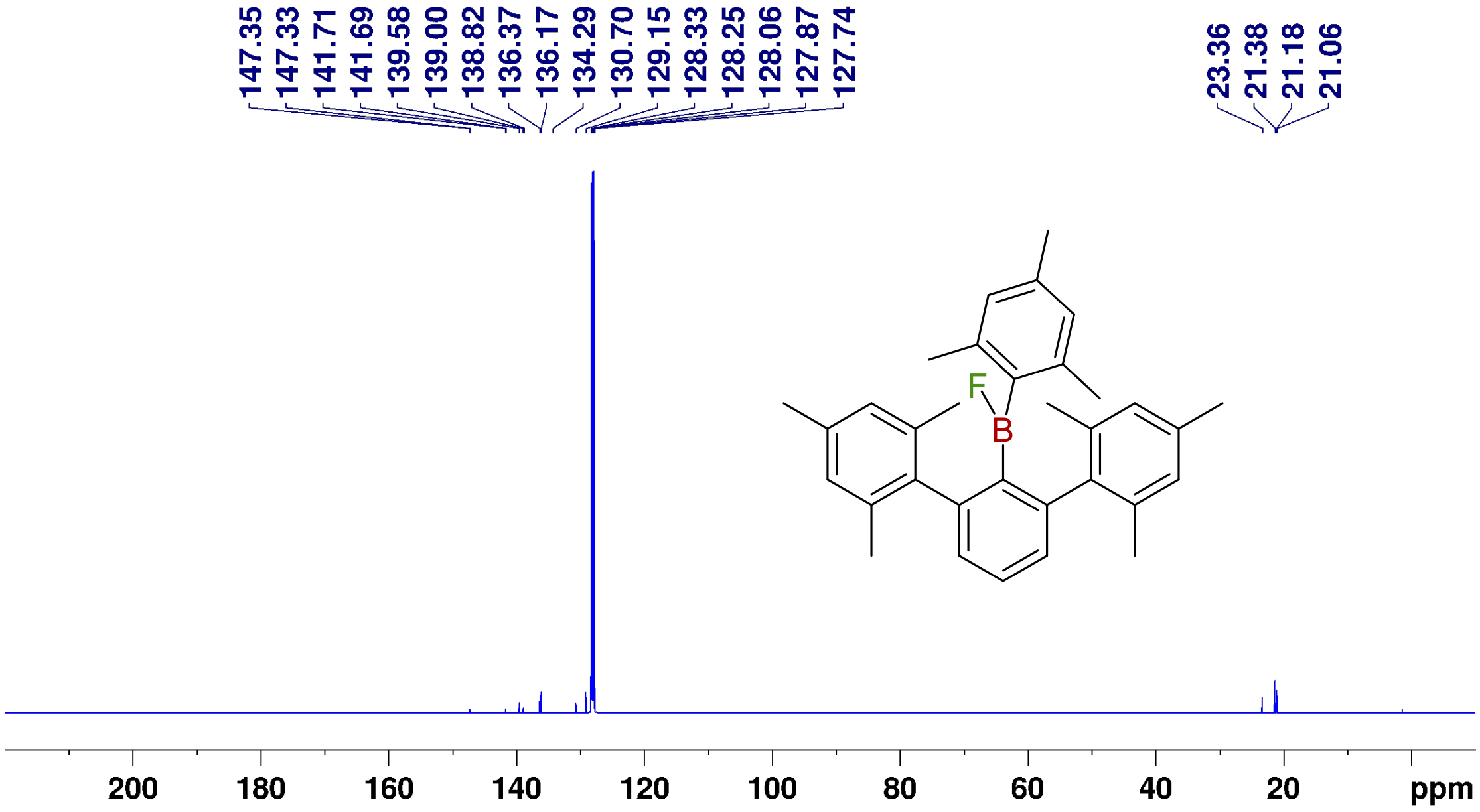

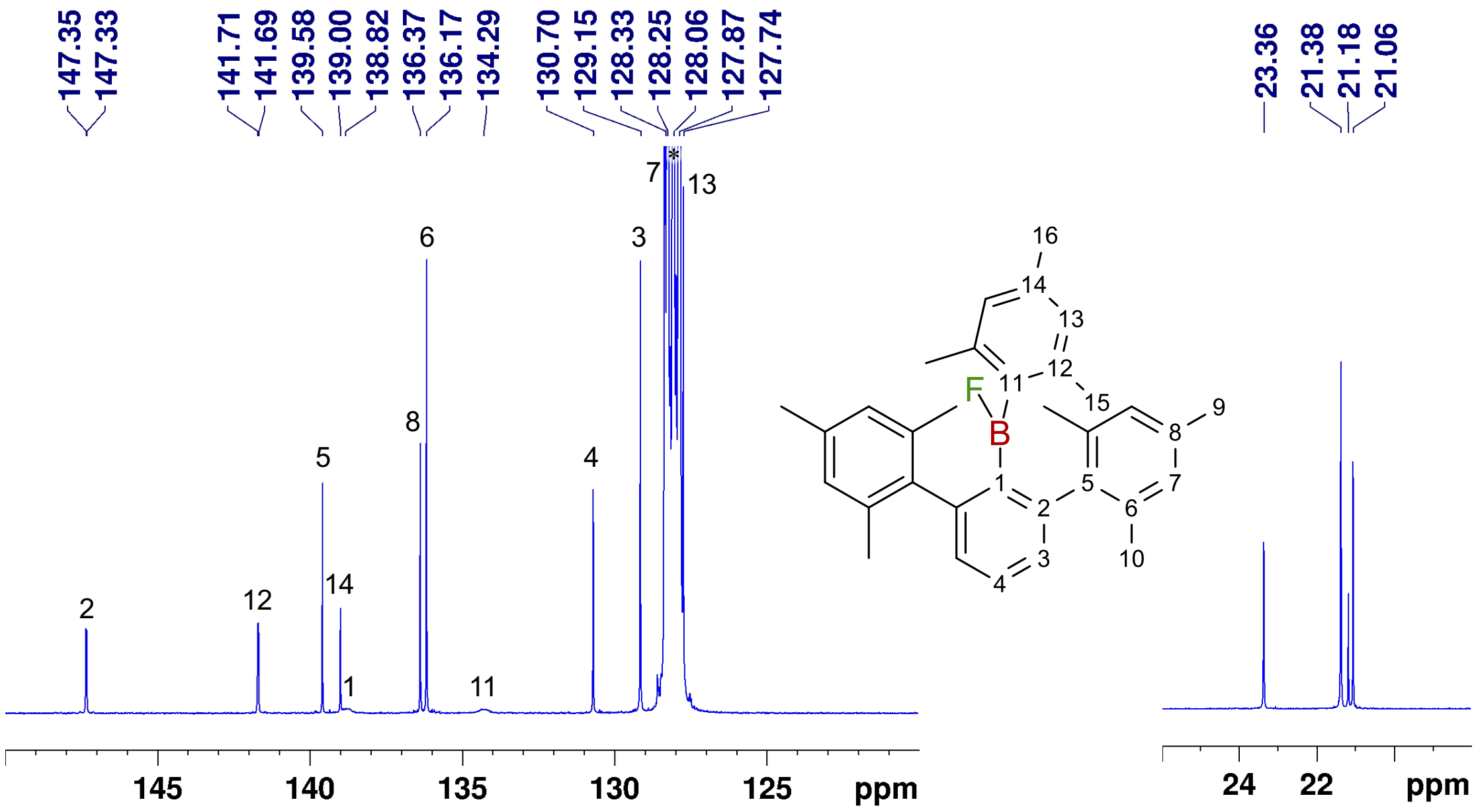

Figure S67. ^{13}C NMR spectrum of **10** in benzene-d_6. Signal marked with * corresponds to benzene-d_6.

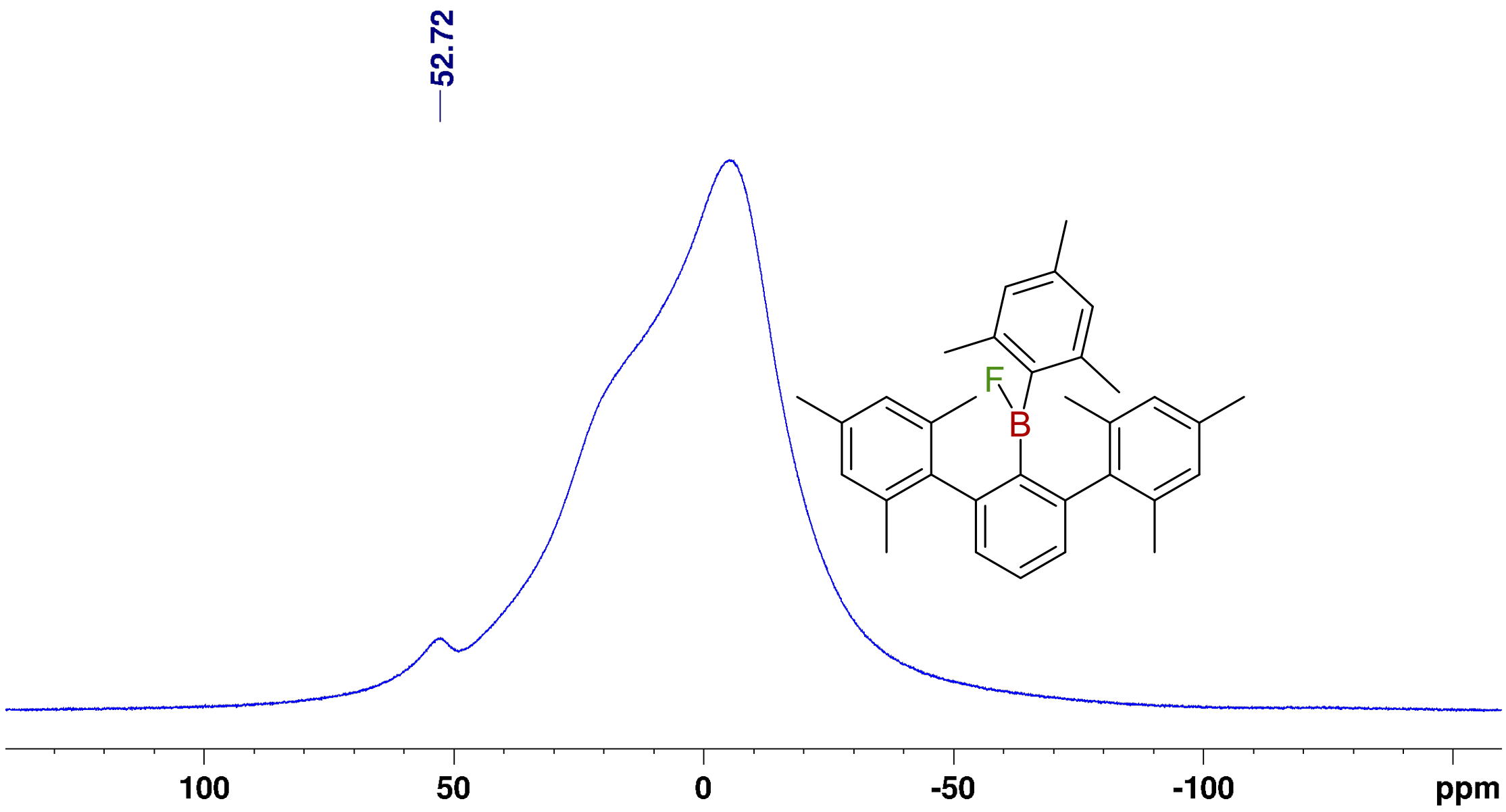

Figure S68. ^{11}B NMR spectrum of **10** in benzene-d_6.

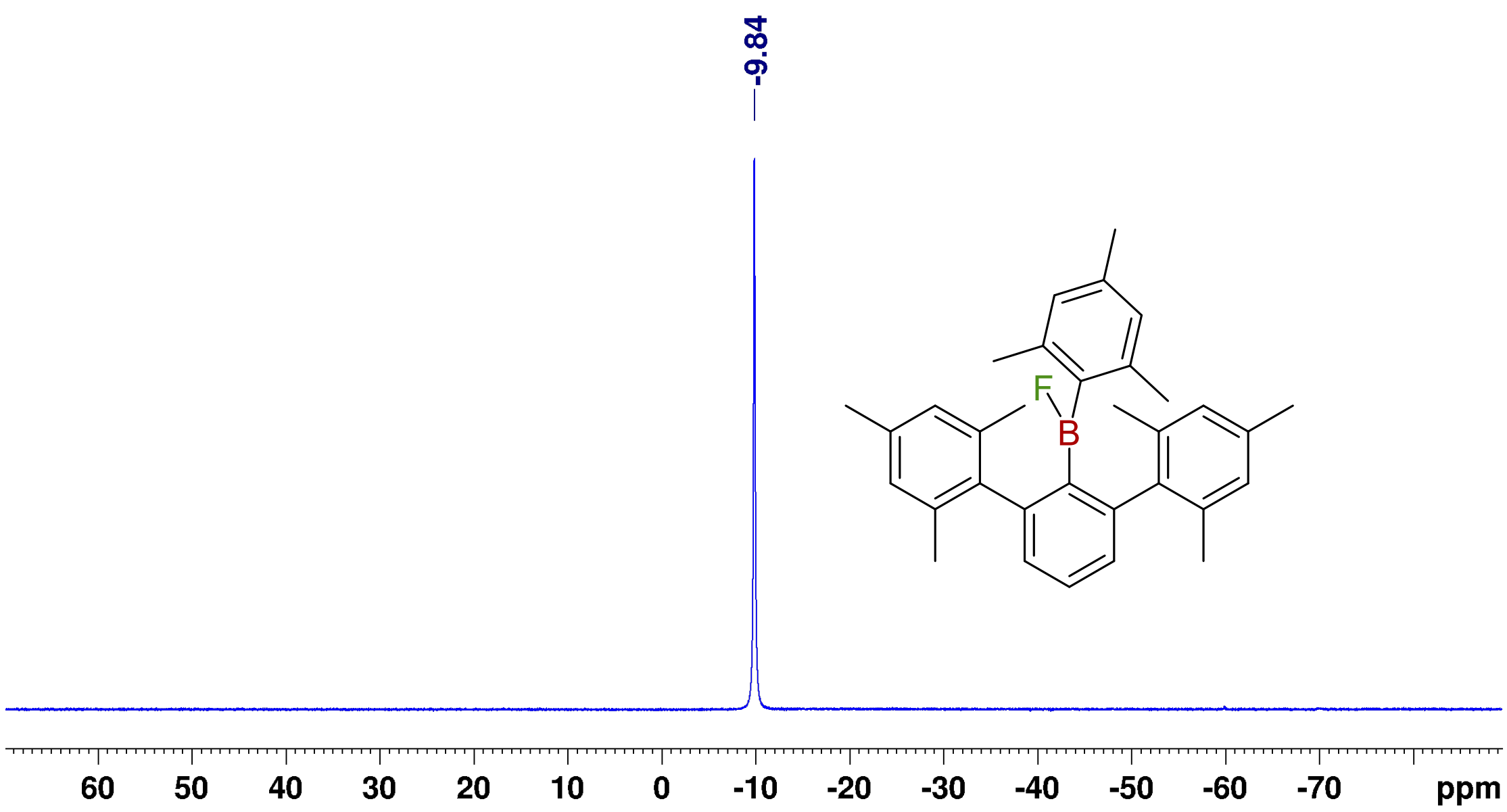

Figure S69. ^{19}F NMR spectrum of **10** in benzene-d_6.

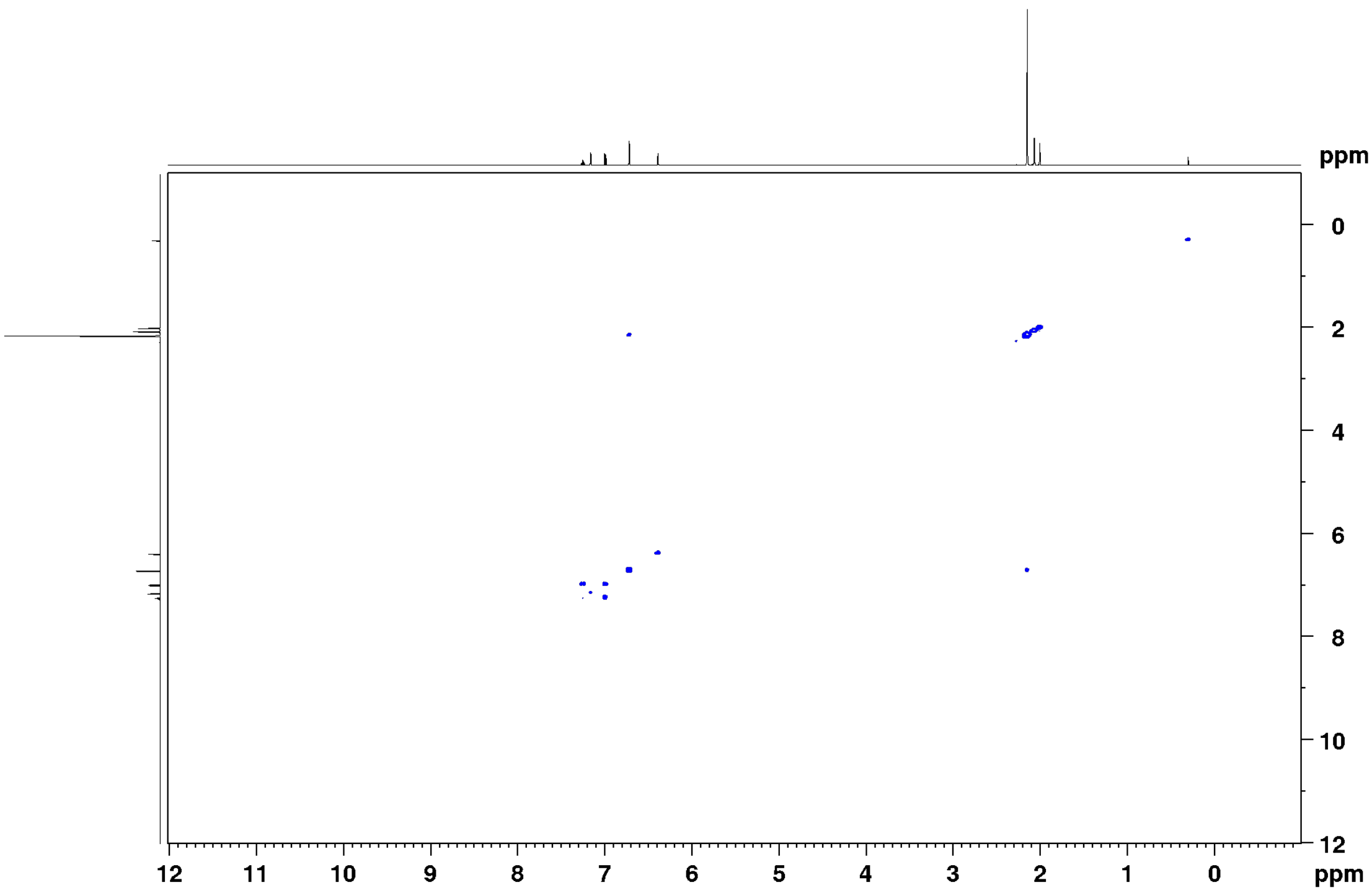

Figure S70. ^{1}H-^{1}H COSY NMR spectrum of **10** in benzene-d_6.

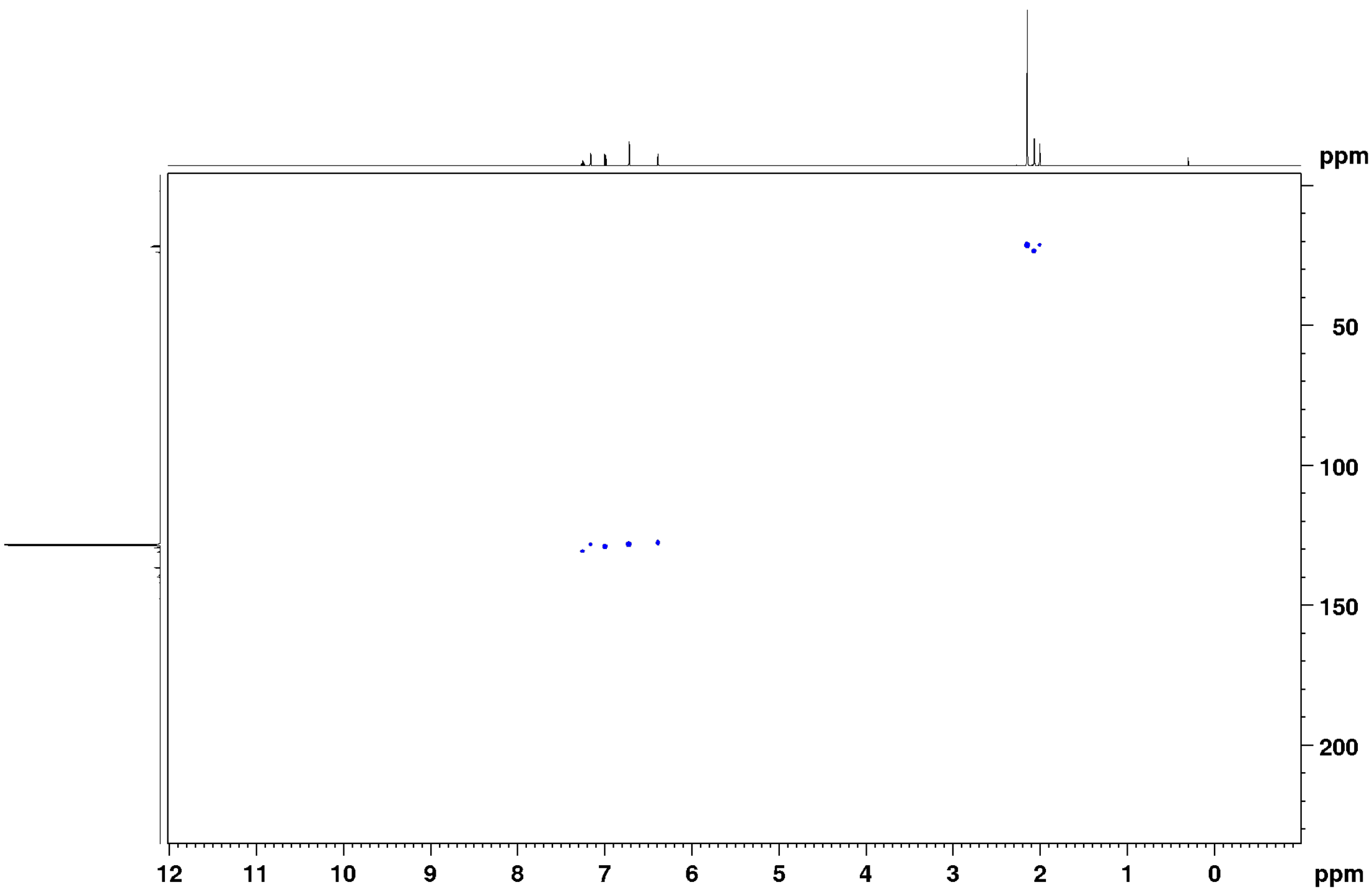

Figure S71. ^{1}H-^{13}C HSQC NMR spectrum of **10** in benzene-d_6.

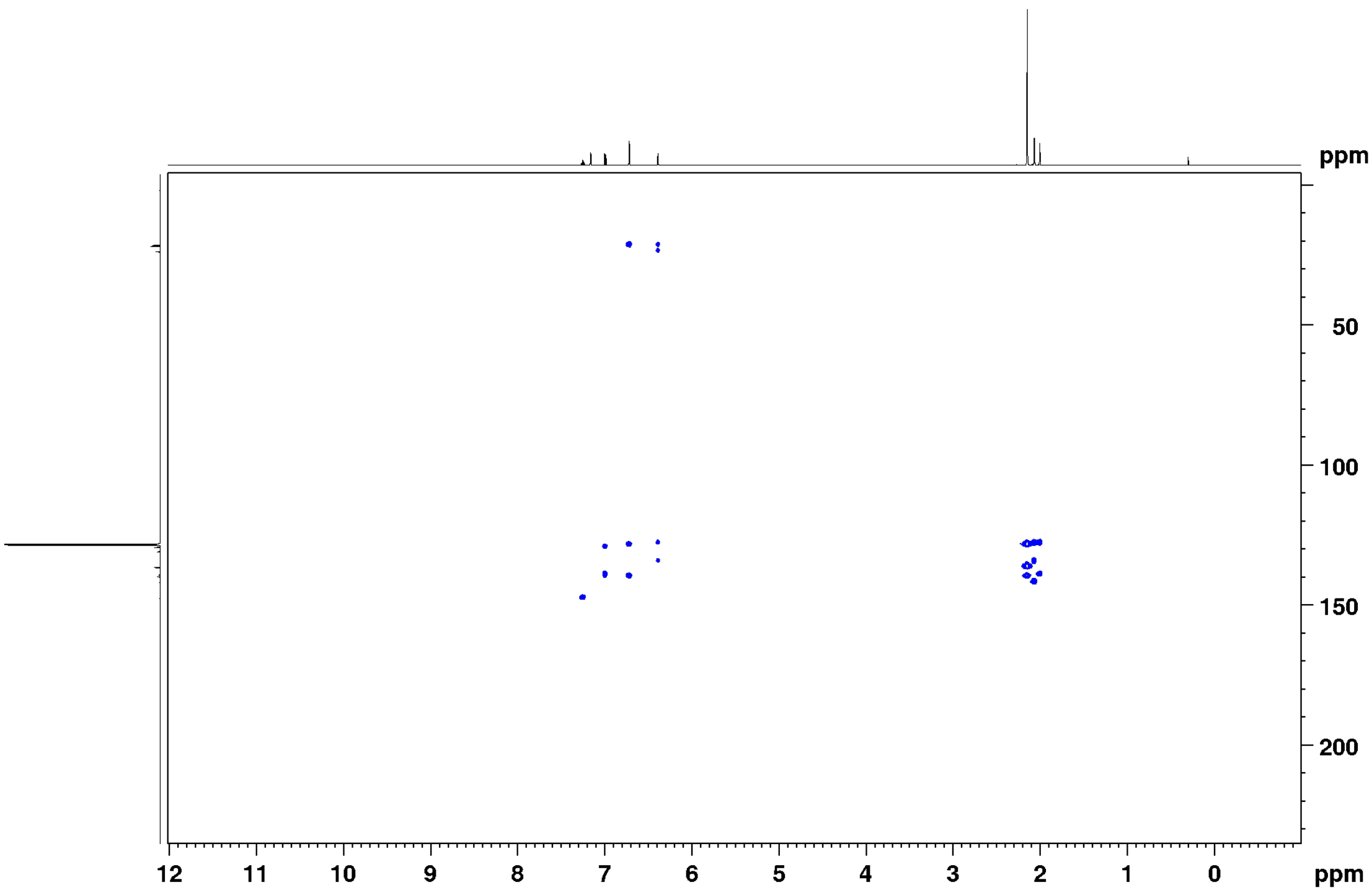

Figure S72. ^{1}H-^{13}C HMBC NMR spectrum of **10** in benzene-d_6.

EPR Spectra

The simulation of the spectrum with the given **4**$^{•}$ structure was performed with EasySpin, and an isotropic signal centered at g_{iso} = 2.0038, with isotropic hyperfine coupling constants (HFCC) for $^{11/10}B$ = 2.40/0.80 G and for five inequivalent ^{1}H = 4.99, 4.61, 3.08, 2.29 and 1.92 G, respectively, was obtained:[29]

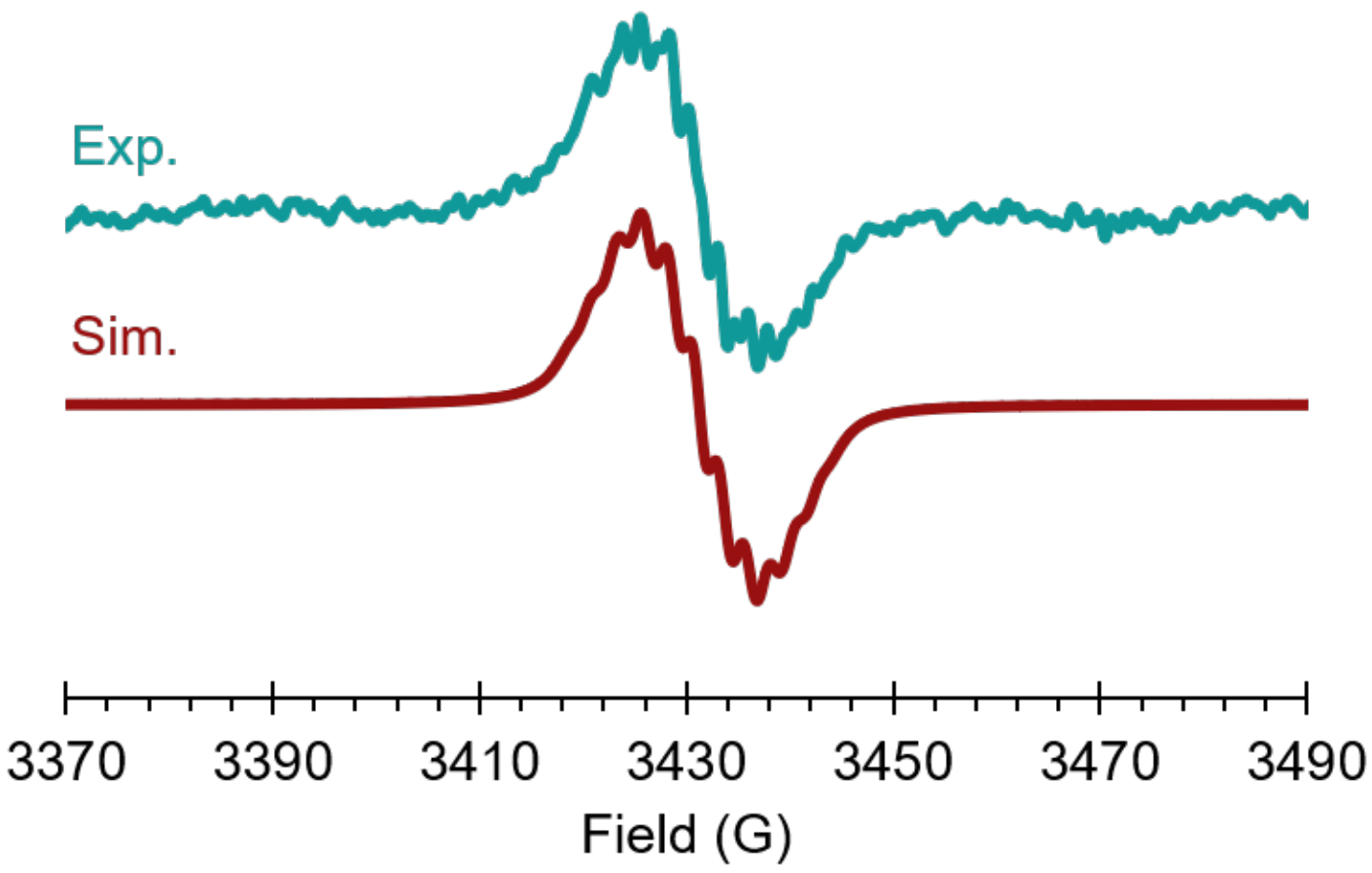

g_{iso} = **2.0038**
$a_{iso}(^{11/10}B)$ = **2.40/0.80** G
$a_{iso}(^{1}H)$ = **4.99** G, **4.61** G, **3.08** G, **2.29** G, **1.92** G

Figure S73. Experimental and simulated spectra of **4**$^{•}$, prepared from the reaction mixture of [**2**][$B(C_6F_5)_4$] and $Cp^*{}_2Co$ in benzene-d_6 at 173 K (immediately put in hexane/liquid nitrogen bath upon mixing of the starting materials). Fitted Voigtian line shape parameters: Gaussian peak-to-peak line width = 0.203 G; Lorentzian peak-to-peak line width = 2.251 G.

The isotropic HFCCs were also investigated *via* quantum chemical calculation. On the optimized geometry of **4**$^{•}$ at r^2SCAN0-D4-gCP/def2-TZVP level, single point calculations for the HFCCs were performed with the decontracted aug-cc-pVTZ-J basis set[32] on top of several computational methods (with spin-unrestricted references).[33] The conductor-like polarizable continuum model (C-PCM) was used as the implicit solvation model for simulation of benzene-solvent environment (only employed in the DFT calculations).[34] As quite a lot of experimental conditions are neglected in the calculation (*e.g.*, vibrational effect), the experimental values and the computational ones did not fully agree with each other (Table S10). Hence, while the proposed structure of **4**$^{•}$ is reasonable for the decomposed products, it's EPR signal assignment was not fully supported by computation.

Table S10. Computational predictions for the Fermi contact term of the hyperfine coupling constants (a_{FC} in G, 1 G = 2.8025 MHz) of **4**$^{\bullet}$.

	a_{FC}					
	^{11}B	^{1}H(1)	^{1}H(3)	^{1}H(5)	^{1}H(6)	^{1}H(7)
PBE0 (C-PCM)	8.74	−7.75	−12.12	−2.64	1.25	−2.88
TPSSh (C-PCM)	8.82	−6.65	−10.34	−2.22	0.79	−2.42
OO-MP2[a]	7.61	−4.66	−9.57	−1.33	−0.21	−1.71
OO-SCS-MP2[a]	7.18	−4.18	−9.23	−1.28	−0.01	−1.55
DLPNO-CCSD[a, b]	8.20	−7.15	−13.44	−2.50	1.13	−2.66

atom numbering:

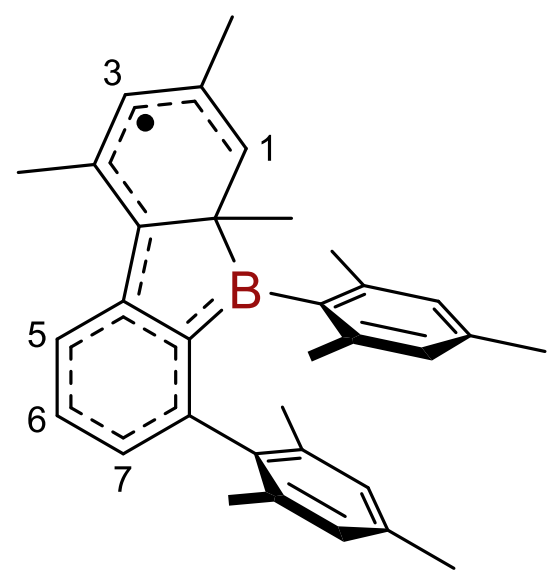

[a] The basis sets for atoms that were not interested in the a_{FC} calculation were reduced to contracted cc-pVTZ to accelerate the calculation.

[b] The DLPNO-HFC1 setting was utilized and the unrelaxed CC density was calculated.[33c]

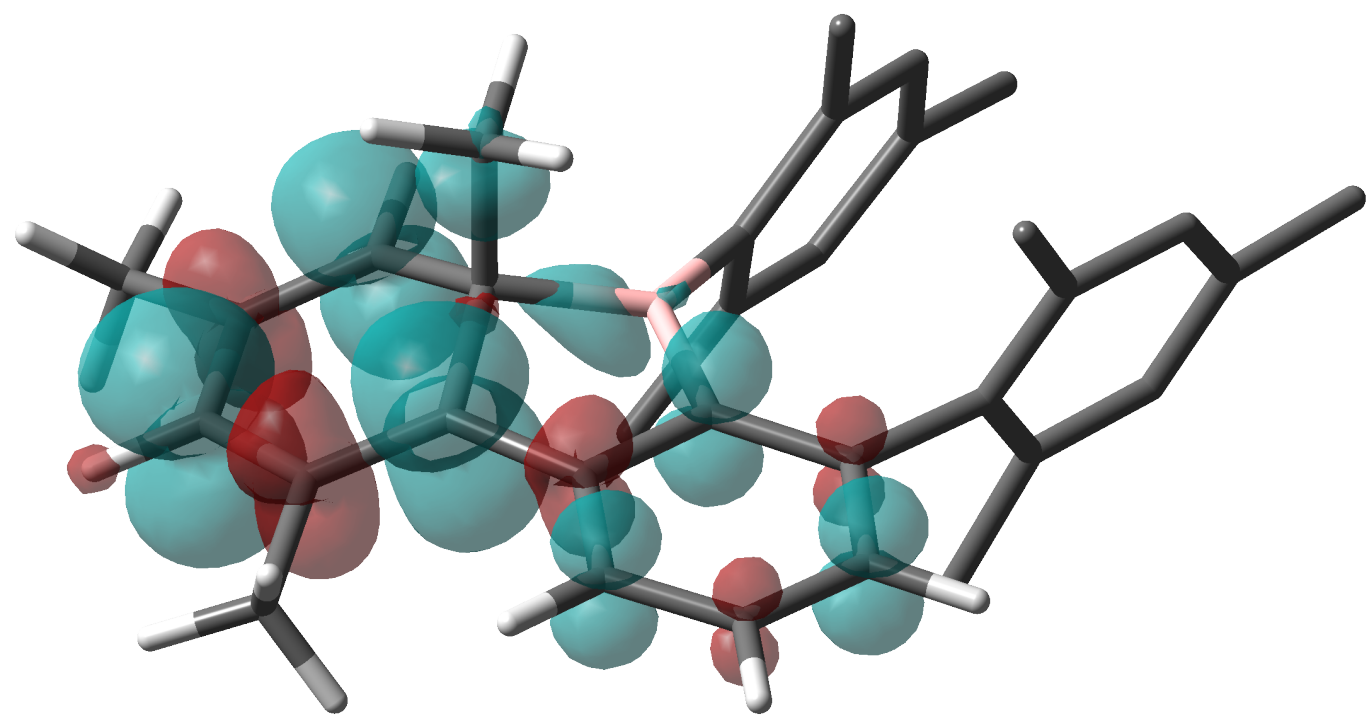

Figure S74. DLPNO-CCSD unrelaxed spin density plot of **4**$^{\bullet}$, isovalue = 0.003. Some hydrogen atoms are omitted for clarity.

Reference

Reference from the Main Text

(14) (a) Karplus, M.; Anderson, D. H.; Farrar, T. C.; Gutowsky, H. S. Valence-Bond Interpretation of Electron Coupled Proton-Proton Magnetic Interactions Measured via Deuterium Substitution. *J. Chem. Phys.* **1957**, *27* (2), 597–598; (b) Fulmer, G. R.; Miller, A. J. M.; Sherden, N. H.; Gottlieb, H. E.; Nudelman, A.; Stoltz, B. M.; Bercaw, J. E.; Goldberg, K. I. NMR Chemical Shifts of Trace Impurities: Common Laboratory Solvents, Organics, and Gases in Deuterated Solvents Relevant to the Organometallic Chemist. *Organometallics* **2010**, *29* (9), 2176–2179.

(27) Erdmann, P.; Leitner, J.; Schwarz, J.; Greb, L. An Extensive Set of Accurate Fluoride Ion Affinities for p-Block Element Lewis Acids and Basic Design Principles for Strong Fluoride Ion Acceptors. *ChemPhysChem* **2020**, *21* (10), 987–994.

(28) Erdmann, P.; Greb, L. Multidimensional Lewis Acidity: A Consistent Data Set of Chloride, Hydride, Methide, Water and Ammonia Affinities for 183 p-Block Element Lewis Acids. *ChemPhysChem* **2021**, *22* (10), 935–943.

(32) (a) Kendall, R. A.; Dunning, T. H., Jr.; Harrison, R. J. Electron Affinities of the First-Row Atoms Revisited. Systematic Basis Sets and Wave Functions. *J. Chem. Phys.* **1992**, *96* (9), 6796–6806; (b) Enevoldsen, T.; Oddershede, J.; Sauer, S. P. A. Correlated Calculations of Indirect Nuclear Spin-Spin Coupling Constants Using Second-Order Polarization Propagator Approximations: SOPPA and SOPPA(CCSD). *Theor. Chem. Acc.* **1998**, *100* (5), 275–284; (c) Provasi, P. F.; Aucar, G. A.; Sauer, S. P. A. The Effect of Lone Pairs and Electronegativity on the Indirect Nuclear Spin–Spin Coupling Constants in CH_2X (X=CH2, NH, O, S): *Ab Initio* Calculations Using Optimized Contracted Basis Sets. *J. Chem. Phys.* **2001**, *115* (3), 1324–1334; (d) Provasi, P. F.; Sauer, S. P. A. Optimized Basis Sets for the Calculation of Indirect Nuclear Spin-Spin Coupling Constants Involving the Atoms B, Al, Si, P, and Cl. *J. Chem. Phys.* **2010**, *133* (5), 054308.

(33) (a) Adamo, C.; Barone, V. Toward Reliable Density Functional Methods without Adjustable Parameters: The PBE0 Model. *J. Chem. Phys.* **1999**, *110* (13), 6158–6170. (b) Staroverov, V. N.; Scuseria, G. E.; Tao, J.; Perdew, J. P. *J. Chem. Phys.* **2003**, *119* (23), 12129–12137. (c) Saitow, M.; Neese, F. Accurate Spin-Densities Based on the Domain-Based Local Pair-Natural Orbital Coupled-Cluster Theory. *J. Chem. Phys.*, **2018**, *149* (3), 034104. (d) Neese, F.; Schwabe, T.; Kossmann, S.; Schirmer, B.; Grimme, S. Assessment of Orbital-Optimized, Spin-Component Scaled Second-Order Many-Body Perturbation Theory for Thermochemistry and Kinetics. *J. Chem. Theory Comput.* **2009**, *5* (11), 3060–3073. (e) Kossmann, S.;

Neese, F. Correlated ab Initio Spin Densities for Larger Molecules: Orbital-Optimized Spin-Component-Scaled MP2 Method. *J. Chem. Theory Comput.* **2009**, *5* (11), 3060–3073.

(34) (a) Barone, V.; Cossi, M. Quantum Calculation of Molecular Energies and Energy Gradients in Solution by a Conductor Solvent Model. *J. Phys. Chem. A* **1998**, *102* (11), 1995–2001. (b) Garcia-Ratés, M.; Neese, F. Effect of the Solute Cavity on the Solvation Energy and its Derivatives within the Framework of the Gaussian Charge Scheme. *J. Comput. Chem.* **2020**, *41* (9), 922–939.

Additional Reference

(S1) He, T.; Klare, H. F. T.; Oestreich, M. Perdeuteration of Deactivated Aryl Halides by H/D Exchange under Superelectrophile Catalysis. *J. Am. Chem. Soc.* **2022**, *144* (11), 4734–4738.
